# Carbene Complexes of Plutonium: Structure, Bonding, and Divergent Reactivity to Lanthanide Analogs

**DOI:** 10.1021/jacs.3c12719

**Published:** 2024-02-01

**Authors:** Jesse Murillo, John A. Seed, Ashley J. Wooles, Meagan S. Oakley, Conrad A. P. Goodwin, Matthew Gregson, David Dan, Nicholas F. Chilton, Andrew J. Gaunt, Stosh A. Kozimor, Stephen T. Liddle, Brian L. Scott

**Affiliations:** † Chemistry Division, Los Alamos National Laboratory, Los Alamos, New Mexico 87545, United States; ‡ Department of Chemistry and Centre for Radiochemistry Research, The University of Manchester, Oxford Road, Manchester M13 9PL, U.K.; § Research School of Chemistry, The Australian National University, Sullivans Creek Road, Canberra, ACT 2601, Australia; ∥ Materials Physics & Applications Division, Los Alamos National Laboratory, Los Alamos, New Mexico 87545, United States

## Abstract

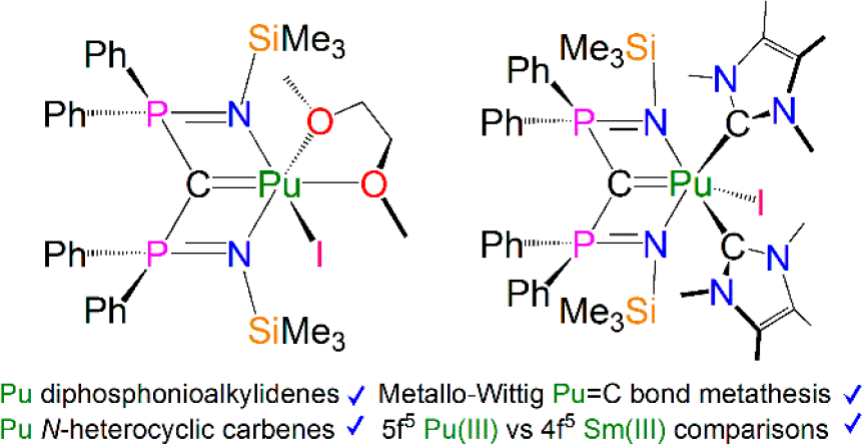

Organoplutonium chemistry was established in 1965, yet structurally authenticated plutonium–carbon bonds remain rare being limited to π-bonded carbocycle and σ-bonded isonitrile and hydrocarbyl derivatives. Thus, plutonium-carbenes, including alkylidenes and N-heterocyclic carbenes (NHCs), are unknown. Here, we report the preparation and characterization of the diphosphoniomethanide-plutonium complex [Pu(BIPM^TMS^H)(I)(μ-I)]_2_ (**1Pu**, BIPM^TMS^H = (Me_3_SiNPPh_2_)_2_CH) and the diphosphonioalkylidene-plutonium complexes [Pu(BIPM^TMS^)(I)(DME)] (**2Pu**, BIPM^TMS^ = (Me_3_SiNPPh_2_)_2_C) and [Pu(BIPM^TMS^)(I)(I^Me4^)_2_] (**3Pu**, I^Me4^ = C(NMeCMe)_2_), thus disclosing non-actinyl transneptunium multiple bonds and transneptunium NHC complexes. These Pu–C double and dative bonds, along with cerium, praseodymium, samarium, uranium, and neptunium congeners, enable lanthanide–actinide and actinide–actinide comparisons between metals with similar ionic radii and isoelectronic 4f^5^ vs 5f^5^ electron-counts within conserved ligand fields over 12 complexes. Quantum chemical calculations reveal that the orbital-energy and spatial-overlap terms increase from uranium to neptunium; however, on moving to plutonium the orbital-energy matching improves but the spatial overlap decreases. The bonding picture that emerges is more complex than the traditional picture of the bonding of lanthanides being ionic and early actinides being more covalent but becoming more ionic left to right. Multiconfigurational calculations on **2M** and **3M** (M = Pu, Sm) account for the considerably more complex UV/vis/NIR spectra for 5f^5^**2Pu** and **3Pu** compared to 4f^5^**2Sm** and **3Sm**. Supporting the presence of Pu=C double bonds in **2Pu** and **3Pu**, **2Pu** exhibits metallo-Wittig bond metathesis involving the highest atomic number element to date, reacting with benzaldehyde to produce the alkene PhC(H)=C(PPh_2_NSiMe_3_)_2_ (**4**) and “PuOI”. In contrast, **2Ce** and **2Pr** do not react with benzaldehyde to produce **4**.

## Introduction

Plutonium (Pu) is the heaviest terrestrial element to occur naturally, though it does so in such small quantities that it is usually regarded as a synthetic element.^1^ While Pu can be viewed as one of the most complex elements in the periodic table and in need of being better understood, investigations into its fundamental properties have been curtailed due to a requirement for specialist handling facilities that satisfy the safety, security, and stewardship challenges associated with this element.^2^ Historically, these restrictions have been worked around by using synthetic surrogates, for example, using lanthanide ions with similar ionic radii or f-electron count or by studying uranium (U) as a close neighbor. However, as synthetic actinide chemistry has developed,^3−7^ it has become clear that there is no accurate surrogate for working with Pu itself given that its substantial relativistic effects cannot be simulated by another element.^8^ Adding further complication to researching Pu is the fact that there is not any metal with the same ionic radius *and* f-electron count with which to make accurate comparisons. Also, the greater effective nuclear charge at Pu compared to U means their redox chemistries are often significantly diverged.^1,2,5^

Where nonaqueous Pu chemistry is concerned,^8−10^ while the area has developed steadily, it is particularly poorly developed with respect to structurally validated complexes. While the first organo-Pu complex, [Pu(C_5_H_5_)_3_], was first reported in 1965,^11^ this compound was only structurally authenticated, as [(η^5^-C_5_H_5_)_2_Pu(μ-η^1^:η^5^-C_5_H_5_)]_*n*_, in 2018,^12^ although [Pu{η^5^-C_5_H_3_(SiMe_3_)_2_}_3_] ^13^ and [K(2.2.2-cryptand)][Pu{η^5^-C_5_H_3_(SiMe_3_)_2_}_3_] ^13^ were reported in 2017. Subsequently, [K(2.2.2-cryptand)][(η^5^-C_5_H_4_SiMe_3_)_3_Pu(η^1^-C_5_H_4_SiMe_3_)],^14^ [(η^5^-C_5_Me_5_)_2_PuI(THF)],^15^ [(η^5^-C_5_H_5_)_3_Pu(CNCy)],^16^ [{η^5^-P(CMeCMe)_2_}_2_Pu(μ-η^6^-CH_2_C_6_H_5_)_2_K],^17^ and [{Pu(η^5^-C_5_H_4_SiMe_3_)_3_}_2_(4,4′-bipy)]^18^ emerged between 2020 and 2023. Following the report of the structure of uranocene in 1969,^19^ [Pu(η^8^-C_8_H_8_)_2_] appeared in 1970,^20^ though it was not structurally characterized until 2020.^14^ However, substituted and reduced derivatives including [Pu{η^8^-C_8_H_6_(SiMe_3_)_2_}_2_] ^21^ and [K(2.2.2-cryptand)][Pu(η^8^-C_8_H_8_)_2_] ^14^ were reported in 2017 and 2020, respectively, with partial X-ray diffraction data reported for [K(L)][Pu(η^8^-C_8_H_8_)_2_] (L = diglyme or (THF)_2_)^22^ in 1974. The only structurally characterized Pu-arene complex is [{C_6_H_4_-1,4-(C_6_H_4_-2-NDipp)_2_}PuI(THF)] (Dipp = 2,6-diisopropylphenyl)^23^ reported in 2023. Though there are prior reports of Pu-alkyls being prepared, for example, [Pu{CH(SiMe_3_)_2_}_3_],^24^ the only structurally authenticated Pu–C σ-bonds are in the aforementioned η^1^-ligated complexes [{η^5^-P(CMeCMe)_2_}_2_Pu(μ-η^6^-CH_2_C_6_H_5_)_2_K],^17^ [K(2.2.2-cryptand)][(η^5^-C_5_H_4_SiMe_3_)_3_Pu(η^1^-C_5_H_4_SiMe_3_)],^14^ and [(η^5^-C_5_H_5_)_2_Pu(μ-η^1^:η^5^-C_5_H_5_)]_*n*_ ^12^ and the isonitrile complex [(η^5^-C_5_H_5_)_3_Pu(CNCy)].^16^ It is hence the case that unambiguously structurally authenticated organo-Pu complexes that provide metal–ligand bond metrics all date from 2017 onward, which stands in contrast to the analogous U and neptunium (Np) organometallics whose structural chemistry spans six and four decades, respectively.^5−10^

The preceding survey emphasizes the dominance of multihapto π-bonded ligands in organo-Pu chemistry.^10^ There are only four formally σ-bonded complexes to date,^12,14,16,17^ and each of those is derived from potentially η^*n*>1^-ligands. It hence follows that there are no Pu–C multiple (alkylidene, Fischer carbene) or dative N-heterocyclic carbene (NHC) bonds, despite the mature nature of carbene chemistry generally. Indeed, where Pu-multiple bonding is concerned more widely, the literature is dominated by plutonyl, PuO_2_^*n*+^, which until recently was mirrored in Np chemistry that was dominated by neptunyl, NpO_2_^*n*+^, and a neptunyl-like bis(imido),^25^ until reports of a Np-mono(oxo)^26^ and diphosphonioalkylidene and NHC complexes of Np ^27^ emerged in 2022. Given that uranium phosphonio-, arsonio-, diphosphonio-, and phosphino-silyl-alkylidene and allenylidene chemistry has grown over four decades,^3−7,28−46^ and that we recently showed that the disphosphoniomethanide {(Me_3_SiNPPh_2_)_2_CH}^1–^ ((BIPM^TMS^H)^1–^), diphosphonioalkylidene {(Me_3_SiNPPh_2_)_2_C}^2–^ ((BIPM^TMS^)^2–^), and NHC C(NMeCMe)_2_ (I^Me4^) ligands were effective at producing rare Np-methanides and the first transuranium-carbon double and Np–C dative bonds,^27^ we turned our attention to establishing Pu congeners.

Here, we report the preparation of a diphosphoniomethanide-Pu complex along with two diphosphonioalkylidene-Pu complexes, one as a 1,2-dimethoxyethane adduct and the other as a bis(I^Me4^) adduct, thus disclosing the first non-actinyl transneptunium multiple bonds and a transneptunium NHC complex. These Pu=C double and Pu–C dative bonds, realized after six decades of organo-Pu endeavor, along with new (Pr and Sm) and previously reported (U, Np, and Ce) diphosphonioalkyldiene complexes provide an opportunity to make lanthanide–actinide and actinide–actinide comparisons between metals with similar ionic radii and isoelectronic 4f^5^ vs 5f^5^ electron-counts within a conserved ligand field over 12 complexes. From this, we elucidate the variance of f- and d-orbital contributions to these M=C double bonds, permitting insight into orbital energy vs spatial overlap components that define the covalency of these M=C bonds, and also to probe the relative magnitudes of interelectronic repulsion (IER), spin–orbit coupling (SOC), and crystal field (CF) effects.

## Results and Discussion

### Synthesis and Reactivity

Our prior work preparing Np-carbenes (diphosphonioalkylidenes and NHCs) emphasized the importance of prechoregraphed, multistep “one-pot” reactions,^27^ a consequence of the small scale of reactions mandated by stewardship of our transuranium stocks, and also the radiological restrictions associated with working in this study with ^239^Pu. That work also demonstrated the necessity of avoiding occluded LiCl and the tendency of transuranium ions to increasingly favor the trivalent state as the 5f-block is traversed left to right, a situation even more pronounced for Pu compared to Np.^1,2,5,8−10^ Hence, we selected [PuI_3_(THF)_4_] ^15,47^ and [Rb(BIPM^TMS^H)] ^48^ as the most eligible starting materials from which to construct Pu-carbene linkages.

**Scheme 1:**
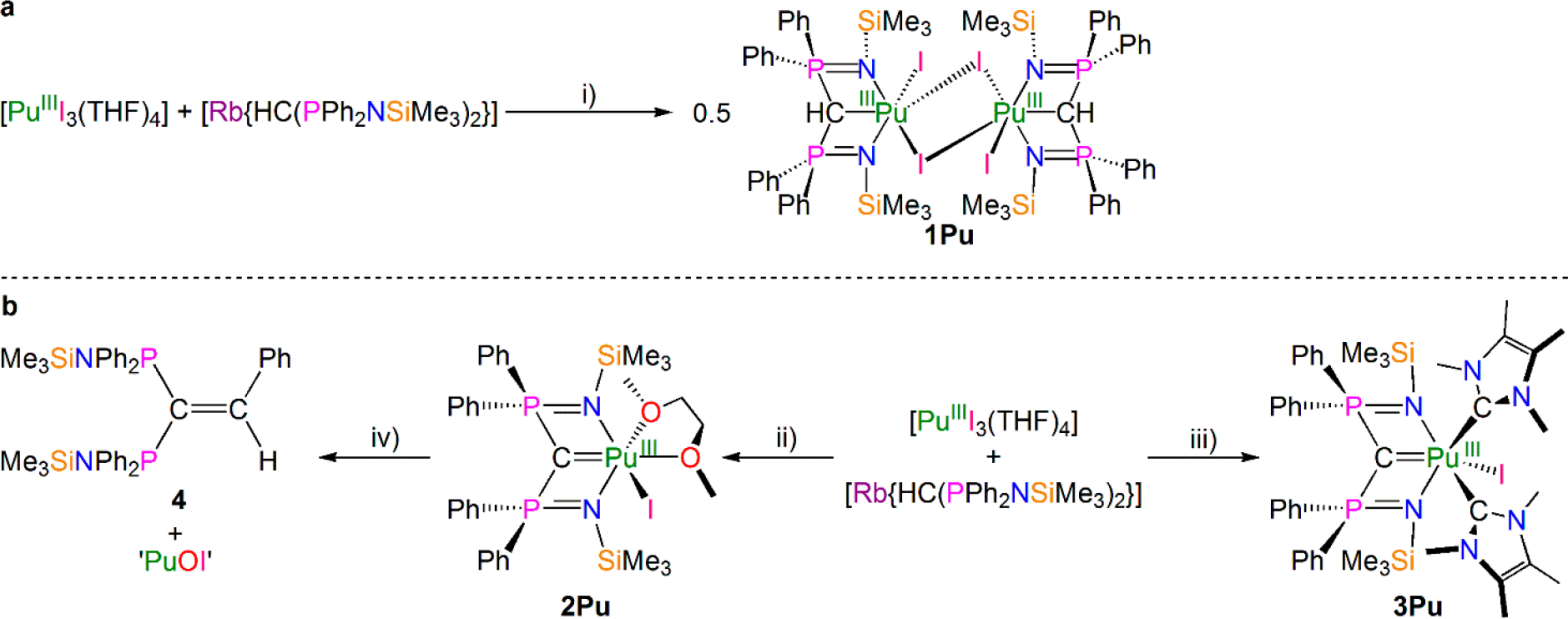
Reactions Producing the Organoplutonium Complexes **1Pu-3Pu** and the Wittig Alkene Product **4** (a) Reaction producing **1Pu**. Reagents and conditions: (i) THF, DME and benzyl potassium, DME/toluene, several filtration steps, benzene recrystallization (-RbI). (b) Reactions producing **2Pu**, **3Pu**, and **4**. Reagents and conditions: (ii) THF, DME and benzyl potassium, DME/toluene recrystallization (-KI); (iii) THF, DME and benzyl potassium, DME and I^Me4^, THF/DME recrystallization (-KI); (iv) C_6_D_6_, PhCHO (-DME). The different outcomes between (i) and (ii) emphasize the importance of using fresh reagents to synthesize transuranium complexes in a small-scale regime.

In our initial attempt to prepare a Pu=C double bond directly, [PuI_3_(THF)_4_] was treated with 1 equiv of [Rb(BIPM^TMS^H)] in THF, with subsequent DME and solid benzyl potassium addition, Scheme 1a. Following multiple filtrations to remove pale amorphous solids, recrystallization from benzene afforded growth conditions for a small quantity of teal crystals of [Pu(BIPM^TMS^H)(I)(μ-I)]_2_·2Benzene (**1Pu·2Benz**) (11% crystalline yield). During our work preparing Np-carbene complexes, we found that use of [Rb(BIPM^TMS^H)] that has been previously exposed to the transuranium glovebox He-atmosphere often resulted in the isolation of products containing (BIPM^TMS^H)^1–^ diphosphoniomethanide even when the reaction conditions had been designed to effect full deprotonation and produce (BIPM^TMS^)^2–^ diphosphonioalkylidene. Since other reactions with air- and moisture-sensitive reagents proceeded as expected in the transuranium glovebox (with acceptable levels of O_2_ and H_2_O in the atmosphere, and solvents verified by [Ti(Cp)_2_(μ-Cl)]_2_ and sodium benzophenone ketyl tests, respectively), we speculated that the [Rb(BIPM^TMS^H)] reagent was being affected by residual solvent vapors in the transuranium glovebox atmosphere; safety constraints imposed by the negative-pressure glovebox mode of operation prevented the atmosphere from being effectively purged of residual solvent vapors.

To avoid the formation of diphosphoniomethanide products, flame-sealed tubes of fresh [Rb(BIPM^TMS^H)] were introduced to the glovebox then opened and used immediately to minimize glovebox solvent/atmospheric exposure. Gratifyingly, this strategy was effective. Treatment of [PuI_3_(THF)_4_] in THF with portion-wise addition of 1 equiv of [Rb(BIPM^TMS^H)], followed by 1 equiv of benzyl potassium and DME afforded, after workup and recrystallization from a toluene/DME solution stored at −35 °C, green crystals of [Pu(BIPM^TMS^)(I)(DME)]·0.5Toluene (**2Pu·0.5Tol**) in 60% yield, Scheme 1b. This successful and consistently reproducible isolation, and yield, of the intended product with doubly deprotonated (BIPM^TMS^)^2–^ diphosphonioalkylidene serves to highlight the nonroutine synthetic challenges and practical constraints associated with small-scale transuranium reactions.

Having established a reliable methodology, and seeking to expand the range of Pu-BIPM^TMS^ complexes, we targeted an NHC adduct. Accordingly, we prepared **2Pu***in situ* and then treated it with 1.75 equiv of I^Me4^;^49^ the use of slightly less than two equivalents of I^Me4^ was in anticipation of the formation of **2Pu** not being quantitative and also to ensure reproducibility by avoiding the formation of imidazolium byproducts. After workup and recrystallization from warm THF solution, green-yellow blocks [Pu(BIPM^TMS^)(I)(I^Me4^)_2_]·0.5Toluene (**3Pu·0.5Tol**) were isolated in 28% yield, Scheme 1b.

In our prior report of Np-carbene complexes,^27^ we showed that [Np(BIPM^TMS^)(I)(DME)] (**2Np**) exhibits metallo-Wittig reactivity with PhCHO, affording the alkene PhC(H)=C(PPh_2_NSiMe_3_)_2_ (**4**),^50^ as evidenced by ^1^H and ^31^P NMR data, and “NpOI”. We were interested to establish whether the Pu=C bond is also capable of effecting metallo-Wittig chemistry and so treated **2Pu** with PhCHO. Indeed, metallo-Wittig reactivity of **2Pu** was found, Scheme 1b, with the characteristic ^1^H and ^31^P NMR spectroscopic data for **4** being observed (Figures S20–S22). This reactivity is consistent with a complex formally containing a Pu=C bond, with multiple bond metathesis presumably producing “PuOI”, though this was not confirmed due to the radiological nature of the work. To benchmark the metallo-Wittig reactivity of trivalent **2Pu** (and **2Np**), we examined the reactivity of the trivalent complexes [M(BIPM^TMS^)(I)(DME)] (M = Ce, **2Ce**;^27^ M = Pr, **2Pr**, see below) with PhCHO. Under the same reaction conditions as for **2Pu**, **2Ce** and **2Pr** do not produce the alkene **4**. Instead, ^31^P NMR spectroscopy (Figures S39 and S40) reveals a complex mixture of products that is consistent with the formation of previously observed methanide species.^51^ The production of methanides in the reactions of **2Ce** and **2Pr** with PhCHO is consistent with prior work with the closely related trivalent complex [Y(BIPM^TMS^)(I)(THF)_2_] that readily engages in C–H activation reactions with a range of ketones.^52^ However, a tetravalent Ce-BIPM^TMS^ complex,^53^ which contains a covalent Ce=C double bond corroborated by a chemical shift anisotropy NMR spectroscopy study,^54^ does engage in metallo-Wittig chemistry.^53^ These observations illustrate an experimental divergence between the reactivity of trivalent Ln=C double bonds^52^ and Ce(IV)– and actinide–carbon double bonds which all execute metallo-Wittig chemistry with ketones and aldehydes, including PhCHO.^27,33−35,50,55^ This hints at macrolevel differences in reactivity that might relate to differing covalency between lanthanide(III)–carbon and lanthanide(IV)– and actinide(III/IV/V/VI)–carbon multiple bonds in this instance.

We previously reported the Ce and Np analogs of **2Pu** and **3Pu**, namely, **2Ce**, **3Ce**, **2Np**, and **3Np**,^27^ and for this study extended that to include the synthesis of the Pr and Sm congeners **2Pr**, **3Pr**, **2Sm**, and **3Sm** (see Supporting Information for further details of all complexes); the 4f^1^ Ce(III), 4f^2^ Pr(III), and 5f^4^ Np(III) complexes provide similarly sized lanthanide and actinide analogs for bond length comparisons to **2Pu** and **3Pu**, and the 4f^5^**2Sm** and **3Sm** complexes provide isoelectronic comparisons to 5f^5^**2Pu** and **3Pu**, respectively. As part of the Pr and Sm work we also prepared the methanide derivatives [M(BIPM^TMS^H)(I)_2_(THF)] (**1M.THF**) and [M(BIPM^TMS^H)(I)_2_(I^Me4^)] (**1M.IMe4**) (M = Pr, Sm); however, since it was not practicable to prepare the Pu-congeners of these complexes, the methanide complexes are not specifically discussed in detail (see Supporting Information for further details). They do, however, provide validation of the formulations of **2M** and **3M** (M = Pr, Sm). Hence, within the context of the previously reported Np and Ce analogs of **2M** and **3M**, alongside computational modeling of the U-congeners, this presents an opportunity to evaluate **2M** and **3M** as a function of actinide and lanthanide identity.

### Solid-State Structures

The solid-state structures of **1Pu**-**3Pu**, **2Pr**, **2Sm**, **3Pr**, and **3Sm** were determined, and selected metrical values and comparisons are summarized in Table 1 along with the corresponding values for **2Np**, **3Np**, **2Ce**, and **3Ce**. Further crystallographic data including those of **1M.THF** and **1M.IMe4** can be found in the Supporting Information (Tables S1–S4).

**Table 1:** Selected Solid-State Metrical and Key IR M=C Vibrational Data for **1Pu**, **2M**, and **3M** (M = Np, Pu, Ce, Pr, Sm)

entry^a^ (CN6 *r*, Å)	M–C_BIPM_ (Å)	ΔM/Pu^b^ (Å)	M–C_IMe4_ (Å)	ΔM/Pu^b^ (Å)	M–N (Å)	ΔM/Pu^b^ (Å)	M–I (Å)	ΔM/Pu^b^ (Å)	M–O (Å)	ΔM/Pu^b^ (Å)	M=C^c^ (cm^–1^)	M=C^d^ (cm^–1^)
**1Pu**	2.732(4)				2.361(3)		3.0249(3)				f	f
(1.00)					2.351(3)		3.1568(3)				f	f
							3.2173(3)					
**2Np**^e^	2.425(7)	0.003			2.414(6)	0.012	3.1065(5)	0.0016	2.524(5)	–0.018	508	f
(1.01)					2.431(6)	0.014			2.636(5)	–0.005	448	f
**2Pu**	2.422(6)				2.402(5)		3.1049(5)		2.542(4)		487	487
(1.00)					2.413(5)				2.641(4)		446	445
**2Ce**^e^	2.477(2)	0.055			2.456(2)	0.054	3.1752(2)	0.0703	2.570(2)	0.028	463	464
(1.01)					2.459(2)	0.046			2.676(2)	0.035	441	441
**2Pr**	2.448(5)	0.026			2.416(4)	0.014	3.1333(5)	0.0284	2.533(5)	–0.009	470	475
(0.99)					2.422(4)	0.009			2.612(4)	–0.029	442	438
**2Sm**	2.381(4)	–0.041			2.387(3)	–0.015	3.0919(3)	–0.0130	2.472(3)	–0.070	463	448
(0.958)					2.396(3)	–0.017			2.548(3)	–0.093	422	415
**3Np**^e^	2.490(6)	0.013	2.677(5)	0.014	2.485(4)	0.007	3.1571(4)	0.0067			473	f
(1.01)			2.751(6)	0.012	2.492(5)	0.003					428	f
**3Pu**	2.477(4)		2.663(5)		2.478(4)		3.1504(4)				472	471
(1.00)			2.739(4)		2.489(4)						426	424
**3Ce**^e^	2.519(2)	0.042	2.737(3)	0.074	2.494(2)	0.016	3.2054(2)	0.0550			474	469
(1.01)			2.806(2)	0.067	2.510(2)	0.021					430	433
**3Pr**	2.492(3)	0.015	2.723(3)	0.060	2.475(2)	–0.003	3.1796(2)	0.0292			473	467
(0.99)			2.784(3)	0.045	2.498(2)	0.009					428	423
**3Sm**	2.444(3)	–0.033	2.667(3)	0.004	2.448(3)	–0.030	3.1329(2)	–0.0175			469	464
(0.958)			2.738(3)	–0.001	2.462(3)	–0.027					412	411

a The metal radii in parentheses are the revised ionic radii reported by Shannon for the metal ions with a coordination number of 6.^60^

b Comparison of the respective lanthanide or Np bond length to the Pu metric; positive Δ values indicate shorter bonds in the Pu complex, whereas negative Δ values indicate that the bond is shorter in the lanthanide or Np complex.

c Key computed M=C vibrations.

d Key experimentally observed IR bands assigned as M=C vibrations.

e Data for the Np and Ce complexes were reported previously.^27^

f Data not available or applicable. There is no comparison of the **1Pu** metrics because it has no isostructural lanthanide or Np analog.

**Figure 1:**
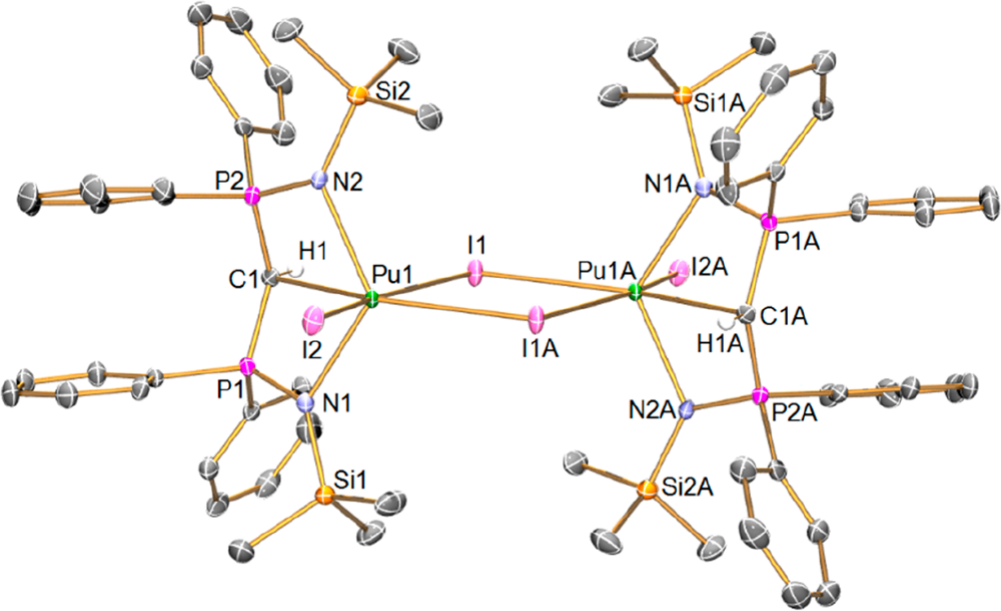
Solid-state molecular structure of **1Pu** at 150 K. Displacement ellipsoids are set at 40% probability, and non-methanide H atoms and lattice solvent are omitted for clarity.

Complex **1Pu**, Figure 1, crystallizes as a halide-bridged dimer; each Pu(III) ion is chelated by a (BIPM^TMS^H)^1–^ ligand, bound in a tridentate manner, and is further coordinated by one terminal iodide and two bridging iodides. Each Pu(III) ion is thus six-coordinate, adopting a distorted octahedral geometry. The P–C_BIPMH_–P angle of 135.8(2)° is typical of the “open book”^51^ conformation of (BIPM^TMS^H)^1–^. The Pu–C_BIPMH_ distance in **1Pu** of 2.732(4) Å is long compared to the sum of the single bond covalent radii of Pu and C (2.47 Å)^56^ but can be compared to the few examples of crystallographically authenticated formal Pu–C σ-bonds that include [K(2.2.2-cryptand)][(η^5^-C_5_H_4_SiMe_3_)_3_Pu(η^1^-C_5_H_4_SiMe_3_)] (2.740(5) Å),^14^ [(η^5^-C_5_H_5_)_2_Pu(μ-η^1^:η^5^-C_5_H_5_)]_*n*_ (2.830(12) and 2.888(12) Å),^12^ [(η^5^-C_5_H_5_)_3_Pu(CNCy)] (2.58(3) Å),^16^ and [{η^5^-P(CMeCMe)_2_}_2_Pu(μ-η^6^-CH_2_C_6_H_5_)_2_K] (2.542(19) and 2.614(19) Å).^17^ The iodide-bridged dimeric structure of **1Pu** does not have any isostructural trivalent f-element (BIPM^TMS^H)^1–^ complexes for direct comparison, but for dimeric trivalent actinide complexes with (BIPM^TMS^H)^1–^ we previously reported [(BIPM^TMS^H)Np(Cl)(μ-Cl)_3_Np{(μ-Cl)Li(DME)(OEt_2_)}(BIPM^TMS^H)],^27^ which exhibits Np–C_BIPMH_ distances of 2.831(4) and 2.838(4) Å. That those Np–C_BIPMH_ distances are ∼0.1 Å longer than the Pu–C_BIPMH_ distances in **1Pu** likely reflects the seven- and six-coordinate natures of the Np and Pu ions in those complexes, respectively. That notion is supported by the Np–C_BIPMH_ distance of 2.753(7) Å in monomeric, six-coordinate [Np(BIPM^TMS^H)(I)_2_(I^Me4^)] (**1Np.IMe4**),^27^ which by the 3σ-criterion is statistically indistinguishable to the Pu–C_(BIPMH)_ distance in dimeric six-coordinate **1Pu**.^1^ The terminal Pu–I distance in **1Pu** of 3.0249(3) Å is consistent with other Pu(III)–I bonds and can be compared to [(η^5^-C_5_Me_5_)_2_PuI(THF)] (3.0353(7) Å),^15^ [PuI_3_(THF)_4_] (3.0712(6)–3.1295(6) Å),^15,57^ [PuI_3_(tachMe_3_)_2_] (tach = 1,3,5=trimethyl-1,3,5-triazacyclohexane, 3.1200(14)–3.1314(12) Å),^58^ [PuI_3_(9S_3_)(NCMe)_2_] (9S_3_ = 1,4,7-trithiacyclononane, 3.0530(6)–3.1275(7) Å),^59^ [PuI_3_{N(CH_2_C_4_H_3_N_2_)_3_}(NCMe)] (3.0982(6)–3.1839(6) Å),^59^ and [{C_6_H_4_-1,4-(C_6_H_4_-2-NDipp)_2_}PuI(THF)] (3.0276(7) Å),^23^ while the bridging Pu–I distances of 3.1568(3) and 3.2173(3) Å have no precedent for direct comparison. The Pu–N distances in **1Pu** are 2.351(3) and 2.361(3) Å, substantially shorter than the seven-coordinate Np–N distances in [(BIPM^TMS^H)Np(Cl)(μ-Cl)_3_Np{(μ-Cl)Li(DME)(OEt_2_)}(BIPM^TMS^H)] (2.451(3)–2.473(4) Å) and **1M.IMe4** (M = Np, 2.423(6), 2.458(6); M = Ce, 2.448(6), 2.493(6); M = Pr, 2.430(9), 2.476(9) Å),^27^ but closer to the Pr–N distances in **1Pr.THF** (2.376(3), 2.412(3) Å); this suggests that the Pu–N distances in **1Pu** are short owing to the six-coordinate nature of the Pu ions combined with the fact two of the three coordinated iodides per Pu center are bridging.

**Figure 2:**
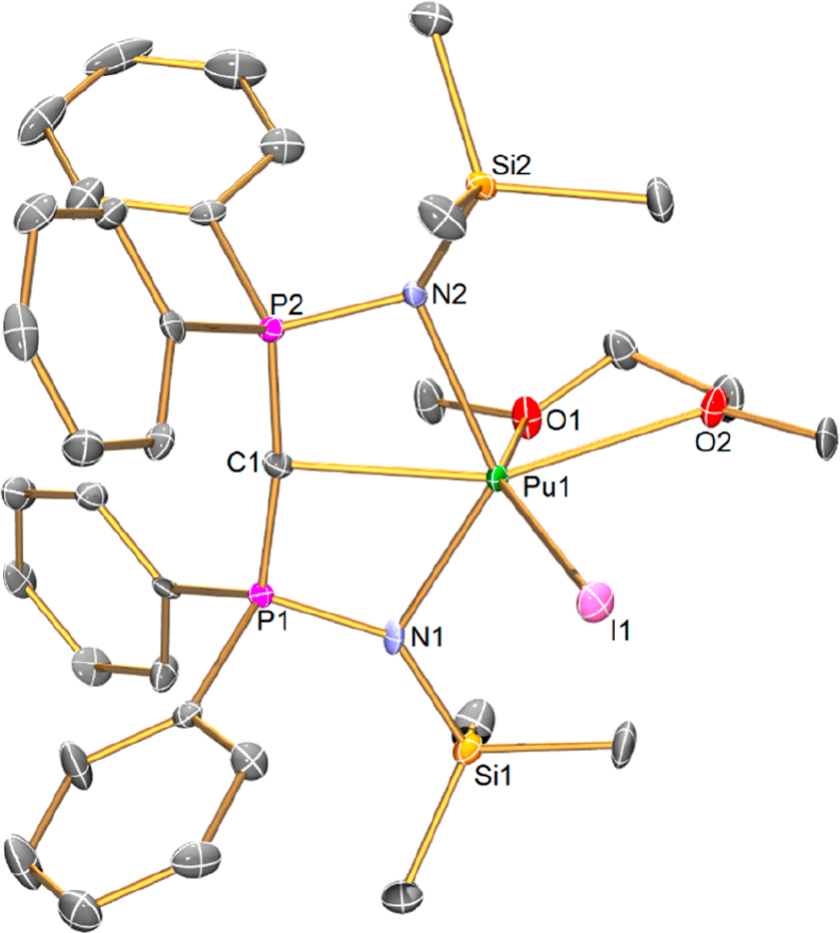
Solid-state molecular structure of **2Pu** at 100 K. Displacement ellipsoids are set at 40% probability, and H atoms and lattice solvent are omitted for clarity.

Complex **2Pu**, Figure 2, is isomorphous to **2Np**, and is monomeric. The Pu(III) ion adopts a distorted octahedral geometry, being chelated by a (BIPM^TMS^)^2–^ ligand, bound in a tridentate manner, and further coordinated by one terminal iodide and the two oxygen atoms of a chelating DME ligand. The C_BIPM_ center is effectively *trans* to one of DME O atoms, with the iodide being *trans* to the other DME O atom; thus the stronger donors are *trans* to the weakest donors in the molecule. This also places the iodide *cis* to the C_BIPM_ center.^42^ The carbene center in **2Pu** adopts a planar (∑∠ = 359.7(4)°) T-shaped geometry with a P–C–P angle of 170.8(4)°, which is statistically indistinguishable from the corresponding angle of 170.4(5)° in **2Np**(27) and close to the values of 167.6(4)° for **2Pr** and 165.2(2)° for **2Sm**. The Pu=C_BIPM_ distance in **2Pu** of 2.422(6) Å is the shortest Pu–C distance of any type to date, the previous being a Pu–CH_2_ bond length of 2.542(19) Å in [{η^5^-P(CMeCMe)_2_}_2_Pu(μ-η^6^-CH_2_C_6_H_5_)_2_K],^17^ and there are no other Pu=C double bonds reported for comparison. However, by the 3σ-criterion the Pu=C distance in **2Pu** is indistinguishable to the Np=C_BIPM_ distance of 2.425(7) Å in **2Np**.^27^ The six-coordinate ionic radii of Ce(III) and Pr(III) are 1.01 and 0.99 Å,^60^ and hence they are good structural comparison points to Pu(III) (1.00 Å).^60^ We note that the Pr=C_BIPM_ distance in **2Pr** (2.448(2) Å) is indistinguishable to the analogous distance in **2Pu** by the 3σ-criterion, but the corresponding **2Ce** (2.477(2) Å) distance is clearly longer than would be predicted based on the ionic radii data. The difference for **2Pu** vs **2Sm** is essentially accounted for by the ionic radii of Pu(III) vs Sm(III) (0.958 Å).^60^ The two P–C_alkylidene_ distances in **2Pu** (1.649(6) and 1.646(6) Å) are statistically the same as those in **2Np**, **2Ce**, and **2Pr**, suggesting that differences in the M=C_BIPM_ distances may reflect a stronger, more covalent M=C_BIPM_ double bond interaction in **2Pu** compared to **2Ce**. The Pu–N distances in **2Pu** (2.402(5) and 2.413(5) Å) are indistinguishable from the Np–N distances in **2Np**,^27^ and, as with the M=C_BIPM_ distances, are indistinguishable from the Pr–N distances in **2Pr**, though they are clearly shorter than the Ce–N distances in **2Ce**; for the latter this is by more than can be accounted for by ionic radius difference alone. The Pu–I distance of 3.1065(5) Å in **2Pu** is unremarkable compared to other Pu(III)–I distances (see above),^15,57−59^ though we note that it is indistinguishable from the corresponding Np–I distance in **2Np**, yet distinctly shorter than the Ce–I and Pr–I distances in **2Ce** an **2Pr**. Lastly, the Pu–O distances in **2Pu** are indistinguishable or only just distinguishable from the Np–O and Pr–O distances in **2Np** and **2Pr**, respectively, but clearly shorter than the Ce–O distances in **2Ce**.

**Figure 3:**
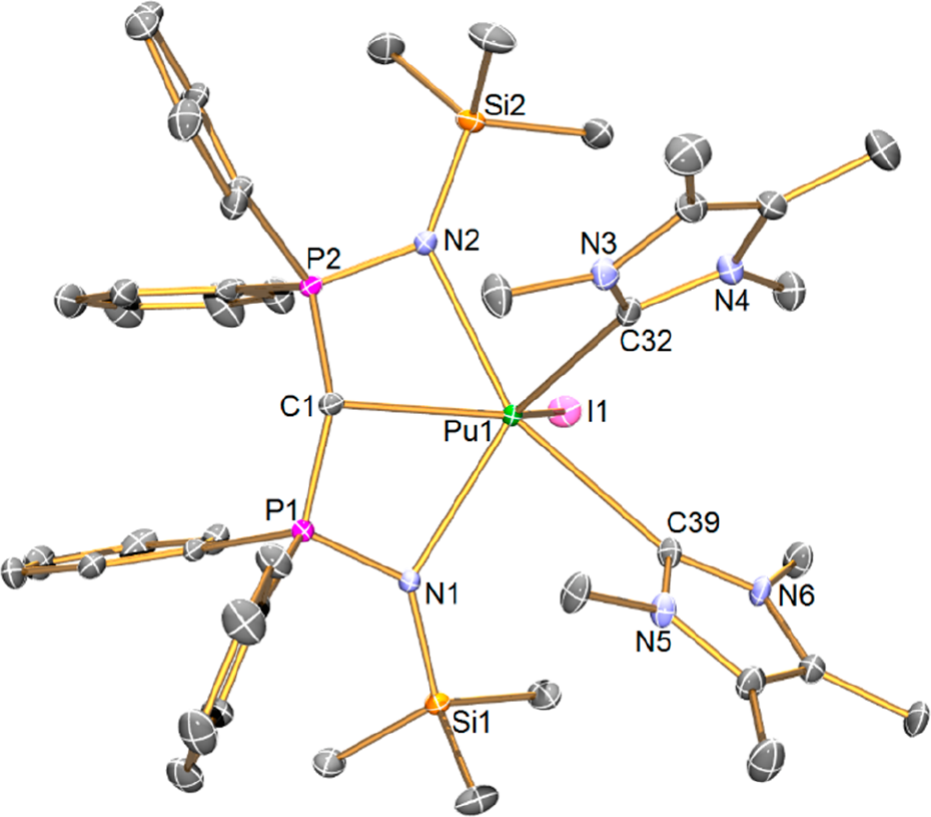
Solid-state molecular structure of **3Pu** at 100 K. Displacement ellipsoids are set at 40% probability, H atoms and lattice solvent are omitted for clarity.

Complex **3Pu**, Figure 3, is isomorphous to **3Np** and is monomeric. The Pu(III) ion in **3Pu** adopts an irregular six-coordinate geometry, being chelated by a (BIPM^TMS^)^2–^ ligand, bound in a tridentate manner, one terminal iodide and two I^Me4^ carbene ligands. The (BIPM^TMS^)^2–^ ligand adopts a more “open-book”^51^ carbene geometry than its planar conformation in **2Pu**, which manifests in the carbene center being pyramidalized (∑∠ = 322.77(19)°) and exhibiting a more bent P–C–P angle of 137.4(3)°. This results in a longer Pu=C_BIPM_ distance in **3Pu** of 2.477(4) Å compared to the corresponding distance in **2Pu**; however by the 3σ-criterion it is within the statistical uncertainty of the corresponding Np=C_BIPM_ distance in **3Np**.^27^ Analogously to the trends found for **2Pu**, the Pu=C_BIPM_ distance is statistically indistinguishable from the corresponding Pr=C_BIPM_ distance of 2.492(3) Å in **3Pr**, but shorter than the Ce=C_BIPM_ distance of 2.519(2) Å and shorter than anticipated from ionic radii differences alone. The Pu=C_BIPM_ distances in **2Pu** and **3Pu** differ by ∼0.05 Å, whereas the corresponding difference for **2Np** and **3Np** is ∼0.07 Å. The two Pu–C_NHC_ distances in **3Pu** (2.663(5) and 2.739(4) Å) are unprecedented examples of Pu–C_NHC_ bonds and hence have no other examples to compare to, nonetheless those distances are within the statistical 3σ-variance of the corresponding Np–C_NHC_ values in **3Np** (2.677(5) and 2.751(6) Å).^27^ However, noting that the **3M**–NHC distances come in pairs of one short and one long, the Pu–C_NHC_ distances in **3Pu** are like-for-like clearly shorter than the corresponding Ce–C_NHC_ (2.737(3) and 2.806(2) Å) and Pr–C_NHC_ (2.723(3) and 2.784(3) Å) metrics in **3Ce** and **3Pr**. Interestingly, the Pu–N_NHC_ distances are statistically indistinguishable from the corresponding Sm–C_NHC_ distances in **3Sm** (2.667(3) and 2.738(3) Å), despite the smaller size of Sm(III) compared to Pu(III),^60^ and when these data are taken together they suggest that the Pu–C_NHC_ bonds may be more covalent than the M–C_NHC_ (M = Ce, Pr, Sm) bonds. The Pu–N distances in **3Pu** (2.478(4) and 2.489(4) Å) are longer (∼0.07 Å) than the corresponding Pu–N distances in **2Pu**, likely reflecting the replacement of DME O-donor atoms with the much more strongly donating C_NHC_ centers. There is little statistically meaningful variance of the Pu–N distances compared to the corresponding Np–N, Ce–N, and Pr–N bonds, and indeed the Sm–N bonds are only marginally shorter from a 3σ-criterion perspective. The Pu–I distance in **3Pu** is 3.1504(4) Å, which is ∼0.05 Å longer than the corresponding Pu–I distance in **2Pu**, again likely reflecting the replacement of DME O-donors with strongly donating C_NHC_ donors. Consistent with the above trends, the Pu–I distance in **3Pu** is indistinguishable to the corresponding Np–I distance in **3Np** ^27^ but shorter than the Ce–I and Pr–I distances in **3Ce** and **3Pr** than ionic radii considerations would predict. In passing, we note that the Ce–I bonds differ by ∼0.03–0.04 Å between **2Ce** and **3Ce**; nonetheless the Pu–I and Np–I distances consistently vary by ∼0.05 Å.

Overall, while it is clear that the metrical data for Np and Pu are often quite similar, for a given **2M** or **3M** comparison, the bonds to Pu are often shorter than would be anticipated for lanthanide congeners based on their respective ionic radii. These data suggest that simple ionic models that adjust solely for ionic radii are not appropriate on their own. While more data are certainly needed, this highlights a limitation associated with using ionic radii to make transuranium actinide–ligand bond distance predictions.

### Spectroscopic Analysis

The ^1^H NMR spectra of **1Pu**-**3Pu** (Figures S12–S19) span the ranges −7 to 8, −1 to 8, and −2 to 8 ppm, respectively. These ranges are surprisingly similar, and for **2Pu** and **3Pu** are notably narrower chemical shift ranges compared to the chemical shift ranges of **2Np** and **3Np** (∼25 ppm, which also in appearance exhibit qualitatively broader resonances)^27^ reflecting the 5f^5^ nature of Pu(III) compared to 5f^4^ Np(III). The ^31^P{^1^H} chemical shifts of **1Pu**-**3Pu** are −83.4, −128.4, and −141.9 ppm, respectively, and while the value for **1Pu** is similar to **1Np.IMe4** (δ_31P_ −54.6 ppm),^27^ the values for **2Pu** and **3Pu** are rather different to **2Np** and **3Np** (δ_31P_ −789 and −740 ppm, respectively),^27^ reflecting the different 5f^*n*^ counts of Np and Pu. However, we note that conversion of (BIPM^TMS^H)^1–^ to (BIPM^TMS^)^2–^ results in shifts to lower frequencies for both Np- and Pu-BIPM^TMS^ complexes.

The Evans method effective magnetic moments of **1Pu**-**3Pu** were determined (Table S5), yielding values of 3.65, 1.35, and 0.90 μ_B_ per molecule, respectively, which, noting that **1Pu** is dinuclear (1.83 μ_B_ per Pu ion), are in fair agreement with the Evans method effective magnetic moments of mononuclear [PuI_3_(THF)_4_] (1.17 μ_B_) and [(η^5^-C_5_Me_5_)_2_PuI(THF)] (0.97 μ_B_).^15^ Those values are consistent with the fact that the predicted μ_eff_ value for ^6^H_5/2_ f-element ions is 0.85 μ_B_, although magnetic moments of ∼1.4 μ_B_ are typically found because the ground state is not well-isolated from low-lying paramagnetic states, and the resulting mixing increases the observed effective magnetic moments. We note in passing that the effective magnetic moments are smallest for **3Pu** and [(η^5^-C_5_Me_5_)_2_PuI(THF)],^15^ which might be predicted to have some of the strongest CFs, but the effective magnetic moments of more complexes need to be obtained to determine whether this constitutes a real trend.

Analytical frequency calculations on geometry optimized models of **2M** and **3M** (M = Np, Pu, Ce, Pr, Sm), Table 1, reveal for each series two principal vibrations that correspond to M=C stretches. The first, at higher energy, is M=C bond contraction with simultaneous M–N bond lengthening and vice versa, whereas the second, lower energy, stretch is isolated M=C contraction and lengthening without any other significant vibrations in the molecule. The computed values are in good agreement with the experimentally observed IR spectra of **2Pu** and **3Pu** (Figures S41 and S42), providing confidence in the DFT calculations. For the **3M** series the energies of the stretches are very close to each other, likely reflecting the pyramidalized nature of the alkylidene centers, and hence the stretches are less responsive to the nature of the metal. However, larger differences are found for the **2M** series, likely reflecting the optimal bonding scenario, and here the differences are significant enough that they can be ordered as Pu > Pr > Ce ∼ Sm. This is in agreement with the bond length data above, and also the bonding analysis that is revealed by the DFT analysis (see below). Unfortunately, experimental IR data for previously reported **2Np** and **3Np** were not obtained, though being part of respective series there can be confidence in the DFT data for those two complexes. Interestingly the calculated vibrational data for **2Np** and **3Np** suggest that the Np=C bonds are stronger than the corresponding Pu=C bonds, which is supported by the DFT analysis (see below). Lastly, the IR data suggest that the Pu=C bond in **2Pu** is stronger than the analogous bond in **3Pu**, in-line with the structural data, binding modes, and DFT bonding analysis.

**Figure 4:**
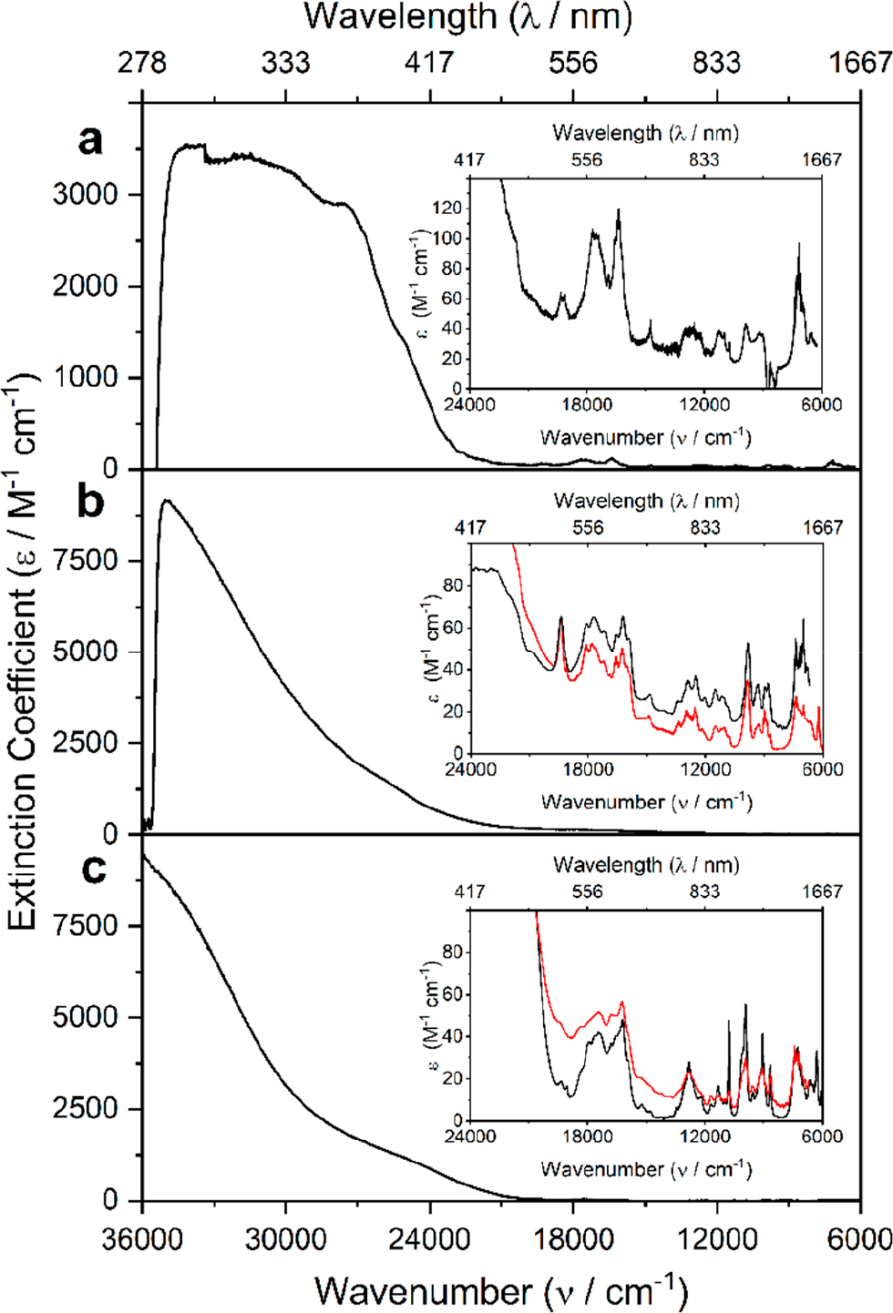
Solution (black) and solid-state (red) UV/vis/NIR spectra for **1Pu**-**3Pu**: (a) **1Pu**, (b) **2Pu**, (c) **3Pu**. The inset of each spectrum highlights the respective vis/NIR region.

The UV/vis/NIR solution spectra of **1Pu**-**3Pu**, Figure 4, are consistent with their Pu(III) formulations, and exhibit signature f−f transition features in the NIR regions observed for other Pu(III) complexes, for example [PuI_3_(THF)_4_],^15^ [(η^5^-C_5_Me_5_)_2_PuI(THF)],^15^ and Pu^3+^ in 1 M perchloric acid solutions.^61^ Specifically, Pu(III) complexes tend to exhibit a characteristic pattern of absorptions at ∼19 000 cm^–1^, a pair of features centered around a barycenter at ∼17 000 cm^–1^, ∼15 000 cm^–1^, and several features centered at ∼12 000 cm^–1^, ∼9000, and ∼7000 cm^–1^; **1Pu**-**3Pu** all exhibit those features with extinction coefficients of 30–120 M^–1^ cm^–1^. For **1Pu**-**3Pu**, strong absorption bands begin at ∼24 000 cm^–1^ and extend well into the UV region. These bands have extinction coefficients of ∼9000 M^–1^ cm^–1^ for **2Pu** and **3Pu** and are assigned as LMCT bands; for **1Pu**, the broad LMCT band is weaker at ∼3500 M^–1^ cm^–1^, likely reflecting that **1Pu** is a methanide complex compared to the carbene formulations of **2Pu** and **3Pu**. Compared to U(III) and Np(III), whose f–d transitions tend to occur around 15 000–22 000 cm^–1^,^7^ the Laporte allowed f–d transitions of Pu(III) would be expected to occur above 24 000 cm^–1^ due to the increasing f–d energy gap as the actinide series is traversed left to right.^15^ Indeed, **1Pu**-**3Pu** shoulder absorptions at ∼27 600, 25 500, and 25 000 cm^–1^, respectively, with extinction coefficients of 1400–1600 M^–1^ cm^–1^, are observed, and these are assigned as f–d transitions. As the ligand fields of **1Pu**-**3Pu** change, becoming more electron-rich, the f–d energy gap would, simplistically, be expected to become smaller. Although the bands attributed to f–d transitions for **2Pu** and **3Pu** are on the edge of strong LMCT bands, it does indeed appear that the f–d bands are energetically ordered **1Pu** > **2Pu** > **3Pu**, though the differences are relatively slight. Where the f–f transitions for **1Pu**-**3Pu** are concerned, each group of features changes quite substantially between the three complexes, so it is not possible to comment on whether the f–f bands are broader or sharper, implying more or less vibronic coupling and covalency, respectively. Since the structure of the f-manifold would be expected to be rather complex in the SOC regime of Pu(III), this is probed with *ab initio* methods (see below). Lastly, to probe whether the solution UV/vis/NIR data for **2Pu** and **3Pu**, and hence the solution structures, are representative of the solid-state structures, we collected solid-state UV/vis/NIR spectra on single crystals of **2Pu** and **3Pu** (Figures S57 and S62); the low yield precluded acquisition of data for **1Pu**. For both **2Pu** and **3Pu**, there is excellent agreement between the solution and solid-state UV/vis/NIR spectra, suggesting that there are no significant electronic changes (and by extension, speciation) upon dissolution of **2Pu** or **3Pu** in toluene or THF, respectively.

### Electronic Structure Analysis

Given that **1Pu** does not have a structural congener with other f-metals, the following discussion focuses on **2Pu** and **3Pu**, since these complexes have structurally authenticated Np, Ce, Pr, and Sm analogs. To probe the electronic structures of **2Pu** and **3Pu**, and the Pr and Sm congeners, DFT calculations were performed (Tables S6–S14). DFT data for **2Np**, **2Ce**, **3Np**, and **3Ce** were reported previously^27^ and are included here to aid comparisons of the Pu, Pr, and Sm calculations (see Supporting Information for full details). We also include the previously calculated data for **2U** and **3U**;^27^ while they are not experimentally realized complexes, there can be confidence in their electronic structure analysis because they are internally validated by the Pu, Np, Ce, Pr, and Sm calculations and align with computational studies on experimentally realized uranium(III/IV/V/VI) analogs.^33,34,41^ By making these comparisons, Table 2, we relate metals that are of similar ionic radii and make direct isoelectronic 5f^5^–4f^5^ comparisons. In general the optimized gas-phase bond lengths and angles agree well with the experimental solid-state metrics, and the analytical frequency calculations derived from those optimized coordinates are consistent with the experimental IR data. Thus, the calculations can be considered to represent reliable qualitative models of the electronic structures examined.

**Table 2:** Selected Computed Properties for **2M** and **3M** (M = U, Np, Pu, Ce, Pr, Sm)

	bond and indices		charges^d^		spin densities^e^		NBO M–C σ-bond component (%)^f^			NBO M–C π-bond component (%)^f^			QTAIM^h^	
entry^a^	bond^b^	BI^c^	M	C	M	C	M^g^	C^g^	M s/p/d/f	M	C	M s/p/d/f	ρ	ε
**2U**^i^	U=C_BIPM_	1.28	1.57	–2.00	3.28	–0.04	14	86	4/1/42/53	13	87	0/0/40/60	0.08	0.20
**2Np**^i^	Np=C_BIPM_	1.40	1.54	–1.96	4.36	–0.07	17	83	4/1/32/63	14	86	0/0/38/62	0.08	0.21
**2Pu**	Pu=C_BIPM_	0.79	1.48	–1.94	5.40	–0.13	20	80	3/1/26/70	15	85	0/0/36/64	0.08	0.10
**2Ce**^i^	Ce=C_BIPM_	1.05	1.32	–1.82	1.07	–0.01	10	90	1/1/61/37	8	92	0/0/65/35	0.07	0.22
**2Pr**	Pr=C_BIPM_	0.80	1.30	–1.73	2.15	–0.06	12	88	1/1/48/50	10	90	0/0/49/51	0.07	0.22
**2Sm**	Sm=C_BIPM_	0.83	1.17	–1.63	5.56	–0.35	23	77	1/1/18/80	25	75	0/0/16/84	0.07	0.16
**3U**^i^	U=C_BIPM_	1.17	1.62	–1.67	3.08	–0.04	14	86	10/1/46/43	10	90	0/1/50/49	0.08	0.17
	U←C_NHC_	0.77		–0.50		–0.03	0	100					0.05	0.03
	U←C_NHC_	0.81		–0.48		–0.03	0	100					0.05	0.03
**3Np**^i^	Np=C_BIPM_	1.20	1.51	–1.64	4.22	–0.05	15	85	9/1/39/51	10	90	0/1/43/56	0.08	0.18
	Np←C_NHC_	0.69		–0.46		–0.03	0	100					0.04	0.03
	Np←C_NHC_	0.65		–0.44		–0.03	0	100					0.05	0.03
**3Pu**	Pu=C_BIPM_	0.59	1.38	–1.60	5.26	–0.05	15	85	10/1/36/53	10	90	1/1/39/59	0.07	0.10
	Pu←C_NHC_	0.33		–0.41		–0.03	0	100					0.05	0.02
	Pu←C_NHC_	0.28		–0.40		–0.03	0	100					0.04	0.02
**3Ce**^i^	Ce=C_BIPM_	0.96	1.29	–1.53	1.01	–0.01	9	91	7/1/65/27	7	93	2/1/60/37	0.07	0.19
	Ce←C_NHC_	0.52		–0.38		–0.01	0	100					0.04	0.03
	Ce←C_NHC_	0.58		–0.36		–0.01	0	100					0.04	0.03
**3Pr**	Pr=C_BIPM_	0.68	1.19	–1.51	2.13	–0.04	10	90	7/1/57/35	8	92	3/1/56/40	0.07	0.21
	Pr←C_NHC_	0.35		–0.32		–0.01	0	100					0.04	0.08
	Pr←C_NHC_	0.31		–0.33		–0.01	0	100					0.04	0.02
**3Sm**	Sm=C_BIPM_	0.71	1.06	–1.42	5.50	–0.28	17	83	5/1/27/67	22	78	2/0/14/84	0.06	0.14
	Sm←C_NHC_	0.26		–0.28		–0.03	0	100					0.04	0.02
	Sm←C_NHC_	0.23		–0.30		–0.02	0	100					0.03	0.04

a All compounds geometry optimized without symmetry constraints at the BP86 TZP/ZORA (all-electron) level.

b M–C bond: M=C_BIPM_ = diphosphonioalkylidene of (BIPM^TMS^)^2–^; M←C_NHC_ = I^Me4^ NHC carbene.

c Nalewajski–Mrozek bond indices.

d MDC_q_ charges.

e MDC_m_ spin densities. Note that a positive value indicates an excess of spin density and a negative value the loss of spin density.

f Natural bond orbital (NBO) analysis.

g Values of 0% for the total M contribution to the M–C bond means the M contribution is below the cutoff threshold of NBO (5%).

h Quantum theory of atoms in molecules (QTAIM) bond critical point topological electron density (ρ) and ellipticity (ε) analysis.

i Data for the Np and Ce complexes were reported previously.^27^

**Figure 5:**
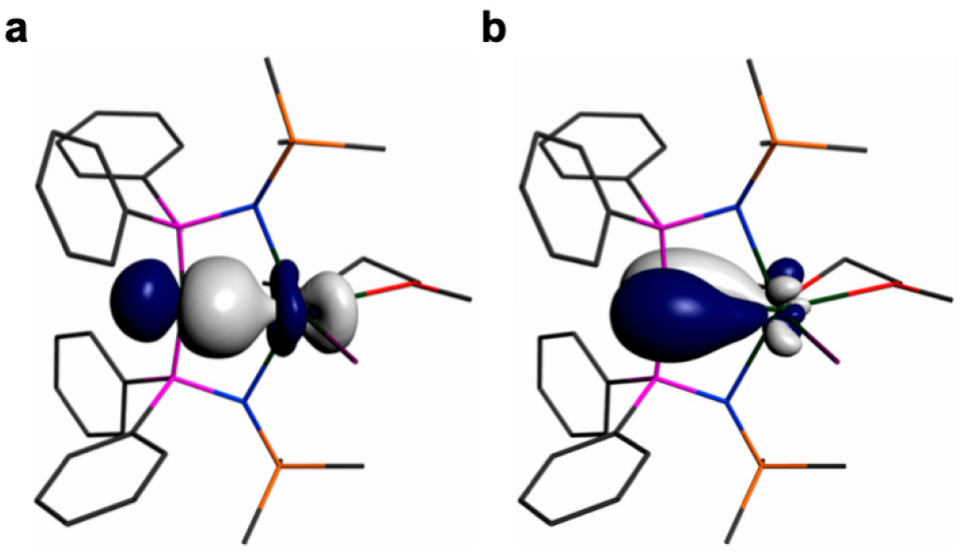
NBO representations of the Pu=C_BIPM_ bonding interactions in **2Pu**: (a) Pu=C_BIPM_ σ-bond; (b) Pu=C_BIPM_ π-bond.

Addressing **2Pu** and **3Pu** together, the computed Pu (1.48, 1.38) and C_BIPM_ (−1.94, −1.60) charges are consistent with the presence of formal Pu(III) ions and (BIPM^TMS^)^2–^ dianions. The computed Pu spin densities (5.40, 5.26) are consistent with 5f^5^ ions with net donation of charge from the ligands, which is corroborated by the negative (−0.13, −0.05) C_BIPM_ spin densities. Where the Pu=C bonds are concerned, NBO analysis reveals σ- and π-bonds for **2Pu**, Figure 5, that are 20 and 15% Pu character, respectively, and within those components 5f- dominates over 6d-contributions (5f/6d: 70/26 and 64/36%). NBO analysis of **3Pu** also finds σ- and π-bonds (Figure S81), with 15 and 10% Pu character, and within those components the 5f-character still dominates over 6d-components (5*f*/6d: 53/36 and 59/39), though less so than in **2Pu**. Thus, the NBO analysis clearly reveals the presence of σ- and π-bonding components in **2Pu** and **3Pu**, although the pyramidalization of the carbene in **3Pu** renders the overlap not as efficient as the T-shaped carbene in **2Pu** which is optimally aligned for double bonding. Considering the Pu ions in **2Pu** and **3Pu** are Pu(III), the Pu % contributions to the Pu=C double bonds are significant. However, the Pu=C bond orders (0.79, 0.59) suggest the presence of very polarized double bonds,^62^ that is each bond order component is a low subinteger value. The topological quantum theory of atoms in molecules (QTAIM) data (ρ: 0.08, 0.07; ε: 0.10, 0.10) reveal low bond critical point electron densities (ρ), and the ellipticity (ε) values are only just entering the double bond range. This suggests that the Pu=C bonds in **2Pu** and **3Pu** should be considered to be borderline double bond interactions rather than full double bonds. Where the Pu–C_IMe4_ bonds of **3Pu** are concerned, those bonds are evidently highly electrostatic, reflecting the formal dative nature of the carbene to Pu donation, and as such the Pu–C_IMe4_ bond orders are low (av ∼0.31).

Next, it is instructive to compare the 5f members of **2M** and **3M** (M = U, Np, Pu). The computed charges and spin densities are as would be expected for these 5f^3^, 5f^4^, and 5f^5^ complexes, respectively, so we focus on the NBO, bond order, and QTAIM metrics. For each **2M** and **3M** series the bond orders follow the trend Np > U > Pu. In contrast, for the **2M** compounds the metal contributions to the M=C bonds is ordered Pu > Np > U for the σ- and π-bonds. The situation is less clear for the **3M** series, where for the M=C σ-bonds are ordered Pu ∼ Np > U and the π-bonds are all similar, and this likely reflects that the M=C bonds are not optimally aligned so they are less responsive to the nature of the coordinated metal. However, irrespective of how varied the Pu % contributions are to the M=C bonds in **2M** and **3M**, it is clear that the 5f/6d compositions vary; specifically, on moving from U to Np to Pu, all the σ- and π-bonds exhibit decreasing 6d-character and corresponding increasing 5f-contributions, reflecting that as the actinide series is traversed left to right the 5f- and 6d-orbitals fall and increase in energy, respectively.^63−68^ The QTAIM ρ and ε values show little variation for the **2M** and **3M** complexes overall, except for the ε values, where according to this topological metric U and Np have better developed double bond interactions compared to Pu.

Extending the above analysis to include the Ce, Pr, and Sm members, for both the **2M** and **3M** series the Ce=C bond order is less than the corresponding U=C and Np=C bonds but greater than the Pu=C congeners. For the **2M** series the Pr=C and Sm=C bond orders are then essentially the same as the Pu=C bond order, and for the **3M** series the Pr=C and Sm=C bond orders are slightly larger than the analogous Pu=C bond order. Nevertheless, overall the bond orders can be arranged as Np > U > Ce > Pu ∼ Pr ∼ Sm. However, NBO analysis reveals a more complex picture. For the **2M** compounds, the Ce=C and Pr=C have, like-for-like, smaller metal contributions to the σ- and π-components of the M=C bonds than U, Np, and Pu; however, the Sm=C σ- and π-components have the largest metal contributions of all the **2M** complexes, with Pu next largest, producing the trend Sm > Pu > Np > U > Pr > Ce. We note that the 4f and 5f metals display parallel trends, that is for M % Pu > Np > U and Sm > Pr > Ce, so the heavier metals have larger M % contributions within a 4f or 5f group. We also note that as found for the 5f metals, the 5d-contributions decrease and the 4f-components increase on moving from Ce to Pr to Sm, which again likely reflects the 4f- and 5d-orbital energies falling and increasing, respectively, left to right in the lanthanide series. The QTAIM ρ values are fairly invariant across the **2M** series, being slightly lower for the 4f vs 5f metals, however a clear difference can be found in the ε values, which are substantial, indicating well-developed double bond interactions for U, Np, Ce, and Pr, and lower for Pu and Sm suggesting that those M=C double bond interactions are less well-developed. Turning to the **3M** series of complexes, in essence the same patterns are observed, though with less variability due to the suboptimal M=C bonding geometry.

The bonding picture that emerges from the above analysis is clearly more complex than the traditional picture of the bonding of lanthanides are ionic and early actinides being covalent and becoming more ionic left to right. However, it is clear that the more f-character, and less d-component, there is in these M=C bonds the greater the M % contribution is, but conversely the lesser the bond order and bond ellipticity are. Traversing the f-blocks, the 4/5f-orbital energies fall with increasing atomic number and d-orbital energies increase.^63−68^ Hence, with respect to the likely carbene energies, the data imply that the orbital energy matching (orbital mixing) increases, and gives greater f-orbital contributions as atomic number increases, however the spatial overlap is reduced. Based on the structural data the implication is that the orbital energy/spatial overlap tension is just tipped to a net increase of covalency for Pu, compared to the lanthanides, and this is consistent with the outcomes of the reactions with PhCHO above, but this is evidently based on superior orbital energy matching but poorer spatial overlap of metal–ligand orbitals. Thus, when considering how covalent the Pu=C bonds are compared to U, Np, Ce, Pr, and Sm analogs it would seem to be that they are most covalent except for Sm on orbital energy matching grounds. When considering just the actinides, it would seem that the orbital energy matching increases moving from U to Np to Pu. However, the spatial overlap increases when moving from U to Np then decreases Np to Pu. The result is then that Np is the most covalent overall.

In order to provide further insight into the electronic structures of **2Pu** and **3Pu** that are experimentally benchmarked and validated, we turned to modeling the UV/vis/NIR data using complete active space self-consistent field spin–orbit (CASSCF-SO) calculations (Tables S15–S30). Furthermore, since we have experimentally realized **2Sm** and **3Sm**, and noting that their UV/vis/NIR spectra are significantly simpler than those of **2Pu** and **3Pu**, this presented an opportunity to directly probe and compare the interplay of CF, SOC, and IER for isostructural 4f^5^ and 5f^5^ complexes. The corresponding calculations for **2Pr** and **3Pr** were also undertaken to provide further benchmarking of the calculations, though poor solubility prevented reliable experimental UV/vis/NIR data for **3Pr** being obtained. In general, absorption features are computed to ∼500 cm^–1^ of experiment, and the relative energy spacings are overall reproduced by the calculations.

The f^5^ free-ion configuration for Pu(III) and Sm(III) defines low-lying ^6^H and ^6^F terms, and higher lying ^4^G, ^4^F, ^4^I, etc. terms. For **2Sm**, **3Sm**, **2Pu**, and **3Pu**, CASSCF(5,7)-SO calculations give a ^6^H ground term as expected (Tables S18–S23). As both Pu(III) and Sm(III) are Kramers ions, we find doubly degenerate CF states. The ground states for **2Sm**, **3Sm**, **2Pu**, and **3Pu** all exhibit *m*_*J*_ mixing owing to their low symmetry, and to compare between compounds we quantize the projection of the total angular momentum along the M-I axis. In **2Sm** and **2Pu**, the ground states are dominated by |*m*_*J*_| = 5/2, whereas **3Sm** and **3Pu** exhibit a much higher degree of mixing in their ground states. The ground state of **3Sm** has a 43% contribution from |*m*_*J*_| = 1/2 and a smaller contribution from |*m*_*J*_| = 3/2 (25%), while **3Pu** has equal 26% contributions from |*m*_*J*_| = 5/2 from |*m*_*J*_| = 1/2. As expected from the low symmetry, the ground state *g*-values are rhombic (Tables S25–S30), affording *g*-values of *g*_1,2,3_ = 0.07, 0.58, 0.90 and 0.01, 0.26, 0.71 for **2Sm** and **3Sm**, respectively, and *g*_1,2,3_ = 0.19, 0.47, 0.67 and 0.09, 0.36, 0.55 for **2Pu** and **3Pu**, respectively (Figures S92 and S93).

In the case of the isoelectronic 4f and 5f ions, it is of considerable interest to compare the relative strengths of IER, SOC, and CF splitting. Here, we define the IER as the energy difference between the barycenters of the ^6^H and ^6^F terms, the SO splitting as the energy difference between the barycenters of the ^6^H_5/2_ and ^6^H_7/2_ multiplets, and the CF splitting as the total splitting range of the ^6^H_5/2_ multiplet. We find the SO and CF splitting for the Pu(III) complexes are approximately double the values for the Sm(III) complexes (Table 3), since there are more significant relativistic effects and larger radial extension of the 5f- vs 4f-orbitals, respectively. However, we find that the IER is approximately 50% larger for Sm(III) than Pu(III): this likely arises as a consequence of the more spatially confined nature of 4f-electrons compared to their 5f counterparts.

**Table 3:** Interelectron Repulsion (IER), Spin Orbit (SO), and Crystal Field (CF) Splitting (cm^–1^) of **2M** and **3M** (M = Pu, Pr, Sm)

	CASSCF-SO			MS-CASPT2-SO			XMS-CASPT2-SO		
entry^a^	IER	SO	CF	IER	SO	CF	IER	SO	CF
**2Sm**	6647	874	407	5150	874	306	5741	784	434
**2Pu**	4499	1687	763	2468	1687	575	3238	1687	770
**3Sm**	6651	874	538	5226	874	563	5835	874	560
**3Pu**	4504	1685	968	2616	1685	1002	3371	1685	1166
**2Pr**	5848	2008	710	4469	2008	612	4694	2008	844
**3Pr**	5857	2009	834	4437	2009	931	4874	2009	942

a All calculations performed at crystal structure geometry.

For Pr(III) the 4f^2^ configuration defines a ground ^3^H term with a low-lying ^3^F term, and a proximal ^1^G term, while ^1^D, ^3^P, and ^1^I terms are much higher in energy; our CASSCF(2,7)-SO calculations considered the lowest three terms, finding a ground ^3^H_4_ multiplet as expected for both **2Pr** and **3Pr** (Tables 3 and S15–S17). Owing to the non-Kramers nature of the 4f^2^ configuration and the low-symmetry of the complexes, the CF removes the degeneracy of all states in the ^3^H_4_ multiplet.

**Figure 6:**
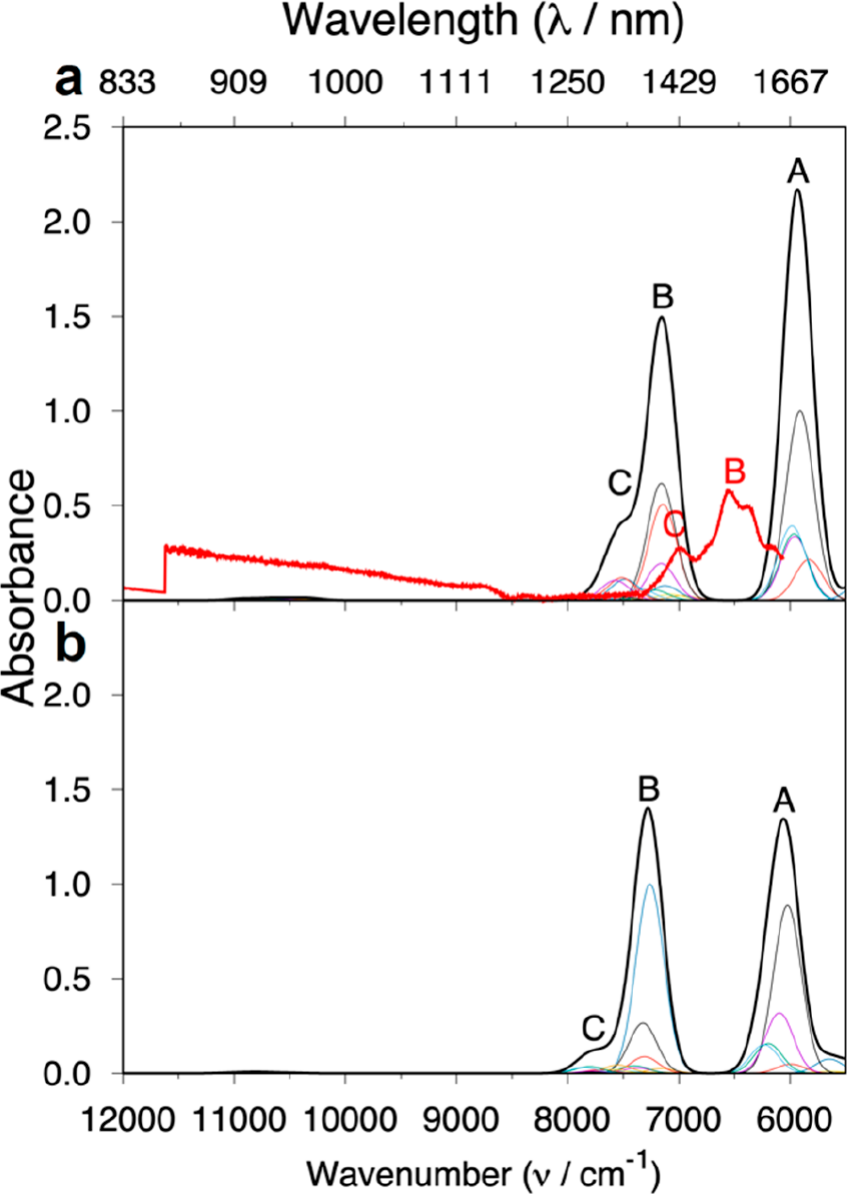
Experimental (red) and computed MS-CASPT2-SO (black) UV/vis/NIR data for **2Pr** and **3Pr**: (a) **2Pr**; (b) **3Pr** (no experimental data available). Individual transitions (rainbow, below black) and total absorption spectrum were Gaussian-broadened with a half-width of 250 cm^–1^.

**Figure 7:**
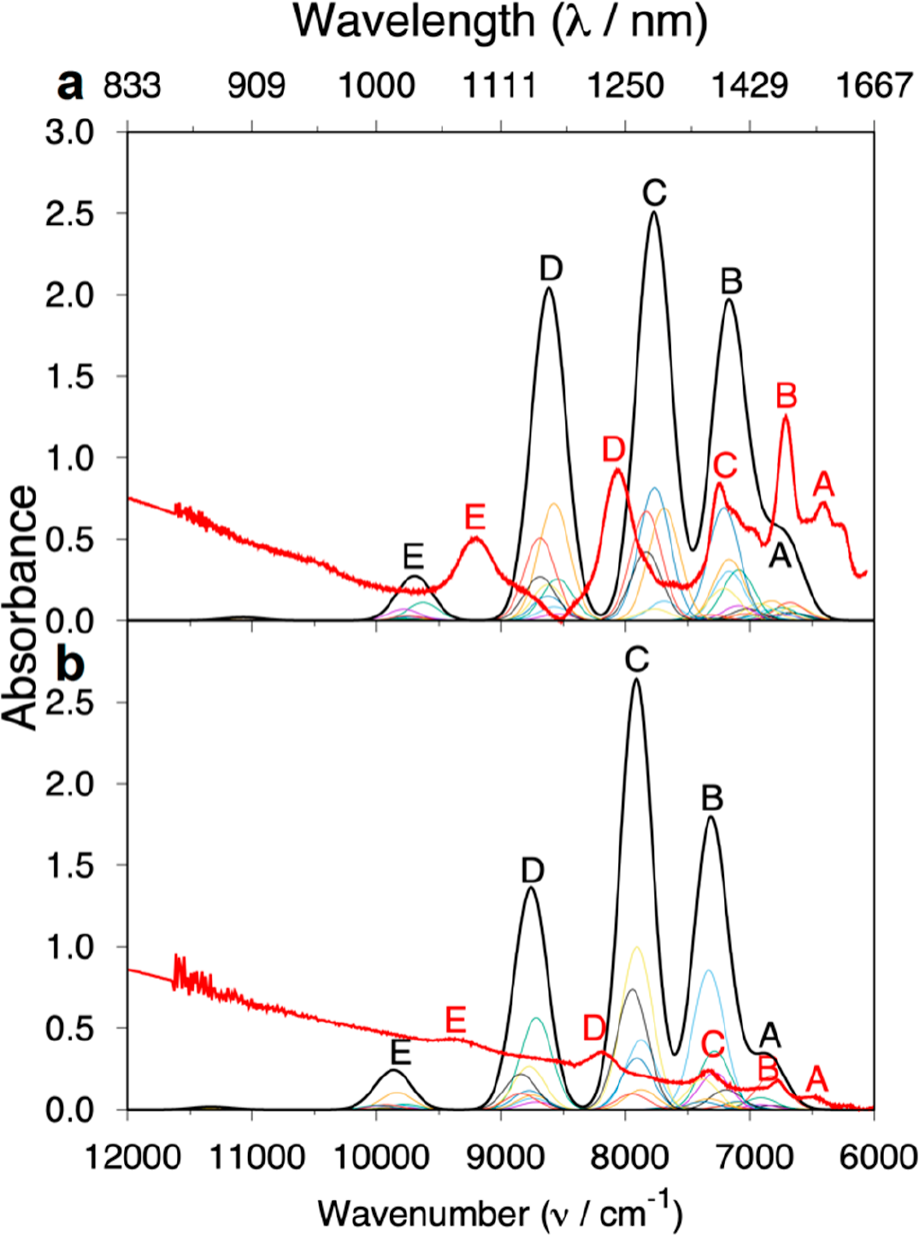
Experimental (red) and computed MS-CASPT2-SO (black) UV/vis/NIR data for **2Sm** and **3Sm**: (a) **2Sm**; (b) **3Sm**. Individual transitions (rainbow, below black) and total absorption spectrum were Gaussian-broadened with a half-width of 250 cm^–1^.

**Figure 8:**
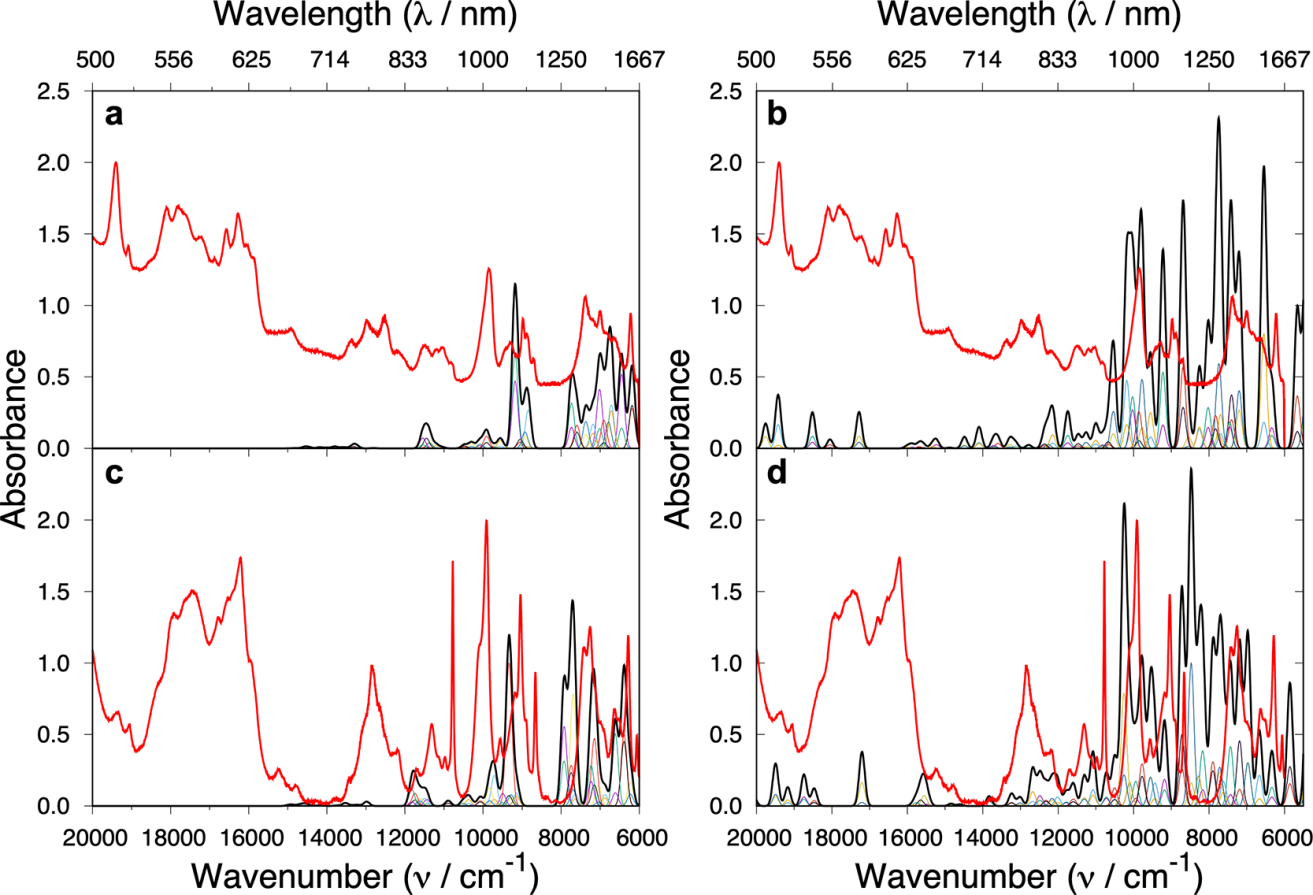
Experimental (red) and computed MS-CASPT2-SO (black) data for **2Pu** and **3Pu**: (a) **2Pu**, (b) **2Pu** with inclusion of an additional 16 quartet states, (c) **3Pu**, and (d) **3Pu** with inclusion of an additional 16 quartet states. Individual transitions (rainbow, below black) and total absorption spectrum were Gaussian-broadened with a half-width of 150 cm^–1^.

In all cases, the complexes fall into the same IER > SO > CF regime. Comparing the two ligand sets across all complexes, the I^Me4^ ligands in **3M** provide a larger CF splitting than the DME ligands in **2M**. Addition of CASPT2 corrections generally reduces the effective IER in all cases, reducing the energy of excited states, while the effect on SOC is negligible (as this is determined using the atomic mean-field method), and the effect on CF splitting is varied (Table 3). From comparison to experimental UV/vis/NIR spectra, we find the MS-CASPT2-SO results are most accurate, Figures 6–8, and hence focus on discussion of these data rather than the CASSCF-SO and XMS-CASPT2-SO data (Figures S94–S99).

The experimental UV/vis/NIR spectrum of **2Pr** is dominated by absorptions in the 6000–7500 cm^–1^ range, consisting of one major peak with several shoulders, Figure 6a. The lowest-energy calculated transition A corresponds to ^3^H_4_ → ^3^F_2_ excitations, which are known to occur at lower energy (∼5000 cm^–1^),^11^ which is out of the range of the experiment. The second calculated peak B corresponds to the main peak observed in the experiment, which arises from ^3^H_4_ → ^3^F_3_ excitations. The fine structure is likely a result of CF splitting of the *J* = 3 state but could correspond to different conformers in solution or phonon sidebands. The higher energy shoulder C corresponds to transitions with mixed ^3^F_4_ and ^1^G_4_ final states, which become mixed because they are nearby in energy and have Δ*J* = 0 and hence are mixed in first-order by SOC. Although ^3^F_4_ is the major component (49–66% ^3^F_4_ vs 30–42% ^1^G_4_), the ^3^H_4_ → ^1^G_4_ transitions are formally spin forbidden (Δ*S* ≠ 0) and this explains the weaker intensity of this feature. The experimental data shows further features near 16 500 and 20 000 cm^–1^ (Figure S67) which likely correspond to the ^1^D and ^3^P terms, respectively.^12^ However, we have not attempted to compute these highly excited states as their inclusion in state-averaged CASSCF calculations would negatively affect the quality of the lower-lying excited states.

The UV/vis/NIR absorption spectra for **2Sm** and **3Sm** show four main features each between 6000 and 8500 cm^–1^, Figure 7 (peaks A–D), and a further lower-intensity peak between 9500 and 10 000 cm^–1^, Figure 7 (peak E). Similar to the spectrum of Sm(III) in LaF_3_,^69,70^ the low-lying states (^6^H_5/2_, ^6^H_7/2_, ^6^H_9/2_, ^6^H_11/2_, ^6^H_13/2_) are energetically well-separated, but ^6^F_1/2_, ^6^F_3/2_, ^6^H_15/2_, and ^6^F_5/2_ overlap. For **2Sm**, peak A corresponds to multiple low-intensity transitions corresponding to excitations to mixed final states composed of mostly ^6^H_15/2_ character with a smaller contribution of ^6^*F*_1/2_ (in some cases up to 30%). The final states corresponding to peak B are approximately a 50:50 mixture of the ^6^H_15/2_ and ^6^F_3/2_ terms. The remaining peaks are more straightforward and involve pure final states: ^6^H_5/2_ → ^6^F_5/2_ (C), ^6^H_5/2_ → ^6^F_7/2_ (D), and ^6^H_5/2_ → ^6^F_9/2_ (E). These transitions are known to lie at ∼7000, ∼8000, and ∼9500 cm^–1^ for Sm(III) in LaF_3_,^69,70^ whereas our MS-CASPT2-SO results place them at ∼7800, ∼8600, and ∼9700 cm^–1^. For **3Sm**, the final states for peak A have a slightly different composition: several have >92% ^6^H_15/2_ character, and some are ∼75% ^6^F_1/2_ character. The final states corresponding to peak B are also mixed, however in contrast to the relatively equal composition for **2Sm**, some of the transitions instead correspond to a state dominated by the ^6^H_15/2_ term (∼70–80%). More similarly to **2Sm**, peaks C–E for **3Sm** correspond to transitions with the same pure final states as discussed above: ^6^H_5/2_ → ^6^F_5/2_ (C), ^6^H_5/2_ → ^6^F_7/2_ (D), and ^6^H_5/2_ → ^6^F_9/2_ (E).

The UV/vis/NIR spectra for the 5f^5^ Pu(III) compounds **2Pu** and **3Pu** contain significantly more features than their 4f^5^ Sm(III) analogues, Figure 8; this results from the stronger SO mixing, larger CF splitting and smaller IER. Interestingly, it is trivial to observe the differences between the spectra of the two compounds here, which must owe to the difference in the CF, a feature that is usually difficult to observe in 4f complexes (cf. Figures 6 and 7). Despite the complexity of the spectra, we observe good agreement between our MS-CASPT2-SO calculated spectra and the experimental data at low energies, Figure 8a and 8c. The intensity patterns are in good agreement and suggest minimal error in the calculated energies. Our experimental UV/vis/NIR spectra do not probe transitions below ∼6000 cm^–1^; however *ab initio* calculations can assign this low-energy region for **2Pu** as transitions from the ground ^6^H_5/2_ multiplet to ^6^H_7/2_ (∼1500–2400 cm^–1^), ^6^H_9/2_ (∼3600–4500 cm^–1^), and to ^6^F_1/2_ (∼5200 cm^–1^). Because of larger CF splitting, these transitions are shifted to slightly higher energies in **3Pu**: ^6^H_7/2_ (∼1600–2700 cm^–1^), ^6^H_9/2_ (∼3900–4900 cm^–1^), and ^6^F_1/2_ (∼5600 cm^–1^). These lower-energy multiplets are reasonably pure (>80%), but compared to the lanthanides, the **2Pu** and **3Pu** transitions are more mixed overall (Table S22). Furthermore, the amount of mixing seems to be slightly larger for **2Pu** compared to **3Pu**, and although less prominent, this is also seen for **2Sm** compared to **3Sm**. With the large amount of mixing, broad assignments can be made to regions of the spectra. The cluster of transitions within ∼6000–8000 cm^–1^ correspond to excitations to mixed ^6^F_5/2_ and ^6^H_11/2_ terms, and the transitions within ∼9000–16 000 cm^–1^ correspond to excitations to states made up of a mixture of ^6^F_9/2_, ^6^H_13/2_, and ^6^F_7/2_ terms. Peaks with weaker intensity near 11 500–12 000 cm^–1^ represent transitions to ^6^F_11/2_ and ^6^H_15/2_ terms. To examine the higher-energy region of the spectra (>17 000 cm^–1^), we have performed calculations with an additional 16 quartet states, however, inclusion of these states deteriorates the quality of the lower-energy states, so these calculations are used to characterize the higher-energy states only, Figure 8b and 8d. The transitions in this region have final quartet states that are extremely mixed (Table S28) and produce weak absorption intensities in comparison to experiment. It is also possible that other transitions (e.g., f–d transitions) contribute to the absorption intensity in this part of the spectra.

## Conclusions

We have established the synthesis and characterization of non-actinyl transneptunium multiple bonds, and plutonium NHC complexes, thus adding plutonium diphosphonioalkylidene and NHC linkages to the limited number of structurally authenticated organoplutonium complexes reported to date. Supporting the presence of Pu=C double bonds in the plutonium-diphosphonioalkylidene complexes, complex **2Pu** engages in metallo-Wittig bond metathesis involving the highest atomic number element to date, reacting with benzaldehyde to produce the corresponding alkene **4** along with “PuOI”. In contrast, the corresponding reactions with **2Ce** and **2Pr** do not produce the alkene **4**, possibly suggesting covalency differences in trivalent actinide– and lanthanide–carbon double bonds that lead to experimentally observable different outcomes in reactivity where more covalency in actinide–carbon double bonds leads to well-controlled metathesis but more ionic lanthanide analogs engage in more aggressive C–H activation reactions.

These Pu=C double and Pu–C dative bonds, along with new (praseodymium and samarium) and previously reported (uranium, neptunium, and cerium) diphosphonioalkyldiene congeners, have provided an opportunity to make lanthanide–actinide and actinide–actinide comparisons between metals with similar ionic radii and isoelectronic 4f^5^ vs 5f^5^ electron-counts within conserved ligand fields over 12 complexes. The bonding picture that emerges is clearly more complex than the traditional picture of the bonding of lanthanides being ionic and early actinides having relatively more covalent character and then becoming more ionic from left to right. Specifically, traversing the actinide series showed lower bond orders and ellipticities for these M=C bonds. Accompanying these changes were greater f- and lower d-orbital character with larger metal percentage contributions to the M=C bonds. Natural bond orbital and electron density topology analyses suggest that the orbital-energy matching (orbital mixing) and spatial-overlap terms increase from uranium to neptunium. In contrast, moving to plutonium improves the orbital-energy matching term and decreases the spatial-overlap. Overall, neptunium is the most covalent actinide in the series; however, the covalency of the plutonium complexes is still significant. Comparisons to the lanthanide analogs reveal a similarly intricate interplay of orbital-energy and spatial-overlap terms that is more complex than the traditional picture of lanthanide and actinide bonding.

Multiconfigurational calculations on **2M** and **3M** (M = Pu, Sm) account for the considerably more complex experimentally measured UV/vis/NIR spectra for **2Pu** and **3Pu** compared to **2Sm** and **3Sm**. The SO and CF splittings for the Pu(III) complexes are approximately double the values for the Sm(III) complexes, owing to more substantial relativistic effects and larger radial extension of the 5f- vs 4f-orbitals, respectively. However, the IER is approximately 50% larger for Sm(III) than Pu(III), likely arising from the more spatially confined nature of 4f-electrons compared to their 5f counterparts.

The recent reports of isolable Np=O,^26^ Np=C,^27^ and now Pu=C linkages, with their associated synthetic methodologies coupled to modern analytical techniques, pave the way for a wider expansion of transuranium metal–ligand multiple bond chemistry. Such advances will complement the major advances of uranium and thorium chemistry in recent years and have important ramifications for our understanding of the coordination and organometallic chemistry of the actinide elements.

## Data Availability

All other data are provided in the Supporting Information or are available from the authors on reasonable request.

## Supporting Information Available

Experimental and computational details and X-ray crystallographic, spectroscopic, magnetic, and quantum chemical calculations (PDF)
