# Supplementary material for: Carbene Complexes of Plutonium: Structure, Bonding, and Divergent Reactivity to Lanthanide Analogs
Source: J Am Chem Soc. 2024 Feb 1;146(6):4098–111. doi: 10.1021/jacs.3c12719 (PMC10870714; doi:10.1021/jacs.3c12719)
Supplement: Supplementary file 1 — ja3c12719_si_001.pdf [file ja3c12719_si_001.pdf]

– Supporting Information –

Carbene Complexes of Plutonium: Structure, Bonding, and Divergent Reactivity to Lanthanide Analogs

Jesse Murillo,[1] John A. Seed,[2] Ashley J. Wooles,[2] Meagan S. Oakley,[2] Conrad A. P. Goodwin,[1,2] Matthew Gregson,[2] David Dan,[1] Nicholas F. Chilton,[2,3*] Andrew J. Gaunt,[1*] Stosh A. Kozimor,[1] Stephen T. Liddle,[2*] Brian L. Scott[3]

[1] Chemistry Division, Los Alamos National Laboratory, Los Alamos, New Mexico 87545, USA.

[2] Department of Chemistry and Centre for Radiochemistry Research, The University of Manchester, Oxford Road, Manchester, M13 9PL, UK.

[3] Research School of Chemistry, The Australian National University, Sullivans Creek Road, Canberra, ACT, 2601, Australia.

[4] Materials Physics & Applications Division, Los Alamos National Laboratory, Los Alamos, New Mexico 87545, USA.

*Correspondence email: nicholas.chilton@manchester.ac.uk, gaunt@lanl.gov, steve.liddle@manchester.ac.uk

Experimental Details

For the Synthesis and Characterization of all Plutonium Compounds

Caution! ^{239}Pu decays principally through α-emission (Q_α = 5.244 MeV) ($t_{1/2}$ = 24,110(30) years) with β/γ-emission hazards also posed by daughter products and other isotopes present in nominally weapons-grade plutonium. Hence, all studies that involved manipulation of ^{239}Pu material were conducted in a specialized transuranium radiological designated area equipped with high efficiency particulate in air (HEPA) filtered hoods and in negative pressure gloveboxes. Safety controls included continuous air monitoring for airborne α-emitting particles and use of hand-held radiation monitoring equipment. Entrance to the laboratory space was controlled with a hand and foot radiation monitoring instrument and a full body personal contamination monitoring station. The handling of free-flowing solids was restricted to be within negative pressure gloveboxes equipped with HEPA filters. In addition to standard laboratory PPE, aqueous solutions were handled using multiple layers of gloves (of a material compatible with the chemicals being handled) combined with DuPont™ Tyvek® 400 sleeves to provide overlapping coverage of the arms.

All transuranium complexes were synthesized at Los Alamos National Laboratories and were carried out in a negative pressure M. Braun LabMaster glovebox configured for safe containment of transuranium isotopes including HEPA-filter outlets and the antechamber plumbed directly into a HEPA-filtered fumehood. The glovebox atmosphere was maintained with a standalone Vacuum Atmosphere Genesis™ oxygen and moisture removal system, and the suitability of the atmosphere was verified using a dilute toluene solution of $[Ti(Cp)_2(\mu\text{-}Cl)]_2$. Anhydrous solvents were purchased and stored for several weeks over activated 4 Å molecular sieves before use.

^{1}H and ^{31}P NMR data were recorded at 298 K on a Bruker AVANCE II 400 MHz spectrometer operating at 400.13 and 161.94 MHz, respectively; chemical shifts are quoted in ppm and are relative to TMS (^{1}H) and 85% H_3PO_4 (^{31}P). Likely due to the nature of NMR containment protocols, NMR

spectra always contain free-ligand even when dissolving pristine crystalline samples. UV/Vis/NIR spectra were recorded on a Varian Cary 6000i spectrophotometer; measurements on toluene solutions were made in a 1 cm path length screw-capped quartz cuvette. Where ε values are reported for molecular complexes, there is an assumed modest error due to the small quantities of weighed material, as is typical for similar chemistry performed on similar scales and working conditions. For solid-state UV/Vis/NIR measurements, single crystals were placed on a quartz slide under oil and spectra obtained at room temperature using a CRAIC Technologies microspectrophotometer; for absorbance measurements, data was collected from 28,557 to 5,886 cm^{-1} (350 to 1,700 nm). IR spectra were recorded on a Nicolet 6700 FT-IR spectrometer as a Nujol mull between two KBr plates, with electrical tape wrapped around the interface of the two plates to protect the sample from air and ensure radiological containment during transport. Extreme care had to be taken when loading the sample inside the glovebox to ensure the exterior surfaces of the salt plates did not become contaminated. Prepared samples were checked for surface contamination via direct and swipe counting methods before loading into the instrument for measurement. Single crystal X-ray studies were carried out at Los Alamos National Laboratories, in radiologically controlled conditions, on a Bruker AXS SMART APEX II diffractometer with a sealed Mo Kα X-ray source (α = 0.71073 Å). Single crystals were coated in NVH oil and mounted inside a 0.5 mm capillary tube which was then sealed under He atmosphere with capillary wax. The capillary was then coated with a thin film of Hard as Nails® (polyurethane) to provide structural integrity and an additional containment layer. Data collection and cell parameter determinations were conducted using the SMART program.[1] Integration of the data and final cell parameter refinements were performed using SAINT software[2] with data absorption correction implemented through SADABS.[3] The structures were solved either by direct or heavy methods and all non-hydrogen atoms were refined by full-matrix least-squares on all unique F^2 values with anisotropic displacement parameters with exceptions noted in the respective CIF files. Except where noted, hydrogen atoms were refined with constrained geometries and riding

thermal parameters. Structure solutions were completed in SHELXTL[4] or Olex2[5] crystallographic packages. ORTEP-3[6] and POV-Ray[7] were employed for molecular graphics.

For the Synthesis and Characterization of all Non-Plutonium Compounds

For all non-transuranium compounds, manipulations were carried out using Schlenk techniques, or an MBraun UniLab glovebox, under an atmosphere of dry nitrogen or argon. Solvents were dried by passage through activated alumina towers and degassed before use. All solvents were stored over potassium mirrors except for ethers, which were stored over activated 3 Å sieves. Deuterated solvents were dried over NaK_2, distilled, degassed by three freeze-pump-thaw cycles and stored under dinitrogen. Glassware was silylated with HMDS under reduced pressure.

Whilst obtainable, some spectra are not reported due to their paramagnetic nature. ^{1}H, ^{29}Si{^{1}H}, and ^{31}P{^{1}H} spectra were recorded variously on Bruker 400/JEOL JNM-ECZ 400 spectrometers operating at 400.1/399.78, 79.5/79.42, and 162.0/161.83 MHz, respectively; chemical shifts are quoted in ppm and are relative to TMS (^{1}H, ^{29}Si) and external 85% H_3PO_4 (^{31}P). Samples were prepared in the glovebox and placed in J. Young PTFE 5mm screw-topped borosilicate NMR tubes. ATR-IR spectra were recorded on a Bruker Alpha spectrometer with a Platinum-ATR module in the glovebox. UV/Vis/NIR spectra were recorded on a Perkin Elmer Lambda 750 spectrometer where data were collected in 10 mm path length cuvettes and run versus the appropriate reference solvent. Elemental microanalyses were carried out by Mr Martin Jennings and Mrs Anne Davies at The University of Manchester School of Chemistry and Mr Stephen Boyer at the Microanalysis Service, London Metropolitan University. Some CHN data, in particular C%, were persistently low, attributed to acknowledged halide impurities on small scale or incomplete combustion.[8] Crystals were examined using either a) an Agilent Supernova diffractometer, equipped with either an Atlas/AtlasS2 or TitanS2 CCD area detector and mirror-monochromated Cu Kα radiation (λ = 1.5418 Å) or b) a Rigaku XtalLAB Synergy-S diffractometer, equipped with a HyPix 6000HE photon counting pixel array

detector with mirror-monochromated Mo Kα (λ = 0.71073) or Cu K α (λ = 1.5418 Å) radiation. Intensities were integrated from a sphere of data recorded on narrow (0.5° or 1.0°) frames by *ω* rotation. Cell parameters were refined from the observed positions of all strong reflections in each data set. Gaussian grid face-indexed absorption corrections with a beam profile correction were applied. The structures were solved either by direct, heavy or dual methods and all non-hydrogen atoms were refined by full-matrix least-squares on all unique F^2 values with anisotropic displacement parameters with exceptions noted in the respective CIF files. Except where noted, hydrogen atoms were refined with constrained geometries and riding thermal parameters. CrysAlisPro[9] was used for control and integration, SHELXT[10] was used for structure solution, and SHELXL[11] and Olex2[5] were employed for structure refinement. ORTEP-3[6] and POV-Ray[7] were employed for molecular graphics.

Materials

The compounds [$MI_3(THF)_4$] (M = Pu or Pr, n = 4; M = Sm, n = 3.5),[12,13] [Rb(BIPM^{TMS}H)],[14] 1,3,4,5-tetramethylimidazol-2-ylidene (I^{Me4}),[15] and benzyl potassium (KBn)[16] were prepared according to published procedures.

Initial attempted synthesis of [Pu(BIPM^{TMS})(I)(DME)] (2Pu) and isolation of [Pu(BIPM^{TMS}H)(I)(μ-I)]$_2$·2Benzene (1Pu·2Benzene)

A 20 mL glass vial was charged with a PTFE-coated stirrer bar and solid [$PuI_3(THF)_4$] (0.034 g, 0.037 mmol). At room temperature, THF (~ 2 mL) was added with stirring resulting in the formation of a turbid orange suspension. Solid [Rb(BIPM^{TMS}H)] (0.024 g, 0.037 mmol) was added in two portions, which caused the mixture to slowly turn a pale teal color from the initial pale-yellow color, along with concomitant precipitation of fine white solids. After stirring for five minutes, all the solvent was removed *in vacuo* to afford a teal powder. To this, DME (1.5 mL) was added with stirring resulting in the formation of a teal suspension. Solid KBn (0.0049 g, 0.037 mmol) was added in several portions which caused the mixture to immediately turn from teal to muddy yellow/brown – the vivid orange

color of the KBn discharged instantaneously as each portion dissolved. After the cloudy mixture was stirred for a further five minutes, all solvent was removed *in vacuo* to afford a green/brown powder. To this, toluene (1.5 mL) and DME (3 drops) were added at room temperature, and the resultant suspension was stirred for two minutes. The suspension was filtered through two glass microfiber filter discs packed in a glass pipette into a 4 mL glass vial. The resultant solution was concentrated *in vacuo* to approximately 0.6 mL before being stored at room temperature for 16 hours causing a small amount of pale material to form. The solution was filtered again through two glass microfiber filter discs packed in a glass pipette into a 4 mL glass vial. Storage of this solution at –35 °C for 16 hours yielded a further crop of pale solid. The solution was filtered again through two glass microfiber filter discs packed in a glass pipette into a 4 mL glass vial. Concentration of this solution to approximately 0.2 mL and storage at –35 °C for 16 hours resulted in no further precipitation of solid. Subsequently, all solvent was removed in vacuo and the resultant solid extracted with benzene (1 mL) and filtered through two glass microfiber filter discs packed in a glass pipette into a 4 mL glass vial. Concentration of this solution to approximately 0.2 mL and storage at room temperature for 16 hours yielded teal plates of **1Pu·2Benz**. Yield: 0.0045 g, 11%. ^{1}H NMR (D_8-THF, 298 K): δ −6.95 (br s, 2H, BIPM^{TMS}H *ipso*-PC*H*P), −0.88 (br s, 36H, BIPM^{TMS}H Si(C*H*$_3$)$_3$), 6.99 (br s, 24H, BIPM^{TMS}H m-ArC*H* and p-ArC*H*), 7.88 (br s, 16H, BIPM^{TMS}H o-ArC*H*) ppm. ^{31}P{^{1}H} NMR (C_6D_6, 298 K): δ −83.36 (PuC*P*$_2$) ppm. Magnetic moment (Evans method, C_6D_6, 298 K): μ_{eff} = 3.65 μ_B. UV/Vis/NIR (toluene) λ_{max} nm (ν/cm^{-1}; ε/M^{-1} cm^{-1}): 313 (31,908, 3,394), 362 (27,609, 2,896), 397 (25,202, 1,452), 430 (23,245, 324), 443 (22,553, 178), 462 (21,636, 98), 517 (19,342, 64), 523 (19,128, 64), 565 (17,712, 104), 572 (17,483, 102), 591 (16,926, 76), 605 (16,534, 98), 609 (16,410, 118), 678 (14,741, 46), 800 (12,497, 40), 845 (11,829, 24), 889 (11,249, 38), 911 (10,979, 38), 932 (10,732, 32), 1,014 (9,864, 44), 1,051 (9,513, 32), 1,086 (9,205, 38), 1,111 (8,999, 36), 1,158 (8,634, 18), 1,385 (7,222, 76), 1,447 (6,910, 52), 1,528 (6,545, 38).

Preparation of [Pu(BIPM^{TMS})(I)(DME)]·0.5Toluene (2Pu·0.5Tol)

A 20 mL glass vial was charged with a PTFE-coated stirrer bar and solid [$PuI_3(THF)_4$] (0.035 g, 0.04 mmol). At room temperature, THF (~ 1.5 mL) was added resulting in the formation of a suspension. Solid [Rb(BIPM^{TMS}H)] (0.0255 g, 0.04 mmol) was added in three portions with stirring over two minutes resulting in an immediate color change from pale tan to colorless, followed by the rapid precipitation of a fine white solid after approximately three minutes. After stirring for 15 minutes, all the solvent was removed *in vacuo* to afford a pale tan powder. To this, DME (~ 1.5 mL) was added resulting in the formation of a tan suspension. Solid KBn (0.0052 g, 0.04 mmol) was added all at once with stirring resulting in the immediate formation of a pale green/brown suspension. After stirring for 15 minutes, all the solvent was removed *in vacuo* to afford a light brown powder. To this, toluene (~ 4 mL) and DME (10 drops) were added at room temperature, and the resultant green suspension stirred for five minutes. The suspension was filtered through a glass microfibre filter disc packed in a glass pipette into a 4 mL glass vial to obtain a clear, emerald-green solution. Concentration of this solution to approximately 0.5 mL and storage at –35°C for 16 hours yielded green rod-shaped crystals of **2Pu·0.5Tol**. Yield: 0.0247 g, 60%. ^{1}H NMR (C_6D_6, 298 K): δ –0.60 (s, 18H, BIPM^{TMS} Si(C*H*$_3$)$_3$), 3.08 (s, 6H, DME C*H*$_3$OCH$_2$), 3.50 (s, 4H, DME C*H*$_2$OCH$_3$), 7.06 (d, *J* = 6.2 Hz, 4H, BIPM^{TMS} p-ArC*H*), 7.12 (t, *J* = 7.0 Hz, 8H, BIPM^{TMS} m-ArC*H*), 7.72 (br s, 8H, BIPM^{TMS} o-ArC*H*) ppm. ^{31}P{^{1}H} NMR (C_6D_6, 298 K): δ –128.38 (PuC*P*$_2$) ppm. FT-IR ν/cm^{-1} (Nujol): 2953 (s), 2924 br (s), 2853 (s), 1496 (w), 1462 (m), 1456 (m), 1436 (m), 1377 (m), 1304 (w), 1282 (w), 1279 (w), 1259 (w), 1241 (w), 1190 (w), 1178 (w), 1190 (w), 1155 (w), 1112 (m), 1059 br (w), 853 (w), 828 br (m), 769 (w), 743 (w), 728 (w), 692 (w), 677 (w), 651 (w), 637 (w), 617 (w), 608 (w), 597 (w), 505 br (w), 487 (w), 476 (w), 465 (w), 465 (w), 445 (w), 415 (m). Magnetic moment (Evans method, C_6D_6, 298 K): μ_{eff} = 1.35 μ_B. UV/Vis/NIR (toluene) λ_{max} nm (ν/cm^{-1}; ε/M^{-1} cm^{-1}): 286 (34,892; 9,144), 515 (19,402; 65), 524 (19,077; 41), 553 (18,096; 52), 561 (17,819; 53), 568 (17,599; 50), 581 (17,218; 44), 593 (16,869; 38), 604 (16,567; 46), 614 (16,281; 51), 623 (16,062; 42), 630 (15,878; 38), 670 (14,921; 18), 747 (13,383; 15), 770 (12,984; 21), 799 (12,519; 21), 609

(12,186; 12), 678 (11,494; 13), 800 (11,201; 12), 845 (11,040; 13), 889 (10,827; 9), 1,014 (9,858; 35), 1,062 (9,418; 12), 1,150 (8,694; 21), 1,126 (8,881; 17), 1,150 (8,694; 9), 1,355 (7,382; 27), 1,427 (7,008; 23), 1,467 (6,818; 17), 1,505 (6,644; 15), 1,607 (6,221; 22).

Preparation of [Pu(BIPM^{TMS})(I)(I^{Me4})$_2$]·0.5Toluene (3Pu·0.5Tol)

A 20 mL glass vial was charged with a PTFE-coated stirrer bar and solid [$PuI_3(THF)_4$] (0.0362 g, 0.04 mmol). At room temperature, THF (~ 2.5 mL) was added resulting in the formation of a suspension. Solid [Rb(BIPM^{TMS}H)] (0.0258 g, 0.04 mmol) was added in three portions with stirring over two minutes resulting in an immediate color change from pale tan to colorless, followed by the rapid precipitation of a fine white solid after approximately three minutes. After stirring for 15 minutes, all the solvent was removed *in vacuo* to afford a pale tan powder. To this, DME (~ 1.5 mL) was added resulting in the formation of a tan suspension. Solid KBn (0.0052 g, 0.04 mmol) was added all at once with stirring resulting in the immediate formation of a pale green/brown suspension. After stirring for 15 minutes, all the solvent was removed *in vacuo* to afford a light brown powder. To this, toluene (~ 3.5 mL) and DME (25 drops) were added at room temperature, and the resultant green suspension stirred for five minutes. The suspension was filtered through a glass microfibre filter disc packed in a glass pipette to obtain a clear, emerald-green solution. A PTFE-coated stirrer bar was added to this solution, and solid I^{Me4} (0.009 g, 0.07 mmol) added in portions with stirring resulting in a color change from green to yellow. The solution was stirred for five minutes before being concentrated *in vacuo* to approximately 1 mL resulting in the precipitation of a yellow microcrystalline solid. To this, was added THF (~ 0.5 mL) and the reaction mixture heated to 100°C for five minutes. During heating, all solids dissolved, and the resultant clear yellow solution was allowed to cool to room temperature over five hours to yield green/yellow block crystals of **3Pu·0.5Tol**. Yield: 0.0132 g, 28%. ^{1}H NMR (D_8-THF, 298 K): δ –1.27 (s, 18H, BIPM^{TMS} Si(C$\underline{H}_3$)$_3$), 1.61 (s, 12H, I^{Me4} C(C$\underline{H}_3$), 4.21 (s, 12H, I^{Me4} N(C$\underline{H}_3$) ppm. The resonances attributed to the 20 protons of BIPM^{TMS} {Ar-(C$\underline{H}$)} could not be definitively assigned due to their broad nature and overlapping

with peaks across the range δ +7.77 to 6.90 ppm. $^{31}P\{^1H\}$ NMR (D_8-THF, 298 K): δ –141.88 (PuC$\underline{P}_2$) ppm. FT-IR ν/cm^{-1} (Nujol): 2953 (s), 2923 br (s), 2853 (s), 1460 (m), 1377 (m), 1366 (m), 1301 (w), 1238 (w), 1111 (w), 1103 (w), 862 (w), 828 (w), 727(w), 693 (w), 1112 (m), 1059 br (w), 853 (w), 828 br (m), 769 (w), 743 (w), 728 (w), 692 (w), 600 (w), 548 (w), 429 (m), 419 (w). Magnetic moment (Evans method, D_8-THF, 298 K): μ_{eff} = 0.90 μ_B. UV/Vis/NIR (THF) λ_{max} nm (ν/cm^{-1}; ε/M^{-1} cm^{-1}): 286 (34,941, 8,711), 399 (25,050, 1,174), 516 (19,395, 19), 525 (19,055, 17), 546 (18,328, 24), 558 (17,934, 38), 567 (17,637, 40), 574 (17,434, 42), 596 (16,790, 37), 605 (16,540, 40), 617 (16,213, 48), 626 (15,969, 29), 657 (15,225, 8), 676 (14,802, 5), 778 (12,850, 28), 819 (12,207, 11), 854 (11,704, 8), 884 (11,317, 17), 911 (10,977, 10), 928 (10,776, 48), 1,009 (9,915, 55), 1,046 (9,558, 14), 1,090 (9,173, 23), 1,106 (9,040, 41), 1,123 (8,905, 17), 1,154 (8,664, 26), 1,349 (7,413, 31), 1,377 (7,263, 35), 1,427 (7,007, 17), 1,507 (6,635, 20), 1,537 (6,505, 17), 1,591 (6,286, 33), 1,650 (6,059, 14).

Reaction of [Pu(BIPM^{TMS})(I)(DME)] (2Pu) with PhCHO to give PhC(H)=C(PPh$_2$NSiMe$_3$)$_2$ (4)

A glass vial was charged with a PTFE-coated stirrer bar and solid [(BIPM^{TMS})Pu(I)(DME)] (0.0072 g, 0.007 mmol). At room temperature, C_6D_6 (~ 1.5 mL) was added with stirring resulting in the formation of a clear light brown solution. At room temperature, a solution of PhCHO (0.0008 g, 0.0075 mmol) in C_6D_6 (~ 1.5 mL) was added dropwise with stirring, and the mixture left to stir for 96 hours resulting in a color change from light brown to light yellow. After which, the mixture was filtered through a glass microfibre filter disc packed in a glass pipette to obtain a clear yellow solution, which was concentrated *in vacuo* to approximately 1.5 mL and used for subsequent NMR spectroscopic analysis. ^1H NMR (C_6D_6, 298 K): δ 0.37 (s, 9H, NSi(C$\underline{H}_3$)$_3$), 0.39 (s, 9H, NSi(C$\underline{H}_3$)$_3$), 6.78 (br, 2H, *m*-ArC$\underline{H}$), 6.89 (br, 6H, *p*-ArC$\underline{H}$ and *o*-ArC$\underline{H}$), 7.02 (br, 8H, *m*-ArC$\underline{H}$), 7.39 (br, 1H, *p*-ArC$\underline{H}$), 7.78 (m, 8H, *o*-ArC$\underline{H}$), 8.04 (dd, $^3J_{PH}$ = 28.4 and 28.3 Hz, 1 H, Ar($\underline{H}$)C=CP$_2$) ppm. ^{31}P NMR (C_6D_6, 298 K): δ 6.75 (d, $^2J_{PP}$ = 35.2 Hz), –7.79 (d, $^2J_{PP}$ = 35.2 Hz) ppm.

Preparation of [Pr(BIPM^{TMS}H)(I)$_2$(THF)]·0.5Toluene (1Pr.THF·0.5Tol)

A Schlenk flask was charged with a PTFE-coated stirrer bar and a solid mixture of [$PrI_3(THF)_4$] (4.64 g, 6.00 mmol) and [Rb(BIPM^{TMS}H)] (3.86 g, 6.00 mmol). At –78°C, THF (20 mL) was added, and the reaction mixture allowed to warm slowly to room temperature resulting in the formation of a yellow suspension, which was stirred for 16 hours. The suspension was then filtered, volatiles removed *in vacuo*, and the off-white solid dried for two hours. This solid was then washed with pentane (2 x 25 mL) to afford **1Pr.THF·0.5Tol** as a colorless powder. Yield: 5.72 g, 93%. Single crystals suitable for X-ray diffraction were grown from a hot toluene solution which was allowed to cool slowly to room temperature. Anal. Calcd for $C_{35}H_{47}I_2N_2OP_2PrSi_2$: C, 41.06; H, 4.63; N, 2.74 %. Found: C, 41.09; H, 4.53; N, 2.42. ^{1}H NMR (D$_8$-THF, 298 K): δ –48.82 (s, 1H, BIPM^{TMS}H ipso-PC$\underline{H}$P), 1.74 (s, 4H, DME OC$\underline{H}_2$CH$_2$), 3.61 (s, 4H, DME OC$\underline{H}_2$CH$_2$), 11.12 (s, 18H, BIPM^{TMS}H Si(C$\underline{H}_3$)$_3$) ppm. The resonances attributed to the 20 protons of BIPM^{TMS}H{Ar-(C$\underline{H}$)} could not be definitively assigned due to their broad nature and overlapping with peaks across the range δ +7.75 to 4.75 ppm. ^{31}P{^{1}H} NMR (D$_8$-THF, 298 K): δ –110.65 (PrC$\underline{P}_2$) ppm. ^{29}Si{^{1}H} NMR (D$_8$-THF, 298 K): δ –1.96 ppm. FTIR v/cm^{-1}: 3052 (w), 2952 (w), 2890 (w), 1435 (m), 1248 (m), 1210 (w), 1185 (w), 1114 (s), 1094 (m), 1047 (m), 1025 (m), 1002 (w), 986 (m), 835 (s), 751 (s), 715 (w), 693 (s), 656 (m) 606 (s), 560 (m), 524 (s), 479 (m), 437 (m). Magnetic moment (Evans method, C_6D_6, 298 K): μ_{eff} = 4.08 μ_B. UV/Vis/NIR λ_{max} nm (ν/cm^{-1}; ε/M^{-1} cm^{-1}): 452 (22,104; 4), 474 (21,106; 4), 480 (20,842; 3), 488 (20,492; 4), 492 (20,309; 3), 580 (17,229; 1), 599 (16,708; 1), 1442 (6,936; 1), 1538 (6,504; 4), 1581 (6,326; 3).

Preparation of [Pr(BIPM^{TMS}H)(I)$_2$(I^{Me4})] (1Pr.IMe4)

A 20 mL glass vial was charged with a PTFE-coated stirrer bar and solid [Pr(BIPM^{TMS}H)(I)$_2$(THF)] (0.052 g, 0.05 mmol). At room temperature, toluene (~ 4 mL) was added with stirring resulting in the formation of a colorless suspension. Solid I^{Me4} (0.006 g, 0.049 mmol) was added all at once with stirring resulting in the immediate formation of a clear yellow solution. This solution was stirred for

five minutes during which time the yellow color faded slightly. After which, the solution was allowed to stand for 24 hours at room temperature leading to the deposition of pale-yellow crystals of **1Pr.IMe4**. Yield: 0.032 g, 59%. Anal. Calcd for $C_{38}H_{51}PrI_2N_4P_2Si_2$: C, 42.42; H, 4.78; N, 5.21%. Found: C, 41.59; H, 4.88; N, 5.48%. The ^{1}H NMR (D_8-THF, 298 K) spectrum displays several unassignable broad resonances across the range δ +20 to –20 ppm. ^{31}P{^{1}H} NMR (D_8-THF, 298 K): δ –169.07 ppm. FTIR *ν*/cm^{-1}: 3050 (w), 2950 (w), 2900 (w), 1435 (m), 1246 (m), 1151 (s), 1111 (s), 1065 (s), 933 (m), 836 (s), 741 (w), 708 (w), 694 (m), 659 (m), 612 (m), 588 (s), 551 (s), 511 (s), 466 (m). The gradual precipitation of [Pr(BIPM^{TMS}H)(I)$_2$(I^{Me4})] from deuterated ethereal and aromatic solvents precluded the acquisition of the Evans method magnetic moment. UV/Vis/NIR (THF) λ_{max} nm (ν/cm^{-1}; ε/M^{-1} cm^{-1}): 452 (22,134; 7), 459 (21,768; 7), 468 (21,377; 7), 482 (20,764; 6), 493 (20,292; 5), 499 (20,024; 5), 505 (19,818; 5), 591 (16,920; 3), 605 (16,529; 3), 1433 (6,977; 2), 1523 (6,564; 3), 1573 (6,359; 3), 1627 (6,145; 2).

Preparation of [Pr(BIPM^{TMS})(I)(DME)] (2Pr)

A 20 mL glass vial was charged with a PTFE-coated stirrer bar and solid [Pr(BIPM^{TMS}H)(I)$_2$(THF)] (0.052 g, 0.05 mmol). At room temperature, DME (~ 2 mL) was added with stirring resulting in the formation of a clear colorless solution. Solid KBn (0.0065 g, 0.05 mmol) was added all at once with stirring resulting in the immediate formation of a yellow suspension. This suspension was stirred for 15 minutes during which time the yellow color faded slightly. After stirring for 15 minutes, all the solvent was removed *in vacuo* and the resultant yellow solid dried for 1 hour. The solid was then extracted with toluene (2 x 2 mL) and filtered through a glass microfibre filter disc packed in a glass pipette to obtain a clear, golden yellow solution. Storage of this solution at –30°C for 24 hours yielded yellow needle crystals of **2Pr**. Yield: 0.038 g, 83%. Anal. Calcd for $C_{35}H_{48}IN_2O_2P_2PrSi_2$: C, 46.00; H, 5.29; N, 3.07%. Found: C, 45.80; H, 4.88; N, 2.85%. ^{1}H NMR (D_8-THF, 298 K): δ –0.69 (s, 18H, BIPM^{TMS} Si(C*H*$_3$)$_3$), 3.31 (s, 6H, DME C*H*$_3$OCH$_2$), 3.38 (s, 4H, DME C*H*$_2$OCH$_3$), 8.08 (t, *J* = 7.3 Hz, 4H, BIPM^{TMS} p-ArC*H*), 8.26 (t, *J* = 7.4 Hz, 8H, BIPM^{TMS} m-ArC*H*), 13.23 (s, 8H, BIPM^{TMS} o-

ArC*H*) ppm. $^{31}P\{^1H\}$ NMR (D_8-THF, 298 K): δ −125.47 (PrC*P*$_2$) ppm. $^{29}Si\{^1H\}$ NMR (D_8-THF, 298 K): δ –22.98 (BIPM^{TMS} *Si*$(CH_3)_3$) ppm. FTIR ν/cm^{-1}: 3051 (w), 2945 (w, br), 2886 (w, br), 1479 (w), 1434 (m), 1356 (m), 1303 (w), 1280 (w), 1243 (s), 1176 (w), 1105 (s), 1057 (s), 1024 (w), 915 (w), 826 (s), 746 (m), 725 (w), 708 (w), 693 (s), 653 (w), 638 (w), 595 (s), 542 (w), 508 (s), 436 (m), 419 (w). Magnetic moment (Evans method, C_6D_6, 298 K): μ_{eff} = 3.56 μ_B. UV/Vis/NIR (THF) λ_{max} nm (ν/cm^{-1}; ε/M^{-1} cm^{-1}): 452 (22,124; 10), 459 (21,777; 10), 467 (21,413; 10), 483 (20,704; 8), 493 (20,276; 7), 500 (20,016; 7), 505 (19,794; 6), 600 (16,644; 5), 606 (16,512; 5), 1430 (6,991; 2), 1529 (6,542; 4), 1574 (6,355; 4), 1624 (6,158; 2).

Preparation of [Pr(BIPM^{TMS})(I)(I^{Me4})$_2$]·0.5Toluene (3Pr·0.5Tol)

A 20 mL glass vial was charged with a PTFE-coated stirrer bar and solid [Pr(BIPM^{TMS}H)(I)$_2$(THF)] (0.052 g, 0.05 mmol). At room temperature, THF (~ 2 mL) was added with stirring resulting in the formation of a clear colorless solution. Solid KBn (0.0065 g, 0.05 mmol) was added all at once with stirring resulting in the immediate formation of a yellow suspension. This suspension was stirred for 15 minutes during which time the yellow color faded slightly. After stirring for 15 minutes, all the solvent was removed *in vacuo* and the resultant yellow solid dried for 1 hour. The solid was then extracted with toluene (2 x 2 mL) and filtered through a glass microfibre filter disc packed in a glass pipette to obtain a clear yellow solution. To this solution was added two equivalents of solid I^{Me4} (0.0123 g, 0.10 mmol) and the solution was briefly shaken until the precipitation of solid could be observed to be beginning (~ 30 seconds). At which point, the solution was allowed to stand at room temperature for 24 hours leading to the deposition of yellow crystals of **3Pr·0.5Tol**. Yield: 0.024 g, 45%. Anal. Calcd for $C_{45}H_{62}IN_6P_2PrSi_2$: C, 50.41; H, 5.83; N, 7.84%. Found: C, 45.72; H, 5.41; N, 6.35%. The final product is consistently contaminated with KI due to the rapid precipitation of both KI and **3Pr** from solution upon the addition of I^{Me4}. Compound **3Pr** is insoluble in aromatic and ethereal solvents precluding its characterization by NMR spectroscopy. ATR-IR ν/cm^{-1}: 3017 (w, br), 2976 (w, br), 1638 (w), 1577 (m), 1479 (w), 1441 (w), 1415 (w), 1305 (m), 1231 (s), 1207 (m),

1182 (m), 1122 (s), 980 (s), 828 (m), 744 (m), 716 (w), 698 (w), 622 (m), 524 (s). The precipitation of **3Pr** from deuterated ethereal and aromatic solvents precluded the acquisition of the Evans method magnetic moment.

Preparation of [Sm(BIPM^{TMS}H)(I)$_2$(THF)]·0.5THF (1Sm.THF·0.5THF)

A Schlenk flask was charged with a PTFE-coated stirrer bar and a solid mixture of [$SmI_3(THF)_{3.5}$] (2.35 g, 3 mmol) and [Rb(BIPM^{TMS}H)] (1.93 g, 3.00 mmol). At –78°C, THF (20 mL) was added, and the reaction mixture allowed to warm slowly to room temperature resulting in the formation of a yellow suspension, which was stirred for 72 hours. The suspension was then allowed to settle before filtering to afford a clear yellow solution. The solution was concentrated *in vacuo* to approximately 8 mL before being stored at –30°C for 24 hours to yield yellow crystals of **1Sm.THF·0.5THF**. Yield: 1.68 g, 54%. Anal. Calcd for $C_{35}H_{47}I_2N_2OP_2Si_2Sm$: C, 40.65; H, 4.58; N, 2.71%. Found: C, 40.42; H, 4.67; N, 2.40%. ^{1}H NMR (D_8-THF, 298 K): δ 0.49 (s, 18H, BIPM^{TMS}H Si(C$\underline{H}_3$)$_3$), 1.69 (s, 4H, DME $OCH_2C\underline{H}_2$), 3.11 (s, 1H, BIPM^{TMS}H ipso-PC$\underline{H}$P), 3.54 (s, 4H, DME OC$\underline{H}_2$CH$_2$), 7.01–7.81 (br, m, 20H, BIPM^{TMS}H ArC$\underline{H}$) ppm. ^{31}P{^{1}H} NMR (D_8-THF, 298 K): δ 39.57 (SmC$\underline{P}_2$) ppm. ^{29}Si{^{1}H} NMR (D_8-THF, 298 K): δ 3.01 (BIPM^{TMS} $\underline{Si}$(CH$_3$)$_3$) ppm. ATR-IR v/cm^{-1}: 3052 (w), 2949 (w), 2887 (w), 1482 (w), 1436 (m), 1307 (w), 1244 (s), 1183 (w), 1151 (w), 1114 (s), 1089 (w), 1056 (w), 1026 (w), 998 (w), 983 (w), 921 (w), 834 (s), 763 (w), 750 (w), 736 (w), 723 (w), 708 (w), 693 (m), 656 (w), 621 (m), 603 (m), 546 (s), 502 (m), 474 (w), 442 (w), 424 (w). Magnetic moment (Evans method, C_6D_6, 298 K): μeff = 1.59 μ_B. UV/Vis/NIR (THF) λ_{max} nm (ν/cm^{-1}; ε/M^{-1} cm^{-1}): 418 (23,946; 58), 1086 (9,208; 6), 1246 (8,028; 7), 1385 (7,223; 7), 1493 (6,697; 8), 1534 (6,518; 7), 1598 (6,257; 5).

Preparation of [Sm(BIPM^{TMS}H)(I)$_2$(I^{Me4})] (1Sm.IMe4)

A 20 mL glass vial was charged with a PTFE-coated stirrer bar and solid [Sm(BIPM^{TMS}H)(I)$_2$(THF)] (0.103 g, 0.10 mmol). At room temperature, toluene (~ 2 mL) was added, and the resulting suspension heated at 50 °C until all material had dissolved (approx. five minutes). To this clear colorless solution

was added solid I^{Me4} in portions (0.0113 g, 0.092 mmol) and the reaction mixture heated to 130 °C for five minutes. The resultant yellow solution was allowed to cool to room temperature before being stored at –30°C for 24 hours to yield yellow crystals of **1Sm.IMe4**. Yield 0.040 g, 37%. Anal. Calcd for $C_{38}H_{51}I_2N_4P_2Si_2Sm$: C, 42.02; H, 4.73; N, 5.16%. Found: C, 40.30; H, 4.77; N, 4.23%. The ^{1}H NMR (D_8-THF, 298 K) spectrum displays several unassignable broad resonances across the range δ +11 to –2 ppm. ^{31}P{^{1}H} NMR (D_8-THF, 298 K): δ –7.20 (SmC$\underline{P}_2$) ppm. ATR-IR *ν*/cm^{-1}: 2950 (w), 1436 (m), 1262 (m), 1133 (m), 1062 (s), 936 (m), 836 (s), 763 (w), 724 (w), 709 (m), 659 (m), 614 (m), 596 (m), 552 (s), 510 (m), 469 (m). The gradual precipitation of [Sm(BIPM^{TMS}H)$(I)_2$(I^{Me4})] from deuterated ethereal and aromatic solvents precluded the acquisition of the Evans method magnetic moment. UV/Vis/NIR (THF) λ_{max} nm (ν/cm^{-1}; ε/M^{-1} cm^{-1}): 414 (24,155; 57), 432 (23,148; 41), 1084 (9,225; 13), 1237 (8,084; 13), 1381 (7,241; 13), 1489 (6,716; 12), 1543 (6,481; 12), 1609 (6,215; 9).

Preparation of [Sm(BIPM^{TMS})(I)(DME)]·2.5Toluene (2Sm·2.5Tol)

A 20 mL glass vial was charged with a PTFE-coated stirrer bar and solid [Sm(BIPM^{TMS}H)$(I)_2$(THF)] (0.103 g, 0.10 mmol). At room temperature, DME (~ 2 mL) was added with stirring resulting in the formation of a clear colorless solution. Solid KBn (0.0137 g, 0.10 mmol) was added all at once with stirring resulting in the immediate formation of a yellow suspension. This suspension was stirred for 15 minutes during which time the yellow color faded slightly. After stirring for 15 minutes, all the solvent was removed *in vacuo* and the resultant yellow solid dried for 10 minutes. The solid was then extracted with toluene (2 x 2 mL) and filtered through a glass microfibre filter disc packed in a glass pipette to obtain a clear, golden yellow solution. Storage of this solution at –30°C for 24 hours yielded yellow needle crystals of **2Sm·2.5Tol**. Yield: 0.072 g, 78%. Anal. Calcd for $C_{35}H_{48}IN_2O_2P_2Si_2Sm$: C, 45.49; H, 5.23; N, 3.03%. Found: C, 45.37; H, 5.25; N, 3.31%. ^{1}H NMR (D_8-THF, 298 K): δ 0.23 (s, 18H, BIPM^{TMS} Si(C$\underline{H}_3$)$_3$), 3.27 (s, 6H, DME C$\underline{H}_3$OCH$_2$), 3.43 (s, 4H, DME C$\underline{H}_2$OCH$_3$), 7.16 (t, *J* = 7.3 Hz, 8H, BIPM^{TMS} m-ArC$\underline{H}$), 7.32 (m, 4H, BIPM^{TMS} p-ArC$\underline{H}$), 7.79 (d, *J* = 7.3 Hz, 8H, BIPM^{TMS}

o-ArC$\underline{H}$) ppm. $^{31}P\{^1H\}$ NMR (D_8-THF, 298 K): δ 59.24 (SmC$\underline{P}_2$) ppm. ATR-IR ν/cm^{-1}: 3053 (w), 2942 (m), 2883 (w), 1434 (m), 1358 (s), 1304 (w), 1284 (w), 1241 (m), 1179 (w), 1118 (w), 1101 (m), 1061 (m), 1041 (m), 999 (w), 828 (s), 746 (m), 725 (w), 708 (w), 694 (s), 653 (m), 638 (m), 618 (w), 597 (s), 523 (w), 508 (s), 479 (w), 448 (m), 414 (m). Magnetic moment (Evans method, C_6D_6, 298 K): μ_{eff} = 1.93 μ_B. UV/Vis/NIR (THF) λ_{max} nm (ν/cm^{-1}; ε/M^{-1} cm^{-1}): 602 (16,611; 7), 822 (12,159; 2), 1085 (9,217; 1), 1243 (8,048; 3), 1380 (7,248; 3), 1434 (6,975; 2), 1491 (6,707; 4), 1561 (6,405; 2), 1599 (6,255; 2).

Preparation of [Sm(BIPM^{TMS})(I)(I^{Me4})$_2$]·0.5Toluene (3Sm·0.5Tol)

A 20 mL glass vial was charged with a PTFE-coated stirrer bar and solid [Sm(BIPM^{TMS}H)(I)$_2$(THF)] (0.052 g, 0.05 mmol). At room temperature, DME (~ 2 mL) was added with stirring resulting in the formation of a clear colorless solution. Solid KBn (0.0065 g, 0.05 mmol) was added all at once with stirring resulting in the immediate formation of a yellow suspension. This suspension was stirred for 15 minutes during which time the yellow color faded slightly. After stirring for 15 minutes, all the solvent was removed *in vacuo* and the resultant yellow solid dried for 10 minutes. The solid was then extracted with toluene (2 x 2 mL) and filtered through a glass microfibre filter disc packed in a glass pipette to obtain a clear, golden yellow solution. To this solution was added two equivalents of solid I^{Me4} (0.0123 g, 0.10 mmol) and the solution was briefly shaken by hand until the precipitation of solid could be observed to be beginning (~ 30 seconds). At which point, the solution was allowed to stand at room temperature for 24 hours leading to the deposition of yellow crystals of **3Sm·0.5Tol**. Yield: 0.021 g, 39%. Anal. Calcd for $C_{45}H_{62}IN_6P_2Si_2Sm$: C, 49.93; H, 5.77; N, 7.76%. Found: C, 39.47; H, 4.75; N, 7.76%. The final product is consistently contaminated with KI due to the precipitation of both KI and **3Sm** from solution upon the addition of I^{Me4}. ^{1}H NMR (D_8-THF, 298 K): δ 0.95 (s, 18H, BIPM^{TMS} Si(C$\underline{H}_3$)$_3$), 1.46 (br), 2.30 (s, 12H) ppm. The resonances attributed to the 20 protons of BIPM^{TMS}\{Ar-(C$\underline{H}$)\} could not be definitively assigned due to their broad nature and overlapping with peaks across the range δ +7.75 to 7.05 ppm. The resonances attributed to \{C(C$\underline{H}_3$)\} and \{N(C$\underline{H}_3$)\} of

the I^{Me4} moiety could not be definitively assigned due to their broad nature and overlapping with peaks from contaminant toluene and D_8-THF solvent used for the NMR experiments. $^{31}P\{^1H\}$ NMR (D_8-THF, 298 K): δ 13.35 (SmC$\underline{P}_2$) ppm. ATR-IR *ν*/cm^{-1}: 3049 (w), 3018 (w), 2946 (w), 1638 (w), 1577 (m), 1477 (w), 1434 (m), 1397 (w), 1368 (m), 1280 (w), 1234 (s), 1210 (w), 1173 (w), 1155 (w), 1101 (m), 1086 (w), 1059 (s), 998 (w), 826 (s), 764 (w), 750 (w), 731 (m), 691 (s), 650 (m), 620 (w), 602 (m), 550 (s), 520 (w), 508 (m), 487 (m), 467 (w), 440 (m). The gradual precipitation of **3Sm** from deuterated ethereal and aromatic solvents precluded the acquisition of the Evans method magnetic moment. UV/Vis/NIR (THF) λ_{max} nm (ν/cm^{-1}; ε/M^{-1} cm^{-1}): 418 (23,923; 230), 734 (13,624; 80), 1069 (9,355; 46), 1221 (8,190; 43), 1364 (7,331; 37), 1476 (6,775; 34), 1501 (6,664; 29), 1607 (6,223; 27).

Representative attempted reactions of 2Ce and 2Pr with PhCHO

A glass vial was charged with a PTFE-coated stirrer bar and solid [M(BIPM^{TMS})(I)(DME)] (M = Ce, **2Ce** or M = Pr, **2Pr**) (0.015 or 0.012 g, 0.019 or 0.015 mmol). At room temperature, C_6D_6 (0.3 mL) was added with stirring. At room temperature, a solution of PhCHO (0.0019 or 0.0015 g, 0.018 or 0.014 mmol) in C_6D_6 (0.25 mL) was added dropwise with stirring. The mixture was allowed to stir for 96 hours. The respective mixtures were then assayed by NMR spectroscopy. Ce-reaction ^{31}P NMR (C_6D_6, 298 K): δ 27.73, 24.44, 21.38, 20.79 ppm. Pr-reaction $^{31}P\{^1H\}$ NMR (C_6D_6, 298 K): δ 36.96, 26.48, 21.27, 16.49 ppm. Since the purpose of the reaction was to ascertain whether metallo-Wittig chemistry occurs and it does not, and because clearly several species form, the ^1H NMR spectra were not examined.

Computational Details

Density Functional Theory and Ab Initio Calculations

Geometry optimizations for **2M** and **3M** (M = Pu, Pr, Sm) were performed using coordinates derived from their crystal structures as the starting points. No constraints were imposed on the structures

during the geometry optimizations. The calculations were performed using the Amsterdam Density Functional (ADF) suite version 2017 with standard convergence criteria, Tables S6-S11.[17,18] The DFT geometry optimizations employed Slater type orbital (STO) triple-ζ-plus polarization all-electron basis sets (from the Dirac and ZORA/TZP database of the ADF suite). Scalar relativistic approaches (spin-orbit neglected) were used within the ZORA Hamiltonian[19-21] for the inclusion of relativistic effects and the local density approximation (LDA) with the correlation potential due to Vosko *et al* was used in all of the calculations.[22] Generalized gradient approximation corrections were performed using the functionals of Becke and Perdew.[23,24] Analytical frequency calculations were carried out within the ADF program. Natural Bond Order (NBO) analyses were carried out with NBO 6.0.19.[25] The Quantum Theory of Atoms in Molecules analysis[26,27] was carried out within the ADF program. The ADF-GUI (ADFview) was used to prepare the three-dimensional plots of the electron density. In all cases, Aufbau formulations were found with the appropriate spin formulations ($5f^5$ Pu(III), sextet; $4f^2$ Pr(III), triplet, $4f^5$ Sm(III), sextet).

Complete-active space self-consistent field (CASSCF) theory[28,29] was used to compute the multiconfigurational wavefunctions of **2M** and **3M** (M = Pu, Pr, Sm) using the experimental XRD structures in *OpenMolcas* v22.06.[30] In the case of Pr(III), which has a ground configuration of $4f^2$, we choose a (2,7) active space, giving rise to $S = 1$ and $S = 0$ spin states, of which we select 9 singlets and 18 triplets, corresponding to the ^3H, ^3F and ^1G terms. The ground configuration for Sm(III) and Pu(III) is $4f^5$ and $5f^5$, respectively, which minimally requires a (5,7) active space to distribute the f electrons within the seven respective f-orbitals. This electron configuration gives rise to $S = 6$, $S = 4$, and $S = 2$ spin states, however, we focus here on the lowest lying 18 $S = 6$ roots corresponding to the lowest lying ^6H and ^6F terms; for Pu(III) we also explored including an additional 16 $S = 4$ roots to model the ^4G and ^4F terms. Scalar relativistic effects were evaluated with the second-order Douglas-Kroll-Hess (DKH2) Hamiltonian[31,32] and spin-orbit effects were included *a posteriori* via Restricted Active Space State Interaction (RASSI-SO).[33-35] ANO-RCC[36,37] basis sets were used for all atoms:

VTZP for An/Ln, VDZP for the first coordination sphere, and remaining atoms with VDZ. Dynamic correlation was included with multi-state (MS-CASPT2) and extended multi-state[37] (XMS-CASPT2) complete active space second order perturbation theory with an imaginary shift 0.2 a.u. to mitigate intruder states.[38-40] Crystal field parameters were projected at the CASSCF-SO, MS-CASPT2-SO, and XMS-CASPT2-SO levels of theory with the molcas_suite program.[41] Transition intensities were computed in the SO basis and were broadened with a half-width of 150-250 cm^{-1}.

Figures

Solid-State Structures

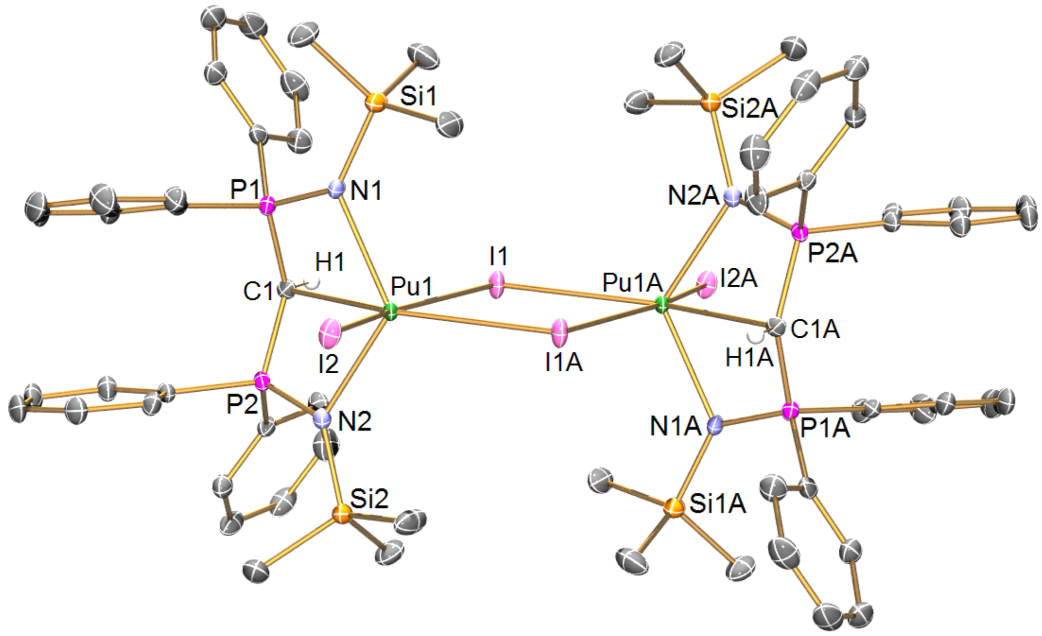

Figure S1. Molecular structure of $[Pu(BIPM^{TMS}H)(I)(\mu\text{-}I)]_2$ (**1Pu**) at 150 K with selected atom labels. Displacement ellipsoids are set at 40% and non-methanide hydrogen atoms are omitted for clarity.

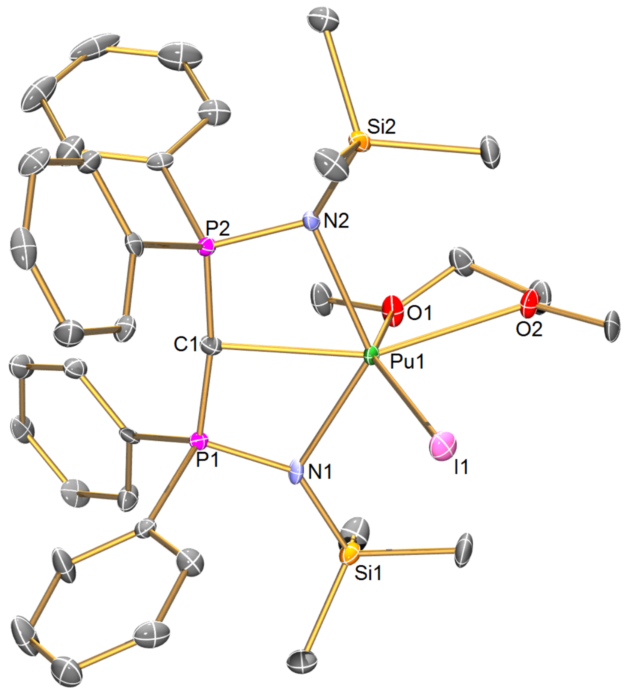

Figure S2. Molecular structure of [Pu(BIPM^{TMS})(I)(DME)] (**2Pu**) at 100 K with selected atom labels. Displacement ellipsoids are set at 40% and hydrogen atoms are omitted for clarity.

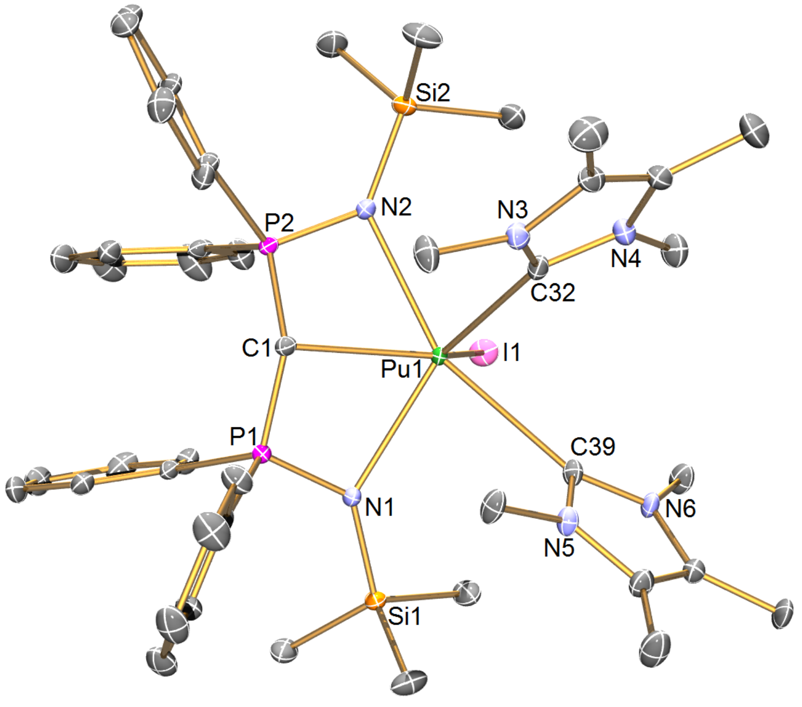

Figure S3. Molecular structure of [Pu(BIPM^{TMS})(I)(I^{Me4})$_2$] (**3Pu**) at 100 K with selected atom labels. Displacement ellipsoids are set at 40% and hydrogen atoms are omitted for clarity.

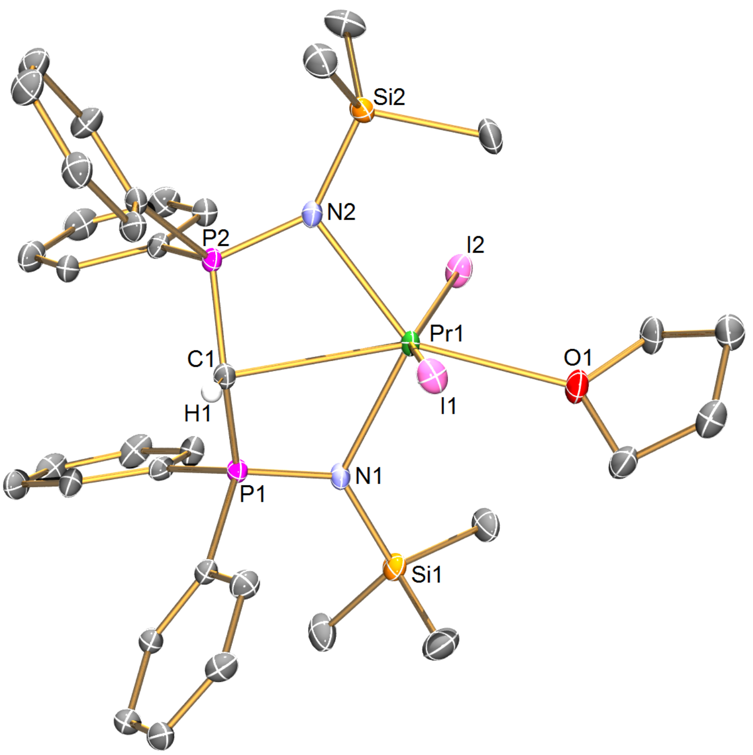

Figure S4. Molecular structure of [Pr(BIPM^{TMS}H)(I)$_2$(THF)] (**1Pr.THF**) at 120 K with selected atom labels. Displacement ellipsoids are set at 40% and non-methanide hydrogen atoms are omitted for clarity.

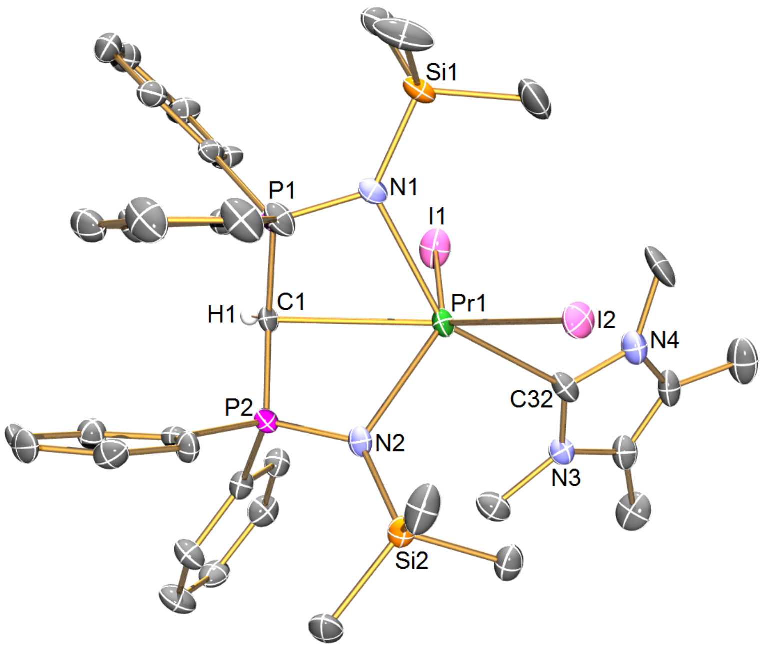

Figure S5. Molecular structure of [Pr(BIPM^{TMS}H)(I)$_2$(I^{Me4})] (**1Pr.IMe4**) at 150 K with selected atom labels. Displacement ellipsoids are set at 40% and non-methanide hydrogen atoms are omitted for clarity.

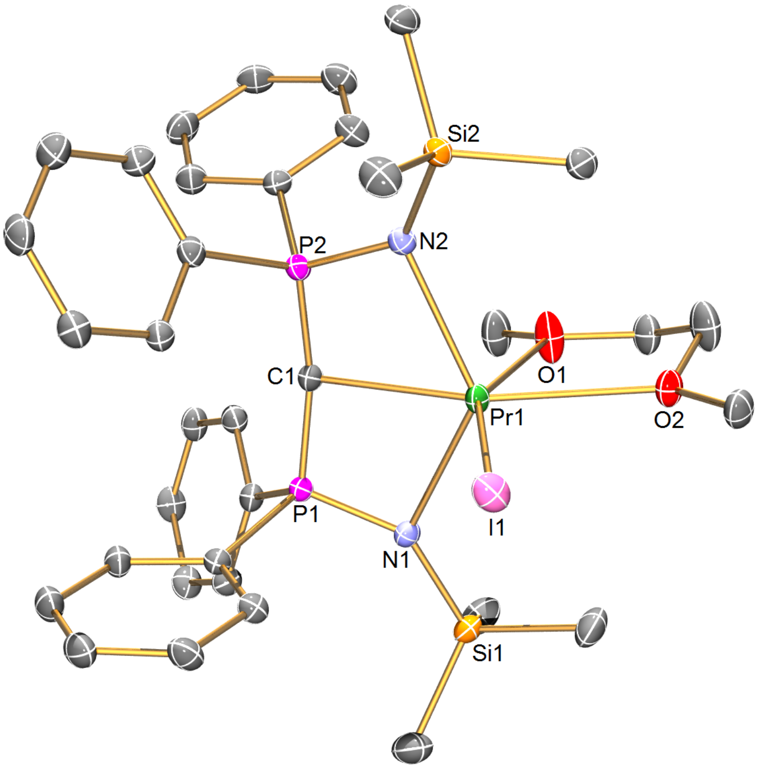

Figure S6. Molecular structure of [Pr(BIPM^{TMS})(I)(DME)] (**2Pr**) at 120 K with selected atom labels. Displacement ellipsoids are set at 40% and hydrogen atoms are omitted for clarity.

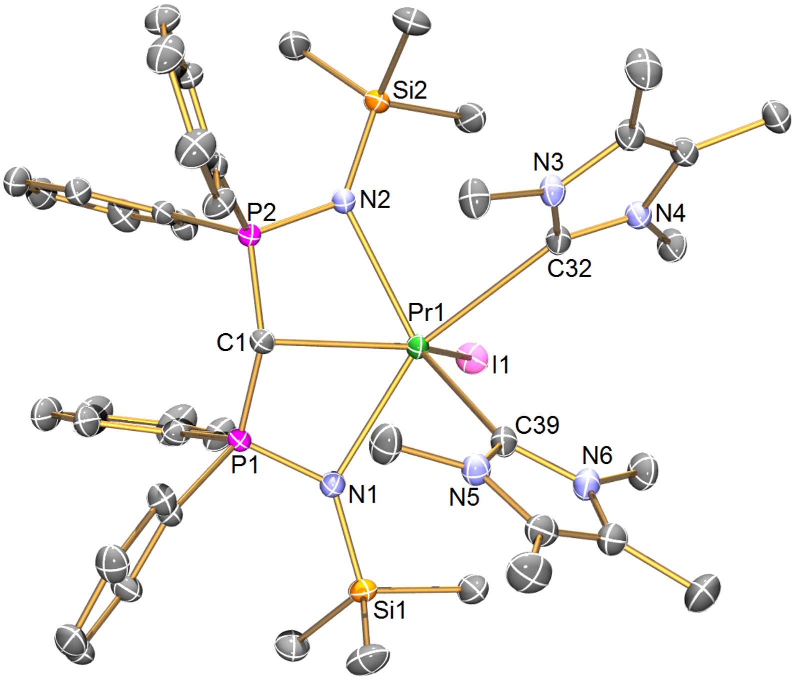

Figure S7. Molecular structure of [Pr(BIPM^{TMS})(I)(I^{Me4})$_2$] (**3Pr**) at 150 K with selected atom labels. Displacement ellipsoids are set at 40% and hydrogen atoms are omitted for clarity.

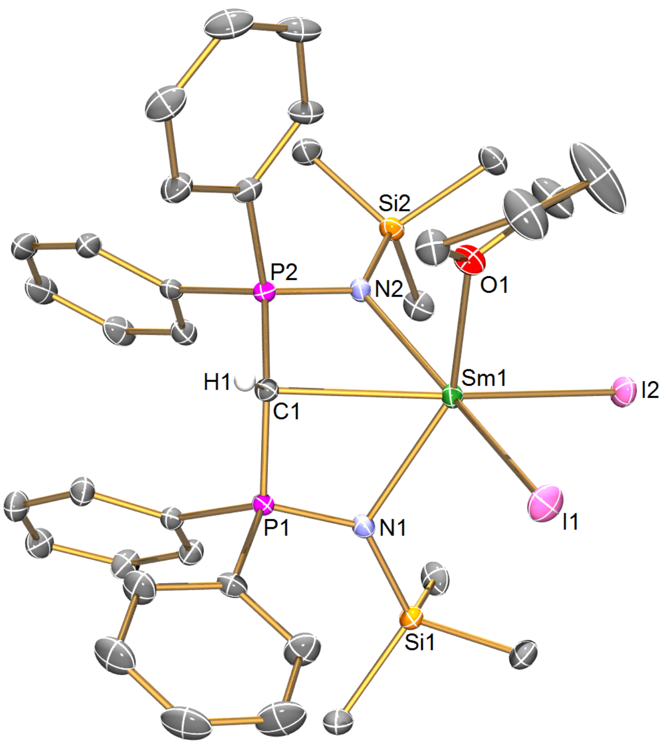

Figure S8. Molecular structure of [Sm(BIPM^{TMS}H)(I)$_2$(THF)] (**1Sm.THF**) at 150 K with selected atom labels. Displacement ellipsoids are set at 40% and non-methanide hydrogen atoms are omitted for clarity.

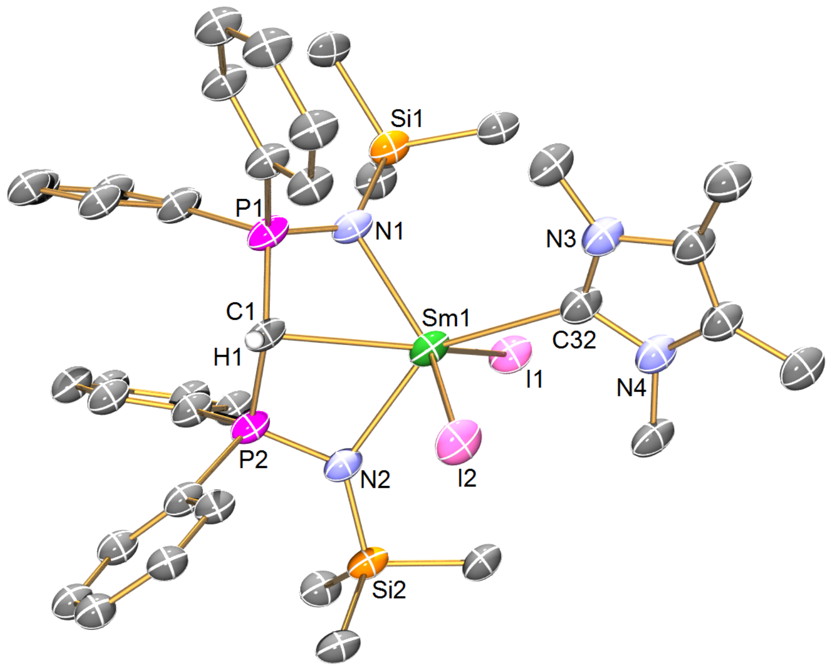

Figure S9. Molecular structure of [Sm(BIPM^{TMS}H)(I)$_2$(I^{Me4})] (**1Sm.IMe4**) at 150 K with selected atom labels. Displacement ellipsoids are set at 20% and non-methanide hydrogen atoms are omitted for clarity.

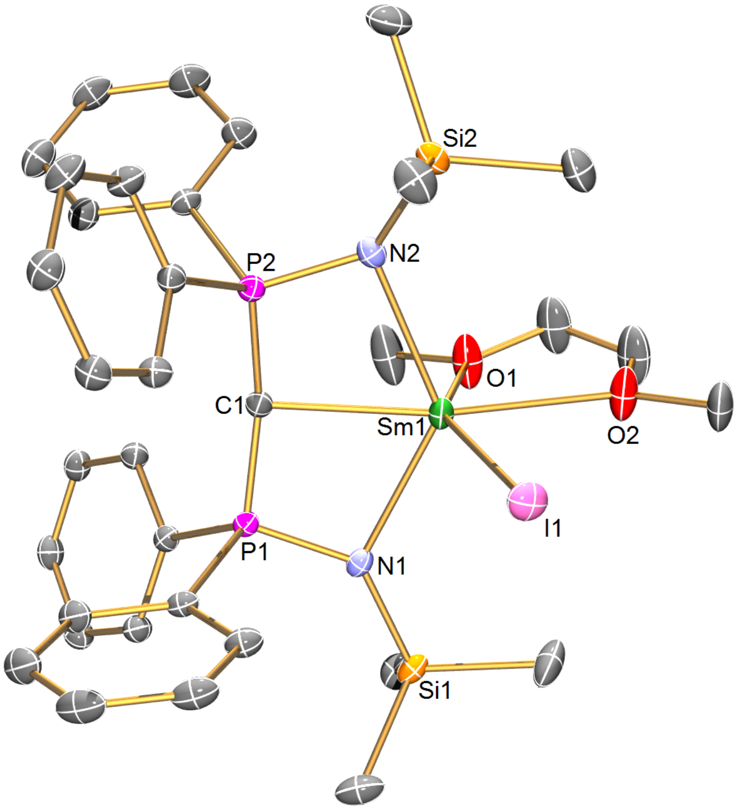

Figure S10. Molecular structure of [Sm(BIPM^{TMS})(I)(DME)] (**2Sm**) at 150 K with selected atom labels. Displacement ellipsoids are set at 40% and hydrogen atoms are omitted for clarity.

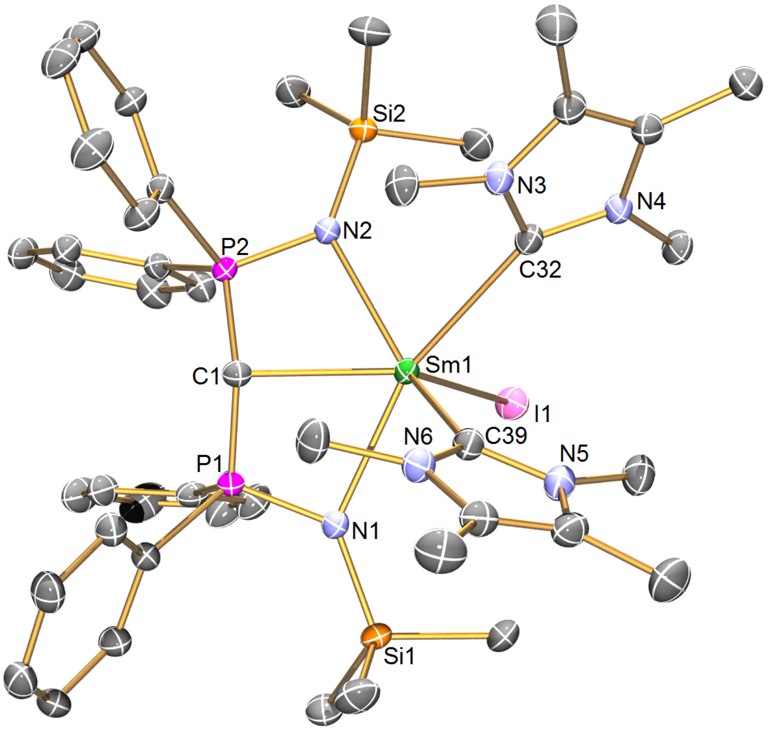

Figure S11. Molecular structure of [Sm(BIPM^{TMS})(I)(I^{Me4})$_2$] (**3Sm**) at 150 K with selected atom labels. Displacement ellipsoids are set at 40% and hydrogen atoms are omitted for clarity.

NMR Spectroscopy

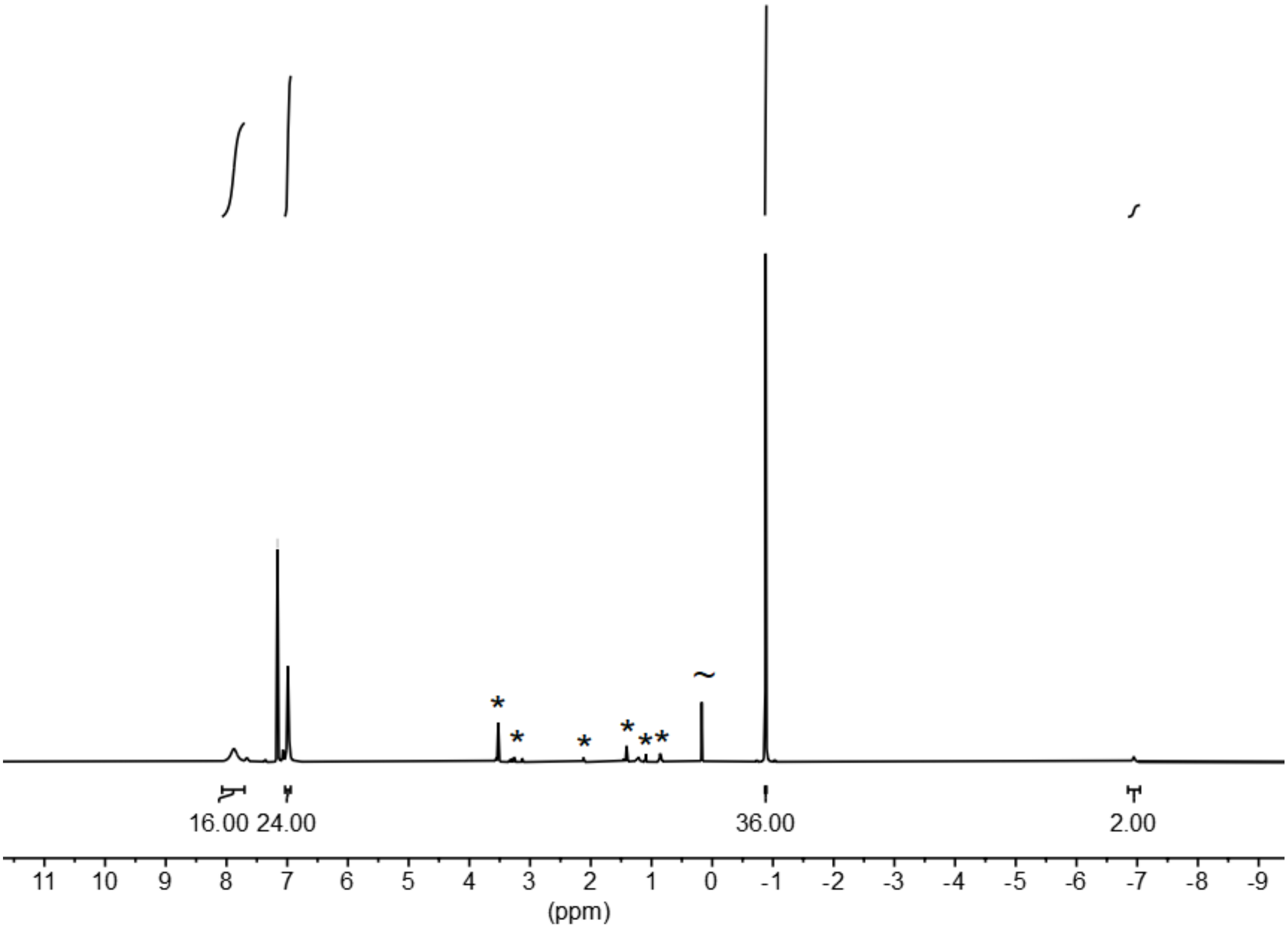

Figure S12. ^{1}H NMR spectrum of $[Pu(BIPM^{TMS}H)(I)(\mu\text{-}I)]_2$ (**1Pu**) in in C_6D_6 with a small volume of D_8-THF added to aid solubility. The tilde (~) denotes an impurity of $BIPM^{TMS}H_2$ likely from a small amount of decomposition given the limitations of our sample containment procedure. The asterisks (*) denote trace solvent impurities: THF (δ ~3.57 (m), 1.40 (m) ppm), toluene (δ ~2.11 (s) ppm), diethyl ether (δ ~3.26 (q), 1.11 (t) ppm), *n*-hexane (δ ~1.24 (m), 0.89 (t) ppm) and *n*-pentane (δ ~1.23 (m), 0.87 (t) ppm).

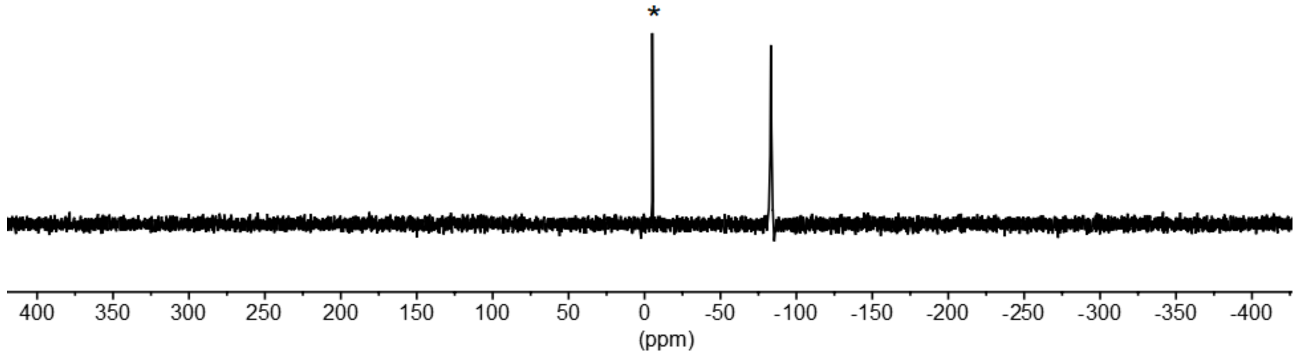

Figure S13. $^{31}P\{^1H\}$ NMR spectrum of $[Pu(BIPM^{TMS}H)(I)(\mu\text{-}I)]_2$ (**1Pu**) in C_6D_6 with a small volume of D_8-THF added to aid solubility. The asterisk (*) at ~ –5 ppm denotes an impurity of $BIPM^{TMS}H_2$.

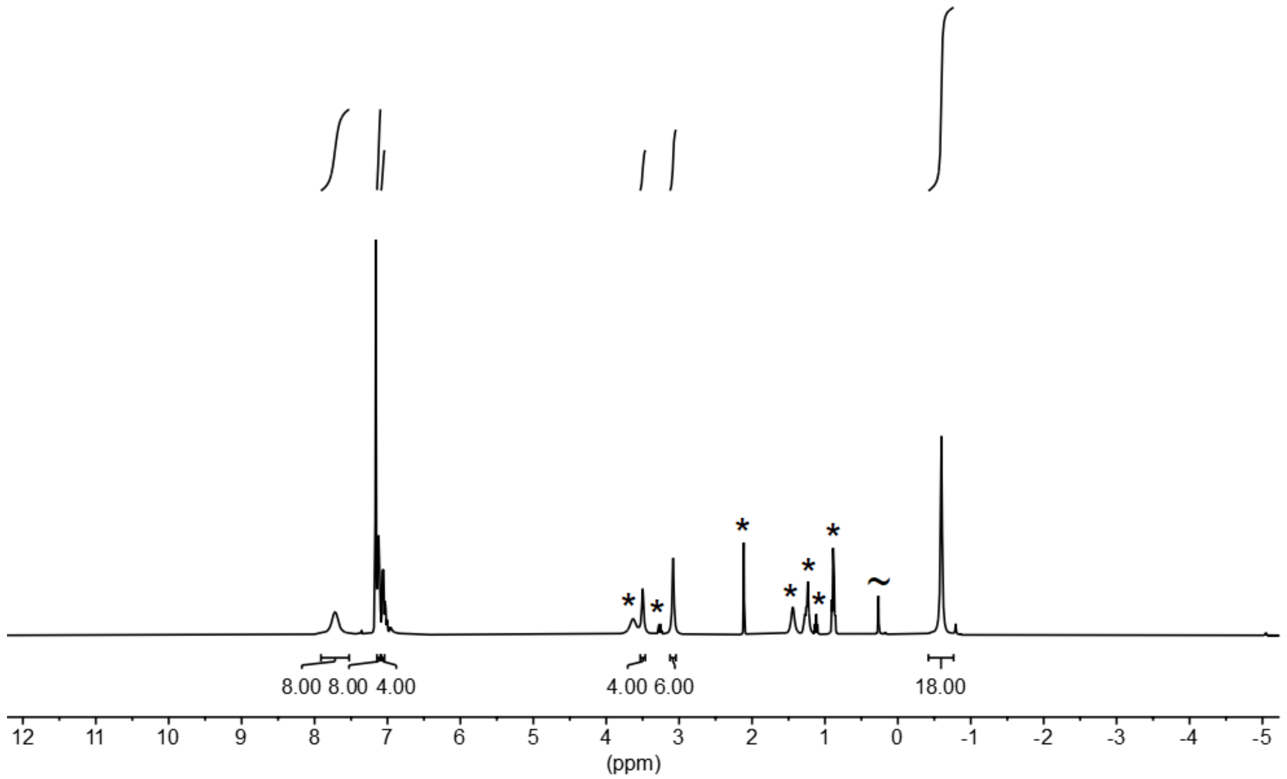

Figure S14. ^1H NMR spectrum of $[(BIPM^{TMS})Pu(I)(DME)]$ (**2Pu**) in C_6D_6. The tilde (~) denotes an impurity of $BIPM^{TMS}H_2$ likely from a small amount of decomposition given the limitations of our sample containment procedure. The asterisks (*) denote trace solvent impurities: THF (δ ~3.57 (m), 1.40 (m) ppm), toluene (δ ~2.11 (s) ppm), diethyl ether (δ ~3.26 (q), 1.11 (t) ppm), *n*-hexane (δ ~1.24 (m), 0.89 (t) ppm) and *n*-pentane (δ ~1.23 (m), 0.87 (t) ppm).

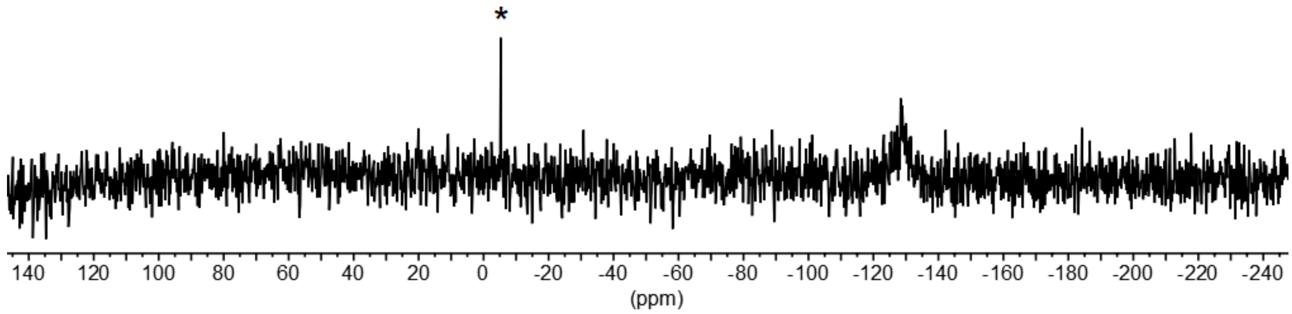

Figure S15. $^{31}P\{^{1}H\}$ NMR spectrum of [Pu(BIPM^{TMS})(I)(DME)] (**2Pu**) in C_6D_6. The asterisk (*) at ~ –5 ppm denotes an impurity of BIPM^{TMS}H$_2$.

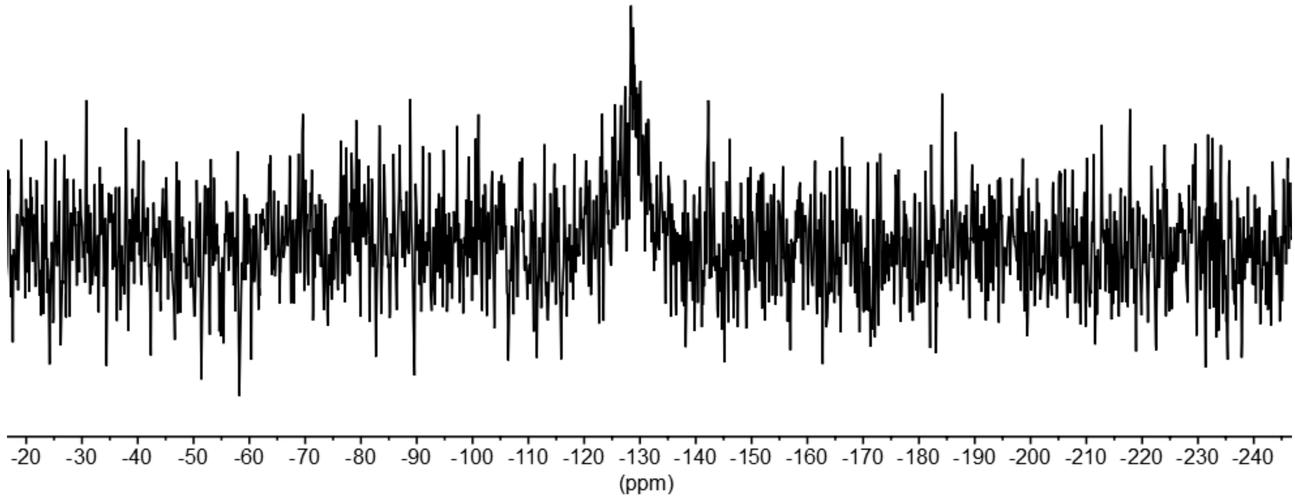

Figure S16. $^{31}P\{^{1}H\}$ NMR spectrum of [Pu(BIPM^{TMS})(I)(DME)] (**2Pu**) in C_6D_6 – zoomed in between ~ –5 and –240 ppm.

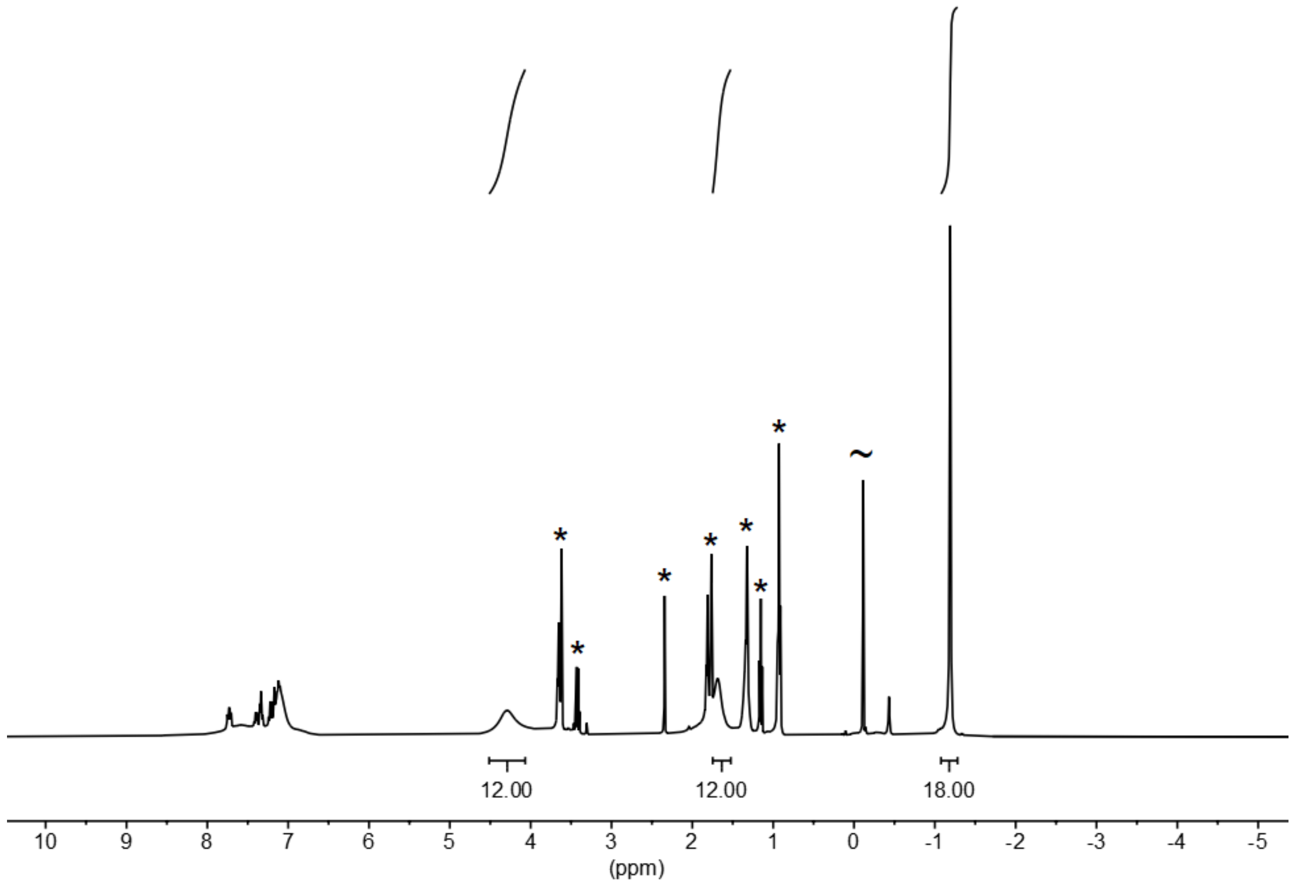

Figure S17. ^{1}H NMR spectrum of [Pu(BIPM^{TMS})(I)(I^{Me4})$_2$] (**3Pu**) in D_8-THF. The tilde (~) denotes an impurity of BIPM^{TMS}H$_2$ likely from a small amount of decomposition given the limitations of our sample containment procedure. The asterisks (*) denote trace solvent impurities: THF (δ ~3.65 (m), 1.77 (m) ppm), toluene (δ ~2.34 (s) ppm), diethyl ether (δ ~3.41 (q), 1.15 (t) ppm), and *n*-pentane (δ ~1.33 (m), 0.93 (t) ppm).

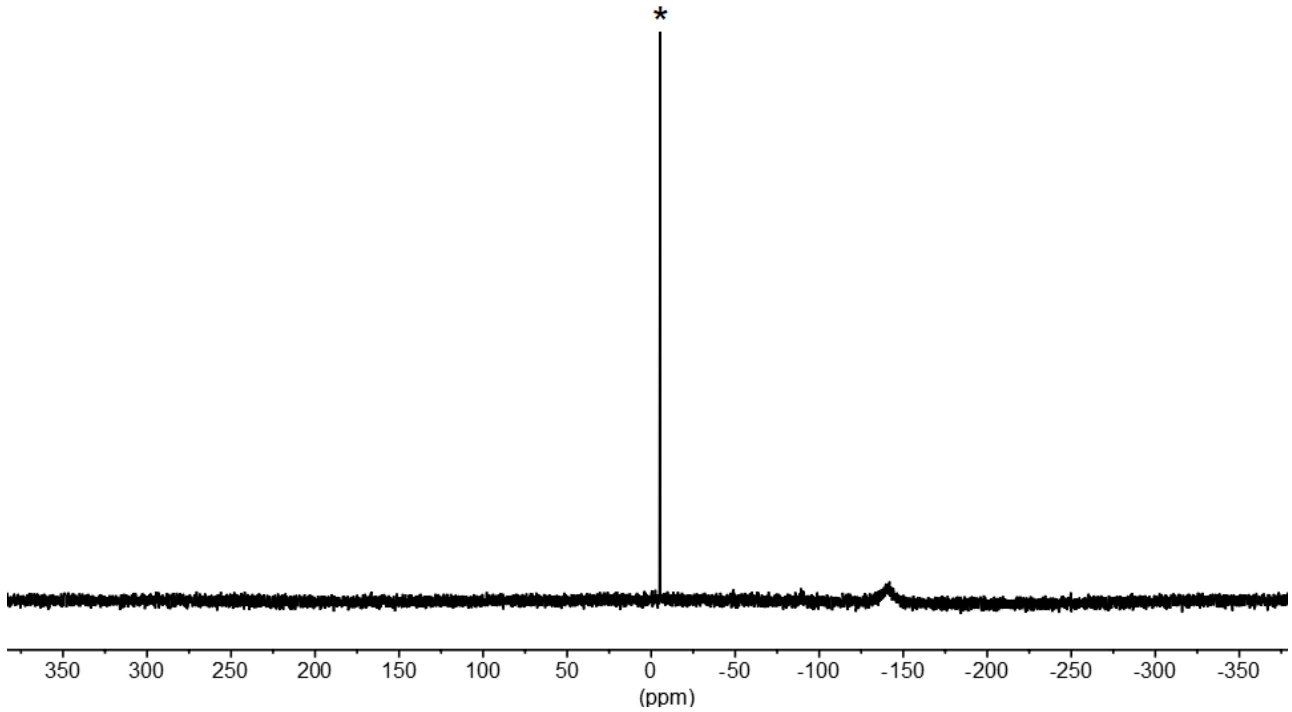

Figure S18. $^{31}P\{^{1}H\}$ NMR spectrum of [Pu(BIPM^{TMS})(I)(I^{Me4})$_2$)] (**3Pu**) in D_8-THF. The asterisk (*) at ~ –5 ppm denotes an impurity of BIPM^{TMS}H$_2$.

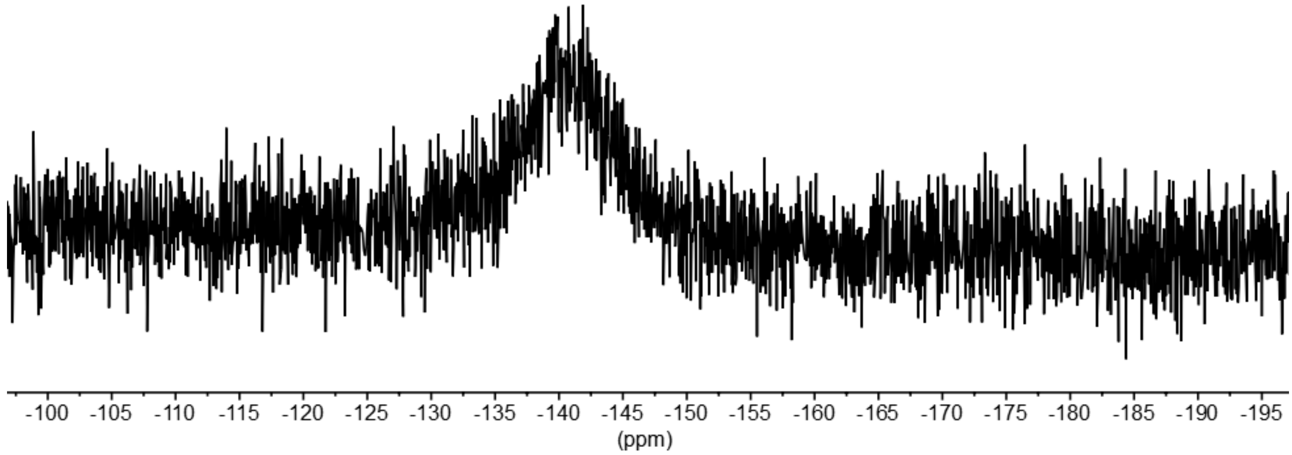

Figure S19. $^{31}P\{^{1}H\}$ NMR spectrum of [Pu(BIPM^{TMS})(I)(I^{Me4})$_2$)] (**3Pu**) in D_8-THF – zoomed in between ~ –100 and –195 ppm.

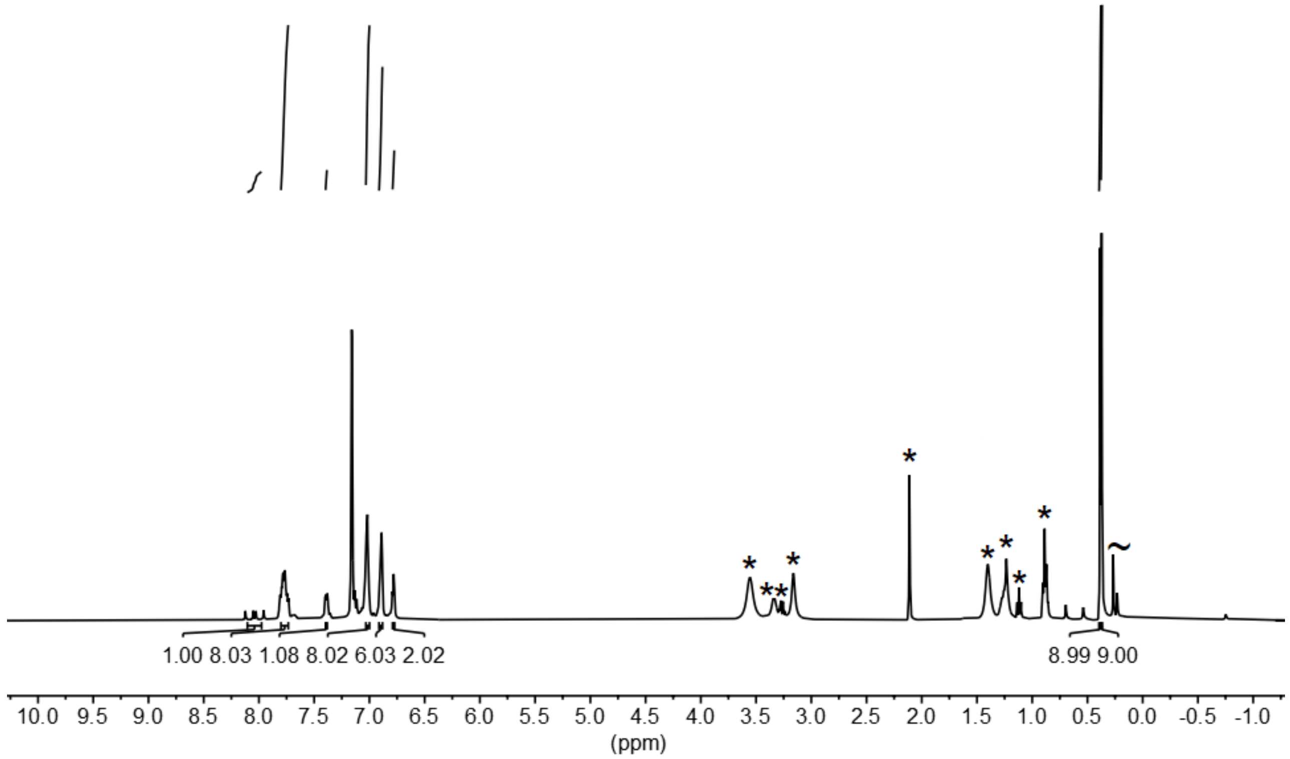

Figure S20. ^{1}H NMR spectrum of *in situ* generated [PhC(H)=C(PPh$_2$NSiMe$_3$)$_2$] (**4**) in C_6D_6 zoomed in between ~ 10 and –1 ppm. The tilde (~) denotes an impurity of BIPM^{TMS}H$_2$. The asterisks (*) denote trace solvent impurities: THF (δ ~3.55 (m), 1.40 (m) ppm), DME (δ ~3.34 (s), 3.16 (s) ppm), toluene (δ ~2.11 (s) ppm), diethyl ether (δ ~3.26 (q), 1.12 (t) ppm), and *n*-pentane (δ ~1.24 (m), 0.89 (t) ppm).

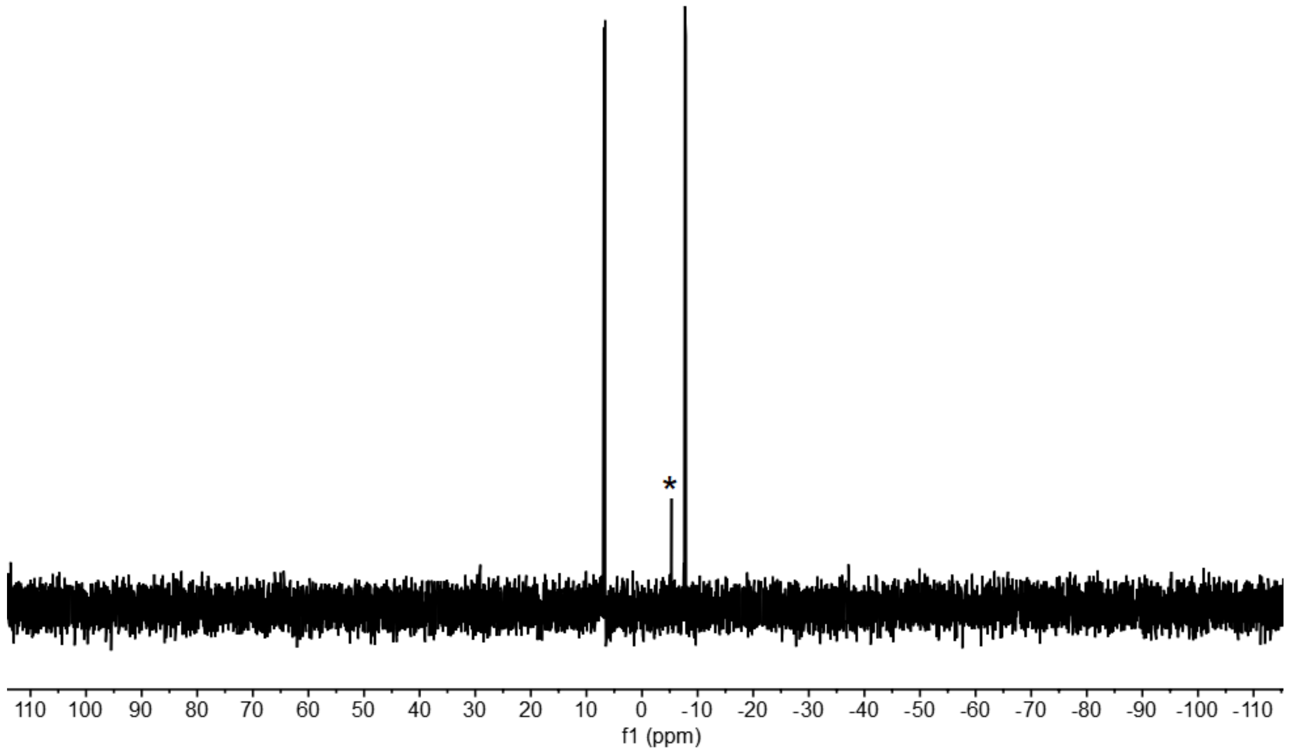

Figure S21. $^{31}P\{^{1}H\}$ NMR spectrum of *in situ* generated [PhC(H)=C(PPh$_2$NSiMe$_3$)$_2$] (**4**) in C_6D_6. The asterisk (*) at ~ –5 ppm denotes an impurity of BIPM^{TMS}H$_2$.

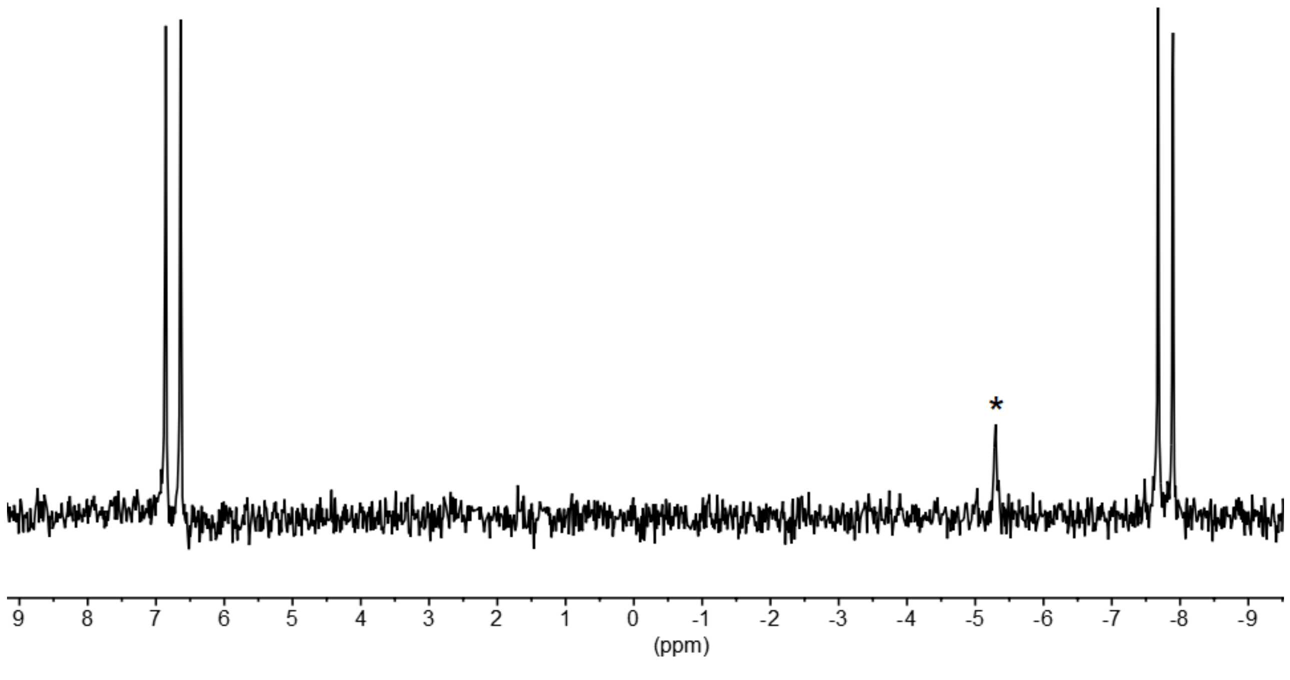

Figure S22. $^{31}P\{^{1}H\}$ NMR spectrum of *in situ* generated [PhC(H)=C(PPh$_2$NSiMe$_3$)$_2$] (**4**) in C_6D_6 – zoomed in between ~ 9 and –9 ppm. The asterisk (*) at ~ –5 ppm denotes an impurity of BIPM^{TMS}H$_2$.

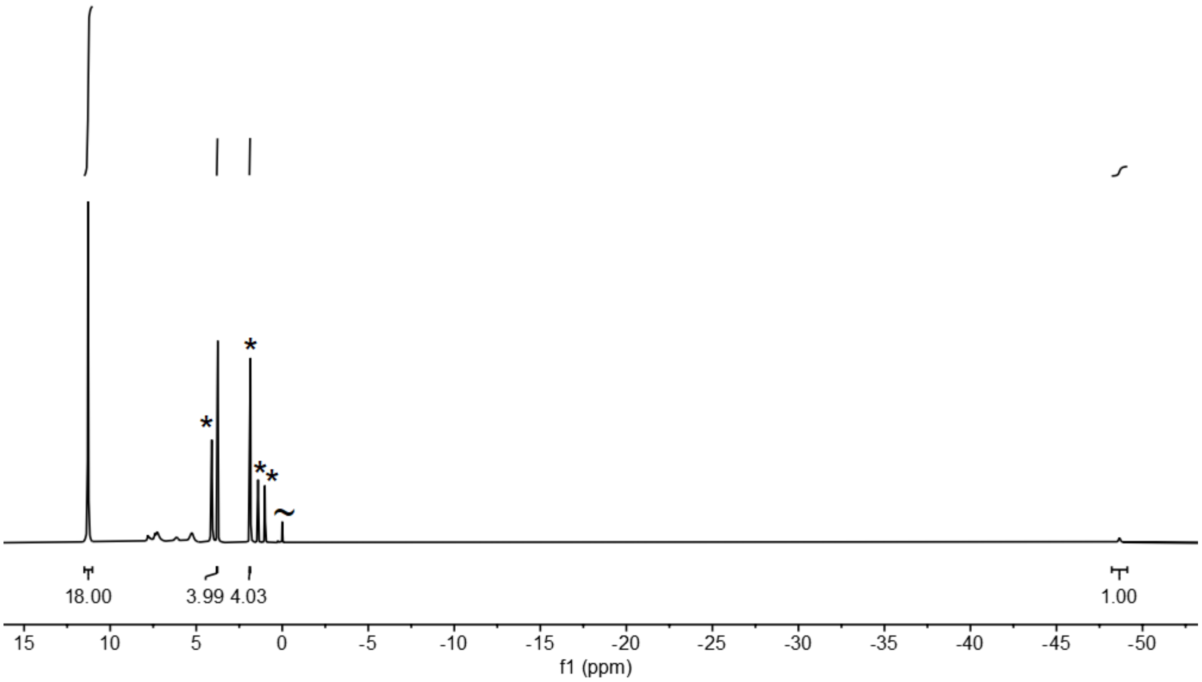

Figure S23. ^{1}H NMR spectrum of [Pr(BIPM^{TMS}H)(I)$_2$(THF)] (**1Pr.THF**) in D$_8$-THF. The tilde (~) denotes an impurity of BIPM^{TMS}H$_2$. The asterisks (*) denote trace solvent impurities: THF (δ ~3.65 (m), 1.77 (m) ppm) and *n*-pentane (δ ~1.33 (m), 0.93 (t) ppm).

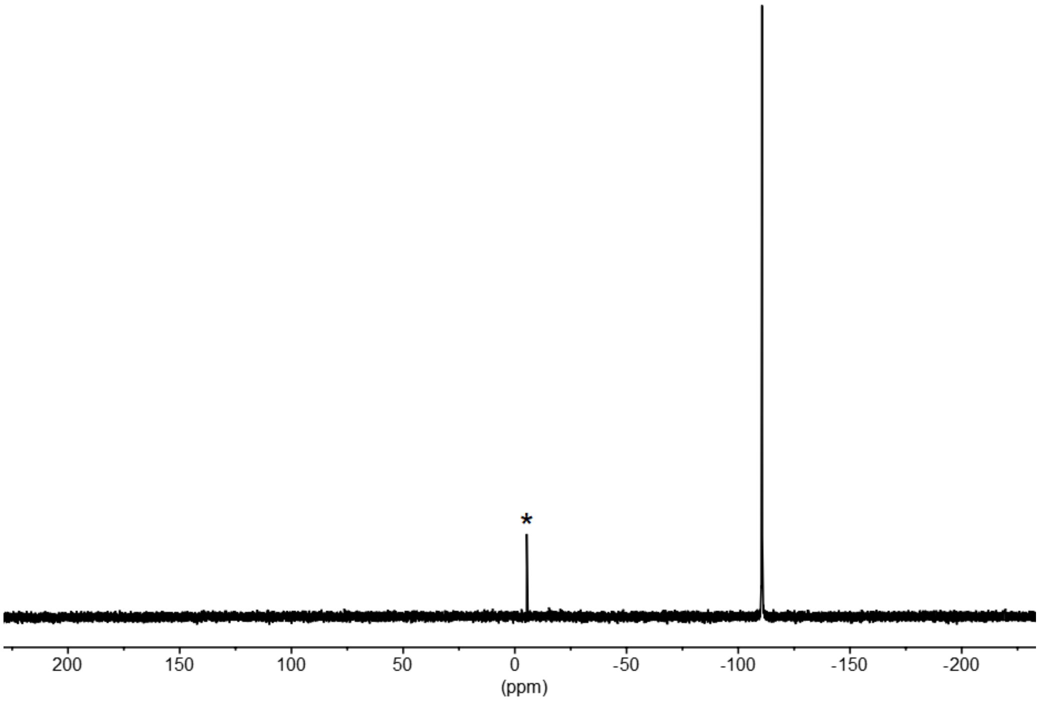

Figure S24. ^{31}P{^{1}H} NMR spectrum of [Pr(BIPM^{TMS}H)(I)$_2$(THF)] (**1Pr.THF**) in D$_8$-THF. The asterisk (*) at ~ –5 ppm denotes an impurity of BIPM^{TMS}H$_2$.

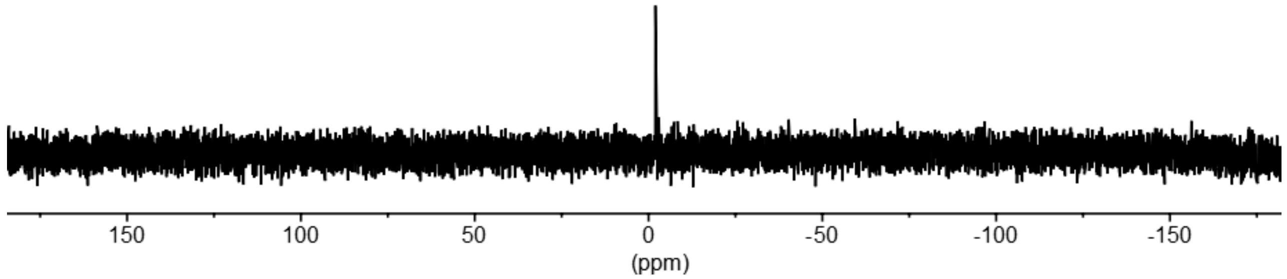

Figure S25. $^{29}Si\{^1H\}$ NMR spectrum of [Pr(BIPM^{TMS}H)(I)$_2$(THF)] (**1Pr.THF**) in D$_8$-THF.

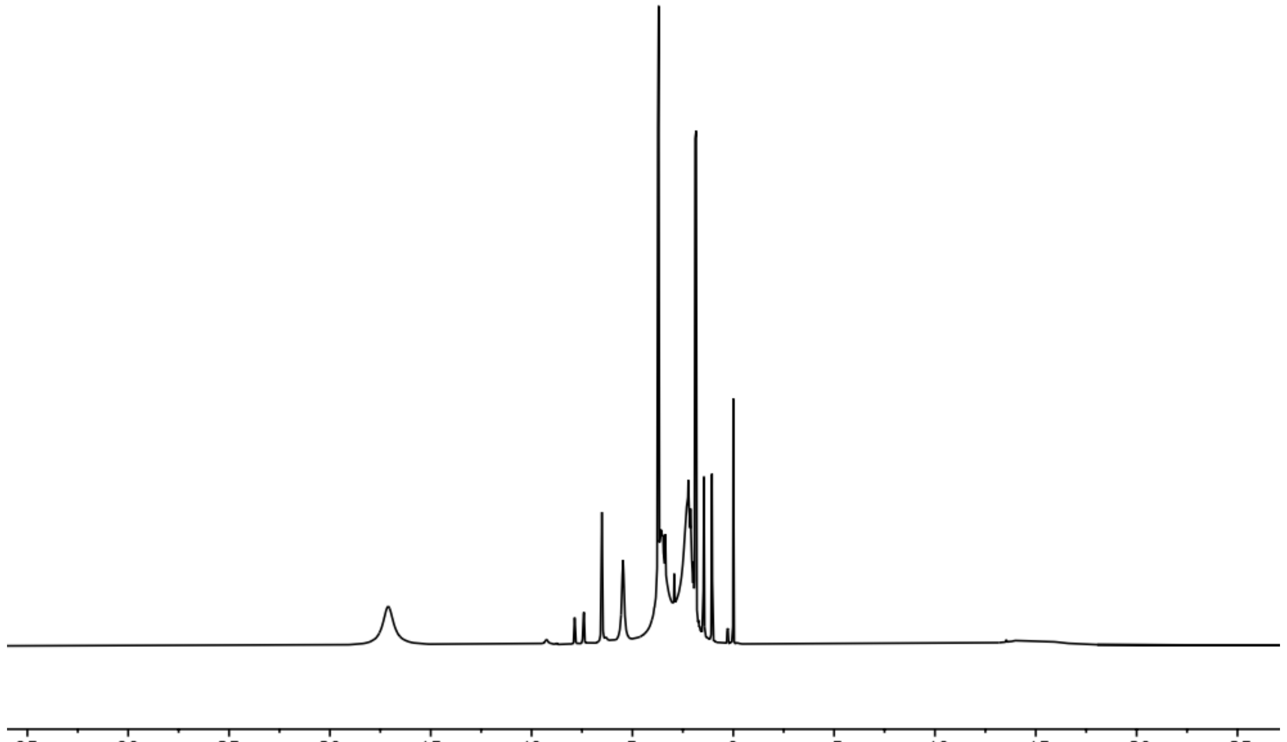

Figure S26. ^1H NMR spectrum of [Pr(BIPM^{TMS}H)(I)$_2$(I^{Me4})] (**1Pr.IMe4**) in D$_8$-THF.

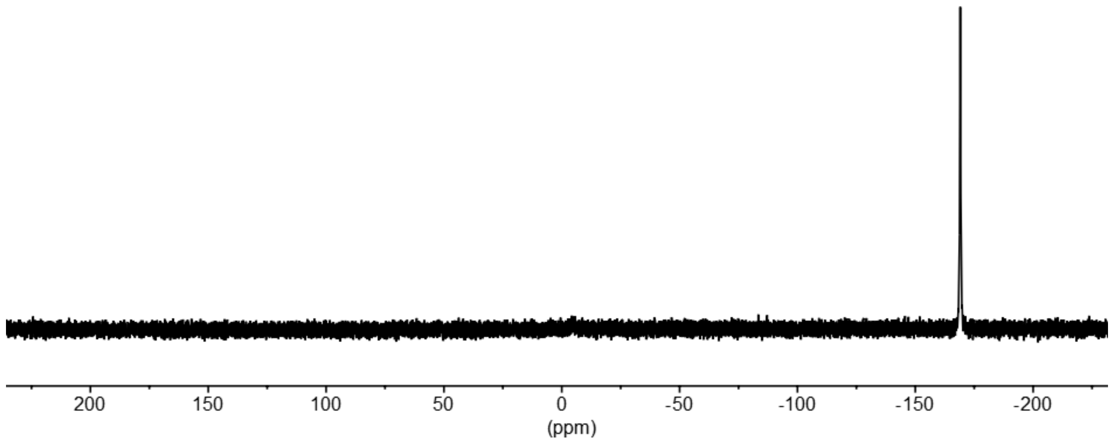

Figure S27. $^{31}P\{^{1}H\}$ NMR spectrum of $[Pr(BIPM^{TMS}H)(I)_2(I^{Me4})]$ (**1Pr.IMe4**) in D_8-THF.

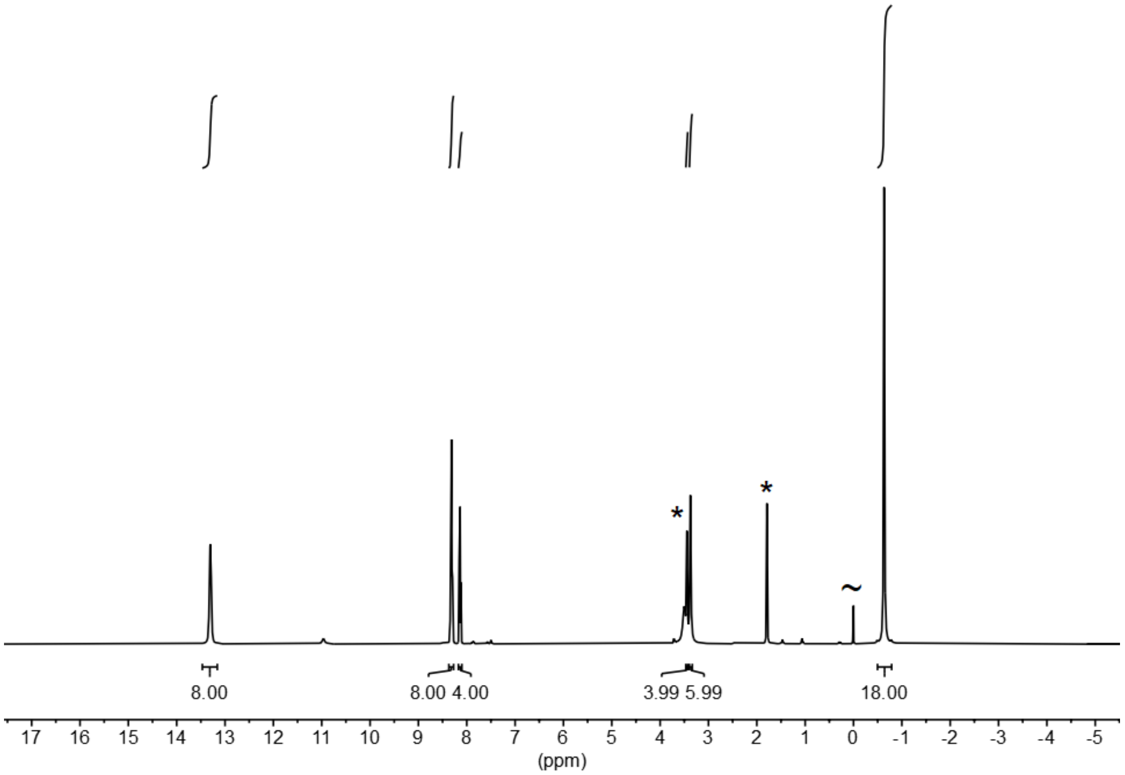

Figure S28. ^{1}H NMR spectrum of $[Pr(BIPM^{TMS})(I)(DME)]$ (**2Pr**) in D_8-THF. The tilde (~) denotes an impurity of $BIPM^{TMS}H_2$. The asterisks (*) denote trace solvent impurities: THF (δ ~3.65 (m), 1.77 (m) ppm).

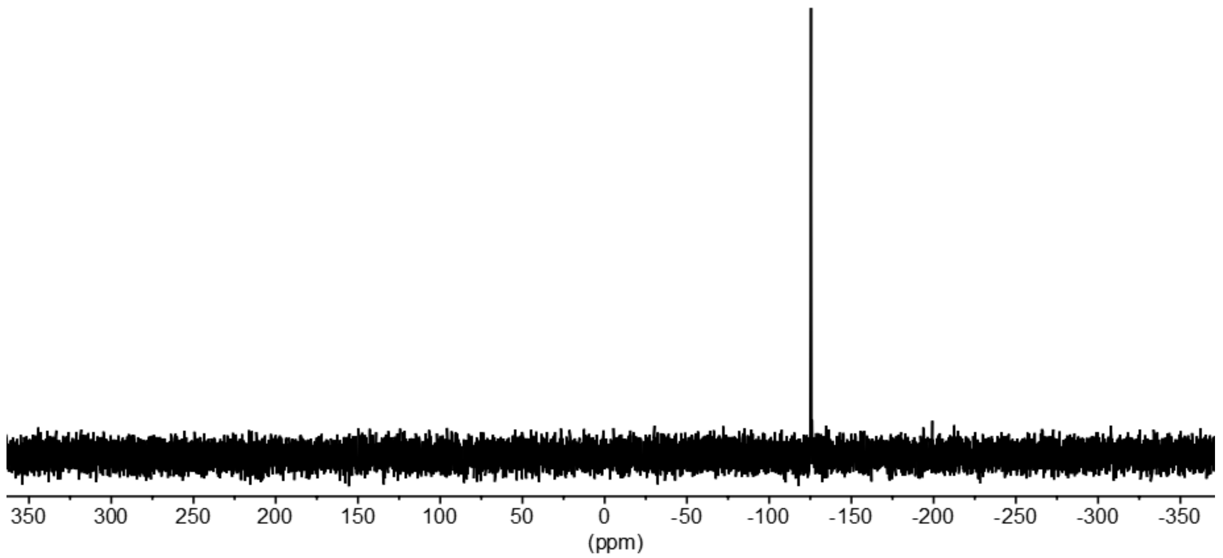

Figure S29. $^{31}P\{^{1}H\}$ NMR spectrum of [Pr(BIPM^{TMS})(I)(DME)] (**2Pr**) in D_8-THF.

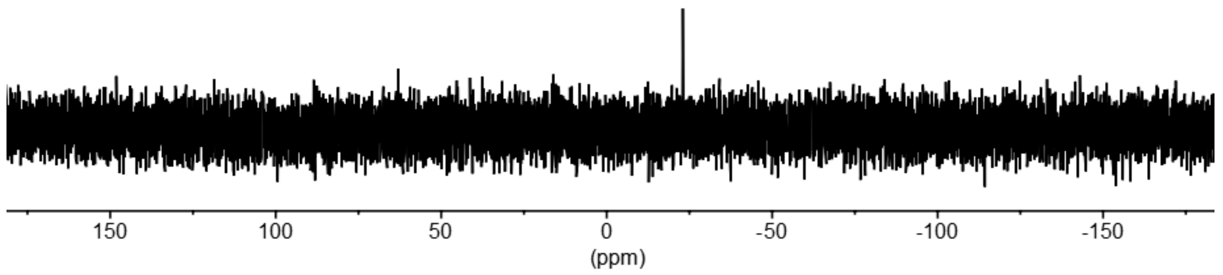

Figure S30. $^{29}Si\{^{1}H\}$ NMR spectrum of [Pr(BIPM^{TMS})(I)(DME)] (**2Pr**) in D_8-THF.

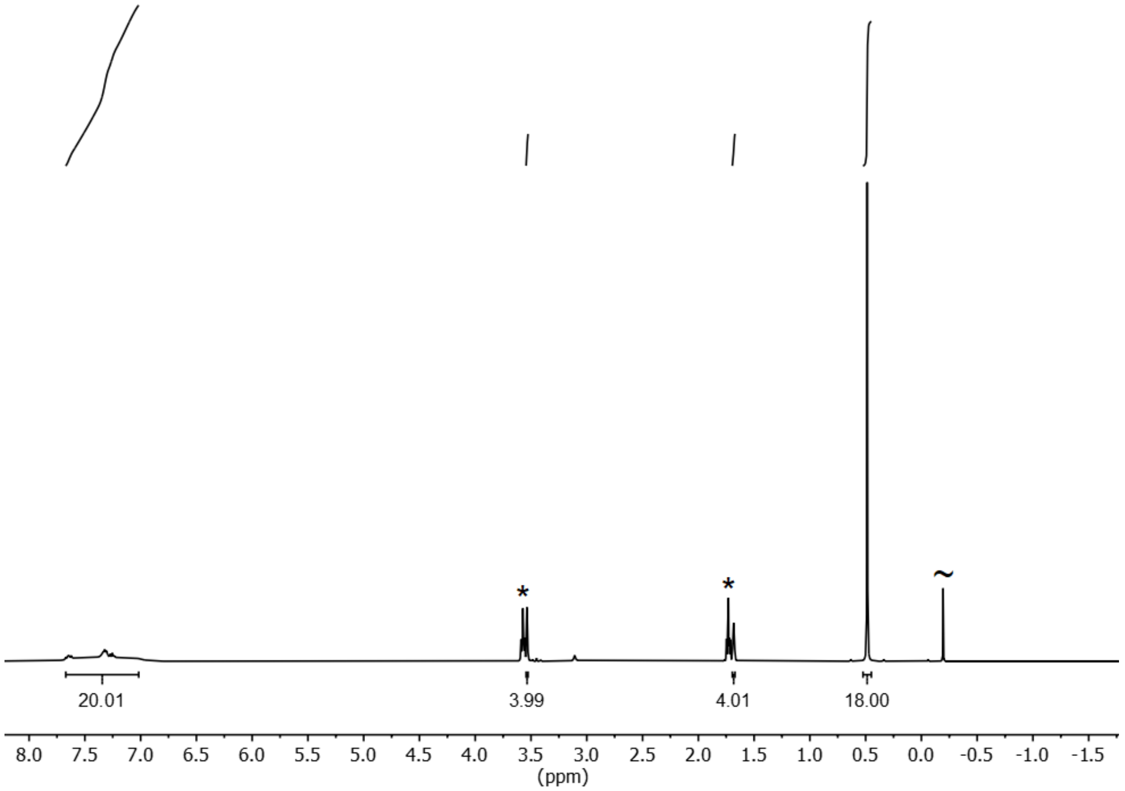

Figure S31. ^{1}H NMR spectrum of [Sm(BIPM^{TMS}H)(I)$_2$(THF)] (**1Sm.THF**) in D_8-THF. The tilde (~) denotes an impurity of BIPM^{TMS}H$_2$. The asterisks (*) denote trace solvent impurities: THF (δ ~3.57 (m), 1.73 (m) ppm).

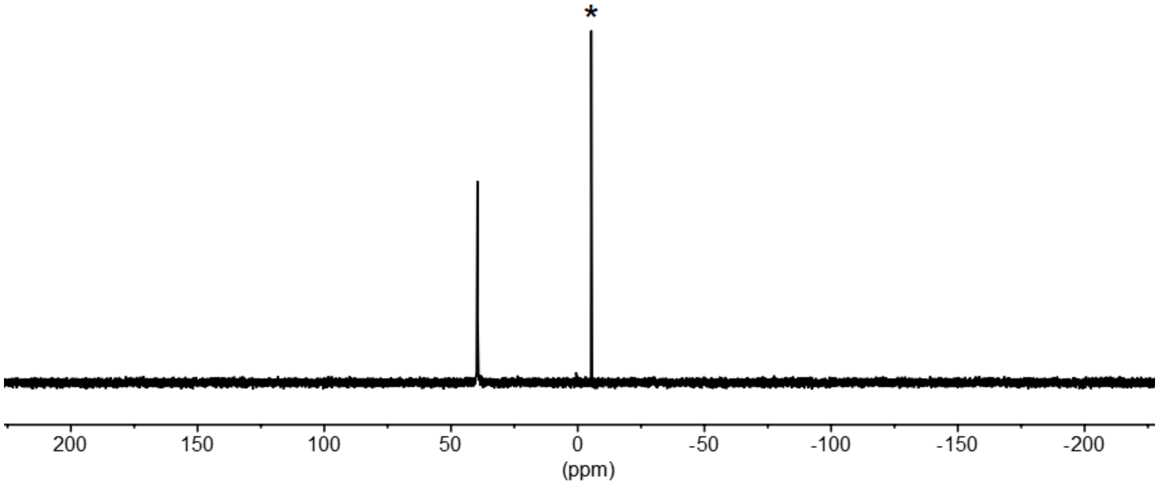

Figure S32. $^{31}P\{^{1}H\}$ NMR spectrum of [Sm(BIPM^{TMS}H)(I)$_2$(THF)] (**1Sm.THF**) in D_8-THF. The asterisk (*) at ~ –5 ppm denotes an impurity of BIPM^{TMS}H$_2$.

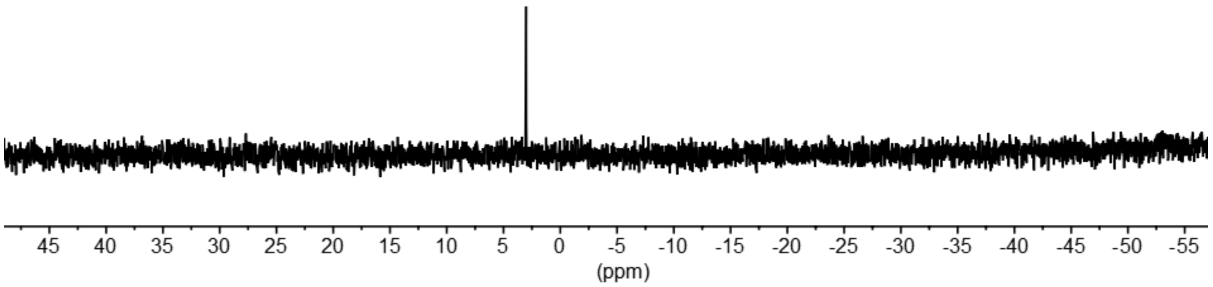

Figure S33. $^{29}Si\{^{1}H\}$ NMR spectrum of [Sm(BIPM^{TMS}H)(I)$_2$(THF)] (**1Sm.THF**) in D$_8$-THF.

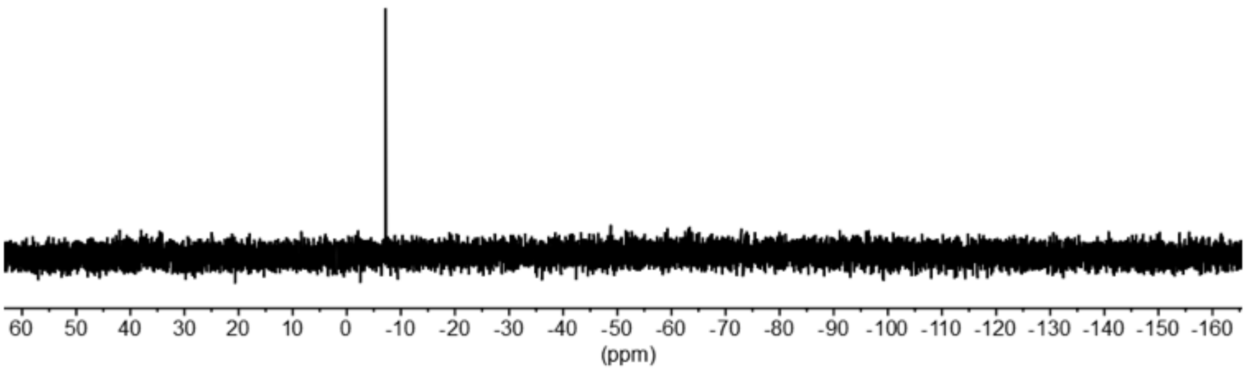

Figure S34. $^{31}P\{^{1}H\}$ NMR spectrum of [Sm(BIPM^{TMS}H)(I)$_2$(I^{Me4})] (**1Sm.IMe4**) in D$_8$-THF.

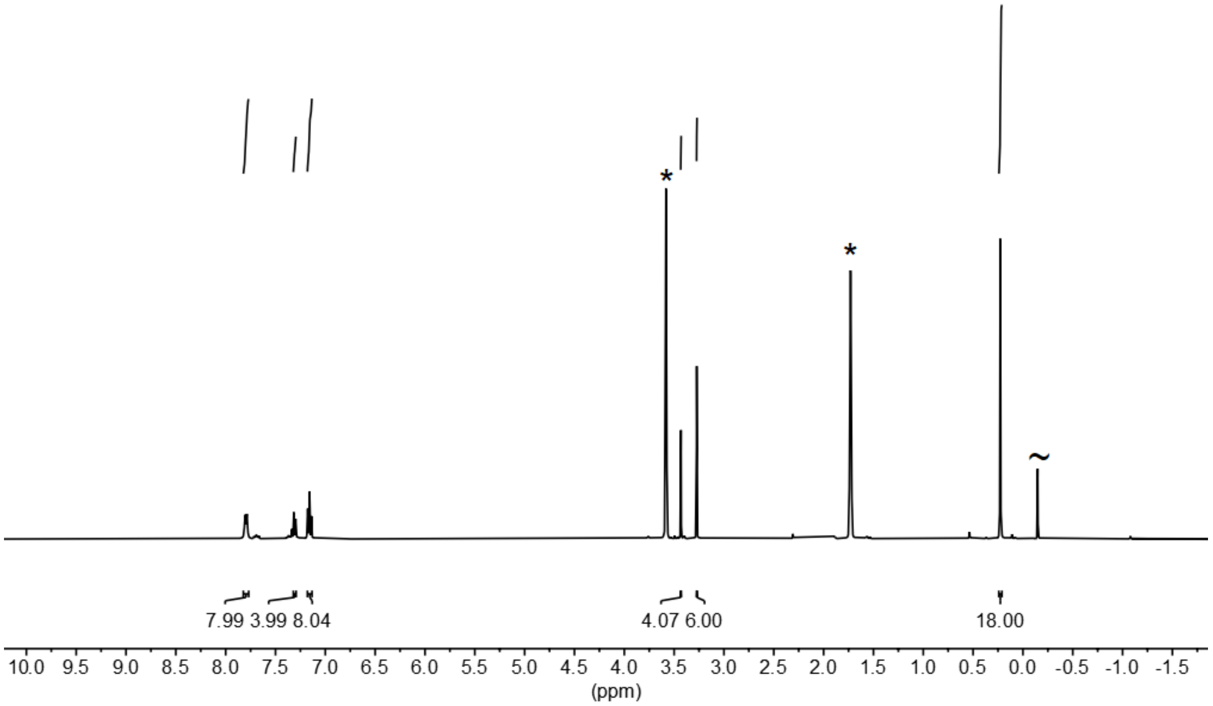

Figure S35. ^{1}H NMR spectrum of [Sm(BIPM^{TMS})(I)(DME)] (**2Sm**) in D$_8$-THF. The tilde (~) denotes an impurity of BIPM^{TMS}H$_2$. The asterisks (*) denote trace solvent impurities: THF (δ ~3.57 (m), 1.73 (m) ppm).

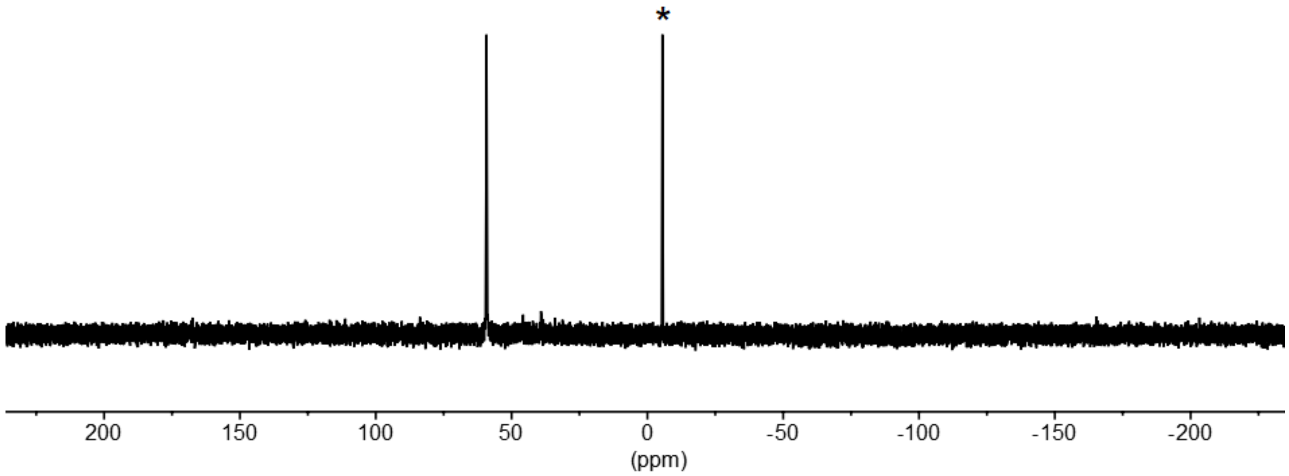

Figure S36. $^{31}P\{^{1}H\}$ NMR spectrum of [Sm(BIPM^{TMS})(I)(DME)] (**2Sm**) in D_8-THF with a few drops of C_6D_6. The asterisk (*) at ~ –5 ppm denotes an impurity of BIPM^{TMS}H_2.

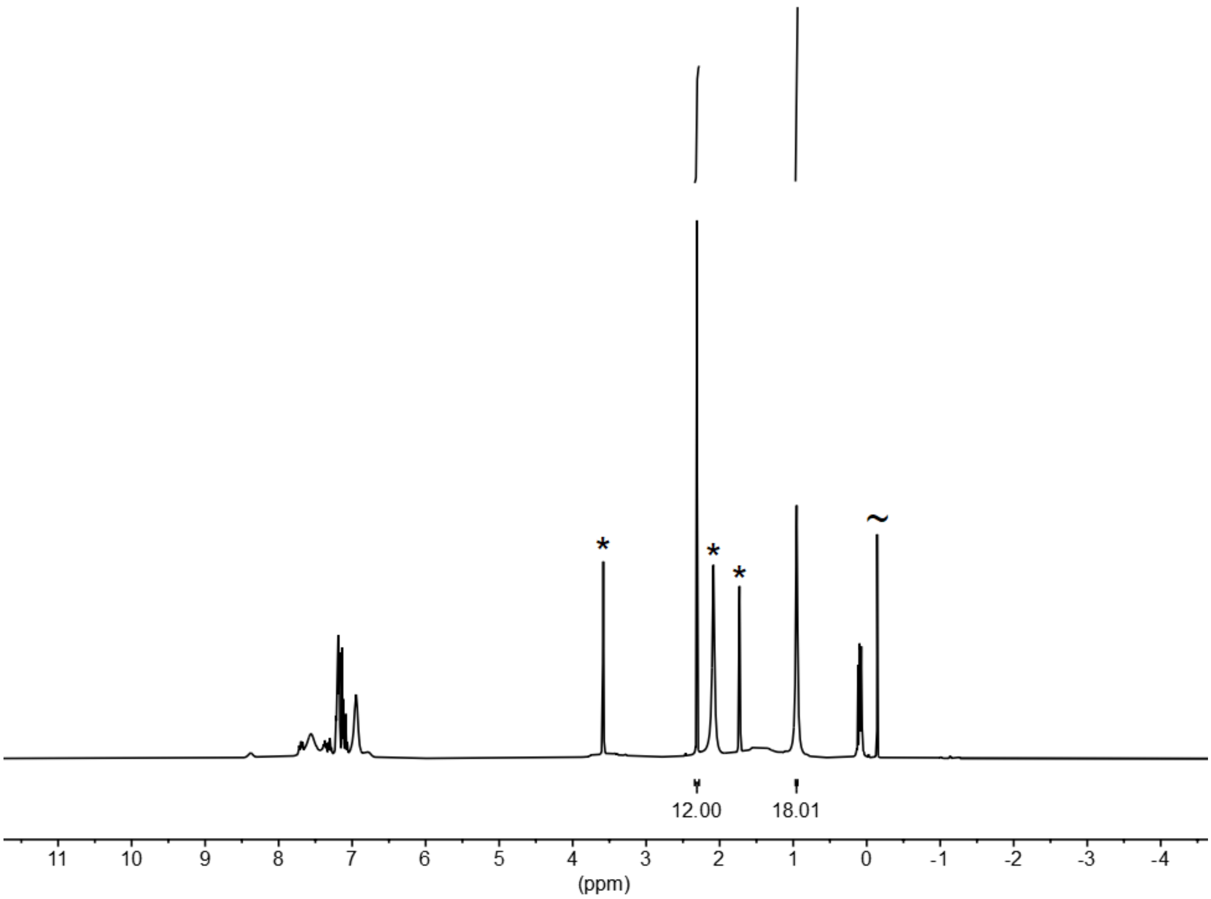

Figure S37. ^{1}H NMR spectrum of [Sm(BIPM^{TMS})(I)(I^{Me4})$_2$] (**3Sm**) in D_8-THF. The tilde (~) denotes an impurity of BIPM^{TMS}H_2. The asterisks (*) denote trace solvent impurities: THF (δ ~3.58 (m), 1.73 (m) ppm), and toluene (δ ~2.08 (s) ppm).

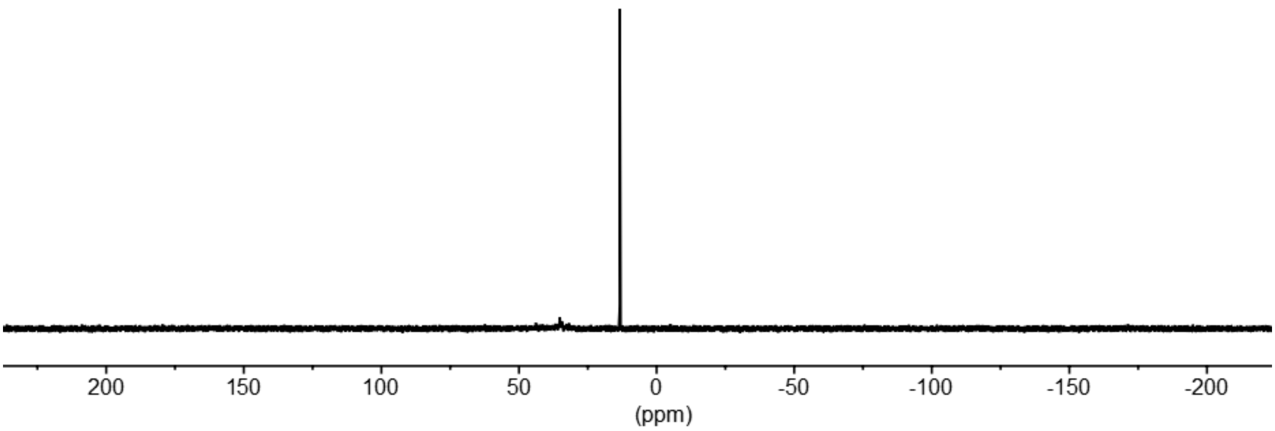

Figure S38. $^{31}P\{^{1}H\}$ NMR spectrum of $[Sm(BIPM^{TMS})(I)(I^{Me4})_2]$ (**3Sm**) in D_8-THF.

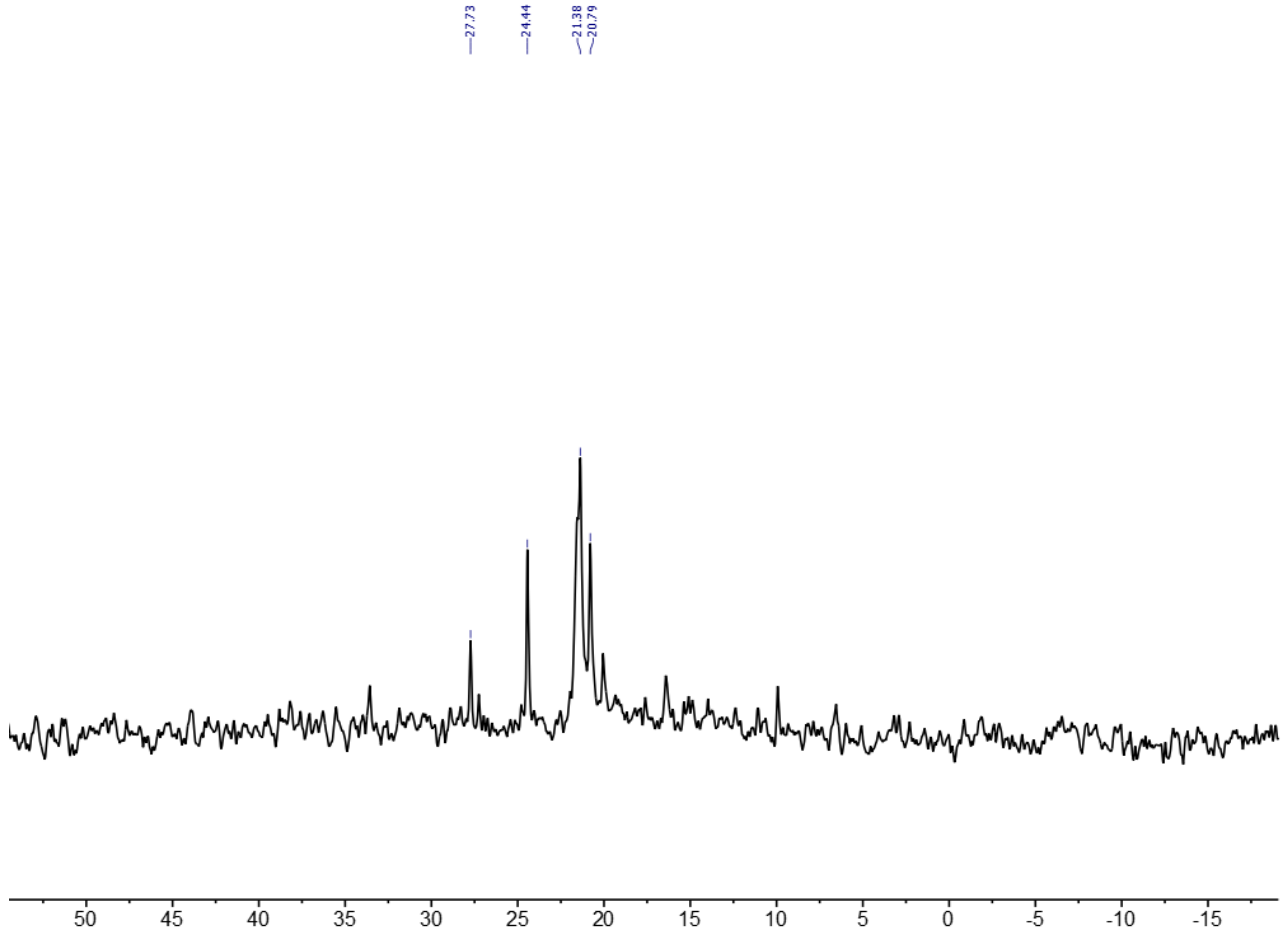

Figure S39. $^{31}P\{^{1}H\}$ NMR spectrum of the attempted reaction between $[Ce(BIPM^{TMS})(I)(DME)]$ (**2Ce**) and PhCHO.

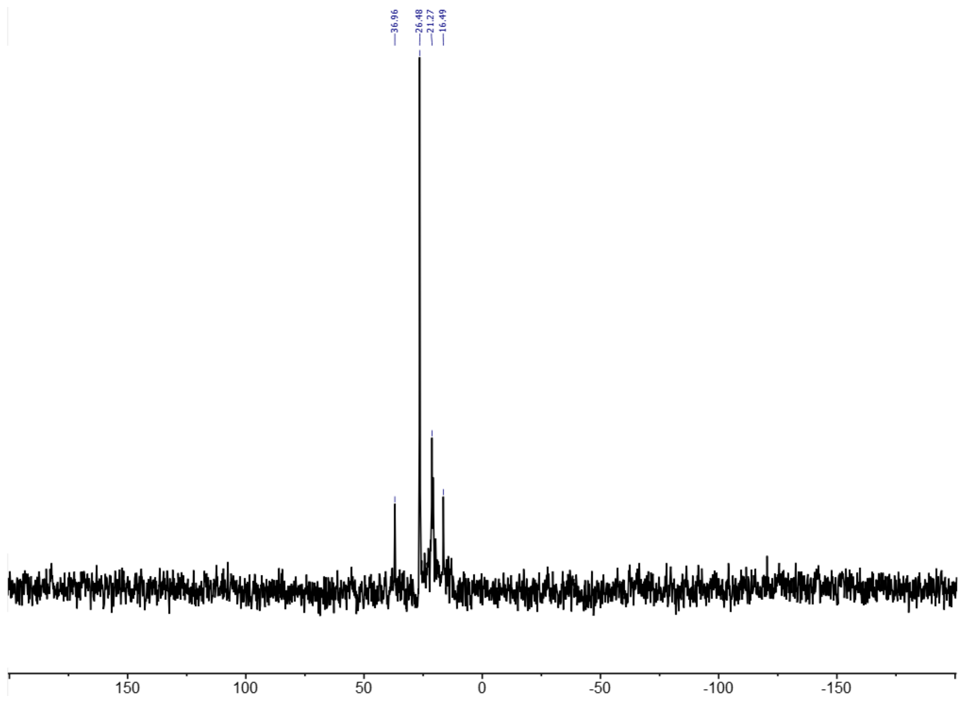

Figure S40. $^{31}P\{^{1}H\}$ NMR spectrum of the attempted reaction between [Pr(BIPM^{TMS})(I)(DME)] (**2Pr**) and PhCHO.

IR Spectroscopy

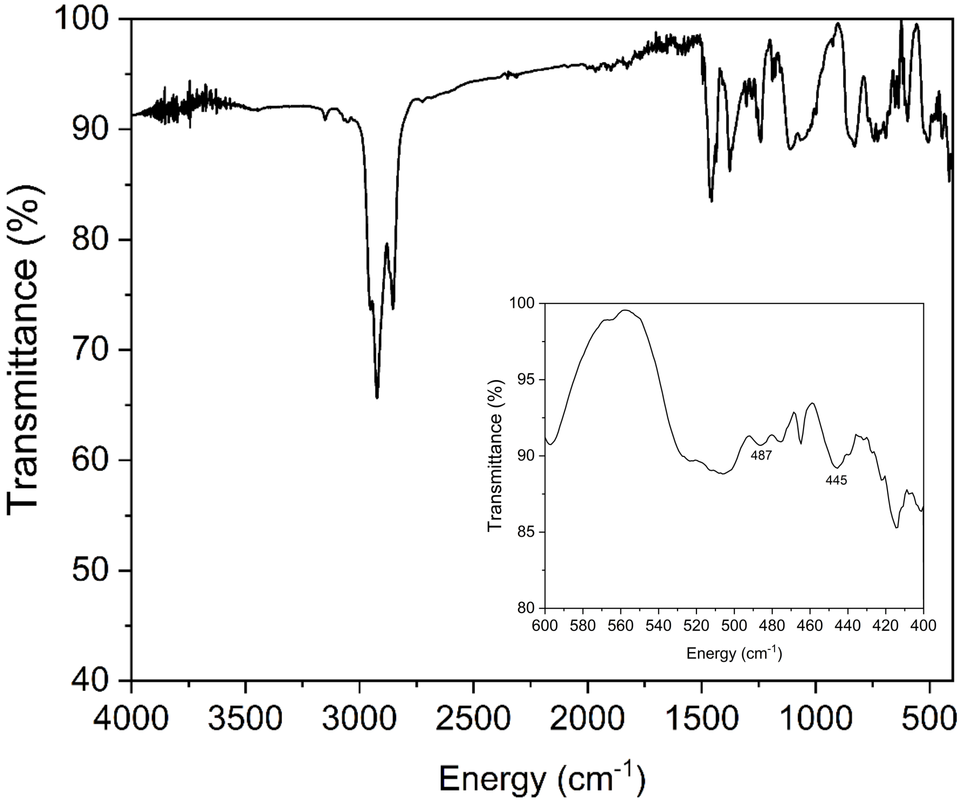

Figure S41. FT-IR spectrum of [Pu(BIPM^{TMS})(I)(DME)] (**2Pu**). Inset: 400-600 cm^{-1} region.

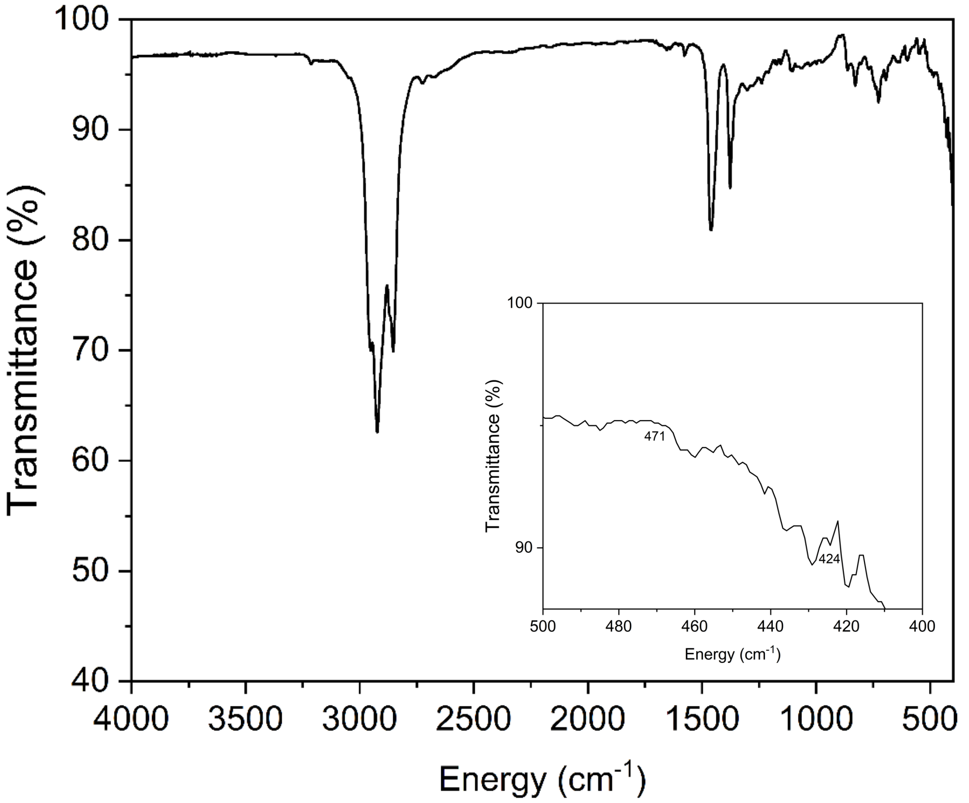

Figure S42. FT-IR spectrum of [Pu(BIPM^{TMS})(I)(I^{Me4})$_2$] (**3Pu**). Inset: 400-600 cm^{-1} region.

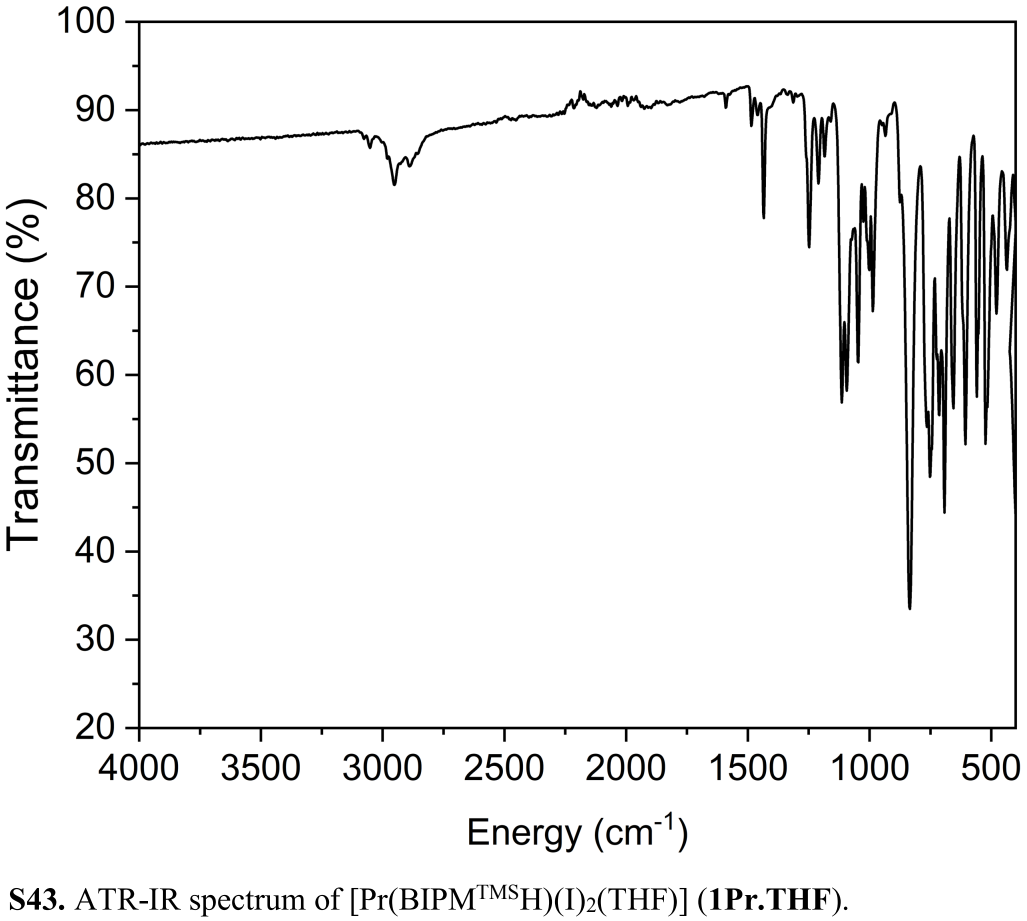

Figure S43. ATR-IR spectrum of [Pr(BIPM^{TMS}H)(I)$_2$(THF)] (**1Pr.THF**).

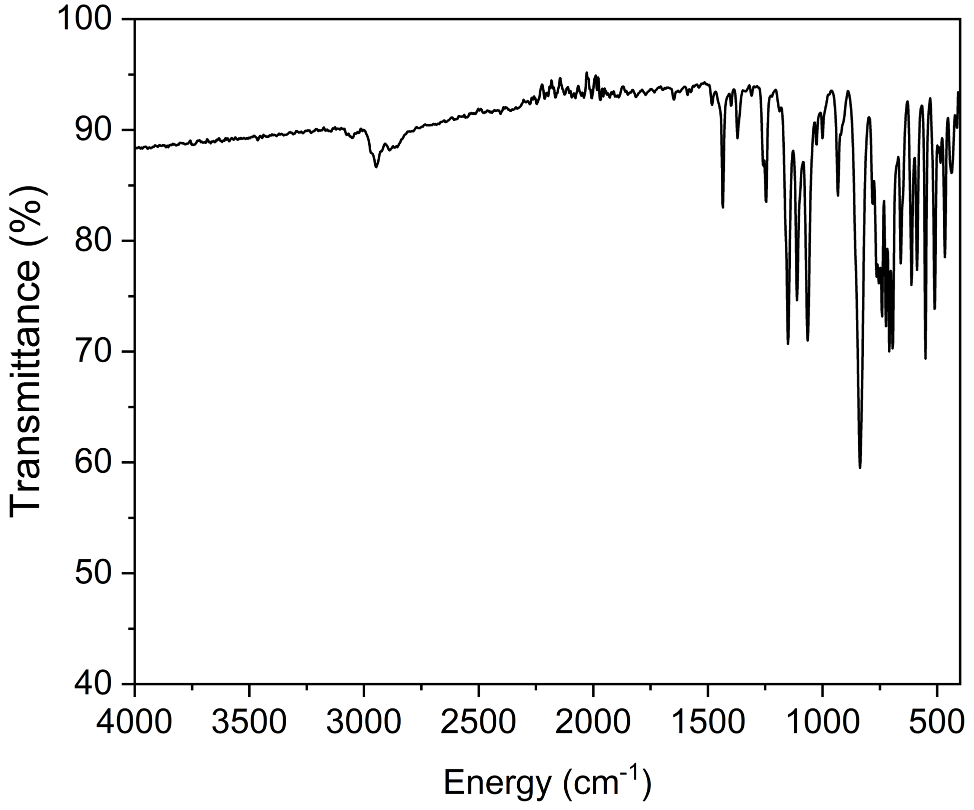

Figure S44. ATR-IR spectrum of [Pr(BIPM^{TMS}H)(I)$_2$(I^{Me4})] (**1Pr.IMe4**).

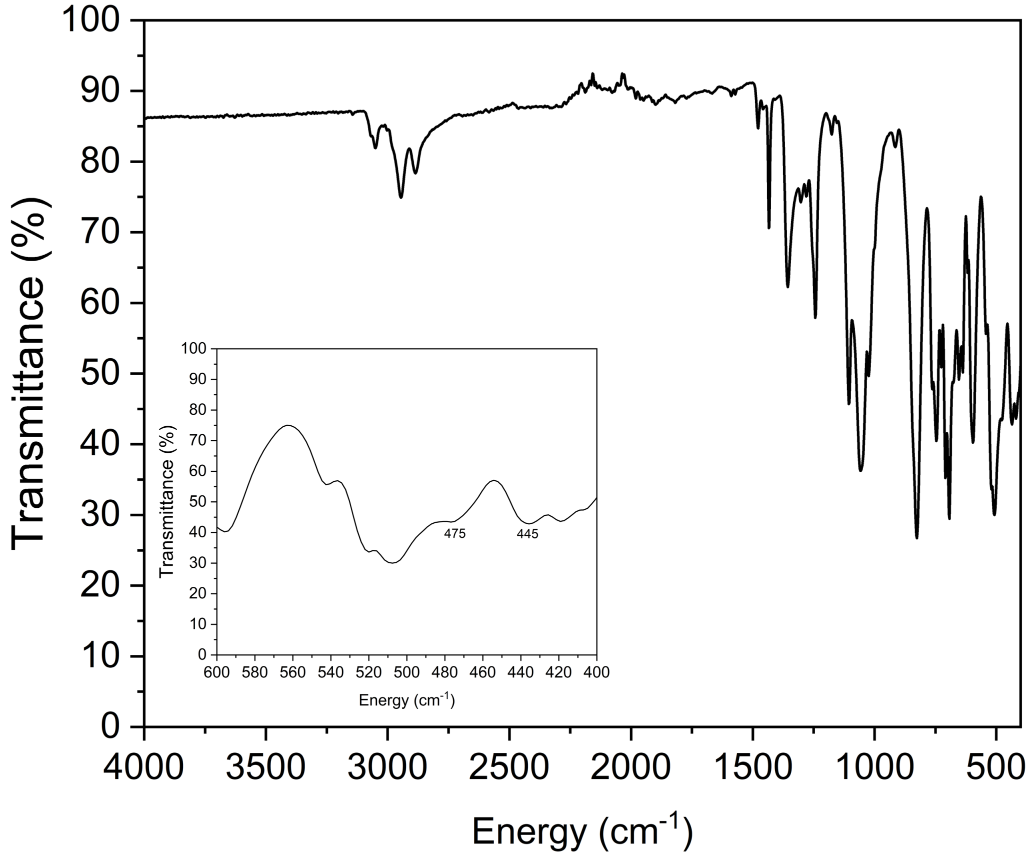

Figure S45. ATR-IR spectrum of [Pr(BIPM^{TMS})(I)(DME)] (**2Pr**). Inset: 400-600 cm^{-1} region.

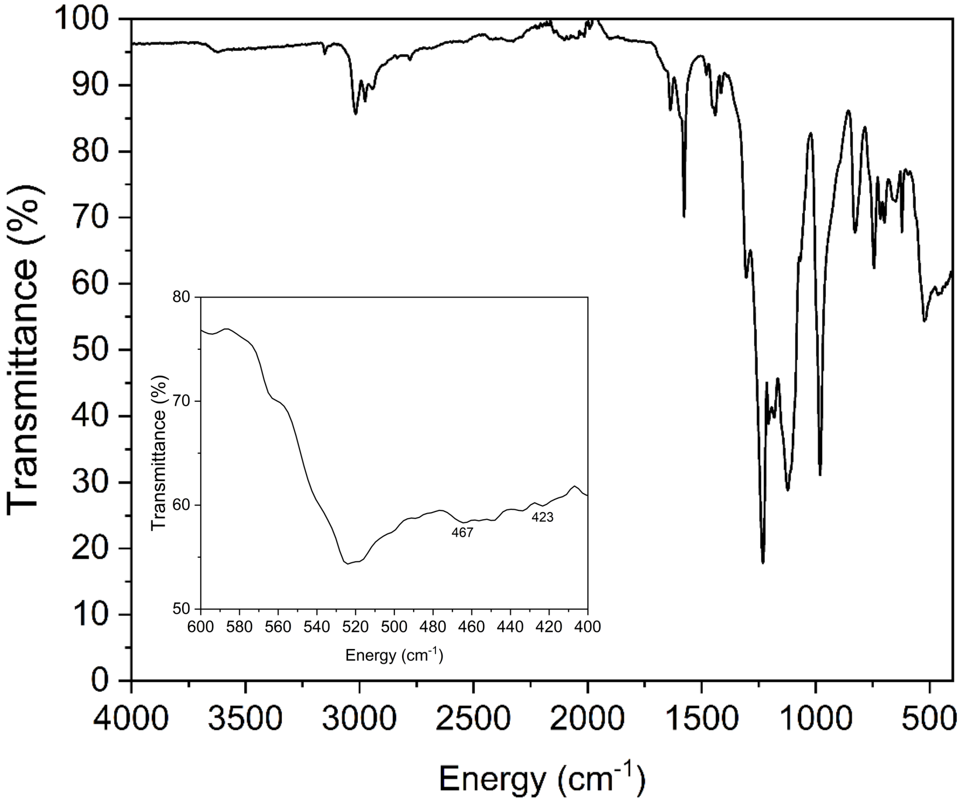

Figure S46. ATR-IR spectrum of [Pr(BIPM^{TMS})(I)(I^{Me4})$_2$] (**3Pr**). Inset: 400-600 cm^{-1} region.

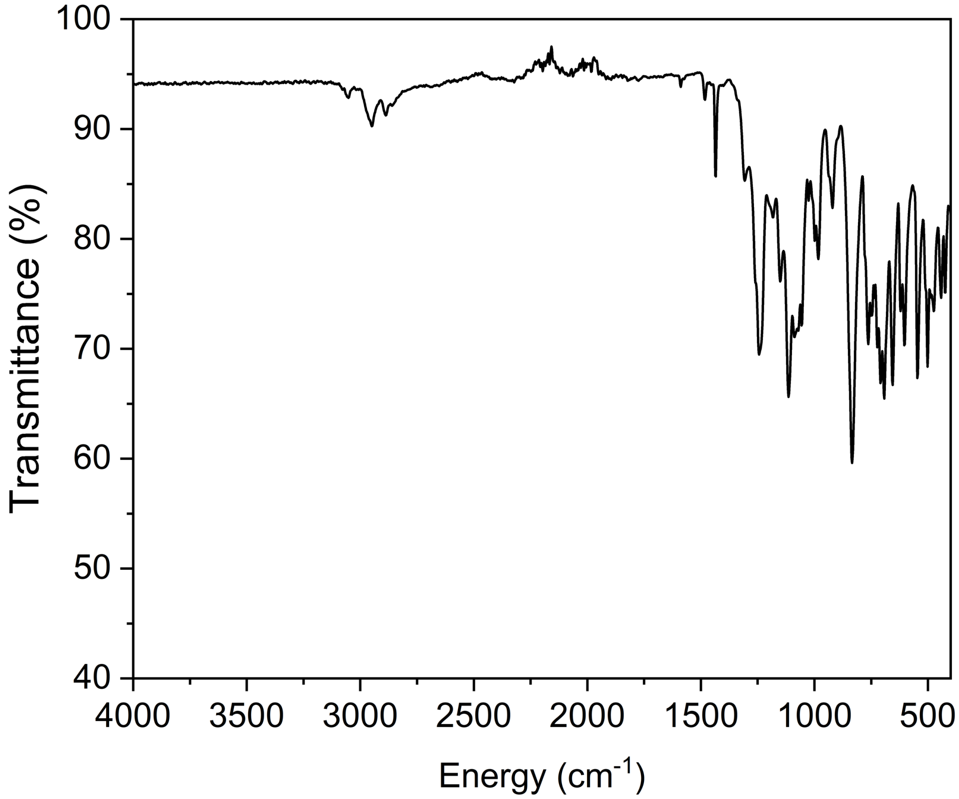

Figure S47. ATR-IR spectrum of [Sm(BIPM^{TMS}H)(I)$_2$(THF)] (**1Sm.THF**).

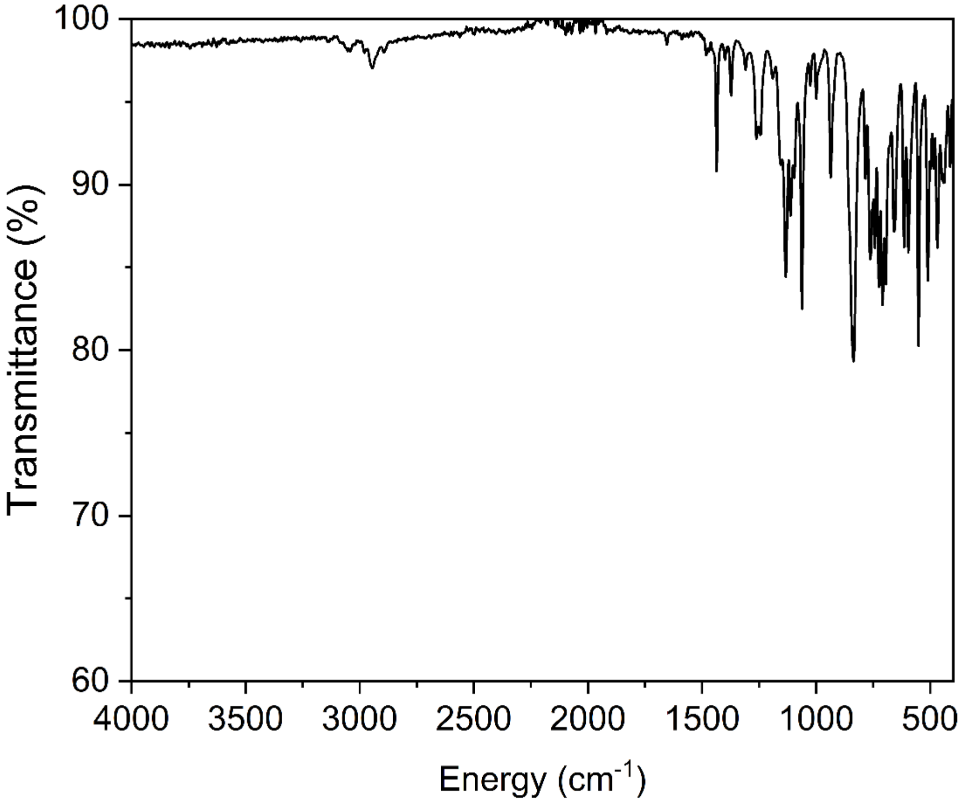

Figure S48. ATR-IR spectrum of [Sm(BIPM^{TMS}H)(I)$_2$(I^{Me4})] (**1Sm.IMe4**).

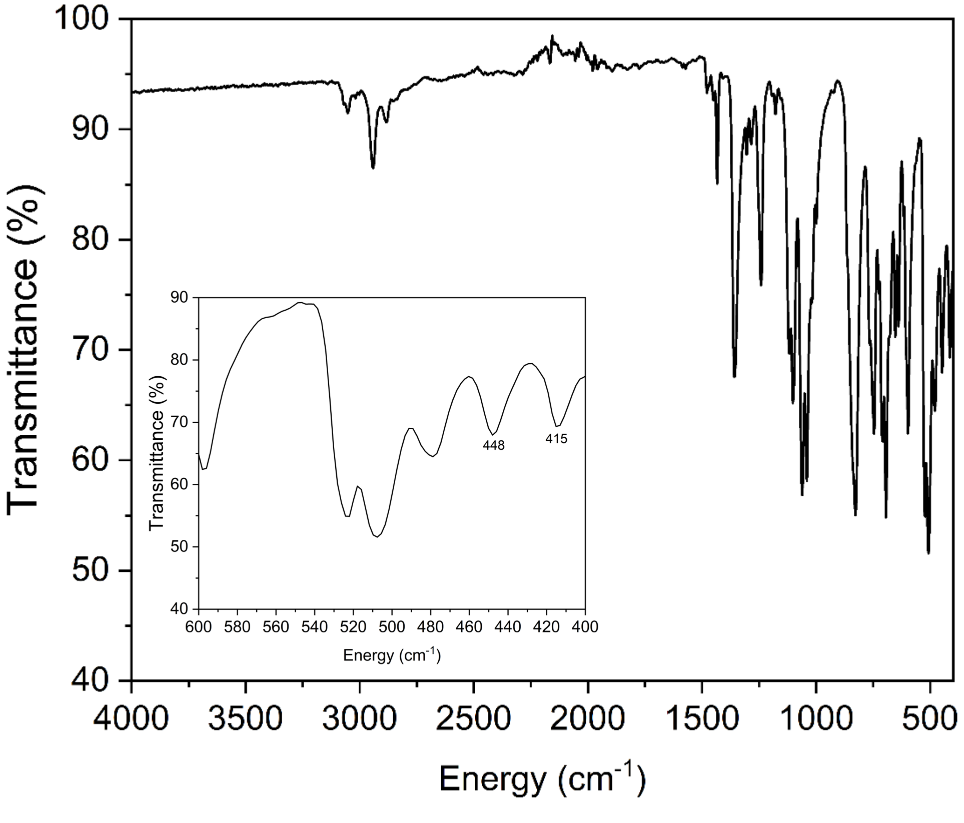

Figure S49. ATR-IR spectrum of [Sm(BIPM^{TMS})(I)(DME)] (**2Sm**). Inset: 400-600 cm^{-1} region.

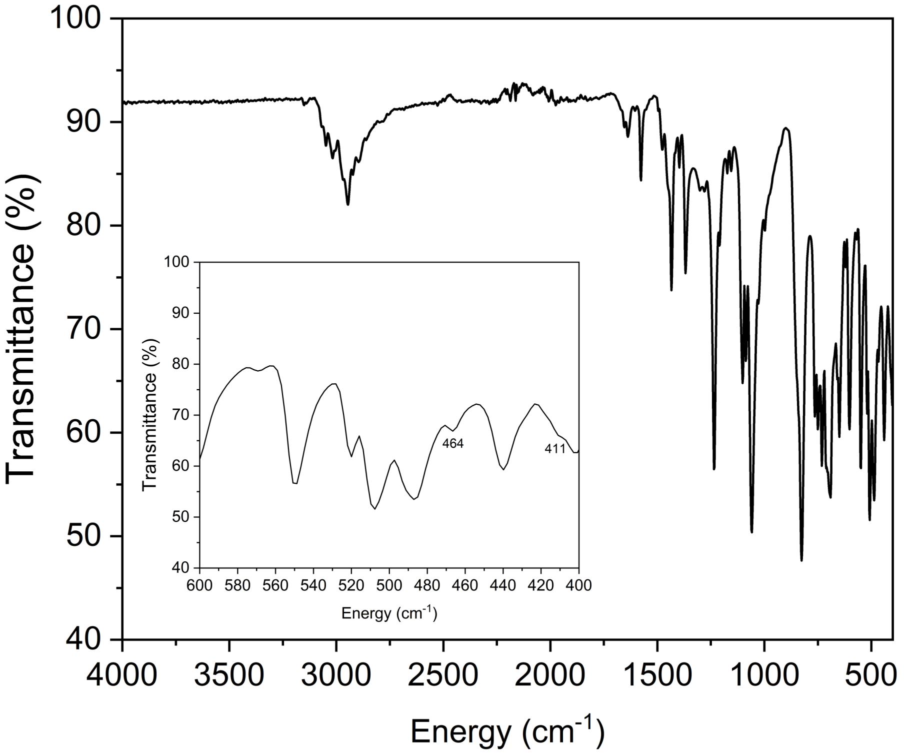

Figure S50. ATR-IR spectrum of [Sm(BIPM^{TMS})(I)(I^{Me4})$_2$] (**3Sm**). Inset: 400-600 cm^{-1} region.

UV/Vis/NIR Spectroscopy

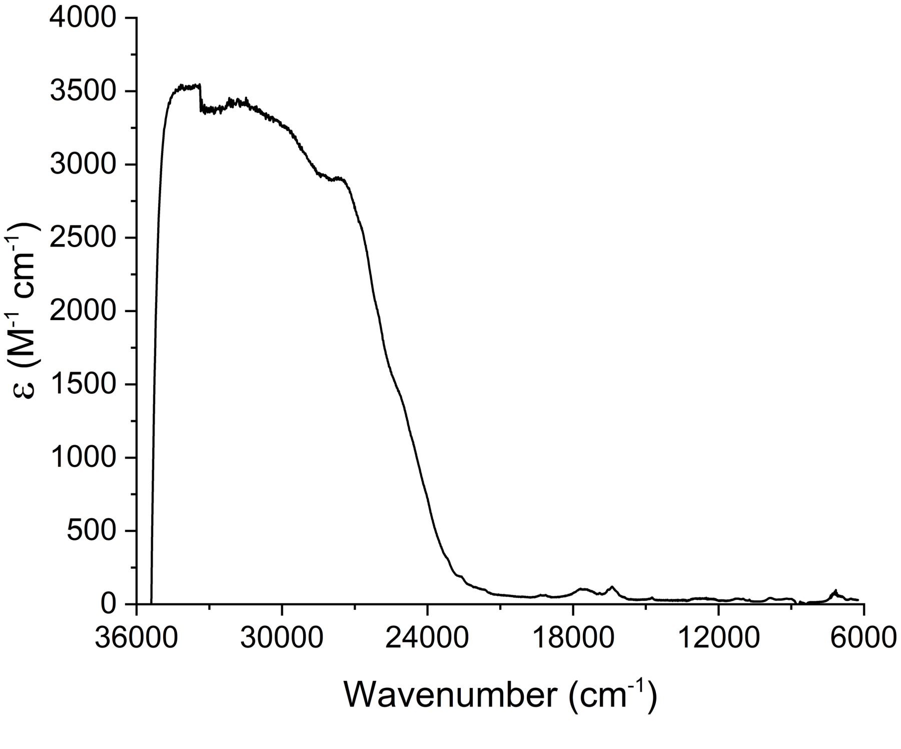

Figure S51. Solution UV/Vis/NIR spectrum of [Pu(BIPM^{TMS}H)(I)(μ-I)]$_2$ (**1Pu**) (0.50 mM in toluene) over the range 36,000 – 6,000 cm^{-1}.

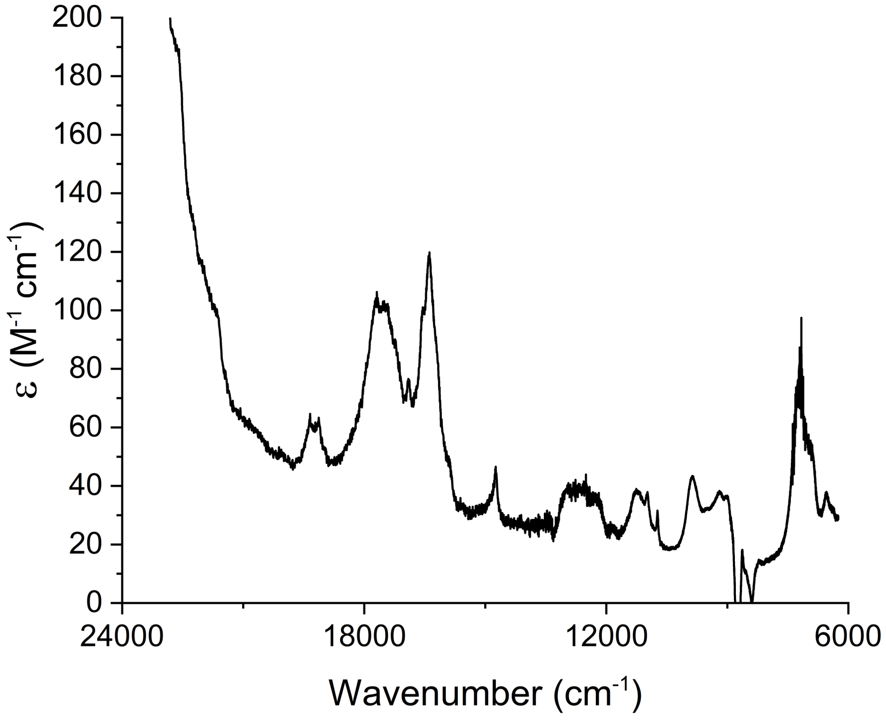

Figure S52. Solution Vis/NIR spectrum of $[Pu(BIPM^{TMS}H)(I)(\mu\text{-}I)]_2$ (**1Pu**) (0.50 mM in toluene) over the range 24,000 – 6,000 cm^{-1}.

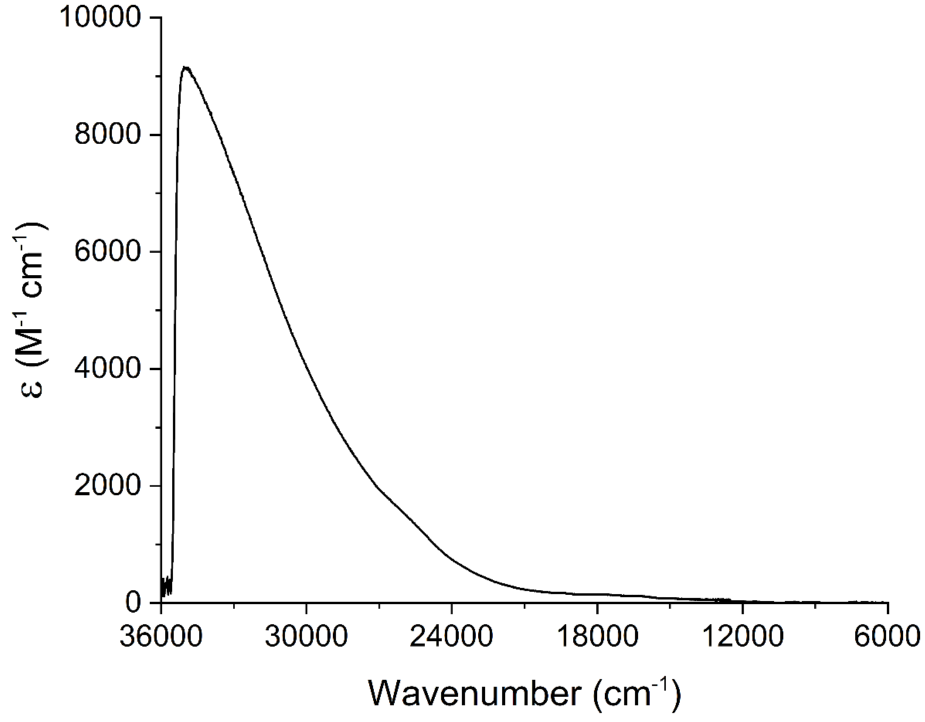

Figure S53. Solution UV/Vis/NIR spectrum of $[Pu(BIPM^{TMS})(I)(DME)]$ (**2Pu**) (0.16 mM in toluene) over the range 36,000-6,000 cm^{-1}.

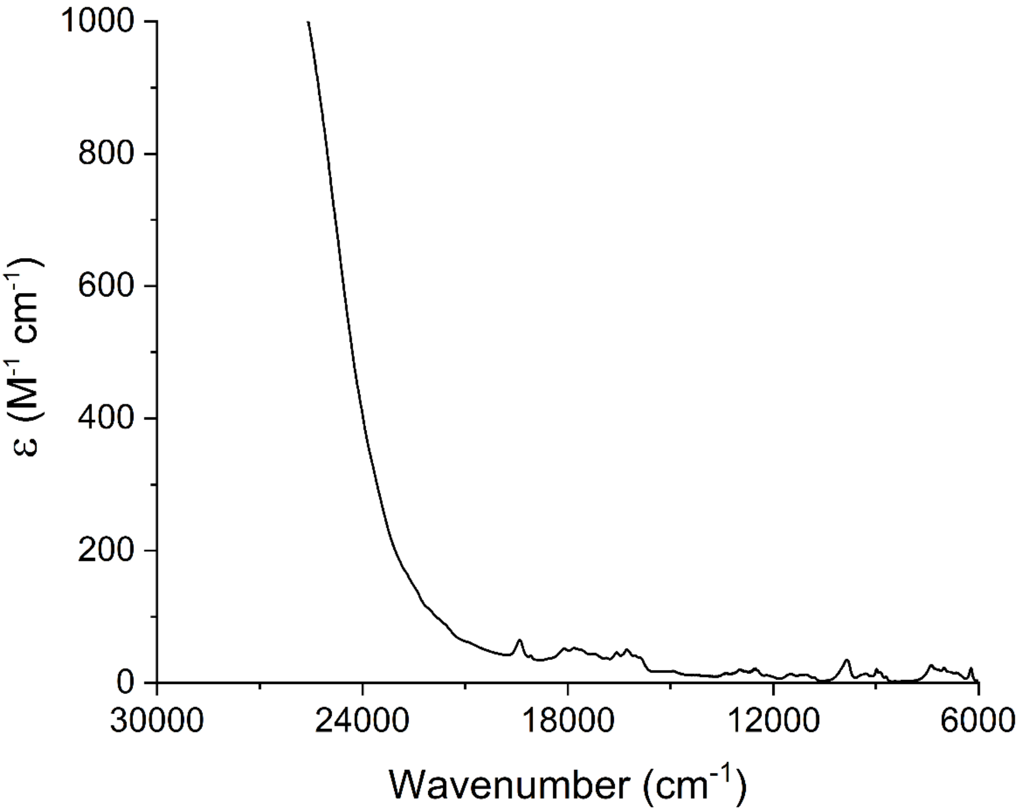

Figure S54. Solution UV/Vis/NIR spectrum of [Pu(BIPM^{TMS})(I)(DME)] (**2Pu**) (2.6 mM in toluene) over the range 30,000-6,000 cm^{-1}.

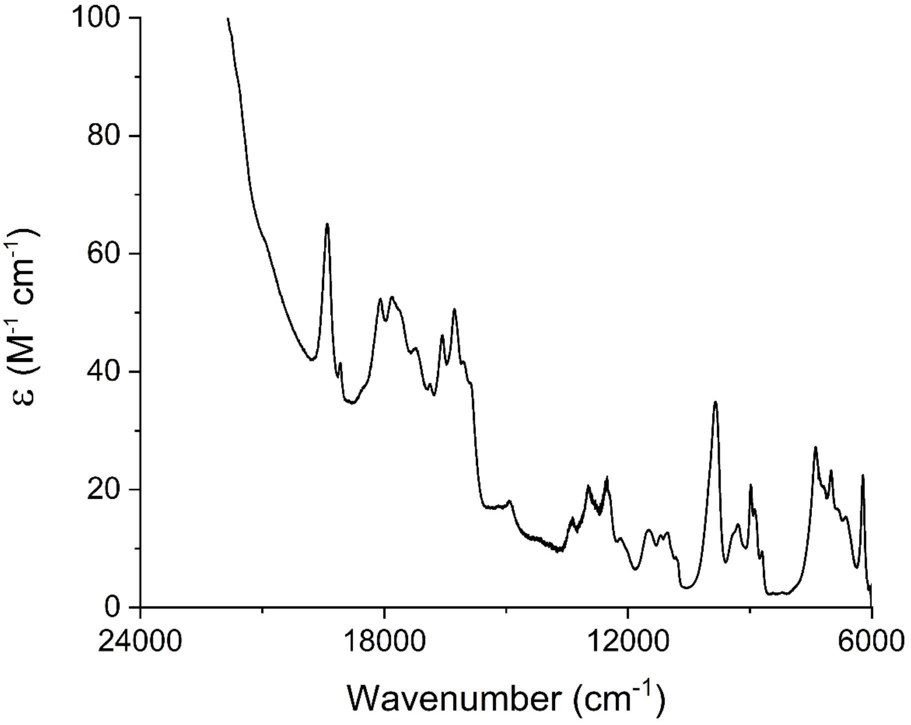

Figure S55. Zoomed-in solution UV/Vis/NIR spectrum of [Pu(BIPM^{TMS})(I)(DME)] (**2Pu**) (2.6 mM in toluene) over the range 24,000-6,000 cm^{-1}.

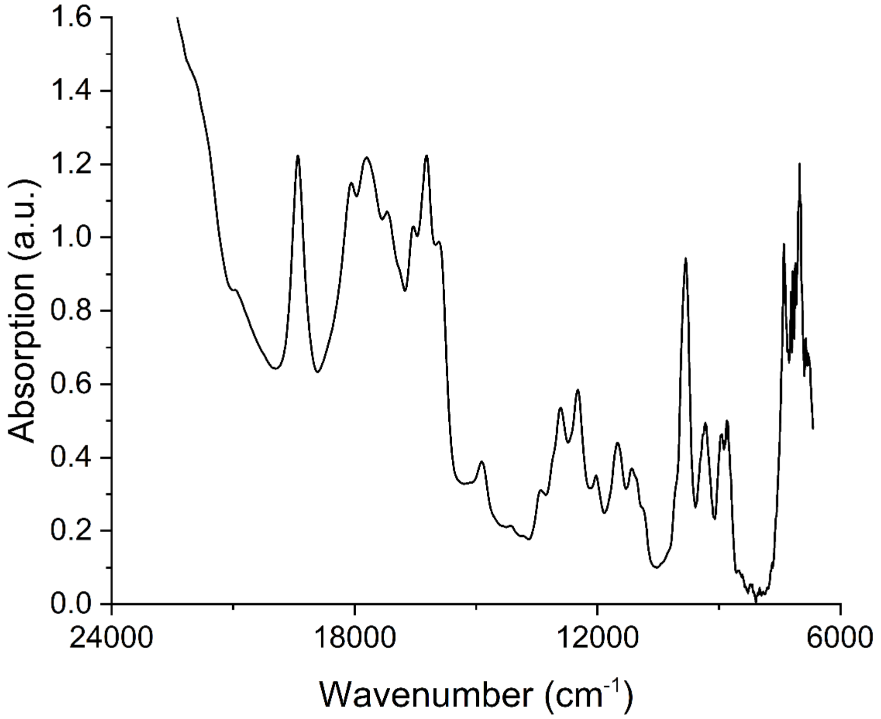

Figure S56. Solid state UV/Vis/NIR spectrum of [Pu(BIPM^{TMS})(I)(DME)] (**2Pu**) over the range 24,000-6,000 cm^{-1}.

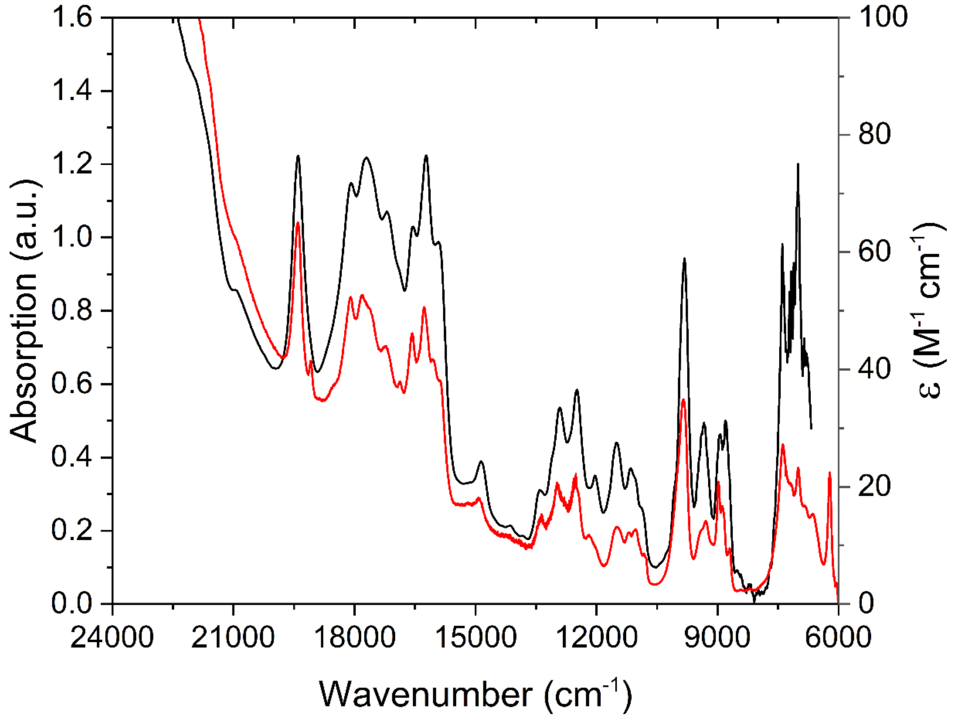

Figure S57. Comparative UV/Vis/NIR spectra of [Pu(BIPM^{TMS})(I)(DME)] (**2Pu**) in solution (2.6 mM in toluene; right y-axis, red) and in the solid-state (left y-axis, black) zoomed-in over the range 24,000-6,000 cm^{-1}.

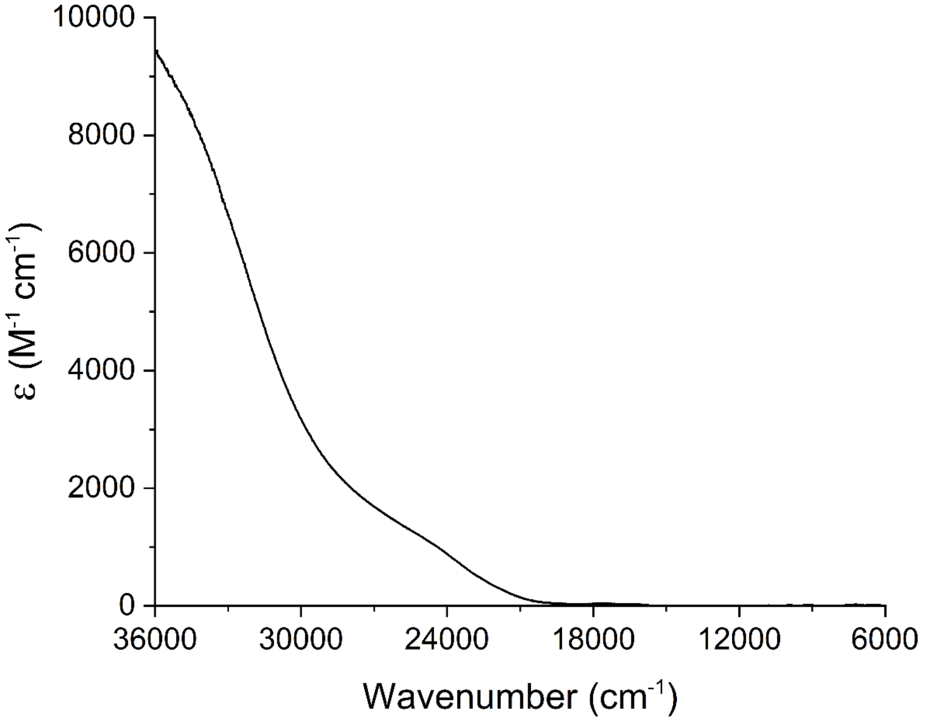

Figure S58. Solution UV/Vis/NIR spectrum of [Pu(BIPM^{TMS})(I)(I^{Me4})$_2$] (**3Pu**) (0.2 mM in THF) over the range 35,000-6,000 cm^{-1}.

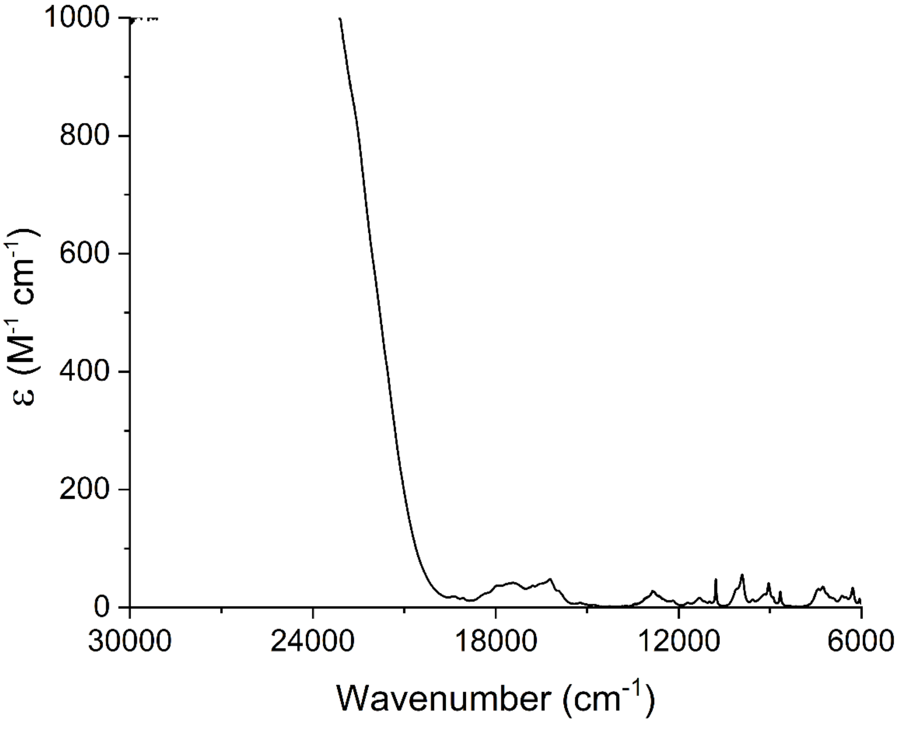

Figure S59. Solution UV/Vis/NIR spectrum of [Pu(BIPM^{TMS})(I)(I^{Me4})$_2$] (**3Pu**) (3.0 mM in THF) over the range 30,000-6,000 cm^{-1}.

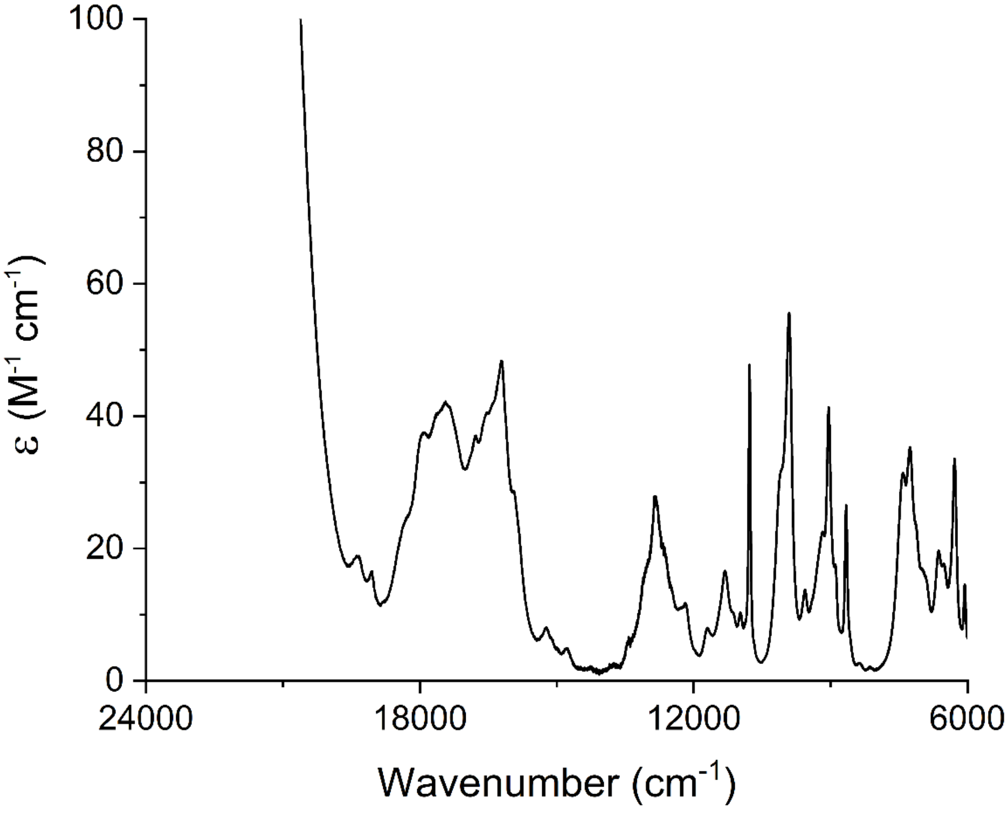

Figure S60. Zoomed-in solution UV/Vis/NIR spectrum of [Pu(BIPM^{TMS})(I)(I^{Me4})$_2$] (**3Pu**) (3.0 mM in THF) over the range 24,000-6,000 cm^{-1}.

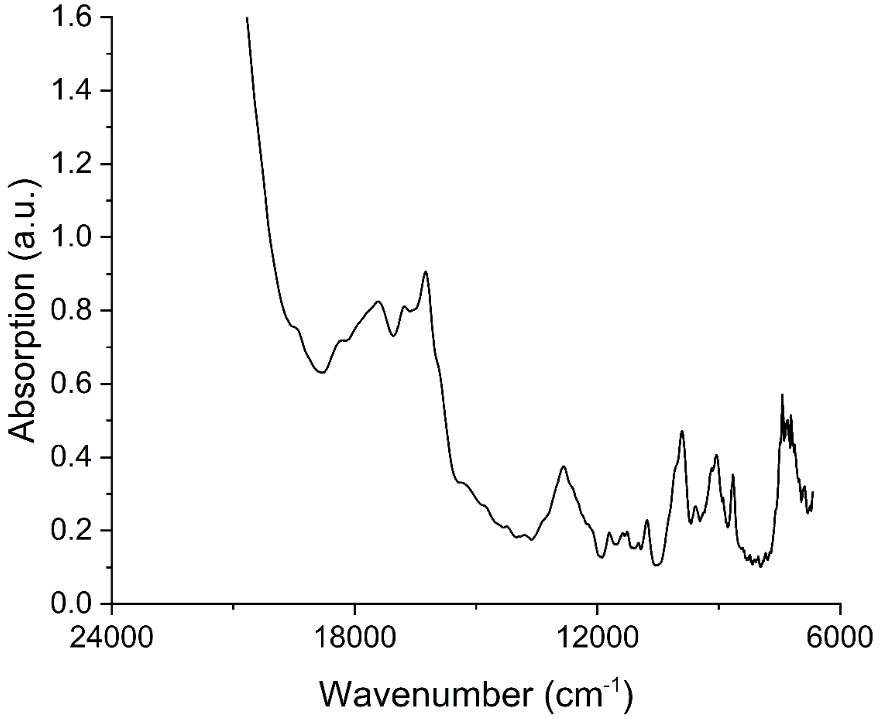

Figure S61. Solid state UV/Vis/NIR spectrum of [Pu(BIPM^{TMS})(I)(I^{Me4})$_2$] (**3Pu**) over the range 24,000-6,000 cm^{-1}.

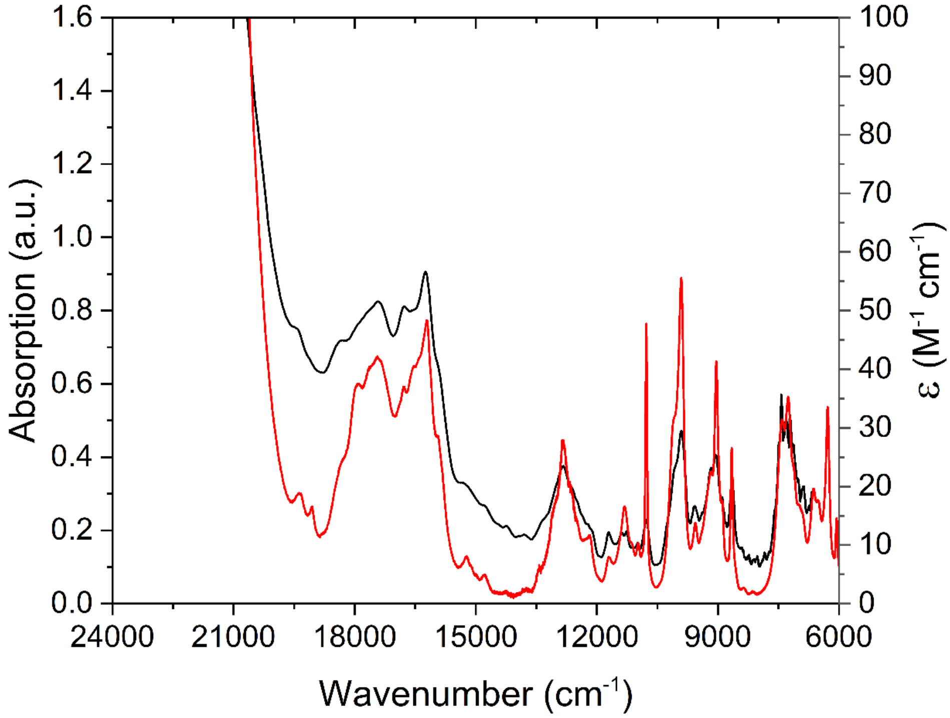

Figure S62. Comparative UV/Vis/NIR spectra of [Pu(BIPM^{TMS})(I)(I^{Me4})$_2$] (**3Pu**) in solution (3.0 mM in THF; right y-axis, red) and in the solid-state (left y-axis, black) zoomed-in over the range 24,000-6,000 cm^{-1}.

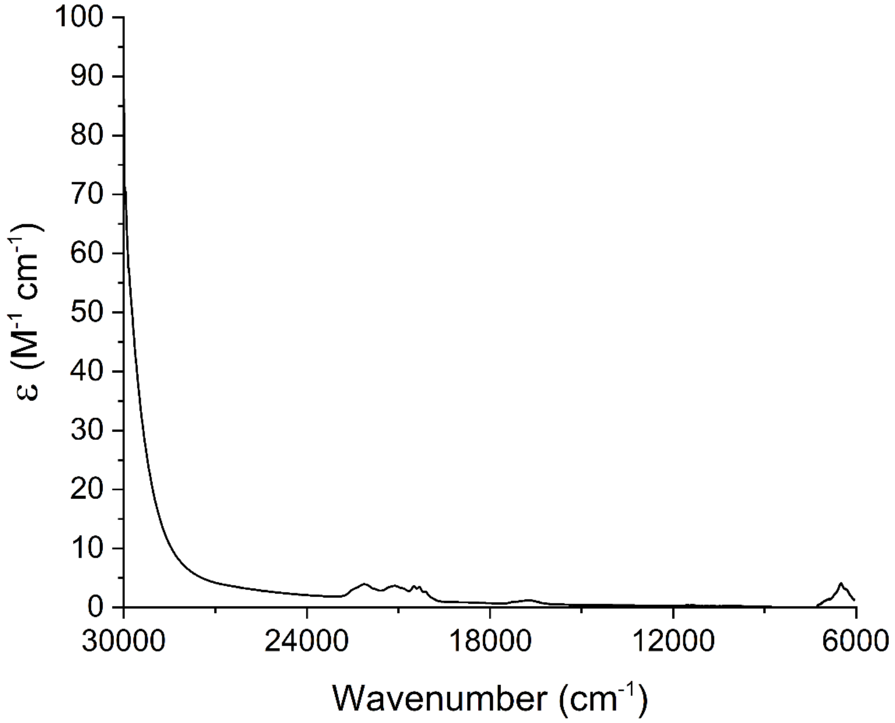

Figure S63. Solution UV/Vis/NIR spectrum of [Pr(BIPM^{TMS}H)(I)$_2$(THF)] (**1Pr.THF**) (54 mM in THF) over the range 30,000-6,000 cm^{-1}.

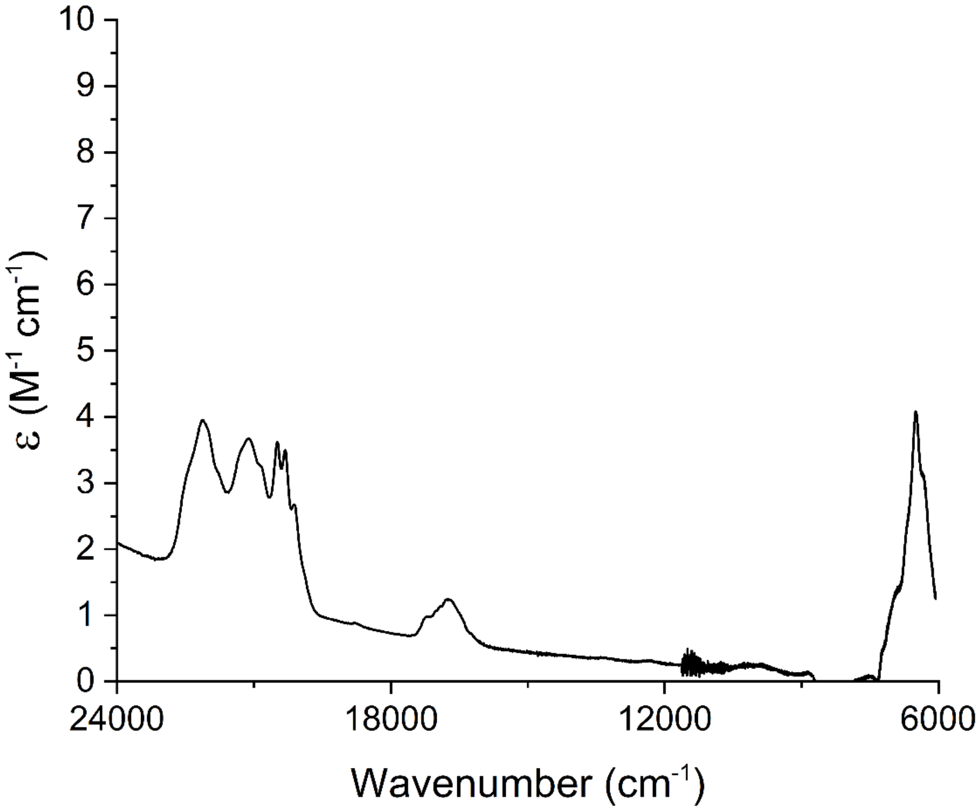

Figure S64. Zoomed-in solution UV/Vis/NIR spectrum of [Pr(BIPM^{TMS}H)(I)$_2$(THF)] (**1Pr.THF**) (54 mM in THF) over the range 24,000-6,000 cm^{-1}.

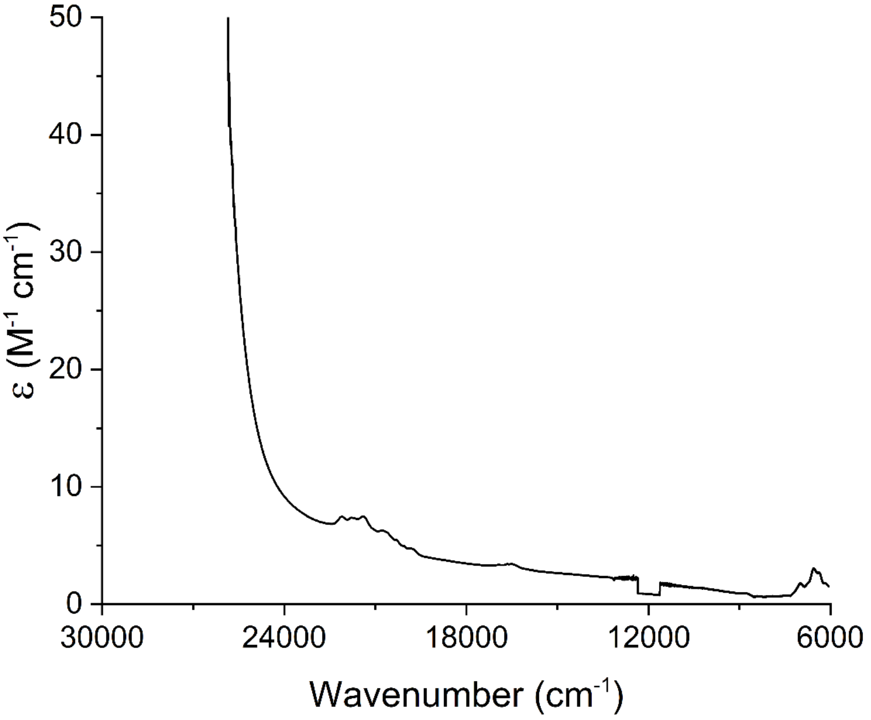

Figure S65. Solution UV/Vis/NIR spectrum of [Pr(BIPM^{TMS}H)(I)$_2$(I^{Me4})] (**1Pr.IMe4**) (44 mM in THF) over the range 30,000-6,000 cm^{-1}.

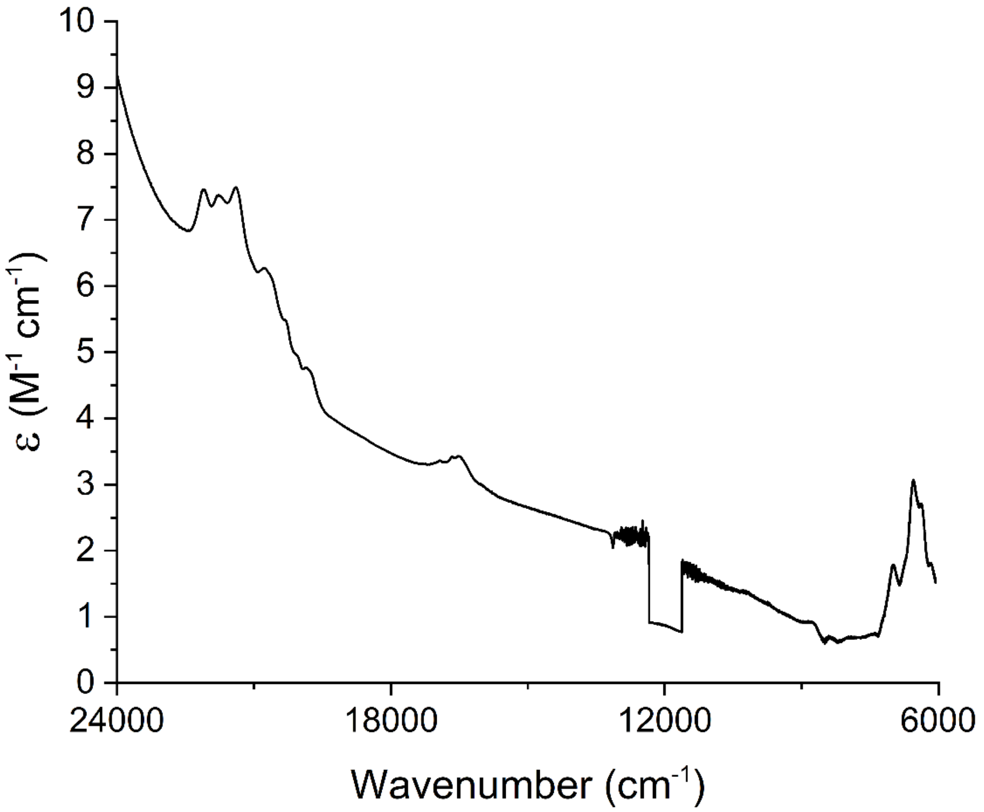

Figure S66. Zoomed-in solution UV/Vis/NIR spectrum of [Pr(BIPM^{TMS}H)(I)$_2$(I^{Me4})] (**1Pr.IMe4**) (44 mM in THF) over the range 24,000-6,000 cm^{-1}.

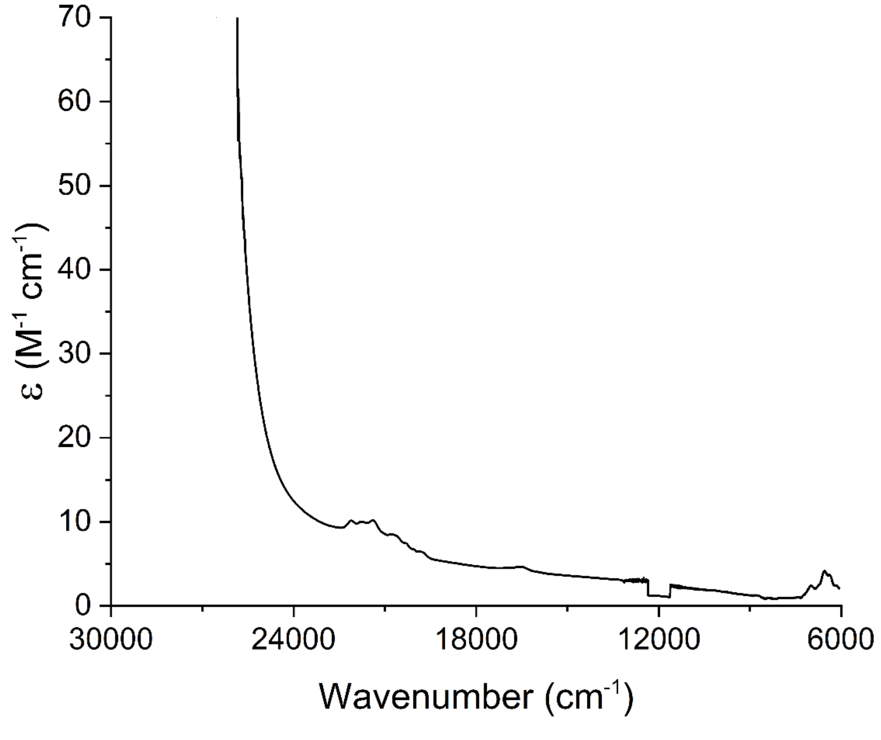

Figure S67. Solution UV/Vis/NIR spectrum of [Pr(BIPM^{TMS})(I)(DME)] (**2Pr**) (64 mM in THF) over the range 30,000-6,000 cm^{-1}.

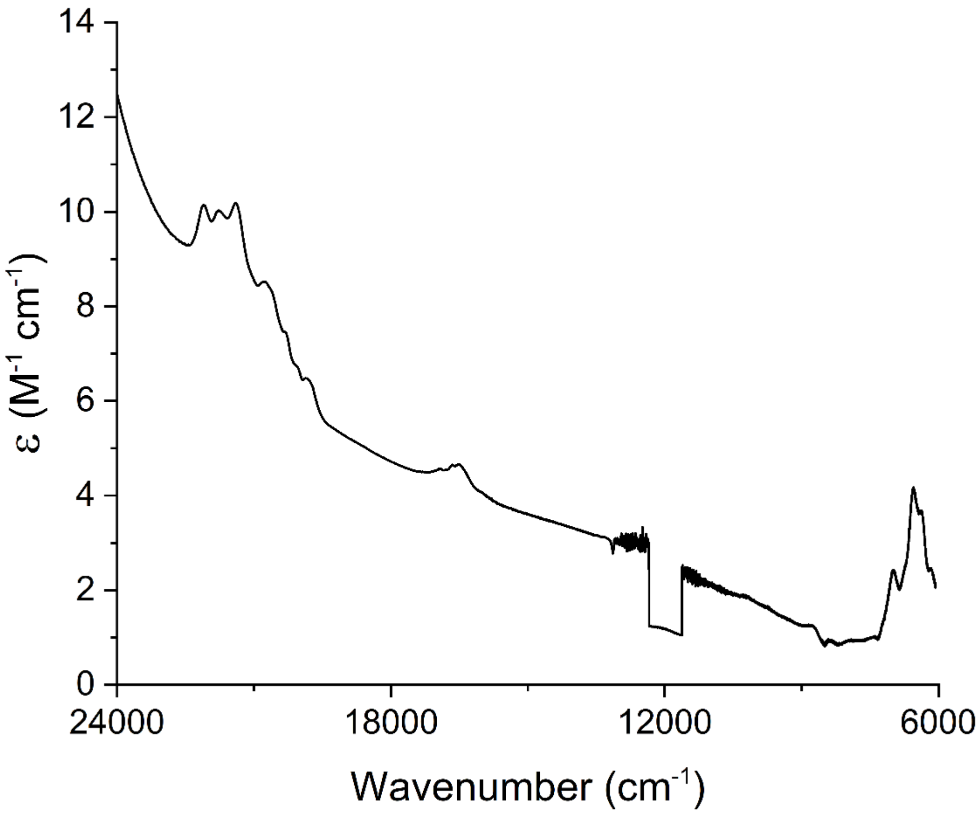

Figure S68. Zoomed-in solution UV/Vis/NIR spectrum of [Pr(BIPM^{TMS})(I)(DME)] (**2Pr**) (64 mM in THF) over the range 24,000-6,000 cm^{-1}.

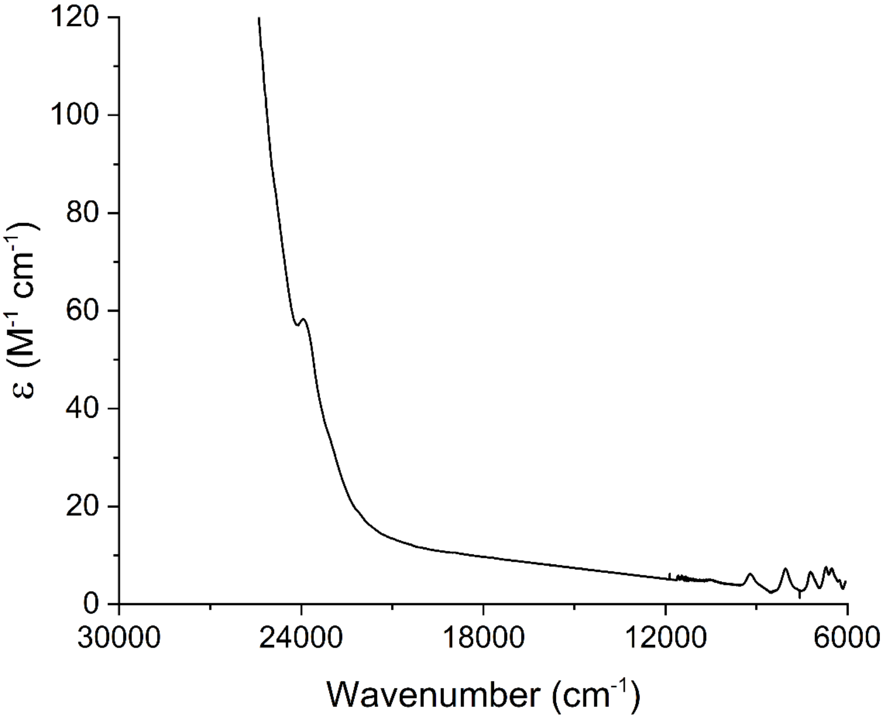

Figure S69. Solution UV/Vis/NIR spectrum of [Sm(BIPM^{TMS}H)(I)$_2$(THF)] (**1Sm.THF**) (25 mM in THF) over the range 30,000-6,000 cm^{-1}.

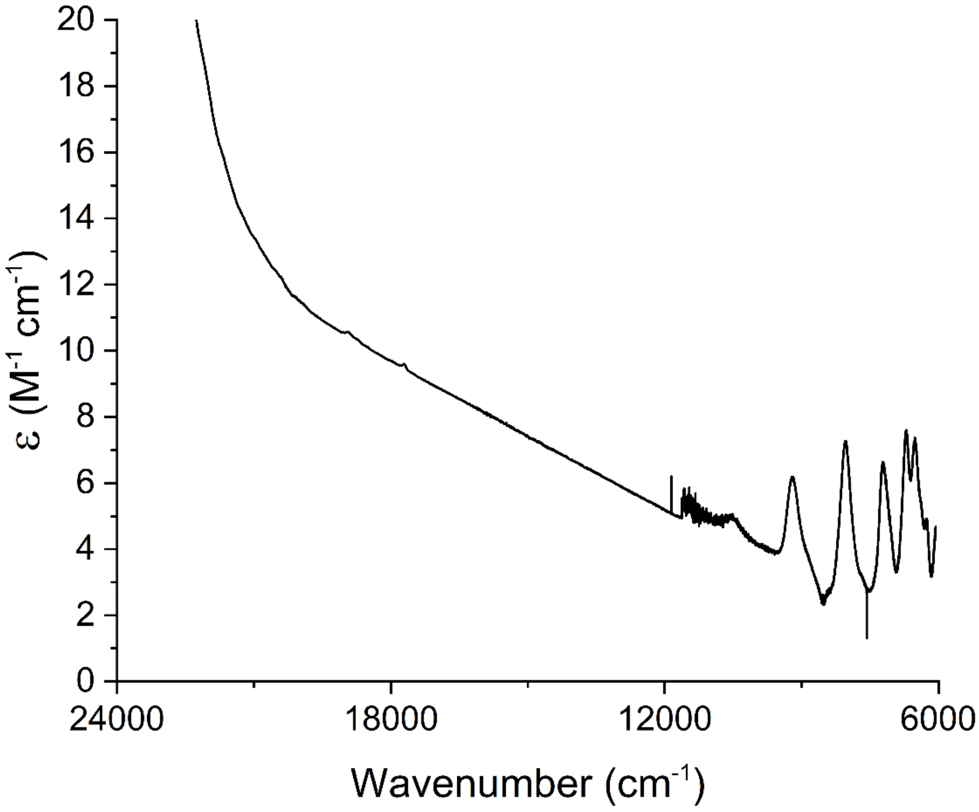

Figure S70. Zoomed-in solution UV/Vis/NIR spectrum of [Sm(BIPM^{TMS}H)(I)$_2$(THF)] (**1Sm.THF**) (25 mM in THF) over the range 24,000-6,000 cm^{-1}.

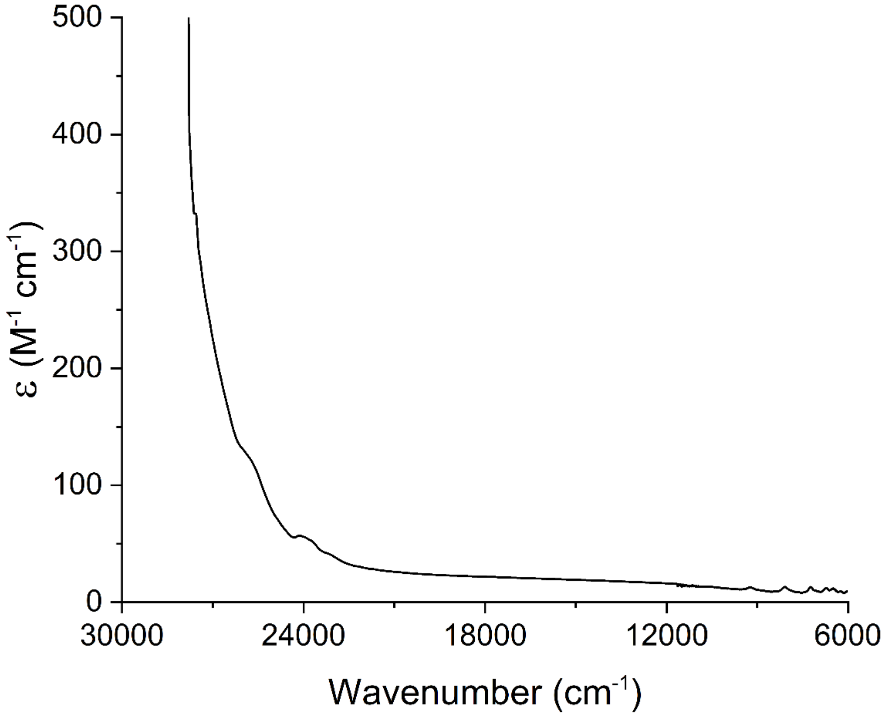

Figure S71. Solution UV/Vis/NIR spectrum of [Sm(BIPM^{TMS}H)(I)$_2$(I^{Me4})] (**1Sm.IMe4**) (13 mM in THF) over the range 30,000-25,000 cm^{-1}.

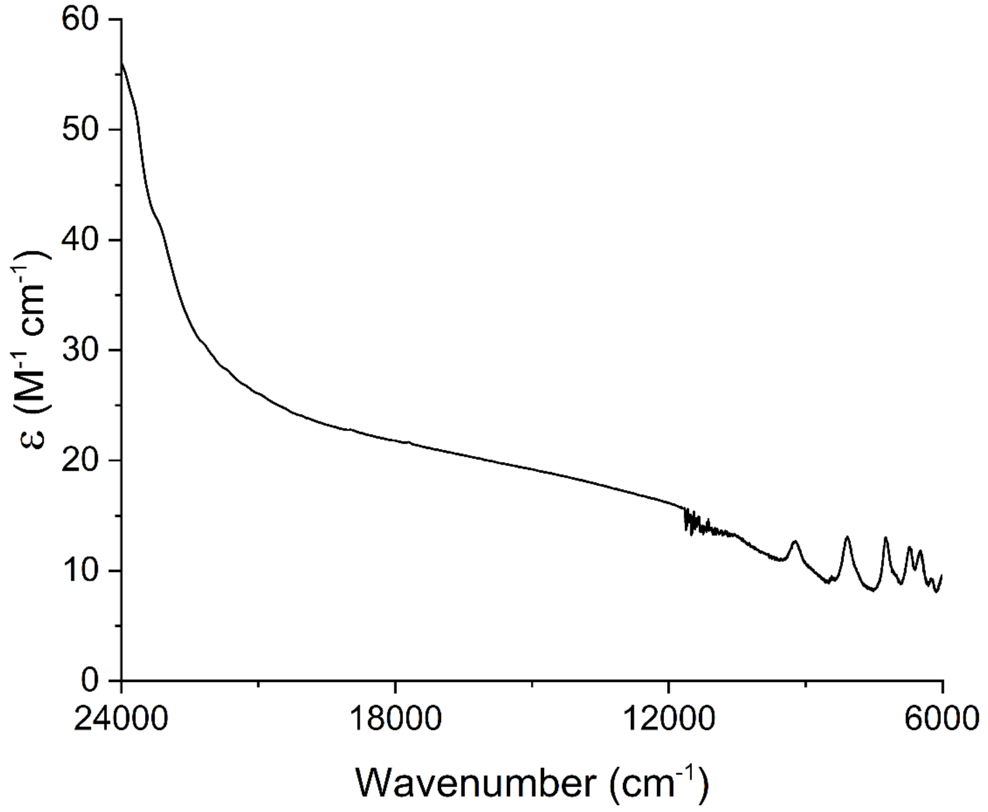

Figure S72. Zoomed-in solution U/-Vis/NIR spectrum of [Sm(BIPM^{TMS}H)(I)$_2$(I^{Me4})] (**1Sm.IMe4**) (13 mM in THF) over the range 24,000-6,000 cm^{-1}.

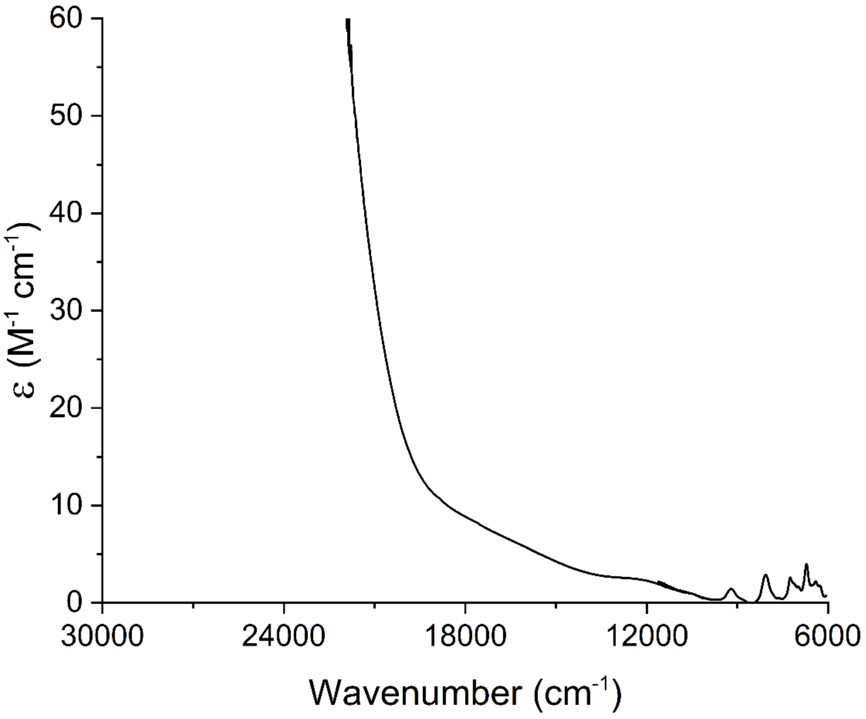

Figure S73. Solution UV/Vis/NIR spectrum of [Sm(BIPM^{TMS})(I)(DME)] (**2Sm**) (61 mM in THF) over the range 30,000-6,000 cm^{-1}.

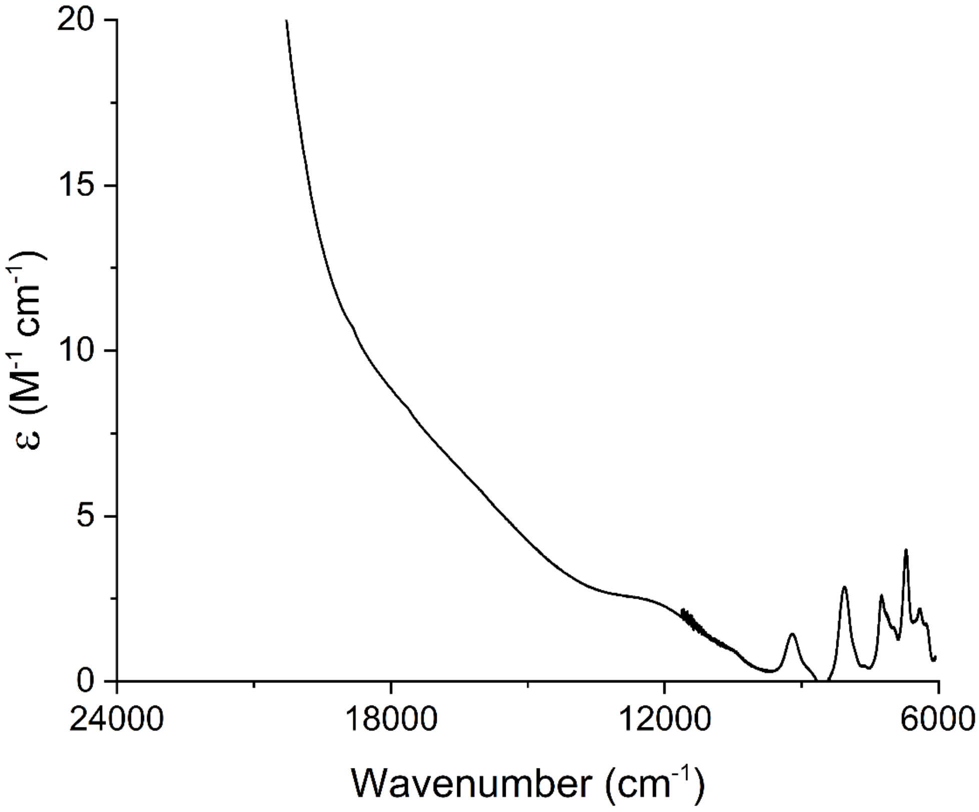

Figure S74. Zoomed-in solution UV/Vis/NIR spectrum of [Sm(BIPM^{TMS})(I)(DME)] (**2Sm**) (61 mM in THF) over the range 25,000-6,000 cm^{-1}.

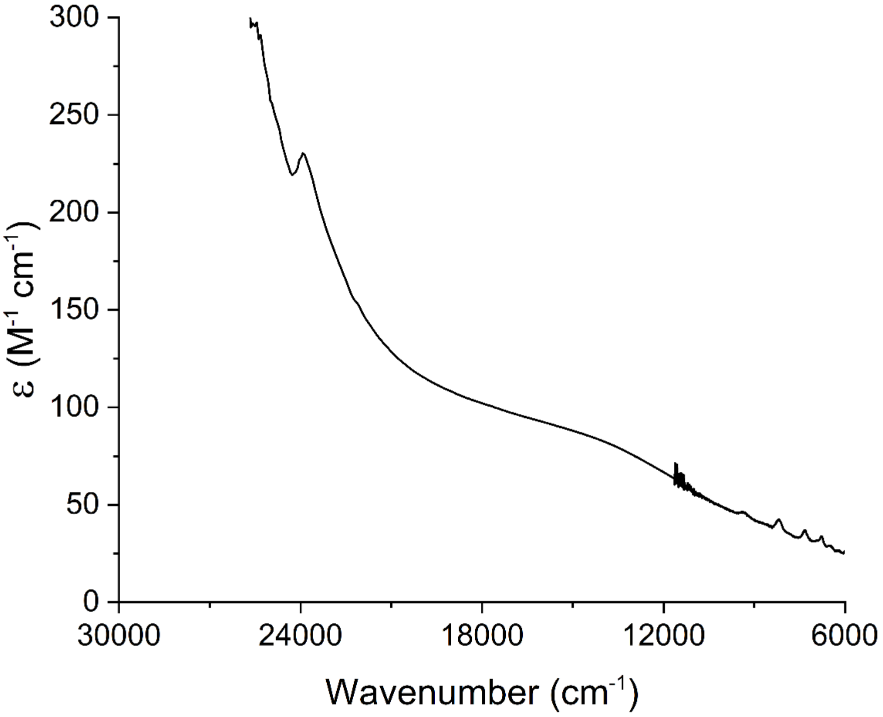

Figure S75. Solution UV/Vis/NIR spectrum of [Sm(BIPM^{TMS})(I)(I^{Me4})$_2$] (**3Sm**) (14mM in THF) over the range 30,000-6,000 cm^{-1}.

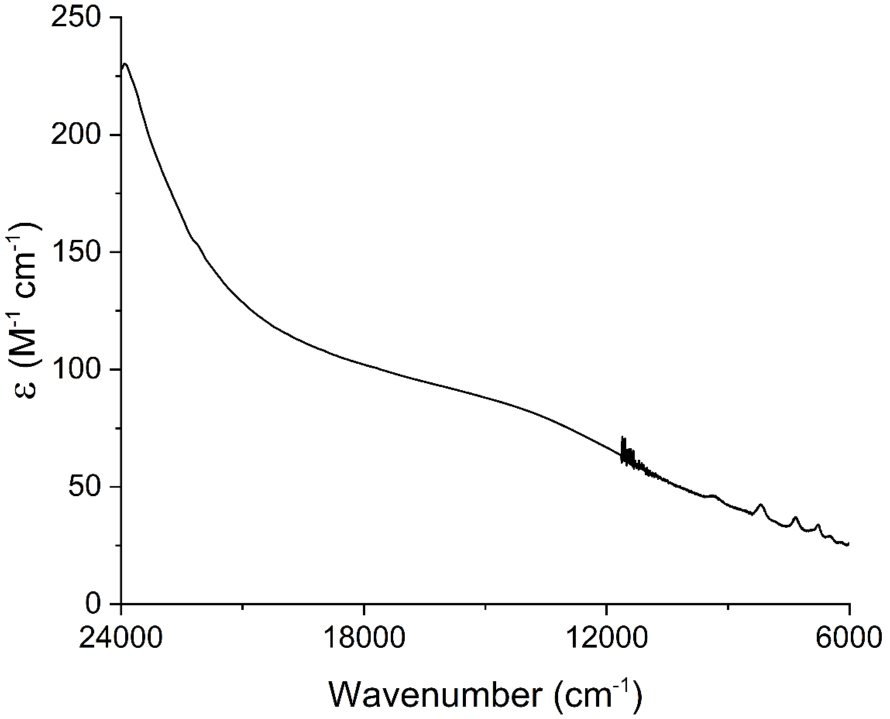

Figure S76. Zoomed-in solution UV/Vis/NIR spectrum of [Sm(BIPM^{TMS})(I)(I^{Me4})$_2$] (**3Sm**) (14 mM in THF) over the range 24,000-6,000 cm^{-1}.

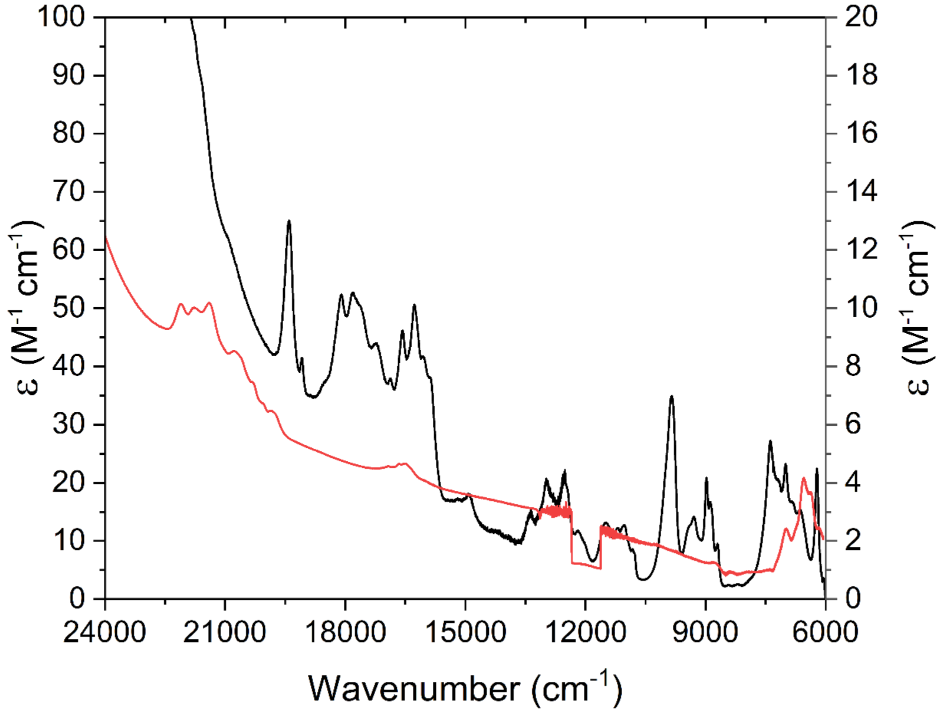

Figure S77. Comparative UV/Vis/NIR spectra of [Pr(BIPM^{TMS})(I)(DME)] (**2Pr**) (64 mM in THF; right y-axis, red) and [Pu(BIPM^{TMS})(I)(DME)] (**2Pu**) (2.6 mM in toluene; left y-axis, black) zoomed-in over the range 24,000-6,000 cm^{-1}.

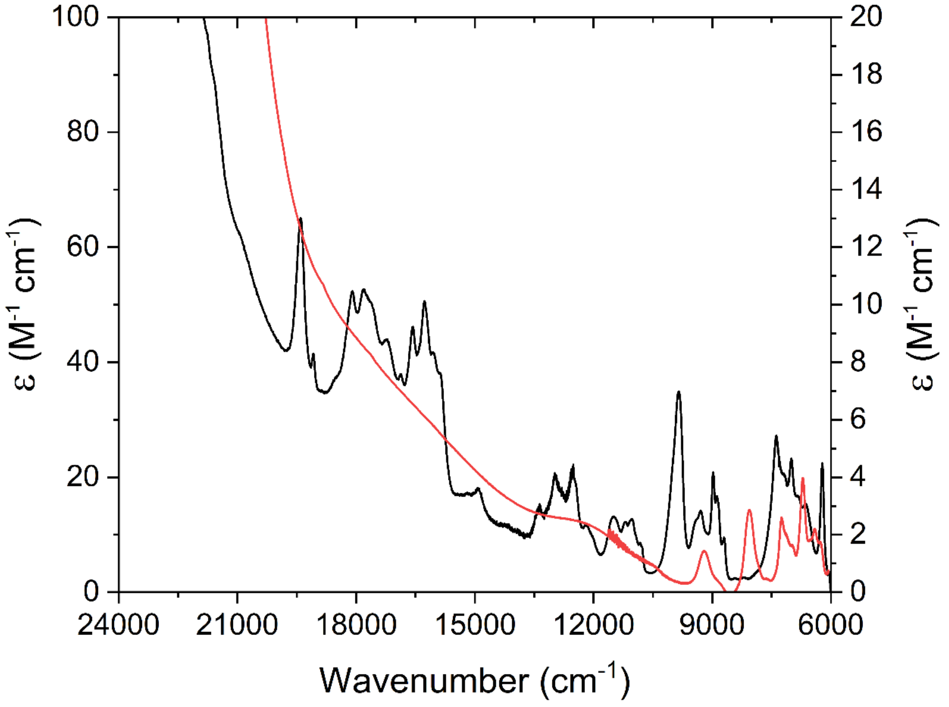

Figure S78. Comparative UV/Vis/NIR spectra of [Sm(BIPM^{TMS})(I)(DME)] (**2Sm**) (64 mM in THF; right y-axis, red) and [Pu(BIPM^{TMS})(I)(DME)] (**2Pu**) (2.6 mM in toluene; left y-axis, black) zoomed-in over the range 24,000-6,000 cm^{-1}.

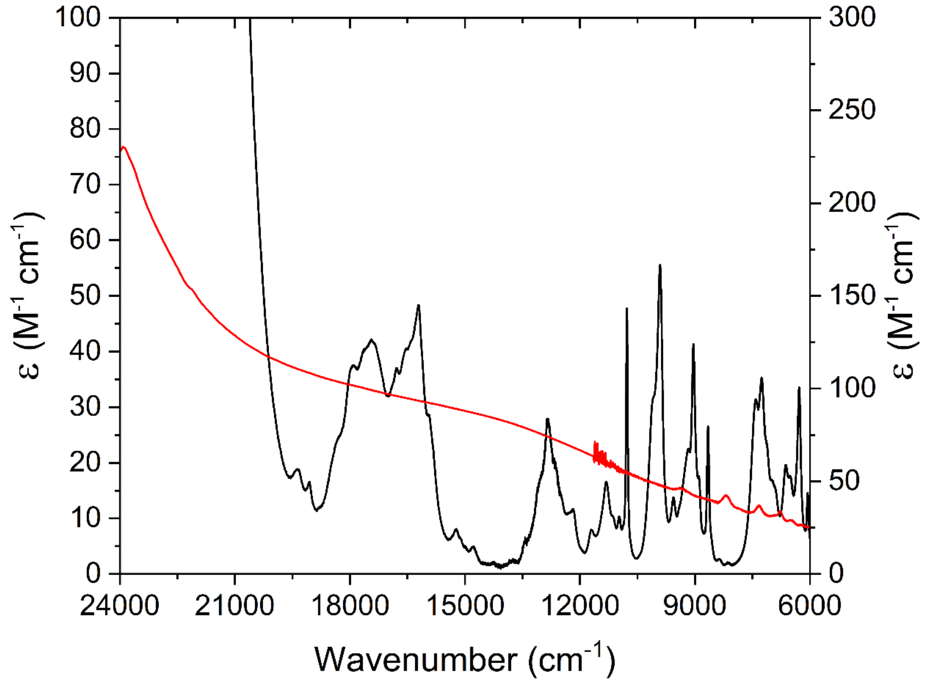

Figure S79. Comparative UV/Vis/NIR spectra of [Sm(BIPM^{TMS})(I)(I^{Me4})$_2$] (**3Sm**) (64 mM in THF; right y-axis, red) and [Pu(BIPM^{TMS})(I)(I^{Me4})$_2$] (**3Pu**) (3.0 mM in THF; left y-axis, black) zoomed-in over the range 24,000-6,000 cm^{-1}.

DFT and NBO representations

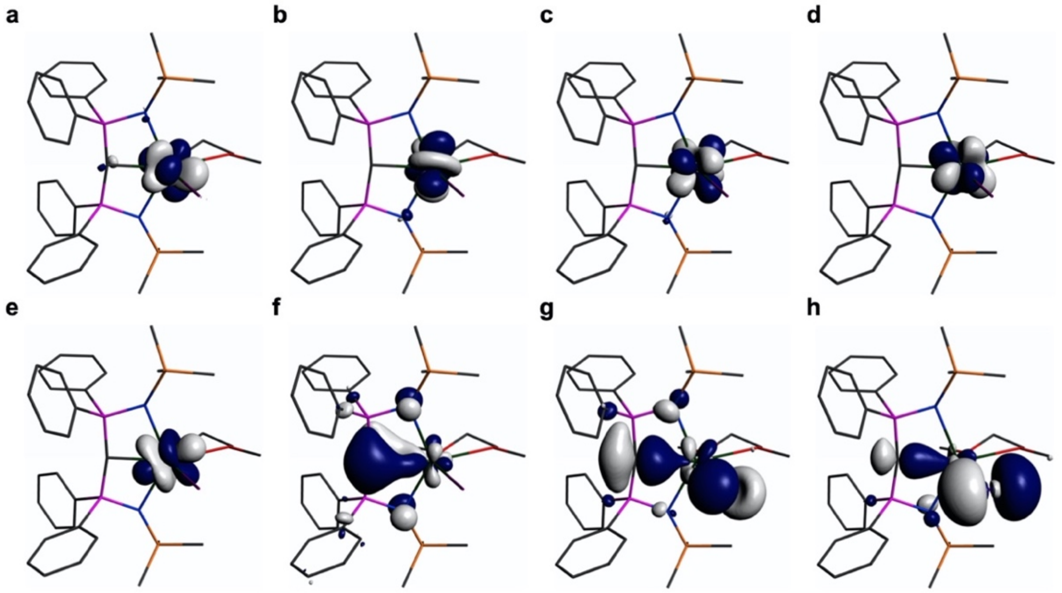

Figure S80. Frontier Kohn Sham Molecular Orbitals of **2Pu** with H-atoms omitted for clarity. (a) HOMO (249a, –3.054 eV). (b) HOMO–1 (248a, –3.103 eV). (c) HOMO–2 (247a, –3.235 eV). (d) HOMO–3 (246a, –3.261 eV). (e) HOMO–4 (245a, –3.321 eV). (f) HOMO–5 (244a, –4.550 eV). (g) HOMO–6 (243a, –4.985 eV). (h) HOMO–8 (241a, –5.204 eV).

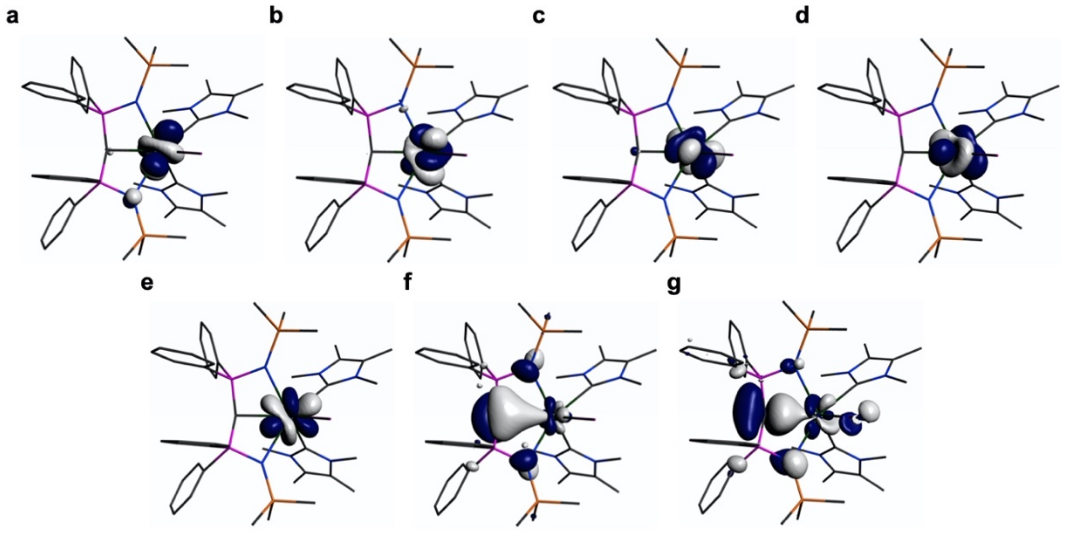

Figure S81. Frontier Kohn Sham Molecular Orbitals of **3Pu** with H-atoms omitted for clarity. (a) HOMO (292a, –2.798 eV). (b) HOMO–1 (291a, –2.839 eV). (c) HOMO–2 (290a, –2.906 eV). (d) HOMO–3 (289a, –3.024 eV). (e) HOMO–4 (288a, –3.053 eV). (f) HOMO–5 (287a, –4.306 eV). (g) HOMO–6 (286a, –4.509 eV).

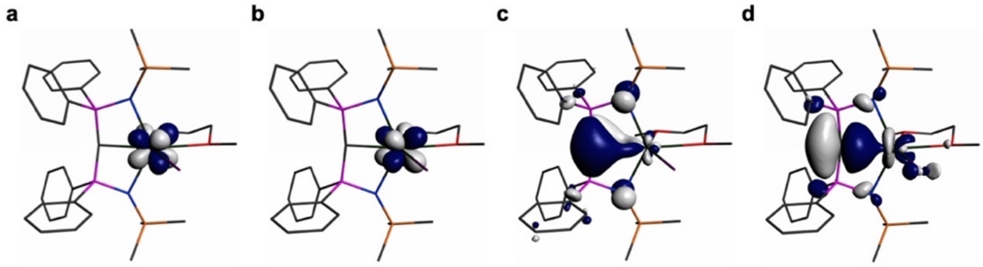

Figure S82. Frontier Kohn Sham Molecular Orbitals of **2Pr** with H-atoms omitted for clarity. (a) HOMO (230a, –2.907 eV). (b) HOMO–1 (229a, –2.922 eV). (c) HOMO–2 (228a, –4.441 eV). (d) HOMO–3 (227a, –4.780 eV).

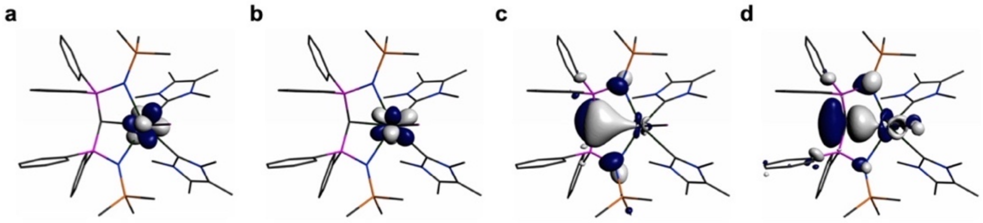

Figure S83. Frontier Kohn Sham Molecular Orbitals of **3Pr** with H-atoms omitted for clarity. (a) HOMO (273a, –2.544 eV). (b) HOMO–1 (272a, –2.583 eV). (c) HOMO–2 (271a, –4.230 eV). (d) HOMO–3 (270a, –4.392 eV).

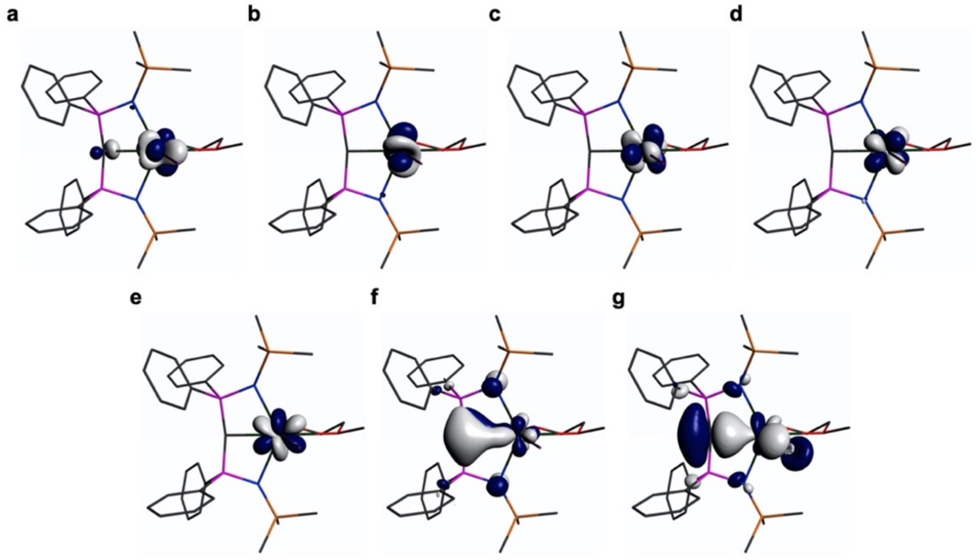

Figure S84. Frontier Kohn Sham Molecular Orbitals of **2Sm** with H-atoms omitted for clarity. (a) HOMO (233a, –3.927 eV). (b) HOMO–1 (232a, –3.953 eV). (c) HOMO–2 (231a, –4.040 eV). (d) HOMO–3 (230a, –4.066 eV). (e) HOMO–4 (229a, –4.154 eV). (f) HOMO–5 (228a, –4.516 eV). (g) HOMO–6 (227a, –4.881 eV).

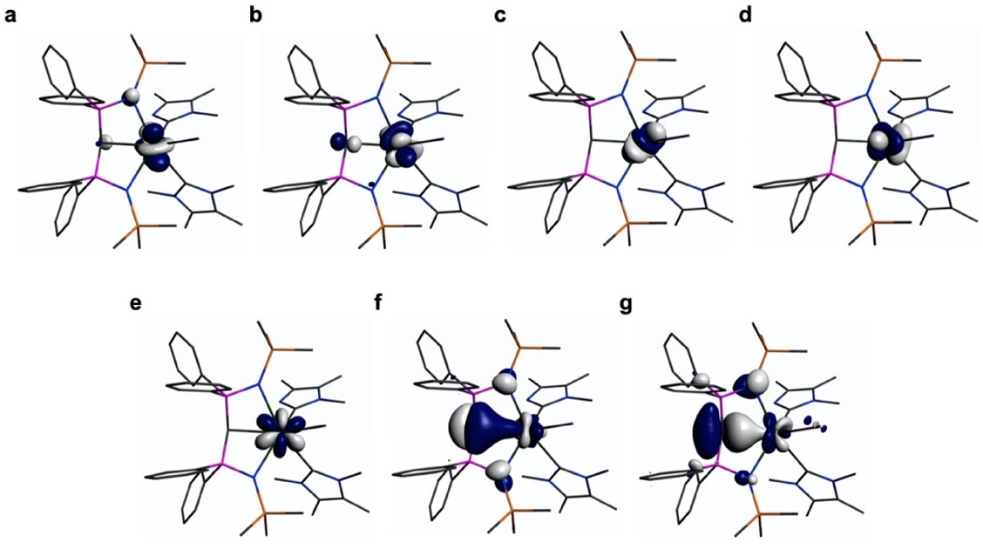

Figure S85. Frontier Kohn Sham Molecular Orbitals of **3Sm** with H-atoms omitted for clarity. (a) HOMO (276a, –3.688 eV). (b) HOMO–1 (275a, –3.724 eV). (c) HOMO–2 (274a, –3.769 eV). (d) HOMO–3 (273a, –3.823 eV). (e) HOMO–4 (272a, –3.927 eV). (f) HOMO–5 (271a, –4.287 eV). (g) HOMO–6 (270a, –4.484 eV).

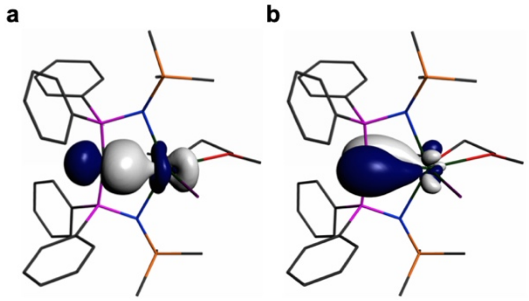

Figure S86. Natural Bond Orbitals of **2Pu** with H-atoms omitted for clarity. (a) Pu=C σ-bond. (b) Pu=C π-bond.

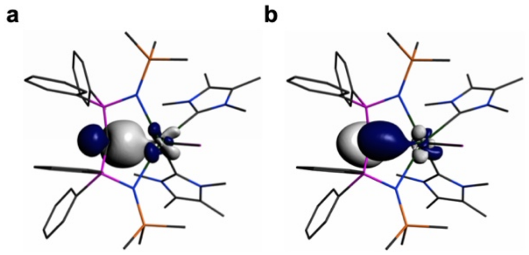

Figure S87. Natural Bond Orbitals of **3Pu** with H-atoms omitted for clarity. (a) Pu=C σ-bond. (b) Pu=C π-bond.

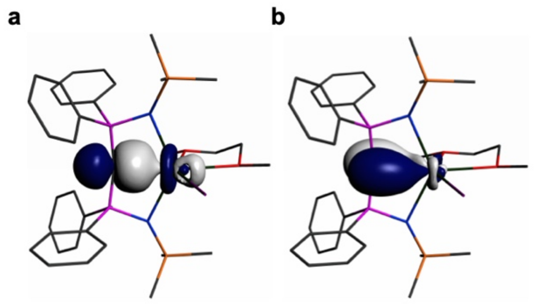

Figure S88. Natural Bond Orbitals of **2Pr** with H-atoms omitted for clarity. (a) Pr=C σ-bond. (b) Pr=C π-bond.

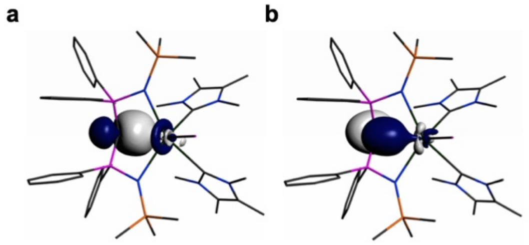

Figure S89. Natural Bond Orbitals of **3Pr** with H-atoms omitted for clarity. (a) Pr=C σ-bond. (b) Pr=C π-bond.

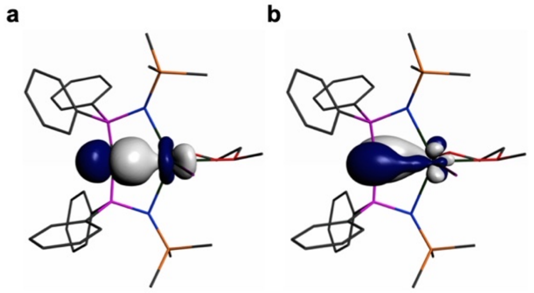

Figure S90. Natural Bond Orbitals of **2Sm** with H-atoms omitted for clarity. (a) Sm=C σ-bond. (b) Sm=C π-bond.

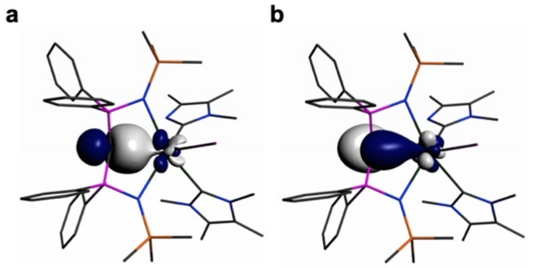

Figure S91. Natural Bond Orbitals of **3Sm** with H-atoms omitted for clarity. (a) Sm=C σ-bond. (b) Sm=C π-bond.

CASSCF-SO modelling

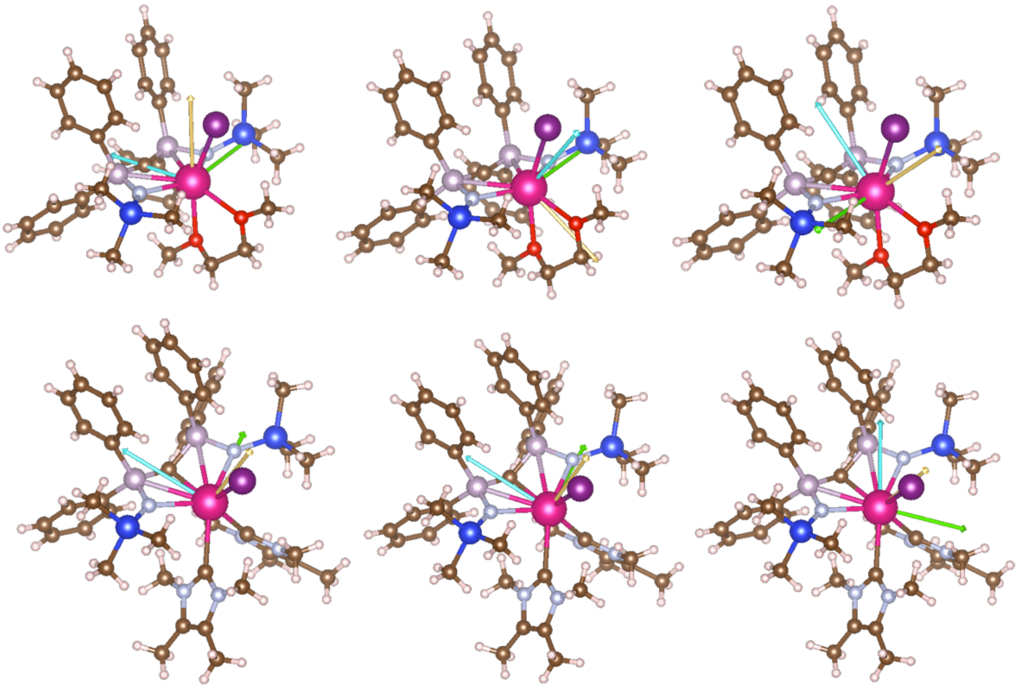

Figure S92. Principal axes for the ground state g-values of **2Sm** (top) and **3Sm** (bottom) in the molecular coordinate frame as calculated via CASSCF-SO (left), MS-CASPT2 (middle), and XMS-CASPT2 (right). The g_1 axis green, g_2 is cyan, and g_3 is yellow.

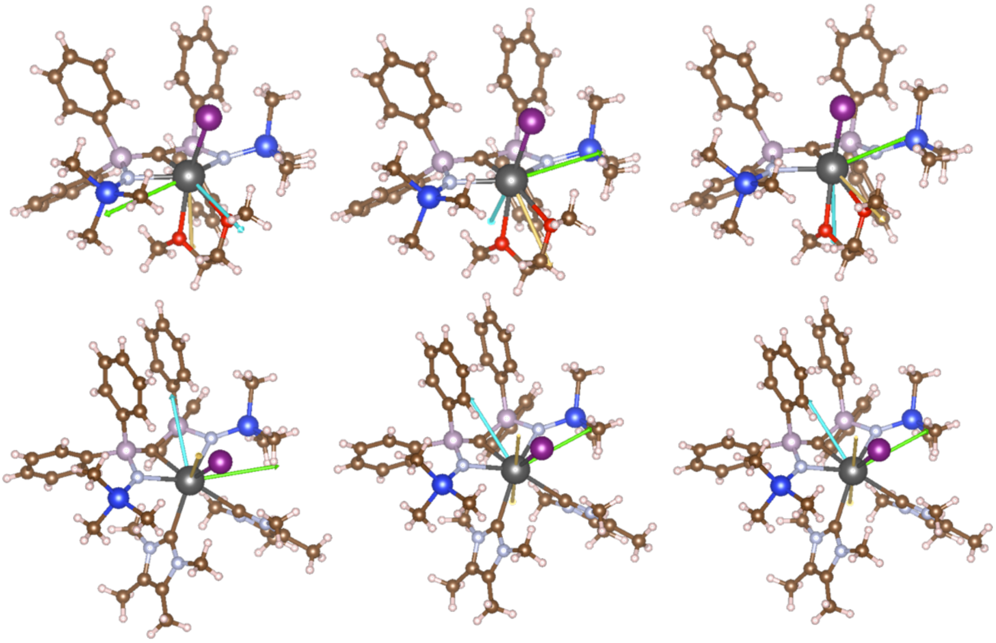

Figure S93. Principal axes for the ground state g-values of **2Pu** (top) and **3Pu** (bottom) in the molecular coordinate frame as calculated via CASSCF-SO (left), MS-CASPT2 (middle), and XMS-CASPT2 (right). The g_1 axis green, g_2 is cyan, and g_3 is yellow.

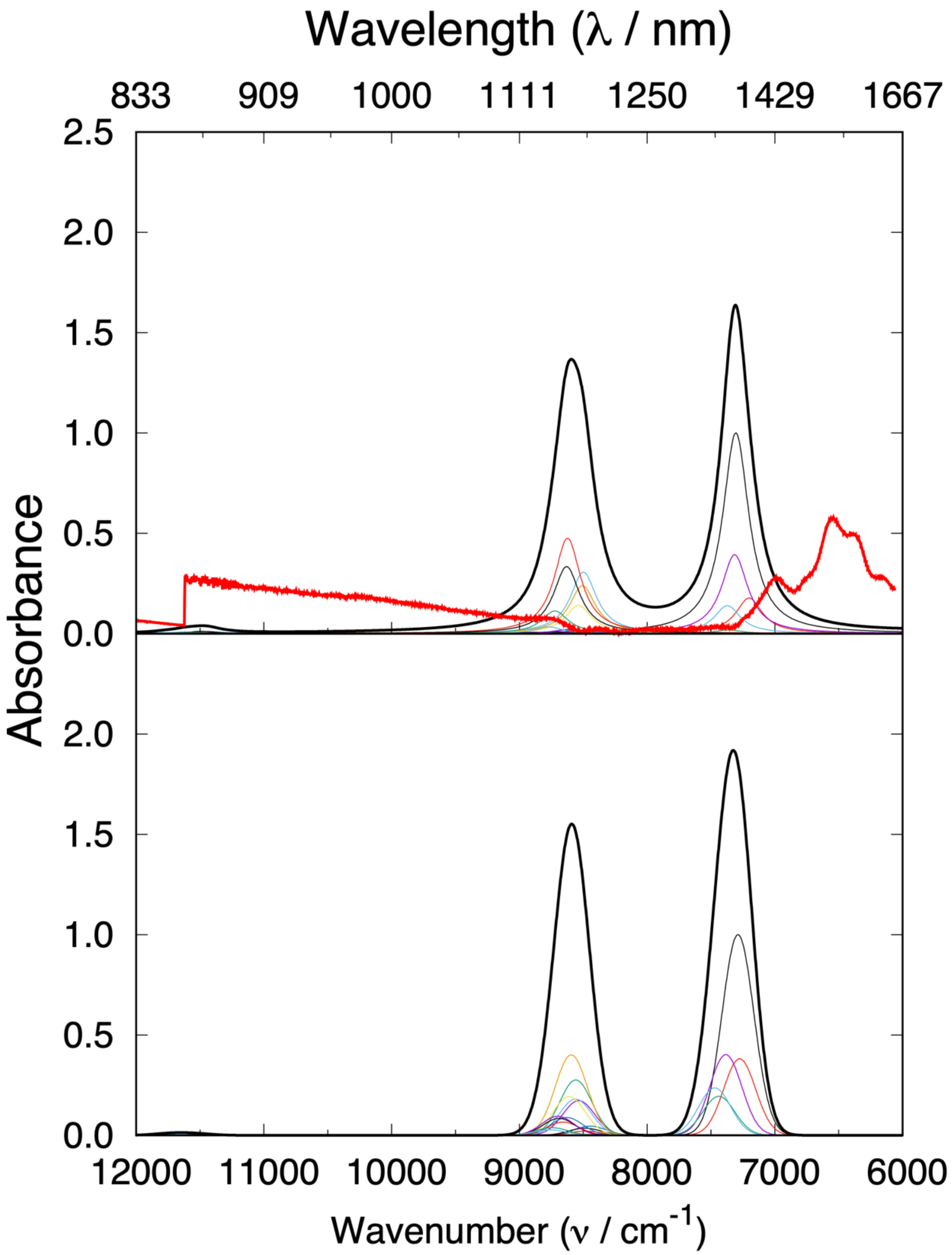

Figure S94. Experimental UV/Vis/NIR (red) compared to CASSCF-SO (black) results calculated for top: **2Pr** and bottom: **3Pr**. Individual transitions (rainbow, below black) and total absorption spectrum was Gaussian-broadened with a half-width of 250 cm^{-1}.

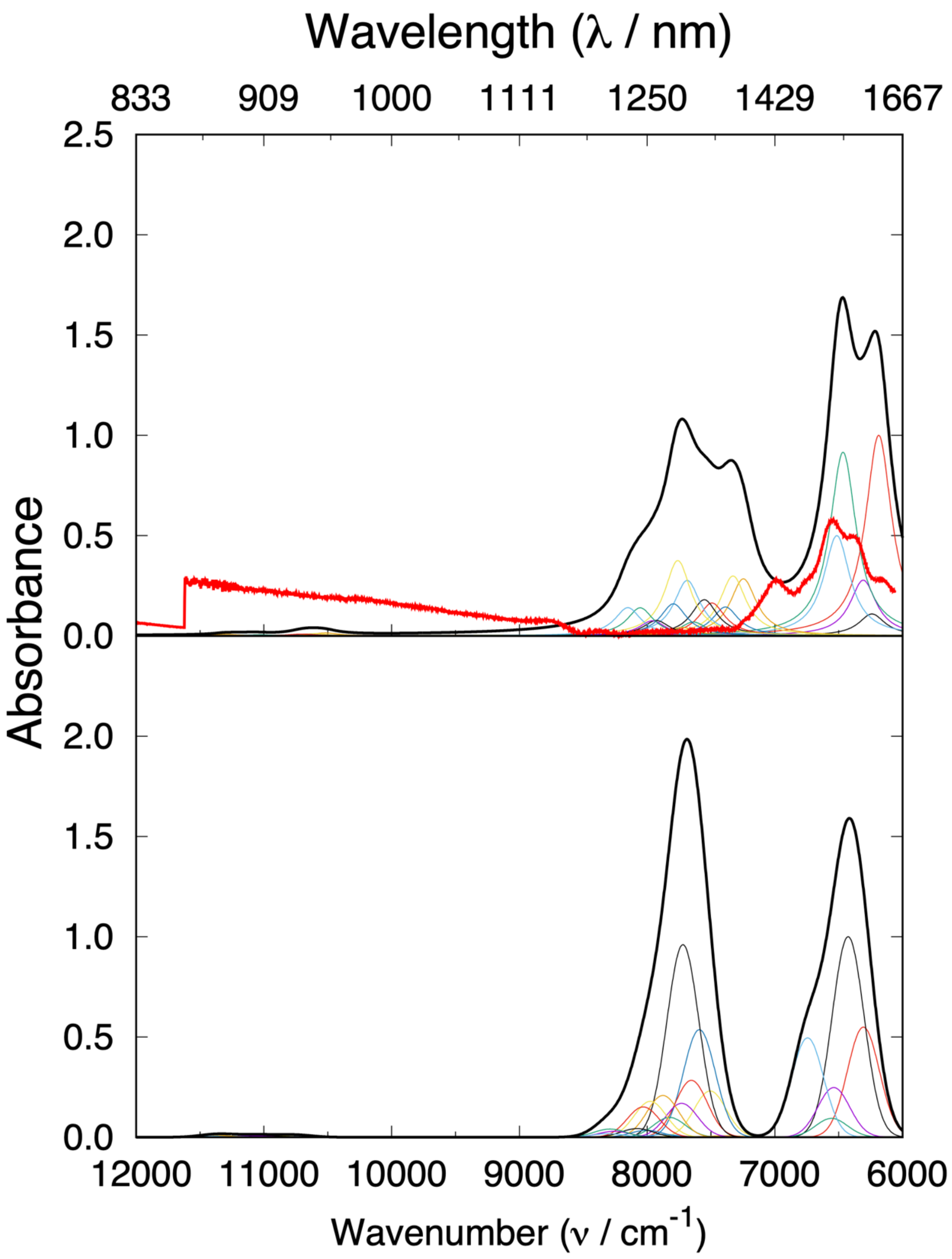

Figure S95. Experimental UV/Vis/NIR (red) compared to XMS-CASPT2-SO (black) results calculated for top: **2Pr** and bottom: **3Pr**. Individual transitions (rainbow, below black) and total absorption spectrum was Gaussian-broadened with a half-width of 250 cm^{-1}.

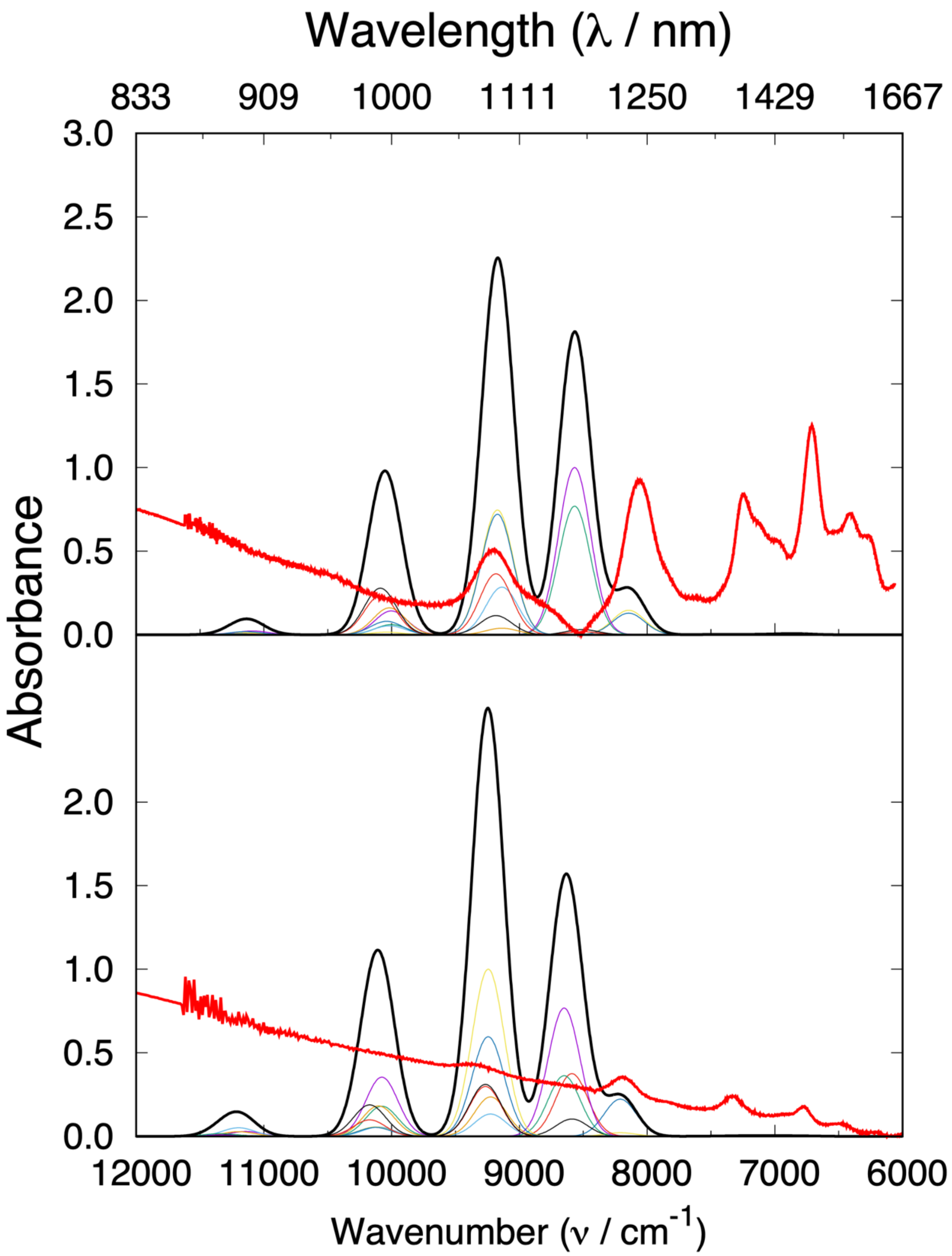

Figure S96. Experimental UV/Vis/NIR (red) compared to CASSCF-SO (black) results calculated for top: **2Sm** and bottom: **3Sm**. Individual transitions (rainbow, below black) and total absorption spectrum was Gaussian-broadened with a half-width of 250 cm^{-1}.

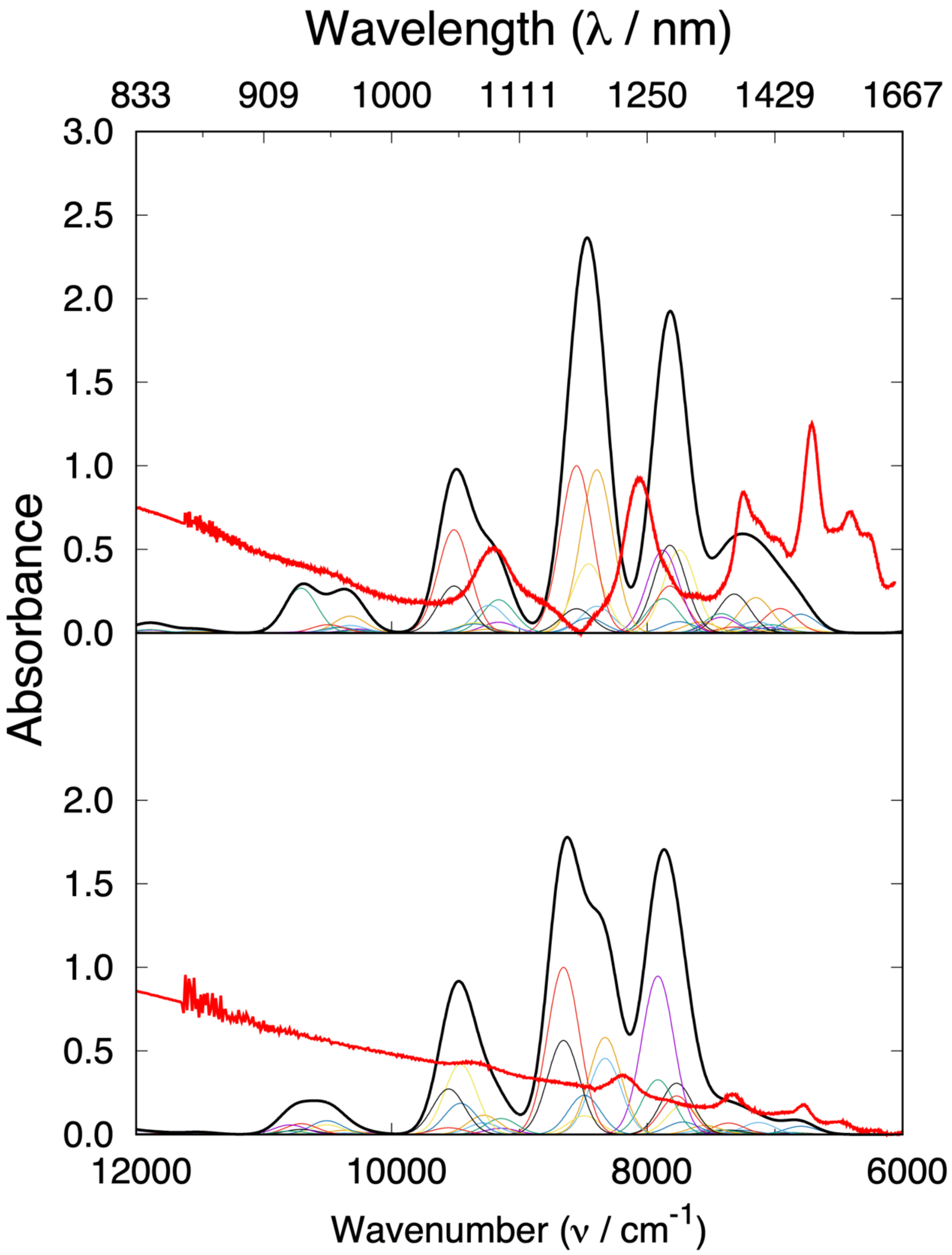

Figure S97. Experimental UV/Vis/NIR (red) compared to XMS-CASPT2-SO (black) results calculated for top: **2Sm** and bottom: **3Sm**. Individual transitions (rainbow, below black) and total absorption spectrum was Gaussian-broadened with a half-width of 250 cm^{-1}.

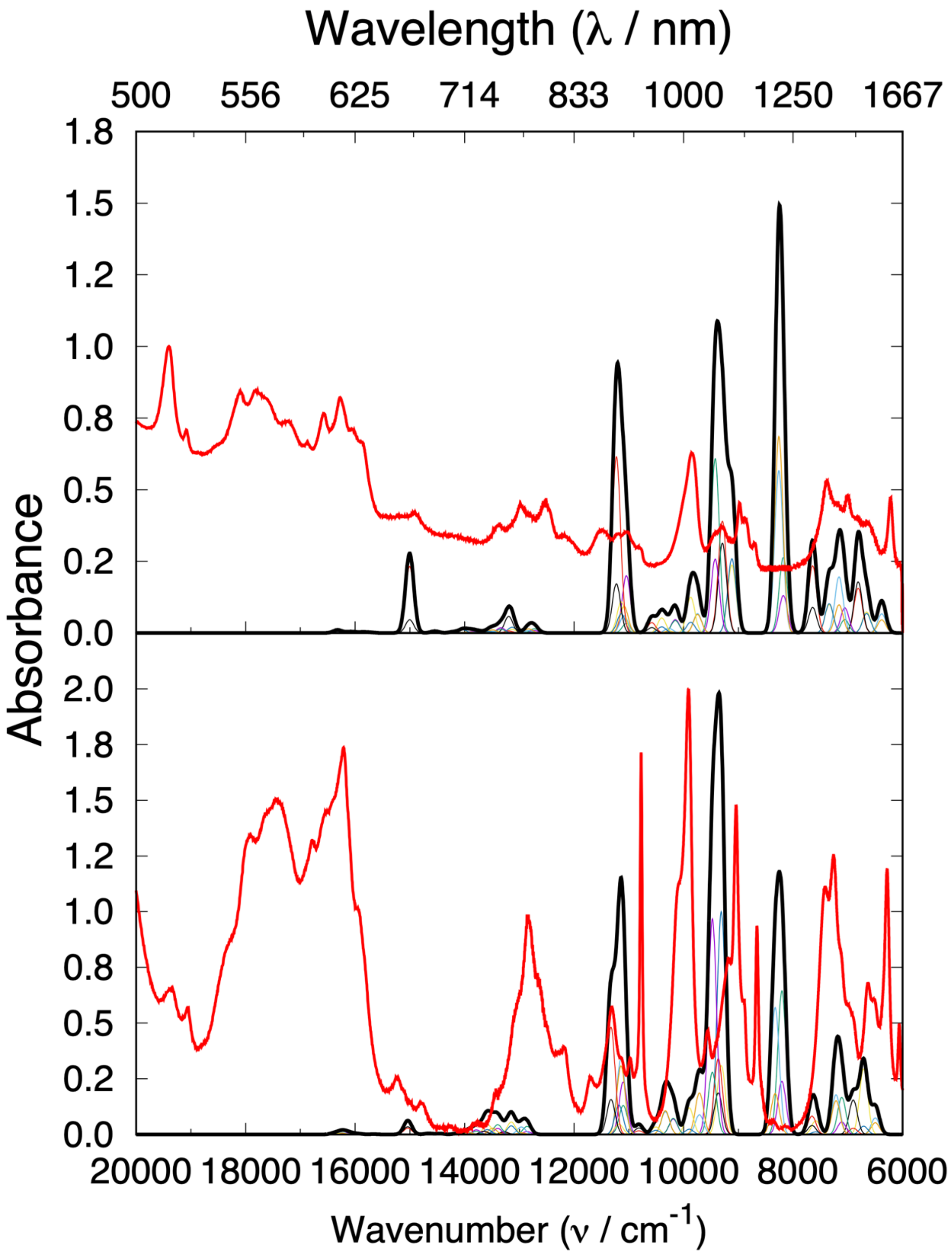

Figure S98. Experimental UV/Vis/NIR (red) compared to CASSCF-SO (black) results calculated for top: **2Pu** and bottom: **3Pu**. Individual transitions (rainbow, below black) and total absorption spectrum was Gaussian-broadened with a half-width of 150 cm^{-1}.

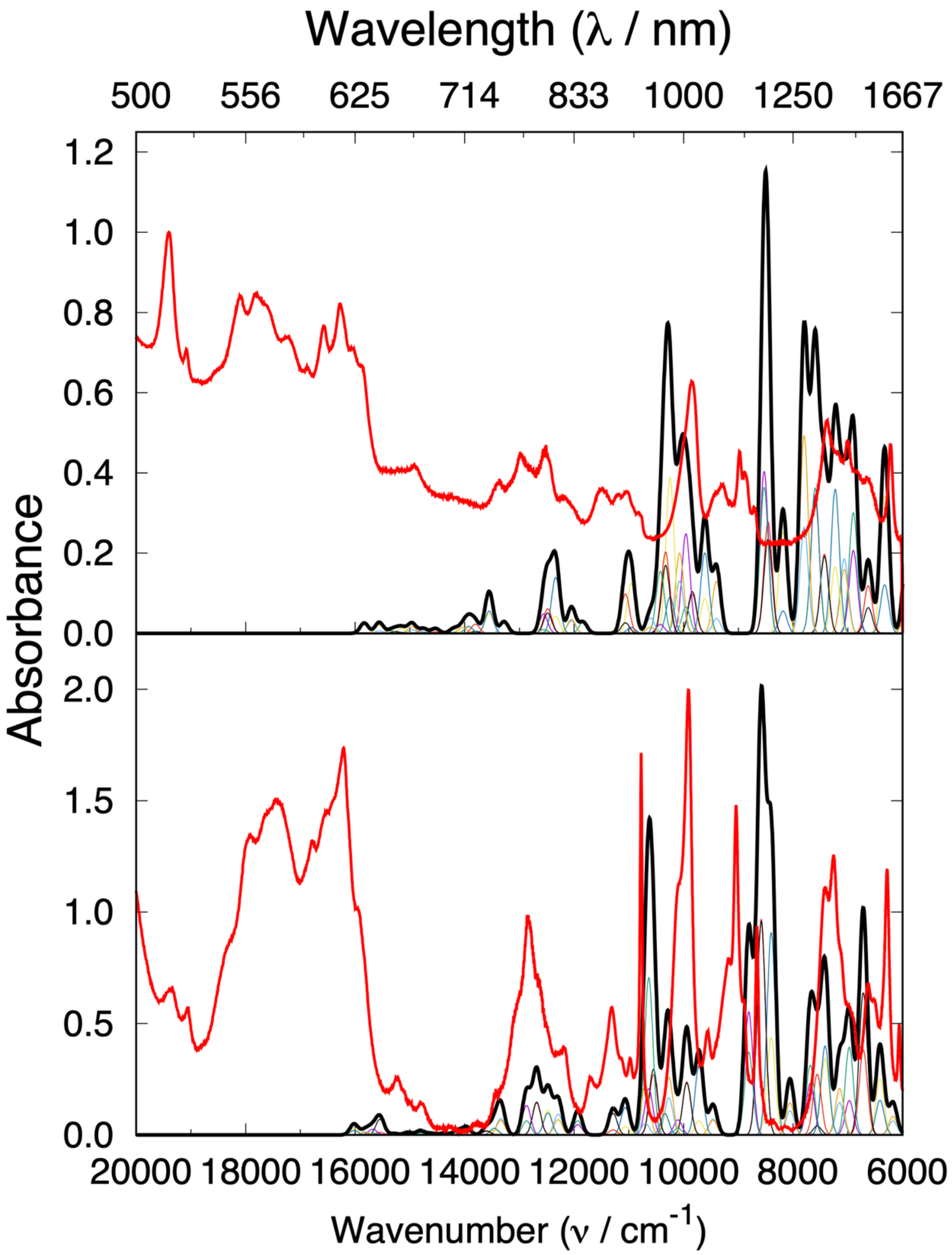

Figure S99. Experimental UV/Vis/NIR (red) compared to XMS-CASPT2-SO (black) results calculated for top: **2Pu** and bottom: **3Pu**. Individual transitions (rainbow, below black) and total absorption spectrum was Gaussian-broadened with a half-width of 150 cm^{-1}.

Tables

Table S1. Crystallographic data for 1Pu-3Pu

Compound	**1Pu**	**2Pu**	**3Pu**
Formula	$C_{74}H_{90}I_4N_4P_4Pu_2Si_4$	$C_{38.50}H_{52}INO_2P_2PuSi_2$	$C_{48.50}H_{66}IN_6P_2PuSi_2$
Fw, g mol^{-1}	2257.33	1058.84	1217.09
Cryst size, mm	0.10 × 0.10 × 0.10	0.12 x 0.05 x 0.02	0.5 × 0.4 × 0.2
Crystal system	monoclinic	monoclinic	monoclinic
Space group	$P2_1/c$	$P2_1/n$	$P2_1/n$
Temperature (K)	150(2)	100(2)	100(2)
a, (Å)	15.8494(5)	9.5777(8)	15.8062(6)
b, (Å)	12.7081(5)	21.9566(19)	20.4275(7)
c, (Å)	20.9632(8)	20.5720(15)	16.3574(6)
α, (°)	90	90	90
β, (°)	96.216(4)	99.283(3)	100.482(2)
γ, (°)	90	90	90
V, (Å^3)	4197.5(3)	4269.5(6)	5193.4(3)
Z	2	4	4
ρ_{calc} g cm^{-3}	1.791	1.652	1.560
μ, mm^{-1}	3.201	2.431	2.009
Measured reflections	54832	72532	91005
Unique reflections, R_{int}	10331, 0.1061	8710, 0.1392	12886, 0.0795
$F^2 > 2\sigma(F^2)$ reflections	8819	6708	11029
Transmission range	0.00020-1.00000	0.5214-0.7458	0.5964-0.7461
R, $R_w{}^a$ ($F^2 > 2\sigma(F^2)$)	0.0392, 0.0939	0.0389, 0.0801	0.0415, 0.0771
R, $R_w{}^a$ (all data)	0.0465, 0.0997	0.0617, 0.0871	0.0520, 0.0798
S^a	1.016	1.047	1.155
Parameters, Restraints	421, 6	443, 29	592, 95
Max.,min. difference map, e Å^{-3}	1.997, −2.417	1.575, -1.668	1.821, −2.626

^{a} Conventional $R = \Sigma||F_o| - |F_c||/\Sigma|F_o|$; $R_w = [\Sigma w(F_o^2 - F_c^2)^2/\Sigma w(F_o^2)^2]^{1/2}$;
$S = [\Sigma w(F_o^2 - F_c^2)^2/\text{no. data} - \text{no. params})]^{1/2}$ for all data.

Table S2. Crystallographic data for 1Pr.THF, 1Pr.IMe4, and 2Pr

Compound	**1Pr.THF**	**1Pr.IMe4**	**2Pr**
Formula	$C_{42}H_{55}I_2N_2OP_2PrSi_2$	$C_{38}H_{51}I_2N_4P_2PrSi_2$	$C_{35}H_{48}IN_2O_2P_2PrSi_2$
Fw, g mol^{-1}	1116.71	1076.66	914.68
Cryst size, mm	0.32 × 0.07 × 0.05	0.099 x 0.064 x 0.05	0.28 × 0.11 × 0.07
Crystal system	triclinic	monoclinic	monoclinic
Space group	P-1	C2/c	$P2_1/n$
Temperature (K)	120(2)	150(2)	120(2)
a, (Å)	9.8555(3)	19.7730(5)	9.8828(2)
b, (Å)	12.4432(4)	11.9298(3)	20.5276(4)
c, (Å)	19.9462(8)	36.6777(9)	20.5581(4)
α, (°)	106.198(3)	90	90
β, (°)	96.325(3)	99.055(2)	102.248(2)
γ, (°)	99.004(3)	90	90
V, (Å^3)	2289.35(14)	8544.0(4)	4075.67(15)
Z	2	8	4
ρ_{calc} g cm^{-3}	1.620	1.674	1.491
μ, mm^{-1}	20.160	21.577	16.658
Measured reflections	16609	53705	14646
Unique reflections, R_{int}	9071, 0.0375	9254, 0.1278	8027, 0.0439
$F^2 > 2\sigma(F^2)$ reflections	8261	8040	7213
Transmission range	0.112-0.559	0.844-1.000	0.113-0.555
R, R_w^{a} ($F^2 > 2\sigma(F^2)$)	0.0341, 0.0855	0.0871, 0.1967	0.0538, 0.1519
R, R_w^{a} (all data)	0.0383, 0.0893	0.0965, 0.2019	0.0586, 0.1591
S^a	1.029	1.091	1.052
Parameters, Restraints	476, 0	452, 4	414, 0
Max.,min. difference map, e Å^{-3}	1.185, −1.530	2.149, -1.844	1.831, −1.793

^{a} Conventional $R = \Sigma||F_o| - |F_c||/\Sigma|F_o|$; $R_w = [\Sigma w(F_o^2 - F_c^2)^2/\Sigma w(F_o^2)^2]^{1/2}$;
$S = [\Sigma w(F_o^2 - F_c^2)^2/\text{no. data} - \text{no. params})]^{1/2}$ for all data.

Table S3. Crystallographic data for 3Pr, 1Sm.THF, and 1Sm.IMe4

Compound	**3Pr**	**1Sm.THF**	**1Sm.IMe4**
Formula	$C_{48.50}H_{66}IN_6P_2PrSi_2$	$C_{39}H_{55}I_2N_2O_2P_2Si_2Sm$	$C_{38}H_{51}I_2N_4P_2Si_2Sm$
Fw, g mol^{-1}	1119.00	1106.12	1086.09
Cryst size, mm	0.23 × 0.18 × 0.12	0.78 × 0.50 × 0.32	0.09 × 0.05 × 0.04
Crystal system	monoclinic	monoclinic	monoclinic
Space group	$P2_1/n$	$P2_1/c$	C2/c
Temperature (K)	150(2)	150(2)	150(2)
a, (Å)	15.84620(10)	16.2927(4)	19.8770(17)
b, (Å)	20.5167(2)	19.5876(4)	11.9099(6)
c, (Å)	16.42100(10)	14.2818(3)	36.4579(19)
α, (°)	90	90	90
β, (°)	100.5720(10)	99.109(2)	99.192(6)
γ, (°)	90	90	90
V, (Å^3)	5248.04(7)	4500.34(17)	8520.0(10)
Z	4	4	8
ρ_{calc} g cm^{-3}	1.416	1.633	1.693
μ, mm^{-1}	13.037	2.834	23.215
Measured reflections	123045	46714	28259
Unique reflections, R_{int}	11431, 0.0675	11583, 0.0491	8471, 0.1191
$F^2 > 2\sigma(F^2)$ reflections	11230	9475	5003
Transmission range	0.151-0.670	0.077-1.000	0.271-0.735
R, $R_w{}^a$ ($F^2 > 2\sigma(F^2)$)	0.0308, 0.0789	0.0346, 0.0755	0.0790, 0.1977
R, $R_w{}^a$ (all data)	0.0315, 0.0793	0.0472, 0.0806	0.1379, 0.2451
S^a	1.067	1.036	1.047
Parameters, Restraints	592, 114	467, 4	452, 276
Max.,min. difference map, e Å^{-3}	0.668, −1.043	1.092, −1.164	1.329, −2.556

^{a} Conventional $R = \Sigma||F_o| - |F_c||/\Sigma|F_o|$; $R_w = [\Sigma w(F_o^2 - F_c^2)^2/\Sigma w(F_o^2)^2]^{1/2}$;
$S = [\Sigma w(F_o^2 - F_c^2)^2/\text{no. data} - \text{no. params})]^{1/2}$ for all data.

Table S4. Crystallographic data for 2Sm and 3Sm

Compound	**2Sm**	**3Sm**
Formula	$C_{52.50}H_{68}IN_2O_2P_2Si_2Sm$	$C_{48.5}0H_{66}IN_6P_2Si_2Sm$
Fw, g mol^{-1}	1154.45	1128.44
Cryst size, mm	0.26 × 0.16 × 0.13	0.23 × 0.16 × 0.13
Crystal system	triclinic	monoclinic
Space group	P-1	$P2_1/n$
Temperature (K)	150(2)	150(2)
a, (Å)	9.88120(10)	15.85410(10)
b, (Å)	16.9346(2)	20.4793(2)
c, (Å)	17.1021(2)	16.38160(10)
α, (°)	91.7890(10)	90
β, (°)	105.1060(10)	100.6230(10)
γ, (°)	90.1460(10)	90
V, (Å^3)	2761.36(6)	5227.63(7)
Z	2	4
ρ_{calc} g cm^{-3}	1.388	1.434
μ, mm^{-1}	13.625	14.373
Measured reflections	60905	119187
Unique reflections, R_{int}	11977, 0.0652	11370, 0.0867
$F^2 > 2\sigma(F^2)$ reflections	11369	11097
Transmission range	0.277-1.000	0.124-0.462
R, $R_w{}^a$ ($F^2 > 2\sigma(F^2)$)	0.0464, 0.1243	0.0363, 0.0958
R, $R_w{}^a$ (all data)	0.0481, 0.1258	0.0373, 0.0966
S^a	1.050	1.059
Parameters, Restraints	620, 307	592, 114
Max.,min. difference map, e Å^{-3}	1.530, −0.961	0.614, −1.112

^{a} Conventional $R = \Sigma||F_o| - |F_c||/\Sigma|F_o|$; $R_w = [\Sigma w(F_o^2 - F_c^2)^2/\Sigma w(F_o^2)^2]^{1/2}$;
$S = [\Sigma w(F_o^2 - F_c^2)^2/\text{no. data} - \text{no. params})]^{1/2}$ for all data.

Table S5. Evans method determination of the magnetic moments of 1Pu-3Pu

**Entry**	**μ_{eff} / μ_B**	**Compound mass / g^a**	**Solvent mass / g**	**M_r / g mol^{-1}**	**Δ peak / Hz**
**1Pu**	3.65 (1.83)*	0.0029	0.3529	2073.05	35.01
**2Pu**	1.35	0.0069	0.4970	1012.77	12.58
**3Pu**	0.90	0.0049	0.2800	1170.96	3.32

^{a} The small masses engender large errors in this methodology, the results should be cautiously interpreted along with other data. ^{b} Spectrometer frequency 400.130 MHz. Simple diamagnetic correction of M_r / −2,000,000 applied. ρd_6-benzene = 0.950 g mL^{-1}; ρd_8-THF = 0.985 g mL^{-1}. *For **1PU**, the value given is for the molecular dimer while the value in brackets is per magnetic center assuming no interactions between the Pu ions.

Table S6. Coordinates and final energy for a single point energy calculation on geometry optimized 2Pu

1.C	-2.771751	-3.290403	-3.550874
2.C	1.345019	0.334624	-3.429705
3.C	-3.275909	3.226956	-3.032837
4.C	-1.732032	-4.090120	-3.058836
5.C	3.412691	1.493059	-3.048638
6.C	-1.913700	3.532559	-2.941810
7.C	-2.981061	-2.017040	-3.012797
8.C	2.732652	-3.468561	-2.676558
9.C	4.705460	1.341991	-2.279620
10.C	-3.872617	2.424927	-2.052152
11.C	-0.914408	-3.618745	-2.028966
12.C	-1.155044	3.038122	-1.876329
13.C	-2.153351	-1.543579	-1.988454
14.C	-1.114827	-2.338869	-1.484727
15.C	-3.111889	1.930244	-0.988719
16.C	-1.741672	2.231989	-0.889054
17.C	4.377106	-2.586688	-0.283251
18.C	5.627483	1.494551	-0.078531
19.C	2.283041	-4.807228	0.059775
20.C	-0.205777	-0.055990	0.157421
21.C	0.230198	5.338481	0.539941
22.C	3.012362	4.151344	0.969702
23.C	-1.719985	-3.569912	1.292674
24.C	-0.568534	-2.765138	1.308440
25.C	-1.723550	1.742081	1.948490
26.C	-2.559603	2.858040	2.130616
27.C	-2.062964	-4.339722	2.409718
28.C	0.224567	-2.732630	2.470209
29.C	-1.647193	0.771814	2.959230
30.C	0.918484	3.859307	3.156774
31.C	-3.307380	2.996407	3.303083
32.C	-1.264905	-4.307958	3.556753
33.C	-0.122515	-3.496865	3.585274
34.C	-2.391478	0.913295	4.133563
35.C	-3.224118	2.023990	4.307055
36.H	-3.415337	-3.660797	-4.350986
37.H	3.611137	1.318282	-4.120708
38.H	1.687285	-0.047438	-4.404145
39.H	-3.872132	3.619375	-3.858403
40.H	-1.444477	4.167963	-3.695279
41.H	-1.562311	-5.084201	-3.475560
42.H	0.799343	1.281052	-3.564120
43.H	-3.790201	-1.388608	-3.388557
44.H	3.011329	-2.541930	-3.202254
45.H	2.993201	2.507903	-2.928314
46.H	1.778141	-3.814195	-3.099746
47.H	3.498756	-4.227531	-2.906249

48.H	5.402301	2.145914	-2.575415
49.H	0.693610	-0.400264	-2.947420
50.H	5.175675	0.366619	-2.496653
51.H	-4.936936	2.191362	-2.111248
52.H	-0.120871	-4.256423	-1.641657
53.H	-0.097880	3.287841	-1.784446
54.H	-2.306503	-0.546409	-1.574492
55.H	4.685668	-1.688345	-0.845050
56.H	5.124984	-3.367710	-0.494839
57.H	6.171976	2.424761	-0.302429
58.H	0.464669	5.475226	-0.527034
59.H	6.265728	0.622163	-0.286646
60.H	-3.590205	1.316391	-0.223614
61.H	1.311725	-5.238421	-0.227221
62.H	3.060690	-5.545775	-0.195589
63.H	3.270219	4.137609	-0.100400
64.H	-2.351946	-3.604889	0.404488
65.H	-0.861237	5.227981	0.627029
66.H	4.427429	-2.346139	0.789855
67.H	5.315297	1.472001	0.971240
68.H	0.510683	6.266547	1.064385
69.H	2.273884	-4.687230	1.153310
70.H	3.307345	5.133014	1.374730
71.H	-2.637290	3.617524	1.351713
72.H	3.619838	3.391427	1.487015
73.H	-2.955109	-4.967221	2.377957
74.H	1.118919	-2.106641	2.501228
75.H	-1.008256	-0.099971	2.812933
76.H	-0.142105	3.786563	3.441232
77.H	-3.959160	3.861955	3.431480
78.H	1.323618	4.787001	3.593585
79.H	1.450226	3.008014	3.609489
80.H	-1.530321	-4.912926	4.425619
81.H	0.504819	-3.462329	4.477139
82.H	-2.325305	0.149219	4.909029
83.H	-3.812165	2.129617	5.220628
84.I	3.392645	0.077821	2.895946
85.N	1.537010	-1.888368	-0.396107
86.N	0.733250	2.351309	0.512711
87.O	2.482085	0.517346	-2.552955
88.O	4.418982	1.434034	-0.872709
89.P	-0.064298	-1.704928	-0.108943
90.P	-0.685028	1.521465	0.449546
91.Pu	2.136905	0.419024	0.081174
92.Si	2.649401	-3.165928	-0.804082
93.Si	1.166953	3.867190	1.287789

Energy: –545.88616850 eV

Table S7. Coordinates and final energy for a single point energy calculation on geometry optimized 3Pu

1.C	-4.364391	0.265113	-3.257484
2.C	4.480428	-0.258045	-3.216392
3.C	1.935804	-3.205334	-3.051881
4.C	-5.568431	-0.153723	-2.680827
5.C	0.139108	4.138547	-2.727953
6.C	-3.293722	0.646529	-2.444753
7.C	-3.848638	-3.412157	-2.079358
8.C	6.912009	-1.470508	-1.919763
9.C	-2.795676	4.268785	-2.060262
10.C	-0.152296	-4.969030	-1.783288
11.C	3.603312	3.065733	-1.742066
12.C	-2.680800	-2.793170	-1.629481
13.C	-5.692564	-0.197887	-1.287502
14.C	-4.760718	-3.943686	-1.161150
15.C	5.627712	-1.220381	-1.200807
16.C	-3.412875	0.613984	-1.045479
17.C	3.440656	-0.531297	-0.955593
18.C	-4.620899	0.179862	-0.473614
19.C	2.369396	-4.276743	-0.234779
20.C	-2.407424	-2.697820	-0.255080
21.C	-4.505897	-3.838662	0.210627
22.C	5.262638	-1.477740	0.095542
23.C	4.589061	5.106056	0.257793
24.C	-0.701913	4.895002	0.095499
25.C	2.213395	2.241179	0.157227
26.C	3.605132	4.030473	0.579480
27.C	-3.340021	-3.213360	0.662305
28.C	-0.941025	-0.157308	0.516816
29.C	6.019437	-2.113022	1.214901
30.C	-3.792390	2.922414	1.169796
31.C	-2.745173	2.008849	1.379176
32.C	3.168252	-1.122386	1.444286
33.C	2.967954	3.722319	1.753560
34.C	-0.484289	-2.672168	1.889319
35.C	-4.310642	3.672045	2.228874
36.C	-0.472871	-4.071419	2.021585
37.C	1.242317	2.028585	2.441157
38.C	-2.259824	1.835127	2.682835
39.C	3.057517	4.346560	3.106644
40.C	-0.145407	-1.893433	3.005170
41.C	-0.127132	-4.672885	3.234824
42.C	-3.790484	3.517876	3.519316
43.C	-2.768345	2.589421	3.745759
44.C	0.212558	-2.489708	4.219302
45.C	0.222471	-3.883548	4.337648
46.H	-4.255380	0.298181	-4.342821
47.H	4.737050	-1.114415	-3.853143

48.H	1.253935	-2.784219	-3.804800
49.H	-6.406725	-0.449959	-3.314550
50.H	5.195420	0.553710	-3.408663
51.H	3.468022	0.072162	-3.478242
52.H	-0.011072	3.513758	-3.620316
53.H	2.356851	-4.143505	-3.449904
54.H	-4.045179	-3.479300	-3.150506
55.H	0.031819	5.196159	-3.020297
56.H	6.771636	-2.125310	-2.793371
57.H	2.763276	-2.493935	-2.922485
58.H	-2.356768	0.978613	-2.893774
59.H	-3.170492	3.553764	-2.807653
60.H	-2.686824	5.247196	-2.557052
61.H	-0.829412	-4.714587	-2.611682
62.H	7.372790	-0.536796	-2.278323
63.H	1.175276	3.993231	-2.392219
64.H	0.431564	-5.853390	-2.089000
65.H	-1.965988	-2.388425	-2.346227
66.H	3.500567	4.048256	-2.219013
67.H	2.997633	2.346571	-2.303005
68.H	-5.670180	-4.433434	-1.514202
69.H	4.656023	2.754523	-1.778402
70.H	7.631103	-1.959668	-1.251939
71.H	-3.567838	4.384298	-1.286724
72.H	-6.625581	-0.531514	-0.830672
73.H	-0.781825	-5.264822	-0.931389
74.H	4.231517	5.751266	-0.559508
75.H	2.704205	-5.257933	-0.610240
76.H	-0.770641	5.950481	-0.216911
77.H	3.253762	-3.624131	-0.180883
78.H	5.567023	4.698905	-0.043646
79.H	-4.215205	3.038557	0.172594
80.H	-4.729873	0.144678	0.611586
81.H	0.323672	4.717968	0.456886
82.H	-5.214948	-4.246734	0.933078
83.H	7.028545	-2.385536	0.883658
84.H	1.983024	-4.421304	0.785305
85.H	4.750558	5.745790	1.133890
86.H	-1.392304	4.748719	0.940781
87.H	-0.748340	-4.699316	1.174582
88.H	5.526609	-3.032993	1.565294
89.H	2.101880	-1.070173	1.198632
90.H	-3.151117	-3.138681	1.734301
91.H	6.121435	-1.440550	2.080348
92.H	-5.124938	4.375001	2.045988
93.H	3.345209	-2.081035	1.943844
94.H	3.435766	-0.300561	2.121654
95.H	0.648869	1.257221	1.931148
96.H	3.762582	5.186133	3.091824
97.H	-1.488934	1.082704	2.849474
98.H	0.556418	2.780618	2.849866

99.H	-0.182647	-0.808652	2.897333
100.H	-0.133502	-5.760656	3.320938
101.H	2.084415	4.734682	3.443931
102.H	1.815146	1.576941	3.263418
103.H	3.410516	3.631394	3.866204
104.H	-4.190580	4.108145	4.345939
105.H	-2.375507	2.446082	4.754415
106.H	0.471206	-1.866667	5.078093
107.H	0.490423	-4.353948	5.285631
108.I	0.739553	0.503029	-4.290650
109.N	4.506200	-0.644784	-1.811361
110.N	-0.986683	2.056549	-0.923367
111.N	0.332019	-2.079414	-0.807712
112.N	3.131430	3.112758	-0.366023
113.N	3.937135	-1.047581	0.211048
114.N	2.134527	2.635477	1.465432
115.P	-1.956709	1.095551	-0.027483
116.P	-0.875960	-1.835302	0.280722
117.Pu	0.828412	0.339396	-1.156332
118.Si	1.064715	-3.559406	-1.415442
119.Si	-1.106886	3.748263	-1.360212

Energy: −703.71364014 eV

Table S8. Coordinates and final energy for a single point energy calculation on geometry optimized 2Pr

1.C	2.971832	-2.539213	-4.134636
2.C	-3.712442	-1.014678	-3.954619
3.C	1.646996	-2.127044	-3.959350
4.C	-3.751488	0.249072	-3.355795
5.C	-3.097897	-2.080527	-3.284048
6.C	3.904161	-2.340410	-3.107729
7.C	0.107671	2.394502	-2.832595
8.C	1.255432	-1.517087	-2.761936
9.C	-3.179553	0.445403	-2.094078
10.C	4.286195	1.970422	-1.970914
11.C	-2.522423	-1.881951	-2.026547
12.C	3.510817	-1.731597	-1.913669
13.C	0.059759	4.575819	-1.846710
14.C	2.182481	-1.310170	-1.731520
15.C	-2.558941	-0.614569	-1.416333
16.C	-0.370112	5.325085	-0.606488
17.C	-0.015741	-0.213923	-0.109331
18.C	3.774246	3.407382	0.673401
19.C	5.286901	0.748971	0.663855
20.C	-5.092151	1.388712	0.875834
21.C	2.190347	-3.087472	0.902166
22.C	2.211067	-1.698293	1.117925
23.C	-3.489211	-2.262964	1.137662

24.C	-2.225247	-1.660304	1.265444
25.C	0.267777	5.656576	1.676232
26.C	-3.229155	3.626995	1.814692
27.C	2.514303	-3.968885	1.937737
28.C	-3.883438	-3.272838	2.020854
29.C	-1.369088	-2.079659	2.296724
30.C	2.556411	-1.210018	2.389505
31.C	-3.022682	-3.685969	3.043861
32.C	-1.766402	-3.085010	3.181294
33.C	2.858123	-3.474378	3.201074
34.C	-3.452215	1.055061	3.455752
35.C	2.876213	-2.092543	3.424586
36.H	3.277421	-3.023705	-5.064093
37.H	-4.162262	-1.170772	-4.936832
38.H	0.912978	-2.289869	-4.750203
39.H	-4.234311	1.082407	-3.869743
40.H	-3.068256	-3.070487	-3.742782
41.H	-0.577915	2.685017	-3.643019
42.H	4.936707	-2.669278	-3.235320
43.H	1.150755	2.544581	-3.147925
44.H	-0.526823	4.934925	-2.711128
45.H	-0.049491	1.345999	-2.560258
46.H	0.218679	-1.212460	-2.604447
47.H	4.296841	1.027590	-2.538478
48.H	3.555122	2.638794	-2.452722
49.H	5.279005	2.438346	-2.073078
50.H	1.131900	4.743436	-2.052177
51.H	-3.206229	1.426174	-1.618753
52.H	-2.041315	-2.716623	-1.513874
53.H	4.237932	-1.600382	-1.112023
54.H	-0.128004	6.394397	-0.734248
55.H	-1.457795	5.229589	-0.439648
56.H	-5.167023	1.835429	-0.127098
57.H	3.004355	4.050032	0.214705
58.H	5.457848	-0.234713	0.201574
59.H	1.918012	-3.484354	-0.077221
60.H	4.737606	3.931885	0.565920
61.H	6.222890	1.323680	0.569269
62.H	-4.164535	-1.953514	0.338889
63.H	-5.277188	0.308516	0.774801
64.H	-3.241286	4.101352	0.820237
65.H	-5.907236	1.804445	1.490601
66.H	0.746255	6.621746	1.447450
67.H	3.549491	3.330925	1.749059
68.H	5.100131	0.582556	1.734925
69.H	2.494746	-5.044689	1.756715
70.H	-4.862778	-3.739949	1.906213
71.H	-0.780351	5.818010	1.971594
72.H	-4.052811	4.064124	2.401557
73.H	-2.291624	3.896179	2.329213
74.H	0.798233	5.144752	2.486342

75.H	-0.384116	-1.621766	2.398266
76.H	2.557271	-0.134514	2.572788
77.H	-3.602313	-0.035836	3.462902
78.H	-3.327780	-4.479139	3.729085
79.H	-1.088030	-3.402822	3.974030
80.H	-4.269951	1.512978	4.036310
81.H	-2.503568	1.269021	3.971998
82.H	3.109335	-4.163790	4.009384
83.H	3.137284	-1.696983	4.407245
84.N	2.303916	0.959970	0.078464
85.N	-2.063588	1.180607	0.752729
86.O	-0.178725	3.171668	-1.645875
87.O	0.343139	4.788853	0.520952
88.Si	3.864702	1.700258	-0.142306
89.Si	-3.423001	1.741834	1.701856
90.P	1.627128	-0.516217	-0.164628
91.P	-1.663283	-0.300521	0.165852
92.I	0.791662	2.341450	3.763797
93.Pr	0.218060	2.041203	0.714538

Energy: −544.49459129 eV

Table S9. Coordinates and final energy for a single point energy calculation on geometry optimized 3Pr

1.C	-2.471055	-2.454188	-4.233568
2.C	-3.748220	-2.958601	-3.966064
3.C	-4.678335	0.637544	-2.998350
4.C	-1.584144	-2.199557	-3.184189
5.C	-4.137748	-3.195558	-2.642920
6.C	1.025164	-4.700328	-2.713210
7.C	-5.870938	0.341367	-2.329440
8.C	3.278747	-2.703791	-2.560844
9.C	0.004353	4.151230	-2.519121
10.C	-3.491361	0.794799	-2.279735
11.C	3.838505	3.478247	-2.008693
12.C	-2.921616	4.059037	-1.806133
13.C	-1.962594	-2.445439	-1.853686
14.C	-3.252647	-2.938105	-1.592503
15.C	-5.866711	0.186786	-0.938724
16.C	-3.480016	0.660494	-0.881555
17.C	5.042692	0.103069	-0.746549
18.C	4.619979	5.719785	-0.147455
19.C	-4.677620	0.337989	-0.219180
20.C	2.437149	2.704738	-0.089385
21.C	2.536643	-4.284225	-0.077872
22.C	3.681480	4.620289	0.225194
23.C	-1.199070	-4.767169	0.250585
24.C	-0.887983	4.830971	0.309933
25.C	-0.925627	-0.530917	0.228794

26.C	-1.059357	-3.438448	0.686549
27.C	3.093028	-0.409987	0.717960
28.C	-1.347685	-5.809700	1.168037
29.C	6.657882	-1.175812	1.467733
30.C	2.989017	4.389702	1.385900
31.C	5.175686	-1.002681	1.508772
32.C	-2.429807	1.481484	1.672231
33.C	-3.306890	2.572661	1.799712
34.C	-1.106546	-3.177809	2.062778
35.C	1.375330	2.615688	2.155388
36.C	-1.359761	-5.540086	2.541755
37.C	2.935436	5.165585	2.660427
38.C	4.260599	-1.345922	2.470475
39.C	-1.246168	-4.218656	2.987283
40.C	1.755580	-1.206383	2.658821
41.C	-1.921760	0.894433	2.839778
42.C	-3.656912	3.066355	3.059026
43.C	4.438630	-1.993720	3.803645
44.C	-3.131690	2.479049	4.217067
45.C	-2.261758	1.389225	4.103735
46.H	-2.159223	-2.261888	-5.261567
47.H	-4.440918	-3.162167	-4.785085
48.H	-4.671548	0.750336	-4.083599
49.H	0.505327	-4.249072	-3.571197
50.H	-0.585468	-1.813777	-3.393737
51.H	2.965932	-2.160232	-3.464317
52.H	-0.085627	3.509661	-3.408567
53.H	-6.800329	0.228664	-2.891078
54.H	1.824512	-5.349033	-3.108803
55.H	3.928698	-3.540893	-2.865447
56.H	-0.207297	5.191024	-2.819709
57.H	-3.118446	3.567583	-2.770071
58.H	-2.565952	1.039331	-2.801419
59.H	-5.135369	-3.580802	-2.426145
60.H	3.638060	4.403983	-2.562983
61.H	3.326558	2.654270	-2.519912
62.H	0.308982	-5.351932	-2.192544
63.H	-2.972100	5.148402	-1.971147
64.H	4.921629	3.293934	-2.005750
65.H	1.050428	4.105504	-2.183317
66.H	3.887464	-2.019046	-1.953166
67.H	-3.746949	3.787381	-1.131789
68.H	4.249801	0.377300	-1.449083
69.H	5.682887	-0.648871	-1.224555
70.H	4.258433	6.290460	-1.016672
71.H	-6.792318	-0.048286	-0.410140
72.H	-1.207239	-4.986446	-0.816529
73.H	-3.565949	-3.130430	-0.565022
74.H	5.623172	5.339840	-0.396581
75.H	5.642258	0.993857	-0.514954
76.H	3.199995	-5.102214	-0.404855

77.H	-1.177950	5.853546	0.015754
78.H	4.730093	6.422942	0.686752
79.H	6.970664	-1.806400	0.621124
80.H	-1.458984	-6.834210	0.809354
81.H	3.135821	-3.589971	0.533318
82.H	1.756326	-4.719503	0.565225
83.H	0.177284	4.856526	0.585499
84.H	-4.686861	0.218230	0.865390
85.H	-3.728428	3.036796	0.908280
86.H	-1.469964	4.561051	1.203867
87.H	7.185666	-0.213644	1.378404
88.H	0.619083	2.011342	1.643012
89.H	7.007509	-1.661998	2.386205
90.H	3.587215	6.045051	2.596235
91.H	0.932749	-0.888740	2.004474
92.H	-1.053864	-2.140086	2.392586
93.H	1.916897	5.524228	2.874897
94.H	0.845287	3.390800	2.719004
95.H	-1.472504	-6.355216	3.259007
96.H	1.951363	1.988752	2.849205
97.H	-1.260307	0.034293	2.728623
98.H	-4.343635	3.910793	3.137812
99.H	1.631766	-2.274214	2.876981
100.H	3.266076	4.567215	3.523259
101.H	1.722216	-0.638804	3.599261
102.H	-1.278959	-3.998789	4.056252
103.H	3.899559	-2.951076	3.867787
104.H	5.499863	-2.197730	3.988924
105.H	4.076310	-1.353684	4.623175
106.H	-1.858747	0.916516	5.001869
107.H	-3.407160	2.864179	5.200691
108.I	1.745375	0.708636	-3.997061
109.N	0.745699	-2.076776	-1.128569
110.N	3.329346	3.581922	-0.647223
111.N	-0.930637	1.958269	-0.733015
112.N	4.437399	-0.434729	0.463117
113.N	2.246933	3.224989	1.161734
114.N	3.011119	-0.972784	1.961653
115.P	-0.767547	-2.052771	-0.508654
116.P	-1.884970	0.850447	0.014854
117.Pr	1.046354	0.414187	-0.941047
118.Si	1.811203	-3.388966	-1.585009
119.Si	-1.189146	3.646221	-1.145206

Energy: −702.24817279 eV

Table S10. Coordinates and final energy for a single point energy calculation on geometry optimized 2Sm

1.C	-3.694983	-1.730802	-3.821910

2.C	2.981371	-3.085039	-3.711311
3.C	1.664992	-2.617200	-3.628168
4.C	-3.720760	-0.365901	-3.513013
5.C	0.436235	1.909090	-3.190460
6.C	-3.093087	-2.633048	-2.932961
7.C	3.877876	-2.825578	-2.665333
8.C	1.249758	-1.888998	-2.507574
9.C	0.832561	4.166310	-2.504793
10.C	-3.149966	0.093733	-2.320428
11.C	4.633397	1.435998	-1.802773
12.C	-2.525094	-2.171615	-1.743536
13.C	0.376311	5.203353	-1.505202
14.C	3.459778	-2.098260	-1.549175
15.C	2.142188	-1.618038	-1.462717
16.C	-2.551829	-0.802541	-1.425348
17.C	-0.051971	-0.231578	-0.106472
18.C	-5.028290	1.972511	0.175694
19.C	3.965633	3.208644	0.588211
20.C	0.550477	5.797758	0.799488
21.C	-2.963729	4.095798	0.953528
22.C	5.184299	0.443866	1.050967
23.C	1.992498	-3.056954	1.365421
24.C	-3.728007	-1.732537	1.365453
25.C	2.017783	-1.652873	1.423915
26.C	-2.401441	-1.268452	1.410269
27.C	-4.239221	-2.512916	2.404505
28.C	2.204581	-3.816330	2.518332
29.C	-1.598335	-1.596359	2.513740
30.C	2.249675	-1.023628	2.660151
31.C	-3.622281	1.843565	2.913457
32.C	-3.430815	-2.840920	3.498971
33.C	-2.110424	-2.379630	3.552286
34.C	2.438057	-3.184528	3.744477
35.C	2.457845	-1.785827	3.813296
36.H	-4.144944	-2.091993	-4.747186
37.H	3.307073	-3.655091	-4.582126
38.H	0.958114	-2.824289	-4.432730
39.H	-4.194498	0.339491	-4.197230
40.H	-0.139081	2.181121	-4.089080
41.H	1.504337	1.819322	-3.437835
42.H	0.512385	4.474452	-3.515703
43.H	-3.072595	-3.698451	-3.166474
44.H	4.903123	-3.195281	-2.721019
45.H	0.075405	0.960548	-2.780342
46.H	4.008931	2.068072	-2.454552
47.H	0.219463	-1.534757	-2.427272
48.H	1.932351	4.067672	-2.498357
49.H	4.663142	0.430555	-2.249194
50.H	-3.170091	1.154511	-2.068308
51.H	5.655961	1.846937	-1.823822
52.H	-0.710622	5.382014	-1.588950

53.H	0.904563	6.151956	-1.707689
54.H	-2.061503	-2.880281	-1.054339
55.H	4.162180	-1.913046	-0.735475
56.H	-4.991673	2.333679	-0.863436
57.H	3.342058	3.863016	-0.043206
58.H	-2.807956	4.408436	-0.091680
59.H	-5.309014	0.908727	0.149614
60.H	4.988481	3.617990	0.572061
61.H	1.304216	6.577849	0.609960
62.H	-0.460079	6.232606	0.752783
63.H	-5.838561	2.514613	0.689377
64.H	1.804523	-3.559939	0.414498
65.H	5.268940	-0.612970	0.756656
66.H	-4.363546	-1.490684	0.511187
67.H	6.189223	0.890018	0.974324
68.H	-3.780629	4.708507	1.366982
69.H	3.584732	3.282534	1.619109
70.H	-2.058818	4.332665	1.537791
71.H	0.713497	5.337860	1.781173
72.H	4.885465	0.466337	2.109384
73.H	2.184381	-4.906721	2.459147
74.H	-5.269753	-2.870933	2.357245
75.H	-0.567665	-1.243233	2.556325
76.H	2.246941	0.065247	2.722076
77.H	-3.918344	0.794194	3.066078
78.H	-4.403077	2.484706	3.353558
79.H	-2.685366	2.014658	3.466265
80.H	-3.827015	-3.457673	4.307410
81.H	-1.473153	-2.632843	4.399549
82.H	2.601920	-3.778968	4.645363
83.H	2.631662	-1.283321	4.765034
84.I	0.669140	2.811634	3.351321
85.N	2.327026	0.841455	0.058086
86.N	-2.051956	1.398848	0.348842
87.O	0.233889	2.905175	-2.161258
88.O	0.688613	4.740500	-0.180377
89.P	1.583781	-0.609202	-0.023831
90.P	-1.714682	-0.180869	0.097976
91.Si	3.969060	1.418034	-0.024852
92.Si	-3.386807	2.250452	1.087760
93.Sm	0.280593	2.140418	0.343097

Energy: –547.07421170 eV

Table S11. Coordinates and final energy for a single point energy calculation on geometry optimized 3Sm

1.C	2.832862	2.850774	-4.705541
2.C	-3.956756	1.544613	-4.583782
3.C	-2.922924	0.618696	-4.405572

4.C	-4.442320	2.258789	-3.481694
5.C	2.806948	2.809235	-3.213438
6.C	-2.370447	0.422349	-3.135325
7.C	1.125619	0.941158	-3.099800
8.C	0.209504	-3.988385	-2.841989
9.C	4.419100	4.706087	-2.452365
10.C	0.304423	-5.304653	-2.377809
11.C	3.465885	3.569992	-2.282798
12.C	-3.882728	2.062548	-2.217044
13.C	-0.928084	4.424451	-2.069978
14.C	-2.826888	1.153741	-2.029874
15.C	-0.156123	-2.961844	-1.964272
16.C	0.027329	-5.585130	-1.033881
17.C	2.154495	2.062343	-1.132819
18.C	3.183523	-1.541257	-0.916714
19.C	6.091324	-2.263567	-0.432701
20.C	-0.426821	-3.227688	-0.614235
21.C	-0.970133	-0.399408	-0.371630
22.C	-0.333055	-4.555181	-0.161382
23.C	-2.926984	4.663690	0.237849
24.C	3.556264	3.609159	0.229874
25.C	5.328968	-1.264721	0.373813
26.C	-4.646266	0.251251	0.432141
27.C	0.031547	4.895688	0.770925
28.C	-3.444356	0.899272	0.764676
29.C	-3.252517	-3.315370	0.777497
30.C	3.492634	-0.032492	1.032692
31.C	-5.711861	0.230184	1.336177
32.C	5.712442	-0.501870	1.446346
33.C	-4.386167	-3.756635	1.466059
34.C	-2.331430	-2.454380	1.399930
35.C	7.020478	-0.410910	2.160297
36.C	2.511859	-3.731742	1.949326
37.C	-3.322171	1.504783	2.026673
38.C	-5.586380	0.846901	2.586194
39.C	-4.619739	-3.333484	2.779308
40.C	-2.586264	-2.018654	2.710884
41.C	4.571162	1.154465	2.951029
42.C	-4.386441	1.477822	2.931796
43.C	-3.721317	-2.456499	3.396389
44.C	0.031742	-3.833159	3.688841
45.C	2.035065	-1.615088	4.089069
46.H	-4.391490	1.700556	-5.572827
47.H	3.493948	3.653981	-5.052066
48.H	-2.554807	0.042005	-5.256532
49.H	1.833444	3.038281	-5.126490
50.H	3.202903	1.907406	-5.137101
51.H	0.410793	-3.763491	-3.891356
52.H	4.586300	4.903893	-3.517920
53.H	-5.262126	2.968029	-3.605856
54.H	0.393505	1.451784	-3.737420

55.H	1.687996	0.215514	-3.704124
56.H	0.583510	-6.110040	-3.059926
57.H	-1.586910	-0.318657	-2.976569
58.H	-1.624155	3.923814	-2.760528
59.H	5.398523	4.497262	-1.995107
60.H	-1.048809	5.513071	-2.199232
61.H	0.583976	0.418906	-2.300482
62.H	0.099769	4.163630	-2.370239
63.H	4.032794	5.633214	-2.001436
64.H	-0.249700	-1.931257	-2.308637
65.H	3.339088	-1.003191	-1.861548
66.H	6.138045	-1.987499	-1.497316
67.H	-4.279471	2.611398	-1.363542
68.H	3.446957	-2.595817	-1.050867
69.H	0.090728	-6.610119	-0.664605
70.H	-3.735822	4.394640	-0.456706
71.H	2.127222	-1.499424	-0.631167
72.H	5.641915	-3.266555	-0.368715
73.H	-4.756921	-0.228232	-0.541808
74.H	7.121201	-2.340744	-0.064203
75.H	-3.078390	-3.656135	-0.244407
76.H	-2.856494	5.764253	0.239668
77.H	3.457476	4.701119	0.263740
78.H	4.612018	3.337483	0.364607
79.H	1.061486	4.667130	0.461018
80.H	-0.125626	5.977105	0.622080
81.H	-0.552852	-4.789832	0.880054
82.H	2.969390	3.185955	1.050877
83.H	-3.231371	4.346168	1.246080
84.H	-5.085234	-4.437017	0.976802
85.H	-6.642432	-0.269409	1.062030
86.H	2.127359	-4.304111	1.091688
87.H	7.749000	-1.093149	1.706017
88.H	7.444511	0.604534	2.118399
89.H	3.364967	-3.126940	1.606344
90.H	-0.049751	4.676384	1.845454
91.H	-2.390513	2.007587	2.290194
92.H	2.893076	-4.452682	2.691620
93.H	5.251921	1.997741	2.770688
94.H	6.926892	-0.685134	3.222360
95.H	3.551389	1.529941	3.096108
96.H	-0.581647	-4.478578	3.042734
97.H	-5.500989	-3.683889	3.319689
98.H	-6.421879	0.833163	3.289017
99.H	-1.880183	-1.345819	3.197525
100.H	2.847330	-1.005155	3.668256
101.H	4.883310	0.637395	3.867246
102.H	-4.277532	1.957993	3.905875
103.H	-3.901110	-2.114831	4.416712
104.H	0.662355	-4.490295	4.311312
105.H	-0.656951	-3.300676	4.360606

106.H	1.341996	-0.931876	4.601736
107.H	2.475162	-2.292189	4.840178
108.I	0.842737	2.277880	3.655985
109.N	2.024140	1.905638	-2.484893
110.N	3.051665	3.091280	-1.033709
111.N	-1.064522	2.227215	0.013142
112.N	3.982728	-0.956908	0.150138
113.N	0.386523	-1.575724	1.603387
114.N	4.581097	0.234960	1.820599
115.P	-2.004075	0.961424	-0.381221
116.P	-0.829733	-1.832502	0.538503
117.Si	-1.237849	3.946622	-0.259515
118.Si	1.179817	-2.641188	2.755861
119.Sm	0.828850	0.799954	0.895922

Energy: −704.62360827 eV

Table S12. Computed bond, indices, charges, and spin densities of 2M and 3M (M = U, Np, Pu, Ce, Pr, Sm).

**Cmpd**	**Bond**	**M-C bond lengths and indices**			**MDC_q charges**		**MDC_m spin density**	
		**Expt.**	**Calc.**	**BI[a]**	**M**	**C**	**M**	**C**
**2U**[b]	$U{=}C_{BIPM}$	-	2.4232	1.28	1.57	−2.00	3.28	−0.04
**3U**[b]	$U{=}C_{BIPM}$	-	2.4618	1.17	1.62	−1.67	3.08	−0.04
	$U{\leftarrow}C_{NHC}$	-	2.7153	0.77		−0.50		−0.03
	$U{\leftarrow}C_{NHC}$	-	2.6538	0.81		−0.48		−0.03
**2Np**[b]	$Np{=}C_{BIPM}$	2.425(7)	2.3899	1.40	1.54	−1.96	4.36	−0.07
**3Np**[b]	$Np{=}C_{BIPM}$	2.490(6)	2.4700	1.20	1.51	−1.64	4.22	−0.05
	$Np{\leftarrow}C_{NHC}$	2.677(5)	2.6719	0.69		−0.46		−0.03
	$Np{\leftarrow}C_{NHC}$	2.751(6)	2.7321	0.65		−0.44		−0.03
**2Pu**	$Pu{=}C_{BIPM}$	2.422(6)	2.3916	0.79	1.48	−1.94	5.40	−0.13
**3Pu**	$Pu{=}C_{BIPM}$	2.477(4)	2.4854	0.59	1.38	−1.60	5.26	−0.05
	$Pu{\leftarrow}C_{NHC}$	2.663(5)	2.6945	0.33		−0.41		−0.03
	$Pu{\leftarrow}C_{NHC}$	2.739(4)	2.7608	0.28		−0.40		−0.03
**2Ce**[b]	$Ce{=}C_{BIPM}$	2.4772(19)	2.4402	1.05	1.32	−1.82	1.07	−0.01
**3Ce**[b]	$Ce{=}C_{BIPM}$	2.519(2)	2.4880	0.96	1.29	−1.53	1.01	−0.01
	$Ce{\leftarrow}C_{NHC}$	2.737(3)	2.7576	0.52		−0.38		−0.01
	$Ce{\leftarrow}C_{NHC}$	2.806(2)	2.8207	0.58		−0.36		−0.01
**2Pr**	$Pr{=}C_{BIPM}$	2.448(5)	2.4123	0.80	1.30	−1.73	2.15	−0.06
**3Pr**	$Pr{=}C_{BIPM}$	2.492(3)	2.4800	0.68	1.19	−1.51	2.13	−0.04
	$Pr{\leftarrow}C_{NHC}$	2.723(3)	2.7605	0.35		−0.32		−0.01
	$Pr{\leftarrow}C_{NHC}$	2.784(3)	2.8118	0.31		−0.33		−0.01
**2Sm**	$Sm{=}C_{BIPM}$	2.381(4)	2.4370	0.83	1.17	−1.63	5.56	−0.35
**3Sm**	$Sm{=}C_{BIPM}$	2.444(3)	2.5063	0.71	1.06	−1.42	5.50	−0.28
	$Sm{\leftarrow}C_{NHC}$	2.667(3)	2.7325	0.26		−0.28		−0.03
	$Sm{\leftarrow}C_{NHC}$	2.738(3)	2.7942	0.23		−0.30		−0.02

[a] Nalewajski-Mrozek bond indices. [b] Reported previously,[42] reproduced here for comparative purposes (in previous work, **2M** (here) = **5M** and **3M** (here) = **6M**).

Table S13. Computed NBO data for 2M and 3M (M = U, Np, Pu, Ce, Pr, Sm).

Cmpd	Bond	M-C σ-bond (%)				M-C π-bond (%)			
		M[a]	C	M s/p/d/f	C s/p	M	C	M s/p/d/f	C s/p
**2U**[b]	U=C_{BIPM}	14	86	4/1/42/53	14/86	13	87	0/0/40/60	0/100
**3U**[b]	U=C_{BIPM}	14	86	10/1/46/43	24/76	10	90	0/1/50/49	1/99
	U←C_{NHC}	0	100		46/54				
	U←C_{NHC}	0	100		46/54				
**2Np**[b]	Np=C_{BIPM}	17	83	4/1/32/63	13/87	14	86	0/0/38/62	0/100
**3Np**[b]	Np=C_{BIPM}	15	85	9/1/39/51	22/78	10	90	0/1/43/56	1/99
	Np←C_{NHC}	0	100		46/54				
	Np←C_{NHC}	0	100		46/54				
**2Pu**	Pu=C_{BIPM}	20	80	3/1/26/70	11/89	15	85	0/0/36/64	0/100
**3Pu**	Pu=C_{BIPM}	15	85	10/1/36/53	20/80	10	90	1/1/39/59	3/97
	Pu←C_{NHC}	0	100		46/54				
	Pu←C_{NHC}	0	100		46/54				
**2Ce**[b]	Ce=C_{BIPM}	10	90	1/1/61/37	8/92	8	92	0/0/65/35	0/100
**3Ce**[b]	Ce=C_{BIPM}	9	91	7/1/65/27	20/80	7	93	2/1/60/37	2/98
	Ce←C_{NHC}	0	100		46/54				
	Ce←C_{NHC}	0	100		46/54				
**2Pr**	Pr=C_{BIPM}	12	88	1/1/48/50	8/92	10	90	0/0/49/51	1/99
**3Pr**	Pr=C_{BIPM}	10	90	7/1/57/35	20/80	8	92	3/1/56/40	2/98
	Pr←C_{NHC}	0	100		46/54				
	Pr←C_{NHC}	0	100		46/54				
**2Sm**	Sm=C_{BIPM}	23	77	1/1/18/80	5/95	25	75	0/0/16/84	2/98
**3Sm**	Sm=C_{BIPM}	17	83	5/1/27/67	14/86	22	78	2/0/14/84	6/94
	Sm←C_{NHC}	0	100		46/54				
	Sm←C_{NHC}	0	100		46/54				

[a] The NBO cut-off is 5%, so M% = 0 means only that the M contribution to that bond is <5%. [b] Reported previously,[42] reproduced here for comparative purposes (in previous work, **2M** (here) = **5M** and **3M** (here) = **6M**).

Table S14. Computed QTAIM data for 2M and 3M (M = U, Np, Pu, Ce, Pr, Sm).

Cmpd	Bond	ρ[a]	$\nabla^2\rho$[b]	H[c]	ε[d]
**2U**[e]	U=C_{BIPM}	0.08	0.12	–0.03	0.20
**3U**[e]	U=C_{BIPM}	0.08	0.11	–0.03	0.17
	U←C_{NHC}	0.05	0.10	–0.01	0.03
	U←C_{NHC}	0.05	0.11	–0.01	0.03
**2Np**[e]	Np=C_{BIPM}	0.08	0.13	–0.04	0.21
**3Np**[e]	Np=C_{BIPM}	0.08	0.12	–0.03	0.18
	Np←C_{NHC}	0.04	0.11	–0.01	0.03
	Np←C_{NHC}	0.05	0.11	–0.01	0.03
**2Pu**	Pu=C_{BIPM}	0.08	0.13	–0.04	0.10
**3Pu**	Pu=C_{BIPM}	0.07	0.12	–0.02	0.10
	Pu←C_{NHC}	0.05	0.10	–0.01	0.02
	Pu←C_{NHC}	0.04	0.09	–0.01	0.02
**2Ce**[e]	Ce=C_{BIPM}	0.07	0.12	–0.03	0.22
**3Ce**[e]	Ce=C_{BIPM}	0.07	0.10	–0.02	0.19
	Ce←C_{NHC}	0.04	0.08	–0.01	0.03
	Ce←C_{NHC}	0.04	0.08	–0.01	0.03
**2Pr**	Pr=C_{BIPM}	0.07	0.12	–0.03	0.22
**3Pr**	Pr=C_{BIPM}	0.07	0.11	–0.02	0.21
	Pr←C_{NHC}	0.04	0.08	–0.06	0.08
	Pr←C_{NHC}	0.04	0.07	–0.04	0.02
**2Sm**	Sm=C_{BIPM}	0.07	0.12	–0.02	0.16
**3Sm**	Sm=C_{BIPM}	0.06	0.11	–0.02	0.14
	Sm←C_{NHC}	0.04	0.09	–0.05	0.02
	Sm←C_{NHC}	0.03	0.08	–0.03	0.04

[a] Topological electron density. [b] Laplacian. [c] Electronic energy density. [d] Bond ellipticity. [e] Reported previously,[42] reproduced here for comparative purposes (in previous work, **2M** (here) = **5M** and **3M** (here) = **6M**)

Table S15. State energies and composition of Pr complexes computed with CASSCF-SO.

2Pr			3Pr		
State	Energy (cm^{-1})	Composition	Energy (cm^{-1})	State	Composition
1	0	0.96|$^3H_4$>	1	0	0.96|$^3H_4$>
2	10.4	0.96|$^3H_4$>	2	34.7	0.95|$^3H_4$>
3	220.7	0.96|$^3H_4$>	3	414.6	0.96|$^3H_4$>
4	301.0	0.96|$^3H_4$>	4	444.5	0.96|$^3H_4$>
5	414.1	0.96|$^3H_4$>	5	600.8	0.95|$^3H_4$>
6	438.1	0.95|$^3H_4$>	6	637.8	0.96|$^3H_4$>
7	494.4	0.96|$^3H_4$>	7	668.9	0.96|$^3H_4$>
8	572.8	0.96|$^3H_4$>	8	877.6	0.95|$^3H_4$>
9	612.3	0.95|$^3H_4$>	9	930.7	0.95|$^3H_4$>
10	2255.0	0.98|$^3H_5$>	10	2292.5	0.97|$^3H_5$>
11	2303.9	0.97|$^3H_5$>	11	2311.2	0.97|$^3H_5$>
12	2468.6	0.98|$^3H_5$>	12	2595.1	0.97|$^3H_5$>
13	2533.1	0.98|$^3H_5$>	13	2646.7	0.97|$^3H_5$>
14	2592.5	0.98|$^3H_5$>	14	2784.6	0.98|$^3H_5$>
15	2652.8	0.97|$^3H_5$>	15	2823.3	0.97|$^3H_5$>
16	2702.5	0.98|$^3H_5$>	16	2870.6	0.98|$^3H_5$>
17	2769.0	0.98|$^3H_5$>	17	2959.1	0.98|$^3H_5$>
18	2786.3	0.98|$^3H_5$>	18	2992.4	0.98|$^3H_5$>
19	2807.5	0.98|$^3H_5$>	19	3152.5	0.97|$^3H_5$>
20	2845.3	0.98|$^3H_5$>	20	3203.5	0.99|$^3H_5$>
21	4557.4	0.96|$^3H_6$>	21	4589.8	0.97|$^3H_6$>
22	4591.0	0.97|$^3H_6$>	22	4602.0	0.97|$^3H_6$>
23	4816.7	0.97|$^3H_6$>	23	4930.2	0.96|$^3H_6$>
24	4855.6	0.98|$^3H_6$>	24	4938.8	0.96|$^3H_6$>
25	4994.2	0.97|$^3H_6$>	25	5130.8	0.94|$^3H_6$>
26	5003.5	0.97|$^3H_6$>	26	5185.4	0.95|$^3H_6$>
27	5058.9	0.97|$^3H_6$>	27	5263.9	0.96|$^3H_6$>
28	5079.6	0.96|$^3H_6$>	28	5316.3	0.96|$^3H_6$>
29	5144.5	0.97|$^3H_6$>	29	5329.5	0.97|$^3H_6$>

30	5227.3	0.97|$^3H_6$>	30	5373.3	0.95|$^3H_6$>
31	5247.1	0.97|$^3H_6$>	31	5408.8	0.97|$^3H_6$>
32	5299.0	0.97|$^3H_6$>	32	5637.5	0.92|$^3H_6$>
33	5352.8	0.97|$^3H_6$>	33	5648.2	0.95|$^3H_6$>
34	5833.1	0.97|$^3F_2$>	34	5994.6	0.96|$^3F_2$>
35	5911.1	0.97|$^3F_2$>	35	6024.1	0.94|$^3F_2$>
36	5958.9	0.96|$^3F_2$>	36	6097.1	0.95|$^3F_2$>
37	5965.5	0.98|$^3F_2$>	37	6197.3	0.93|$^3F_2$>
38	5984.3	0.97|$^3F_2$>	38	6233.4	0.95|$^3F_2$>
39	6973.7	0.96|$^3F_3$>	39	7158.4	0.97|$^3F_3$>
40	7077.4	0.94|$^3F_3$>	40	7194.7	0.95|$^3F_3$>
41	7126.7	0.95|$^3F_3$>	41	7260.8	0.96|$^3F_3$>
42	7145.7	0.96|$^3F_3$>	42	7310.1	0.95|$^3F_3$>
43	7158.5	0.94|$^3F_3$>	43	7324.1	0.90|$^3F_3$>
44	7160.8	0.93|$^3F_3$>	44	7375.3	0.84|$^3F_3$>
45	7223.5	0.95|$^3F_3$>	45	7402.7	0.88|$^3F_3$>
46	7352.6	0.49|$^3F_4$> + 0.42|$^1G_4$>	46	7480.5	0.41|$^1G_4$> + 0.49|$^3F_4$>
47	7383.3	0.55|$^3F_4$> + 0.40|$^1G_4$>	47	7548.6	0.39|$^1G_4$> + 0.53|$^3F_4$>
48	7400.0	0.51|$^3F_4$> + 0.40|$^1G_4$>	48	7602.0	0.37|$^1G_4$> + 0.14|$^3F_3$> + 0.47|$^3F_4$>
49	7485.0	0.53|$^3F_4$> + 0.38|$^1G_4$>	49	7660.7	0.37|$^1G_4$> + 0.53|$^3F_4$>
50	7515.4	0.58|$^3F_4$> + 0.38|$^1G_4$>	50	7712.4	0.36|$^1G_4$> + 0.61|$^3F_4$>
51	7538.3	0.56|$^3F_4$> + 0.40|$^1G_4$>	51	7763.6	0.35|$^1G_4$> + 0.60|$^3F_4$>
52	7572.2	0.59|$^3F_4$> + 0.35|$^1G_4$>	52	7776.9	0.36|$^1G_4$> + 0.56|$^3F_4$>
53	7685.8	0.33|$^1G_4$> + 0.65|$^3F_4$>	53	7803.5	0.34|$^1G_4$> + 0.59|$^3F_4$>
54	7739.5	0.30|$^1G_4$> + 0.66|$^3F_4$>	54	7848.7	0.36|$^1G_4$> + 0.58|$^3F_4$>
55	10332.1	0.52|$^1G_4$> + 0.47|$^3F_4$>	55	10456.8	0.52|$^1G_4$> + 0.46|$^3F_4$>
56	10373.6	0.53|$^1G_4$> + 0.45|$^3F_4$>	56	10520.9	0.54|$^1G_4$> + 0.44|$^3F_4$>
57	10414.3	0.56|$^1G_4$> + 0.43|$^3F_4$>	57	10600.3	0.56|$^1G_4$> + 0.43|$^3F_4$>
58	10471.0	0.57|$^1G_4$> + 0.42|$^3F_4$>	58	10699.1	0.57|$^1G_4$> + 0.42|$^3F_4$>
59	10548.3	0.56|$^1G_4$> + 0.43|$^3F_4$>	59	10755.6	0.59|$^1G_4$> + 0.39|$^3F_4$>
60	10616.5	0.59|$^1G_4$> + 0.40|$^3F_4$>	60	10797.4	0.57|$^1G_4$> + 0.41|$^3F_4$>
61	10679.8	0.61|$^1G_4$> + 0.38|$^3F_4$>	61	10866.1	0.60|$^1G_4$> + 0.38|$^3F_4$>

62	10921.7	$0.65\|^1G_4> + 0.33\|^3F_4>$	62	10959.0	$0.63\|^1G_4> + 0.35\|^3F_4>$
63	10991.8	$0.67\|^1G_4> + 0.32\|^3F_4>$	63	11041.5	$0.65\|^1G_4> + 0.33\|^3F_4>$

Table S16. State energies and composition of Pr complexes computed with MS-CASPT2-SO.

**2Pr**			**3Pr**		
**State**	**Energy (cm^{-1})**	**Composition**	**State**	**Energy (cm^{-1})**	**Composition**
1	0.0	$0.96\|^3H_4>$	1	0.0	$0.96\|^3H_4>$
2	10.4	$0.96\|^3H_4>$	2	34.7	$0.95\|^3H_4>$
3	220.7	$0.96\|^3H_4>$	3	414.6	$0.96\|^3H_4>$
4	301.0	$0.96\|^3H_4>$	4	444.5	$0.96\|^3H_4>$
5	414.1	$0.96\|^3H_4>$	5	600.8	$0.95\|^3H_4>$
6	438.1	$0.95\|^3H_4>$	6	637.8	$0.96\|^3H_4>$
7	494.4	$0.96\|^3H_4>$	7	668.9	$0.96\|^3H_4>$
8	572.8	$0.96\|^3H_4>$	8	877.6	$0.95\|^3H_4>$
9	612.3	$0.95\|^3H_4>$	9	930.7	$0.95\|^3H_4>$
10	2255.0	$0.98\|^3H_5>$	10	2292.5	$0.97\|^3H_5>$
11	2303.9	$0.97\|^3H_5>$	11	2311.2	$0.97\|^3H_5>$
12	2468.6	$0.98\|^3H_5>$	12	2595.1	$0.97\|^3H_5>$
13	2533.1	$0.98\|^3H_5>$	13	2646.7	$0.97\|^3H_5>$
14	2592.5	$0.98\|^3H_5>$	14	2784.6	$0.98\|^3H_5>$
15	2652.8	$0.97\|^3H_5>$	15	2823.3	$0.97\|^3H_5>$
16	2702.5	$0.98\|^3H_5>$	16	2870.6	$0.98\|^3H_5>$
17	2769.0	$0.98\|^3H_5>$	17	2959.1	$0.98\|^3H_5>$
18	2786.3	$0.98\|^3H_5>$	18	2992.4	$0.98\|^3H_5>$
19	2807.5	$0.98\|^3H_5>$	19	3152.5	$0.97\|^3H_5>$
20	2845.3	$0.98\|^3H_5>$	20	3203.5	$0.99\|^3H_5>$
21	4557.4	$0.96\|^3H_6>$	21	4589.8	$0.97\|^3H_6>$
22	4591.0	$0.97\|^3H_6>$	22	4602.0	$0.97\|^3H_6>$
23	4816.7	$0.97\|^3H_6>$	23	4930.2	$0.96\|^3H_6>$
24	4855.6	$0.98\|^3H_6>$	24	4938.8	$0.96\|^3H_6>$
25	4994.2	$0.97\|^3H_6>$	25	5130.8	$0.94\|^3H_6>$

26	5003.5	0.97|$^3H_6$>	26	5185.4	0.95|$^3H_6$>
27	5058.9	0.97|$^3H_6$>	27	5263.9	0.96|$^3H_6$>
28	5079.6	0.96|$^3H_6$>	28	5316.3	0.96|$^3H_6$>
29	5144.5	0.97|$^3H_6$>	29	5329.5	0.97|$^3H_6$>
30	5227.3	0.97|$^3H_6$>	30	5373.3	0.95|$^3H_6$>
31	5247.1	0.97|$^3H_6$>	31	5408.8	0.97|$^3H_6$>
32	5299.0	0.97|$^3H_6$>	32	5637.5	0.92|$^3H_6$>
33	5352.8	0.97|$^3H_6$>	33	5648.2	0.95|$^3H_6$>
34	5833.1	0.97|$^3F_2$>	34	5994.6	0.96|$^3F_2$>
35	5911.1	0.97|$^3F_2$>	35	6024.1	0.94|$^3F_2$>
36	5958.9	0.96|$^3F_2$>	36	6097.1	0.95|$^3F_2$>
37	5965.5	0.98|$^3F_2$>	37	6197.3	0.93|$^3F_2$>
38	5984.3	0.97|$^3F_2$>	38	6233.4	0.95|$^3F_2$>
39	6973.7	0.96|$^3F_3$>	39	7158.4	0.97|$^3F_3$>
40	7077.4	0.94|$^3F_3$>	40	7194.7	0.95|$^3F_3$>
41	7126.7	0.95|$^3F_3$>	41	7260.8	0.96|$^3F_3$>
42	7145.7	0.96|$^3F_3$>	42	7310.1	0.95|$^3F_3$>
43	7158.5	0.94|$^3F_3$>	43	7324.1	0.90|$^3F_3$>
44	7160.8	0.93|$^3F_3$>	44	7375.3	0.84|$^3F_3$>
45	7223.5	0.95|$^3F_3$>	45	7402.7	0.88|$^3F_3$>
46	7352.6	0.49|$^3F_4$> + 0.42|$^1G_4$>	46	7480.5	0.49|$^3F_4$> + 0.41|$^1G_4$>
47	7383.3	0.55|$^3F_4$> + 0.40|$^1G_4$>	47	7548.6	0.53|$^3F_4$> + 0.39|$^1G_4$>
48	7400.0	0.51|$^3F_4$> + 0.40|$^1G_4$>	48	7602.0	0.47|$^3F_4$> + 0.37|$^1G_4$> + 0.14|$^3F_3$>
49	7485.0	0.53|$^3F_4$> + 0.38|$^1G_4$>	49	7660.7	0.53|$^3F_4$> + 0.37|$^1G_4$>
50	7515.4	0.58|$^3F_4$> + 0.38|$^1G_4$>	50	7712.4	0.61|$^3F_4$> + 0.36|$^1G_4$>
51	7538.3	0.56|$^3F_4$> + 0.40|$^1G_4$>	51	7763.6	0.60|$^3F_4$> + 0.35|$^1G_4$>
52	7572.2	0.59|$^3F_4$> + 0.35|$^1G_4$>	52	7776.9	0.56|$^3F_4$> + 0.36|$^1G_4$>
53	7685.8	0.65|$^3F_4$> + 0.33|$^1G_4$>	53	7803.5	0.59|$^3F_4$> + 0.34|$^1G_4$>
54	7739.5	0.66|$^3F_4$> + 0.30|$^1G_4$>	54	7848.7	0.58|$^3F_4$> + 0.36|$^1G_4$>
55	10332.1	0.52|$^1G_4$> + 0.47|$^3F_4$>	55	10456.8	0.52|$^1G_4$> + 0.46|$^3F_4$>
56	10373.6	0.53|$^1G_4$> + 0.45|$^3F_4$>	56	10521.0	0.54|$^1G_4$> + 0.44|$^3F_4$>
57	10414.3	0.56|$^1G_4$> + 0.43|$^3F_4$>	57	10600.3	0.56|$^1G_4$> + 0.43|$^3F_4$>

58	10471.0	0.57|$^1G_4$> + 0.42|$^3F_4$>	58	10699.1	0.57|$^1G_4$> + 0.42|$^3F_4$>
59	10548.3	0.56|$^1G_4$> + 0.43|$^3F_4$>	59	10755.6	0.59|$^1G_4$> + 0.39|$^3F_4$>
60	10616.5	0.59|$^1G_4$> + 0.40|$^3F_4$>	60	10797.4	0.57|$^1G_4$> + 0.41|$^3F_4$>
61	10679.8	0.61|$^1G_4$> + 0.38|$^3F_4$>	61	10866.1	0.60|$^1G_4$> + 0.38|$^3F_4$>
62	10921.7	0.65|$^1G_4$> + 0.33|$^3F_4$>	62	10959.0	0.63|$^1G_4$> + 0.35|$^3F_4$>
63	10991.8	0.67|$^1G_4$> + 0.32|$^3F_4$>	63	11041.5	0.65|$^1G_4$> + 0.33|$^3F_4$>

Table S17. State energies and composition Pr complexes computed with XMS-CASPT2 -SO.

**2Pr**			**3Pr**		
**State**	**Energy (cm^{-1})**	**Composition**	**State**	**Energy (cm^{-1})**	**Composition**
1	0.0	0.96|$^3H_4$>	1	0.0	0.96|$^3H_4$>
2	79.3	0.95|$^3H_4$>	2	37.3	0.95|$^3H_4$>
3	184.3	0.95|$^3H_4$>	3	330.9	0.96|$^3H_4$>
4	324.5	0.95|$^3H_4$>	4	428.5	0.95|$^3H_4$>
5	440.3	0.95|$^3H_4$>	5	514.7	0.95|$^3H_4$>
6	523.6	0.94|$^3H_4$>	6	568.0	0.93|$^3H_4$>
7	566.3	0.94|$^3H_4$>	7	702.8	0.95|$^3H_4$>
8	736.1	0.94|$^3H_4$>	8	921.1	0.94|$^3H_4$>
9	844.5	0.93|$^3H_4$>	9	942.3	0.94|$^3H_4$>
10	2288.0	0.97|$^3H_5$>	10	2220.4	0.95|$^3H_5$>
11	2343.7	0.96|$^3H_5$>	11	2430.4	0.97|$^3H_5$>
12	2388.7	0.97|$^3H_5$>	12	2536.3	0.97|$^3H_5$>
13	2550.7	0.97|$^3H_5$>	13	2608.8	0.96|$^3H_5$>
14	2656.5	0.95|$^3H_5$>	14	2660.3	0.96|$^3H_5$>
15	2717.2	0.96|$^3H_5$>	15	2762.2	0.97|$^3H_5$>
16	2740.9	0.95|$^3H_5$>	16	2912.7	0.97|$^3H_5$>
17	2819.4	0.94|$^3H_5$>	17	2951.0	0.96|$^3H_5$>
18	2938.6	0.96|$^3H_5$>	18	3052.1	0.97|$^3H_5$>
19	2989.8	0.96|$^3H_5$>	19	3189.3	0.97|$^3H_5$>
20	3228.4	0.94|$^3H_5$>	20	3218.1	0.96|$^3H_5$>

21	4596.2	0.96|$^3H_6$>	21	4629.8	0.97|$^3H_6$>
22	4645.7	0.96|$^3H_6$>	22	4663.5	0.96|$^3H_6$>
23	4731.6	0.94|$^3H_6$>	23	4873.4	0.97|$^3H_6$>
24	4792.5	0.96|$^3H_6$>	24	4897.2	0.96|$^3H_6$>
25	4946.7	0.92|$^3H_6$>	25	5074.7	0.94|$^3H_6$>
26	5030.7	0.93|$^3H_6$>	26	5119.0	0.96|$^3H_6$>
27	5132.7	0.94|$^3H_6$>	27	5260.8	0.95|$^3H_6$>
28	5180.2	0.95|$^3H_6$>	28	5279.1	0.97|$^3H_6$>
29	5290.9	0.86|$^3H_6$>	29	5384.4	0.94|$^3H_6$>
30	5390.0	0.92|$^3H_6$>	30	5417.5	0.94|$^3H_6$>
31	5492.1	0.91|$^3H_6$>	31	5483.4	0.96|$^3H_6$>
32	5549.0	0.90|$^3H_6$>	32	5656.7	0.93|$^3H_6$>
33	5595.9	0.88|$^3H_6$>	33	5682.4	0.95|$^3H_6$>
34	6187.0	0.90|$^3F_2$>	34	6307.0	0.94|$^3F_2$>
35	6240.2	0.85|$^3F_2$>	35	6426.5	0.91|$^3F_2$>
36	6308.3	0.91|$^3F_2$>	36	6538.7	0.95|$^3F_2$>
37	6466.3	0.89|$^3F_2$>	37	6555.9	0.90|$^3F_2$>
38	6514.9	0.89|$^3F_2$>	38	6744.0	0.91|$^3F_2$>
39	7246.5	0.74|$^3F_3$> + 0.10|$^3F_4$>	39	7387.2	0.77|$^3F_3$>
40	7327.8	0.82|$^3F_3$>	40	7506.7	0.78|$^3F_3$> + 0.10|$^3F_4$>
41	7386.9	0.84|$^3F_3$>	41	7590.9	0.61|$^3F_3$> + 0.17|$^3F_4$> + 0.16|$^1G_4$>
42	7492.6	0.52|$^3F_3$> + 0.22|$^3F_4$> + 0.18|$^1G_4$>	42	7653.4	0.69|$^3F_3$> + 0.14|$^3F_4$> + 0.11|$^1G_4$>
43	7552.4	0.52|$^3F_3$> + 0.26|$^3F_4$> + 0.18|$^1G_4$>	43	7719.7	0.52|$^3F_3$> + 0.24|$^1G_4$> + 0.19|$^3F_4$>
44	7573.0	0.37|$^3F_3$> + 0.32|$^1G_4$> + 0.27|$^3F_4$>	44	7729.5	0.39|$^1G_4$> + 0.34|$^3F_4$>+ 0.21|$^3F_3$>
45	7638.5	0.51|$^3F_3$> + 0.25|$^3F_4$> + 0.20|$^1G_4$>	45	7824.5	0.38|$^3F_3$> + 0.30|$^1G_4$> + 0.28|$^3F_4$>
46	7686.1	0.71|$^3F_3$> + 0.13|$^1G_4$> + 0.11|$^3F_4$>	46	7865.4	0.34|$^3F_3$> + 0.31|$^1G_4$> + 0.30|$^3F_4$>
47	7724.9	0.42|$^1G_4$> + 0.40|$^3F_4$> + 0.13|$^3F_3$>	47	7876.2	0.36|$^3F_3$> + 0.32|$^1G_4$> + 0.28|$^3F_4$>
48	7759.6	0.61|$^3F_3$> + 0.18|$^1G_4$> + 0.15|$^3F_4$>	48	7975.2	0.65|$^3F_3$> + 0.16|$^1G_4$> + 0.14|$^3F_4$>
49	7794.3	0.44|$^3F_4$> + 0.37|$^1G_4$> + 0.16|$^3F_3$>	49	8003.9	0.38|$^3F_4$> + 0.32|$^1G_4$> + 0.26|$^3F_3$>
50	7894.6	0.38|$^3F_3$> + 0.31|$^3F_4$> + 0.27|$^1G_4$>	50	8030.8	0.43|$^3F_3$> + 0.29|$^3F_4$> + 0.24|$^1G_4$>
51	7927.6	0.49|$^3F_4$> + 0.38|$^1G_4$>	51	8078.3	0.48|$^3F_4$> + 0.34|$^1G_4$> + 0.15|$^3F_3$>
52	7958.6	0.44|$^3F_4$> + 0.33|$^1G_4$> + 0.21|$^3F_3$>	52	8256.4	0.38|$^3F_4$> + 0.34|$^1G_4$> + 0.25|$^3F_3$>

53	8053.9	0.52|$^3F_4$> + 0.33|$^1G_4$> + 0.13|$^3F_3$>	53	8290.9	0.50|$^3F_4$> + 0.39|$^1G_4$>
54	8150.6	0.57|$^3F_4$> + 0.35|$^1G_4$>	54	8305.7	0.35|$^3F_3$> + 0.32|$^3F_4$> + 0.30|$^1G_4$>
55	10518.3	0.51|$^3F_4$> + 0.48|$^1G_4$>	55	10678.5	0.46|$^1G_4$> + 0.53|$^3F_4$>
56	10601.1	0.49|$^1G_4$> + 0.49|$^3F_4$>	56	10745.9	0.54|$^3F_4$> + 0.44|$^1G_4$>
57	10672.5	0.50|$^1G_4$> + 0.48|$^3F_4$>	57	10760.3	0.52|$^3F_4$> + 0.46|$^1G_4$>
58	10703.3	0.51|$^1G_4$> + 0.48|$^3F_4$>	58	10828.2	0.50|$^1G_4$> + 0.48|$^3F_4$>
59	10767.5	0.53|$^1G_4$> + 0.46|$^3F_4$>	59	10990.8	0.54|$^1G_4$> + 0.45|$^3F_4$>
60	10864.8	0.55|$^1G_4$> + 0.43|$^3F_4$>	60	11105.8	0.52|$^1G_4$> + 0.46|$^3F_4$>
61	11045.1	0.59|$^1G_4$> + 0.39|$^3F_4$>	61	11138.9	0.57|$^1G_4$> + 0.42|$^3F_4$>
62	11163.6	0.61|$^1G_4$> + 0.37|$^3F_4$>	62	11303.6	0.57|$^1G_4$> + 0.41|$^3F_4$>
63	11300.0	0.62|$^1G_4$> + 0.36|$^3F_4$>	63	11395.0	0.58|$^1G_4$> + 0.40|$^3F_4$>

Table S18. State energies and composition of the Sm complexes computed with CASSCF-SO.

2Sm			3Sm		
**State**	**Energy (cm^{-1})**	**Composition**	**State**	**Energy (cm^{-1})**	**Composition**
1	0	0.99|$^6H_{5/2}$>	1	0.0	0.97|$^6H_{5/2}$>
2	0	0.99|$^6H_{5/2}$>	2	0.0	0.97|$^6H_{5/2}$>
3	200.6	0.96|$^6H_{5/2}$>	3	282.4	0.98|$^6H_{5/2}$>
4	200.6	0.96|$^6H_{5/2}$>	4	282.4	0.98|$^6H_{5/2}$>
5	406.9	0.96|$^6H_{5/2}$>	5	537.8	0.92|$^6H_{5/2}$>
6	406.9	0.96|$^6H_{5/2}$>	6	537.8	0.92|$^6H_{5/2}$>
7	866.6	0.98|$^6H_{7/2}$>	7	914.6	0.97|$^6H_{7/2}$>
8	866.6	0.98|$^6H_{7/2}$>	8	914.6	0.97|$^6H_{7/2}$>
9	1106.5	0.96|$^6H_{7/2}$>	9	1162.9	0.92|$^6H_{7/2}$>
10	1106.5	0.96|$^6H_{7/2}$>	10	1162.9	0.92|$^6H_{7/2}$>
11	1197.2	0.94|$^6H_{7/2}$>	11	1277.6	0.90|$^6H_{7/2}$>
12	1197.2	0.94|$^6H_{7/2}$>	12	1277.6	0.90|$^6H_{7/2}$>
13	1261.6	0.96|$^6H_{7/2}$>	13	1393.3	0.95|$^6H_{7/2}$>
14	1261.6	0.96|$^6H_{7/2}$>	14	1393.3	0.95|$^6H_{7/2}$>

15	2027.6	$0.98\|^6H_{7/2}>$	15	2062.2	$0.98\|^6H_{7/2}>$
16	2027.6	$0.98\|^6H_{7/2}>$	16	2062.2	$0.98\|^6H_{7/2}>$
17	2135.9	$0.96\|^6H_{7/2}>$	17	2214.5	$0.94\|^6H_{7/2}>$
18	2135.9	$0.96\|^6H_{7/2}>$	18	2214.5	$0.94\|^6H_{7/2}>$
19	2289.3	$0.97\|^6H_{7/2}>$	19	2359.6	$0.95\|^6H_{7/2}>$
20	2289.3	$0.97\|^6H_{7/2}>$	20	2359.6	$0.95\|^6H_{7/2}>$
21	2343.8	$0.97\|^6H_{7/2}>$	21	2409.5	$0.95\|^6H_{7/2}>$
22	2343.8	$0.97\|^6H_{7/2}>$	22	2409.5	$0.95\|^6H_{7/2}>$
23	2388.6	$0.97\|^6H_{7/2}>$	23	2546.9	$0.97\|^6H_{7/2}>$
24	2388.6	$0.97\|^6H_{7/2}>$	24	2546.9	$0.97\|^6H_{7/2}>$
25	3377.2	$0.99\|^6H_{11/2}>$	25	3401.7	$0.98\|^6H_{11/2}>$
26	3377.2	$0.99\|^6H_{11/2}>$	26	3401.7	$0.98\|^6H_{11/2}>$
27	3475.1	$0.98\|^6H_{11/2}>$	27	3536.5	$0.97\|^6H_{11/2}>$
28	3475.1	$0.98\|^6H_{11/2}>$	28	3536.5	$0.97\|^6H_{11/2}>$
29	3568.5	$0.97\|^6H_{11/2}>$	29	3664.0	$0.96\|^6H_{11/2}>$
30	3568.5	$0.97\|^6H_{11/2}>$	30	3664.0	$0.96\|^6H_{11/2}>$
31	3703.7	$0.98\|^6H_{11/2}>$	31	3765.4	$0.97\|^6H_{11/2}>$
32	3703.7	$0.98\|^6H_{11/2}>$	32	3765.4	$0.97\|^6H_{11/2}>$
33	3744.9	$0.98\|^6H_{11/2}>$	33	3808.9	$0.96\|^6H_{11/2}>$
34	3744.9	$0.98\|^6H_{11/2}>$	34	3808.9	$0.96\|^6H_{11/2}>$
35	3787.2	$0.98\|^6H_{11/2}>$	35	3979.4	$0.99\|^6H_{11/2}>$
36	3787.2	$0.98\|^6H_{11/2}>$	36	3979.4	$0.99\|^6H_{11/2}>$
37	4971.1	$0.99\|^6H_{13/2}>$	37	4966.5	$0.99\|^6H_{13/2}>$
38	4971.1	$0.99\|^6H_{13/2}>$	38	4966.5	$0.99\|^6H_{13/2}>$
39	5055.4	$0.99\|^6H_{13/2}>$	39	5112.4	$0.98\|^6H_{13/2}>$
40	5055.4	$0.99\|^6H_{13/2}>$	40	5112.4	$0.98\|^6H_{13/2}>$
41	5153.3	$0.98\|^6H_{13/2}>$	41	5226.1	$0.98\|^6H_{13/2}>$
42	5153.3	$0.98\|^6H_{13/2}>$	42	5226.1	$0.98\|^6H_{13/2}>$
43	5229.8	$0.97\|^6H_{13/2}>$	43	5330.8	$0.98\|^6H_{13/2}>$

44	5229.8	$0.97\|^6H_{13/2}>$	44	5330.8	$0.98\|^6H_{13/2}>$
45	5363.1	$0.98\|^6H_{13/2}>$	45	5410.9	$0.97\|^6H_{13/2}>$
46	5363.1	$0.98\|^6H_{13/2}>$	46	5410.9	$0.97\|^6H_{13/2}>$
47	5395.1	$0.99\|^6H_{13/2}>$	47	5492.1	$0.97\|^6H_{13/2}>$
48	5395.1	$0.99\|^6H_{13/2}>$	48	5492.1	$0.97\|^6H_{13/2}>$
49	5441.5	$0.98\|^6H_{13/2}>$	49	5667.0	$0.99\|^6H_{13/2}>$
50	5441.5	$0.98\|^6H_{13/2}>$	50	5667.0	$0.99\|^6H_{13/2}>$
51	6789.2	$0.99\|^6H_{15/2}>$	51	6736.9	$0.99\|^6H_{15/2}>$
52	6789.2	$0.99\|^6H_{15/2}>$	52	6736.9	$0.99\|^6H_{15/2}>$
53	6873.1	$0.99\|^6H_{15/2}>$	53	6945.4	$0.99\|^6H_{15/2}>$
54	6873.1	$0.99\|^6H_{15/2}>$	54	6945.4	$0.99\|^6H_{15/2}>$
55	6939.0	$0.98\|^6H_{15/2}>$	55	7052.2	$0.99\|^6H_{15/2}>$
56	6939.0	$0.98\|^6H_{15/2}>$	56	7052.2	$0.99\|^6H_{15/2}>$
57	7041.5	$0.98\|^6H_{15/2}>$	57	7122.5	$0.99\|^6H_{15/2}>$
58	7041.5	$0.98\|^6H_{15/2}>$	58	7122.5	$0.99\|^6H_{15/2}>$
59	7150.7	$0.98\|^6H_{15/2}>$	59	7195.3	$0.98\|^6H_{15/2}>$
60	7150.7	$0.98\|^6H_{15/2}>$	60	7195.3	$0.98\|^6H_{15/2}>$
61	7249.0	$0.99\|^6H_{15/2}>$	61	7338.1	$0.98\|^6H_{15/2}>$
62	7249.0	$0.99\|^6H_{15/2}>$	62	7338.1	$0.98\|^6H_{15/2}>$
63	7362.1	$1.00\|^6H_{15/2}>$	63	7517.1	$0.99\|^6H_{15/2}>$
64	7362.1	$1.00\|^6H_{15/2}>$	64	7517.1	$0.99\|^6H_{15/2}>$
65	7482.2	$0.99\|^6H_{15/2}>$	65	7643.6	$1.00\|^6H_{15/2}>$
66	7482.2	$0.99\|^6H_{15/2}>$	66	7643.6	$1.00\|^6H_{15/2}>$
67	8144.8	$0.97\|^6F_{1/2}>$	67	8210.5	$0.94\|^6F_{1/2}>$
68	8144.8	$0.97\|^6F_{1/2}>$	68	8210.5	$0.94\|^6F_{1/2}>$
69	8508.5	$0.98\|^6F_{3/2}>$	69	8588.6	$0.96\|^6F_{3/2}>$
70	8508.5	$0.98\|^6F_{3/2}>$	70	8588.6	$0.96\|^6F_{3/2}>$
71	8567.4	$0.96\|^6F_{3/2}>$	71	8649.6	$0.93\|^6F_{3/2}>$
72	8567.4	$0.96\|^6F_{3/2}>$	72	8649.6	$0.93\|^6F_{3/2}>$

73	9136.4	0.99|$^6F_{5/2}$>	73	9225.5	0.98|$^6F_{5/2}$>
74	9136.4	0.99|$^6F_{5/2}$>	74	9225.5	0.98|$^6F_{5/2}$>
75	9171.8	0.99|$^6F_{5/2}$>	75	9243.9	0.97|$^6F_{5/2}$>
76	9171.8	0.99|$^6F_{5/2}$>	76	9243.9	0.97|$^6F_{5/2}$>
77	9183.0	0.97|$^6F_{5/2}$>	77	9265.6	0.97|$^6F_{5/2}$>
78	9183.0	0.97|$^6F_{5/2}$>	78	9265.6	0.97|$^6F_{5/2}$>
79	10003.5	0.99|$^6F_{7/2}$>	79	10076.3	0.99|$^6F_{7/2}$>
80	10003.5	0.99|$^6F_{7/2}$>	80	10076.3	0.99|$^6F_{7/2}$>
81	10018.1	0.99|$^6F_{7/2}$>	81	10103.2	0.98|$^6F_{7/2}$>
82	10018.1	0.99|$^6F_{7/2}$>	82	10103.2	0.98|$^6F_{7/2}$>
83	10039.2	0.99|$^6F_{7/2}$>	83	10127.6	0.98|$^6F_{7/2}$>
84	10039.2	0.99|$^6F_{7/2}$>	84	10127.6	0.98|$^6F_{7/2}$>
85	10088.1	0.98|$^6F_{7/2}$>	85	10172.2	0.98|$^6F_{7/2}$>
86	10088.1	0.98|$^6F_{7/2}$>	86	10172.2	0.98|$^6F_{7/2}$>
87	11092.2	1.00|$^6F_{9/2}$>	87	11142.7	0.99|$^6F_{9/2}$>
88	11092.2	1.00|$^6F_{9/2}$>	88	11142.7	0.99|$^6F_{9/2}$>
89	11120.1	0.99|$^6F_{9/2}$>	89	11198.2	0.99|$^6F_{9/2}$>
90	11120.1	0.99|$^6F_{9/2}$>	90	11198.2	0.99|$^6F_{9/2}$>
91	11145.7	0.99|$^6F_{9/2}$>	91	11254.4	0.99|$^6F_{9/2}$>
92	11145.7	0.99|$^6F_{9/2}$>	92	11254.4	0.99|$^6F_{9/2}$>
93	11210.0	0.99|$^6F_{9/2}$>	93	11291.2	0.98|$^6F_{9/2}$>
94	11210.0	0.99|$^6F_{9/2}$>	94	11291.2	0.98|$^6F_{9/2}$>
95	11240.5	0.99|$^6F_{9/2}$>	95	11342.1	1.00|$^6F_{9/2}$>
96	11240.5	0.99|$^6F_{9/2}$>	96	11342.1	1.00|$^6F_{9/2}$>
97	12420.4	1.00|$^6F_{11/2}$>	97	12437.2	1.00|$^6F_{11/2}$>
98	12420.4	1.00|$^6F_{11/2}$>	98	12437.2	1.00|$^6F_{11/2}$>
99	12452.7	1.00|$^6F_{11/2}$>	99	12543.1	0.99|$^6F_{11/2}$>
100	12452.7	1.00|$^6F_{11/2}$>	100	12543.1	0.99|$^6F_{11/2}$>
101	12499.6	0.99|$^6F_{11/2}$>	101	12595.4	0.99|$^6F_{11/2}$>

102	12499.6	0.99|$^6F_{11/2}$>	102	12595.4	0.99|$^6F_{11/2}$>
103	12540.0	0.99|$^6F_{11/2}$>	103	12645.8	0.99|$^6F_{11/2}$>
104	12540.0	0.99|$^6F_{11/2}$>	104	12645.8	0.99|$^6F_{11/2}$>
105	12605.3	0.99|$^6F_{11/2}$>	105	12712.1	1.00|$^6F_{11/2}$>
106	12605.3	0.99|$^6F_{11/2}$>	106	12712.1	1.00|$^6F_{11/2}$>
107	12693.0	1.00|$^6F_{11/2}$>	107	12792.1	1.00|$^6F_{11/2}$>
108	12693.0	1.00|$^6F_{11/2}$>	108	12792.1	1.00|$^6F_{11/2}$>

Table S19. State energies and composition of the Sm complexes computed with MS-CASPT2-SO.

**2Sm**			**3Sm**		
**State**	**Energy (cm^{-1})**	**Composition**	**State**	**Energy (cm^{-1})**	**Composition**
1	0.0	0.98|$^6H_{5/2}$>	1	0.0	0.96|$^6H_{5/2}$>
2	0.0	0.98|$^6H_{5/2}$>	2	0.0	0.96|$^6H_{5/2}$>
3	139.9	0.95|$^6H_{5/2}$>	3	318.5	0.97|$^6H_{5/2}$>
4	139.9	0.95|$^6H_{5/2}$>	4	318.5	0.97|$^6H_{5/2}$>
5	305.8	0.95|$^6H_{5/2}$>	5	563.3	0.93|$^6H_{5/2}$>
6	305.8	0.95|$^6H_{5/2}$>	6	563.3	0.93|$^6H_{5/2}$>
7	842.1	0.96|$^6H_{7/2}$>	7	925.0	0.96|$^6H_{7/2}$>
8	842.1	0.96|$^6H_{7/2}$>	8	925.0	0.96|$^6H_{7/2}$>
9	1064.6	0.96|$^6H_{7/2}$>	9	1179.7	0.91|$^6H_{7/2}$>
10	1064.6	0.96|$^6H_{7/2}$>	10	1179.7	0.91|$^6H_{7/2}$>
11	1104.2	0.94|$^6H_{7/2}$>	11	1297.2	0.91|$^6H_{7/2}$>
12	1104.2	0.94|$^6H_{7/2}$>	12	1297.2	0.91|$^6H_{7/2}$>
13	1235.6	0.96|$^6H_{7/2}$>	13	1431.1	0.96|$^6H_{7/2}$>
14	1235.6	0.96|$^6H_{7/2}$>	14	1431.1	0.96|$^6H_{7/2}$>
15	2013.4	0.96|$^6H_{7/2}$>	15	2080.3	0.96|$^6H_{7/2}$>
16	2013.4	0.96|$^6H_{7/2}$>	16	2080.3	0.96|$^6H_{7/2}$>
17	2060.0	0.96|$^6H_{7/2}$>	17	2223.8	0.93|$^6H_{7/2}$>
18	2060.0	0.96|$^6H_{7/2}$>	18	2223.8	0.93|$^6H_{7/2}$>

19	2218.7	0.97|$^6H_{7/2}$>	19	2384.5	0.95|$^6H_{7/2}$>
20	2218.7	0.97|$^6H_{7/2}$>	20	2384.5	0.95|$^6H_{7/2}$>
21	2281.8	0.97|$^6H_{7/2}$>	21	2430.3	0.95|$^6H_{7/2}$>
22	2281.8	0.97|$^6H_{7/2}$>	22	2430.3	0.95|$^6H_{7/2}$>
23	2354.3	0.97|$^6H_{7/2}$>	23	2585.8	0.97|$^6H_{7/2}$>
24	2354.3	0.97|$^6H_{7/2}$>	24	2585.8	0.97|$^6H_{7/2}$>
25	3347.1	0.96|$^6H_{11/2}$>	25	3407.3	0.97|$^6H_{11/2}$>
26	3347.1	0.96|$^6H_{11/2}$>	26	3407.3	0.97|$^6H_{11/2}$>
27	3404.3	0.97|$^6H_{11/2}$>	27	3547.5	0.95|$^6H_{11/2}$>
28	3404.3	0.97|$^6H_{11/2}$>	28	3547.5	0.95|$^6H_{11/2}$>
29	3509.4	0.96|$^6H_{11/2}$>	29	3675.3	0.96|$^6H_{11/2}$>
30	3509.4	0.96|$^6H_{11/2}$>	30	3675.3	0.96|$^6H_{11/2}$>
31	3632.8	0.98|$^6H_{11/2}$>	31	3790.2	0.97|$^6H_{11/2}$>
32	3632.8	0.98|$^6H_{11/2}$>	32	3790.2	0.97|$^6H_{11/2}$>
33	3663.4	0.97|$^6H_{11/2}$>	33	3839.7	0.96|$^6H_{11/2}$>
34	3663.4	0.97|$^6H_{11/2}$>	34	3839.7	0.96|$^6H_{11/2}$>
35	3744.2	0.97|$^6H_{11/2}$>	35	4014.3	0.99|$^6H_{11/2}$>
36	3744.2	0.97|$^6H_{11/2}$>	36	4014.3	0.99|$^6H_{11/2}$>
37	4935.7	0.94|$^6H_{13/2}$>	37	4942.6	0.96|$^6H_{13/2}$>
38	4935.7	0.94|$^6H_{13/2}$>	38	4942.6	0.96|$^6H_{13/2}$>
39	4982.2	0.95|$^6H_{13/2}$>	39	5120.9	0.96|$^6H_{13/2}$>
40	4982.2	0.95|$^6H_{13/2}$>	40	5120.9	0.96|$^6H_{13/2}$>
41	5084.4	0.96|$^6H_{13/2}$>	41	5246.9	0.97|$^6H_{13/2}$>
42	5084.4	0.96|$^6H_{13/2}$>	42	5246.9	0.97|$^6H_{13/2}$>
43	5159.8	0.96|$^6H_{13/2}$>	43	5346.1	0.97|$^6H_{13/2}$>
44	5159.8	0.96|$^6H_{13/2}$>	44	5346.1	0.97|$^6H_{13/2}$>
45	5254.1	0.95|$^6H_{13/2}$>	45	5434.2	0.96|$^6H_{13/2}$>
46	5254.1	0.95|$^6H_{13/2}$>	46	5434.2	0.96|$^6H_{13/2}$>
47	5319.6	0.97|$^6H_{13/2}$>	47	5519.4	0.97|$^6H_{13/2}$>

48	5319.6	$0.97\|^6H_{13/2}>$	48	5519.4	$0.97\|^6H_{13/2}>$
49	5341.4	$0.97\|^6H_{13/2}>$	49	5687.5	$0.99\|^6H_{13/2}>$
50	5341.4	$0.97\|^6H_{13/2}>$	50	5687.5	$0.99\|^6H_{13/2}>$
51	6635.2	$0.61\|^6H_{15/2}> + 0.24\|^6F_{1/2}>$	51	6663.2	$0.92\|^6H_{15/2}>$
52	6635.2	$0.61\|^6H_{15/2}> + 0.24\|^6F_{1/2}>$	52	6663.2	$0.92\|^6H_{15/2}>$
53	6678.7	$0.52\|^6H_{15/2}> + 0.34\|^6F_{1/2}>$	53	6827.0	$0.74\|^6F_{1/2}> + 0.19\|^6H_{15/2}>$
54	6678.7	$0.52\|^6H_{15/2}> + 0.34\|^6F_{1/2}>$	54	6827.0	$0.74\|^6F_{1/2}> + 0.19\|^6H_{15/2}>$
55	6745.3	$0.65\|^6H_{15/2}> + 0.18\|^6F_{1/2}>$	55	6913.4	$0.76\|^6H_{15/2}> + 0.12\|^6F_{1/2}>$
56	6745.3	$0.65\|^6H_{15/2}> + 0.18\|^6F_{1/2}>$	56	6913.4	$0.76\|^6H_{15/2}> + 0.12\|^6F_{1/2}>$
57	6825.4	$0.76\|^6H_{15/2}>$	57	7065.1	$0.91\|^6H_{15/2}>$
58	6825.4	$0.76\|^6H_{15/2}>$	58	7065.1	$0.91\|^6H_{15/2}>$
59	6925.6	$0.78\|^6H_{15/2}>$	59	7093.1	$0.72\|^6H_{15/2}> + 0.21\|^6F_{3/2}>$
60	6925.6	$0.78\|^6H_{15/2}>$	60	7093.1	$0.72\|^6H_{15/2}> + 0.21\|^6F_{3/2}>$
61	7021.2	$0.86\|^6H_{15/2}>$	61	7192.4	$0.81\|^6H_{15/2}> + 0.15\|^6F_{3/2}>$
62	7021.2	$0.86\|^6H_{15/2}>$	62	7192.4	$0.81\|^6H_{15/2}> + 0.15\|^6F_{3/2}>$
63	7090.7	$0.52\|^6H_{15/2}> + 0.42\|^6F_{3/2}>$	63	7282.9	$0.67\|^6F_{3/2}> + 0.28\|^6H_{15/2}>$
64	7090.7	$0.52\|^6H_{15/2}> + 0.42\|^6F_{3/2}>$	64	7282.9	$0.67\|^6F_{3/2}> + 0.28\|^6H_{15/2}>$
65	7166.2	$0.55\|^6F_{3/2}> + 0.39\|^6H_{15/2}>$	65	7330.7	$0.51\|^6F_{3/2}> + 0.43\|^6H_{15/2}>$
66	7166.2	$0.55\|^6F_{3/2}> + 0.39\|^6H_{15/2}>$	66	7330.7	$0.51\|^6F_{3/2}> + 0.43\|^6H_{15/2}>$
67	7208.1	$0.49\|^6F_{3/2}> + 0.45\|^6H_{15/2}>$	67	7379.8	$0.71\|^6H_{15/2}> + 0.23\|^6F_{3/2}>$
68	7208.1	$0.49\|^6F_{3/2}> + 0.45\|^6H_{15/2}>$	68	7379.8	$0.71\|^6H_{15/2}> + 0.23\|^6F_{3/2}>$
69	7316.0	$0.94\|^6H_{15/2}>$	69	7548.0	$0.97\|^6H_{15/2}>$
70	7316.0	$0.94\|^6H_{15/2}>$	70	7548.0	$0.97\|^6H_{15/2}>$
71	7417.7	$0.95\|^6H_{15/2}>$	71	7659.4	$0.97\|^6H_{15/2}>$
72	7417.7	$0.95\|^6H_{15/2}>$	72	7659.4	$0.97\|^6H_{15/2}>$
73	7689.1	$0.93\|^6F_{5/2}>$	73	7873.4	$0.94\|^6F_{5/2}>$
74	7689.1	$0.93\|^6F_{5/2}>$	74	7873.4	$0.94\|^6F_{5/2}>$
75	7765.4	$0.91\|^6F_{5/2}>$	75	7907.3	$0.92\|^6F_{5/2}>$
76	7765.4	$0.91\|^6F_{5/2}>$	76	7907.3	$0.92\|^6F_{5/2}>$

77	7832.1	0.85|$^6F_{5/2}$> + 0.12|$^6H_{15/2}$>	77	7944.6	0.92|$^6F_{5/2}$>
78	7832.1	0.85|$^6F_{5/2}$> + 0.12|$^6H_{15/2}$>	78	7944.6	0.92|$^6F_{5/2}$>
79	8545.0	0.96|$^6F_{7/2}$>	79	8716.0	0.97|$^6F_{7/2}$>
80	8545.0	0.96|$^6F_{7/2}$>	80	8716.0	0.97|$^6F_{7/2}$>
81	8573.7	0.97|$^6F_{7/2}$>	81	8743.9	0.96|$^6F_{7/2}$>
82	8573.7	0.97|$^6F_{7/2}$>	82	8743.9	0.96|$^6F_{7/2}$>
83	8622.7	0.93|$^6F_{7/2}$>	83	8773.5	0.97|$^6F_{7/2}$>
84	8622.7	0.93|$^6F_{7/2}$>	84	8773.5	0.97|$^6F_{7/2}$>
85	8686.4	0.93|$^6F_{7/2}$>	85	8840.6	0.96|$^6F_{7/2}$>
86	8686.4	0.93|$^6F_{7/2}$>	86	8840.6	0.96|$^6F_{7/2}$>
87	9626.5	0.98|$^6F_{9/2}$>	87	9777.4	0.98|$^6F_{9/2}$>
88	9626.5	0.98|$^6F_{9/2}$>	88	9777.4	0.98|$^6F_{9/2}$>
89	9678.3	0.96|$^6F_{9/2}$>	89	9836.1	0.98|$^6F_{9/2}$>
90	9678.3	0.96|$^6F_{9/2}$>	90	9836.1	0.98|$^6F_{9/2}$>
91	9719.0	0.97|$^6F_{9/2}$>	91	9905.7	0.98|$^6F_{9/2}$>
92	9719.0	0.97|$^6F_{9/2}$>	92	9905.7	0.98|$^6F_{9/2}$>
93	9745.1	0.96|$^6F_{9/2}$>	93	9926.1	0.98|$^6F_{9/2}$>
94	9745.1	0.96|$^6F_{9/2}$>	94	9926.1	0.98|$^6F_{9/2}$>
95	9779.9	0.98|$^6F_{9/2}$>	95	9984.6	0.99|$^6F_{9/2}$>
96	9779.9	0.98|$^6F_{9/2}$>	96	9984.6	0.99|$^6F_{9/2}$>
97	10935.7	0.99|$^6F_{11/2}$>	97	11059.3	1.00|$^6F_{11/2}$>
98	10935.7	0.99|$^6F_{11/2}$>	98	11059.3	1.00|$^6F_{11/2}$>
99	10966.7	0.99|$^6F_{11/2}$>	99	11185.3	0.99|$^6F_{11/2}$>
100	10966.7	0.99|$^6F_{11/2}$>	100	11185.3	0.99|$^6F_{11/2}$>
101	11043.1	0.98|$^6F_{11/2}$>	101	11236.9	0.99|$^6F_{11/2}$>
102	11043.1	0.98|$^6F_{11/2}$>	102	11236.9	0.99|$^6F_{11/2}$>
103	11082.8	0.98|$^6F_{11/2}$>	103	11287.3	0.99|$^6F_{11/2}$>
104	11082.8	0.98|$^6F_{11/2}$>	104	11287.3	0.99|$^6F_{11/2}$>
105	11145.1	0.98|$^6F_{11/2}$>	105	11350.9	0.99|$^6F_{11/2}$>

106	11145.1	0.98|$^6F_{11/2}$>	106	11350.9	0.99|$^6F_{11/2}$>
107	11238.4	0.99|$^6F_{11/2}$>	107	11416.6	0.99|$^6F_{11/2}$>
108	11238.4	0.99|$^6F_{11/2}$>	108	11416.6	0.99|$^6F_{11/2}$>

Table S20. State energies and composition of the Sm complexes computed with XMS-CASPT2-SO.

2Sm			3Sm		
**State**	**Energy (cm^{-1})**	**Composition**	**State**	**Energy (cm^{-1})**	**Composition**
1	0.0	0.94|$^6H_{5/2}$>	1	0.0	0.98|$^6H_{5/2}$>
2	0.0	0.94|$^6H_{5/2}$>	2	0.0	0.98|$^6H_{5/2}$>
3	242.3	0.90|$^6H_{5/2}$>	3	230.9	0.94|$^6H_{5/2}$>
4	242.3	0.90|$^6H_{5/2}$>	4	230.9	0.94|$^6H_{5/2}$>
5	433.7	0.90|$^6H_{5/2}$>	5	560.3	0.91|$^6H_{5/2}$>
6	433.7	0.90|$^6H_{5/2}$>	6	560.3	0.91|$^6H_{5/2}$>
7	887.3	0.86|$^6H_{7/2}$>	7	886.7	0.97|$^6H_{7/2}$>
8	887.3	0.86|$^6H_{7/2}$>	8	886.7	0.97|$^6H_{7/2}$>
9	1105.3	0.90|$^6H_{7/2}$>	9	1112.8	0.92|$^6H_{7/2}$>
10	1105.3	0.90|$^6H_{7/2}$>	10	1112.8	0.92|$^6H_{7/2}$>
11	1262.9	0.89|$^6H_{7/2}$>	11	1291.2	0.87|$^6H_{7/2}$>
12	1262.9	0.89|$^6H_{7/2}$>	12	1291.2	0.87|$^6H_{7/2}$>
13	1405.0	0.87|$^6H_{7/2}$>	13	1426.6	0.89|$^6H_{7/2}$>
14	1405.0	0.87|$^6H_{7/2}$>	14	1426.6	0.89|$^6H_{7/2}$>
15	2088.3	0.88|$^6H_{7/2}$>	15	2033.5	0.96|$^6H_{7/2}$>
16	2088.3	0.88|$^6H_{7/2}$>	16	2033.5	0.96|$^6H_{7/2}$>
17	2207.4	0.88|$^6H_{7/2}$>	17	2173.0	0.94|$^6H_{7/2}$>
18	2207.4	0.88|$^6H_{7/2}$>	18	2173.0	0.94|$^6H_{7/2}$>
19	2351.4	0.89|$^6H_{7/2}$>	19	2383.0	0.90|$^6H_{7/2}$>
20	2351.4	0.89|$^6H_{7/2}$>	20	2383.0	0.90|$^6H_{7/2}$>
21	2452.6	0.91|$^6H_{7/2}$>	21	2476.9	0.90|$^6H_{7/2}$>
22	2452.6	0.91|$^6H_{7/2}$>	22	2476.9	0.90|$^6H_{7/2}$>

23	2539.5	$0.91\vert{}^{6}H_{7/2}>$	23	2572.0	$0.93\vert{}^{6}H_{7/2}>$
24	2539.5	$0.91\vert{}^{6}H_{7/2}>$	24	2572.0	$0.93\vert{}^{6}H_{7/2}>$
25	3391.0	$0.90\vert{}^{6}H_{11/2}>$	25	3349.0	$0.96\vert{}^{6}H_{11/2}>$
26	3391.0	$0.90\vert{}^{6}H_{11/2}>$	26	3349.0	$0.96\vert{}^{6}H_{11/2}>$
27	3526.8	$0.92\vert{}^{6}H_{11/2}>$	27	3484.8	$0.96\vert{}^{6}H_{11/2}>$
28	3526.8	$0.92\vert{}^{6}H_{11/2}>$	28	3484.8	$0.96\vert{}^{6}H_{11/2}>$
29	3643.6	$0.94\vert{}^{6}H_{11/2}>$	29	3681.8	$0.95\vert{}^{6}H_{11/2}>$
30	3643.6	$0.94\vert{}^{6}H_{11/2}>$	30	3681.8	$0.95\vert{}^{6}H_{11/2}>$
31	3793.1	$0.92\vert{}^{6}H_{11/2}>$	31	3805.7	$0.93\vert{}^{6}H_{11/2}>$
32	3793.1	$0.92\vert{}^{6}H_{11/2}>$	32	3805.7	$0.93\vert{}^{6}H_{11/2}>$
33	3904.8	$0.95\vert{}^{6}H_{11/2}>$	33	3867.3	$0.94\vert{}^{6}H_{11/2}>$
34	3904.8	$0.95\vert{}^{6}H_{11/2}>$	34	3867.3	$0.94\vert{}^{6}H_{11/2}>$
35	4026.5	$0.90\vert{}^{6}H_{11/2}>$	35	4066.9	$0.94\vert{}^{6}H_{11/2}>$
36	4026.5	$0.90\vert{}^{6}H_{11/2}>$	36	4066.9	$0.94\vert{}^{6}H_{11/2}>$
37	4997.3	$0.92\vert{}^{6}H_{13/2}>$	37	4940.2	$0.98\vert{}^{6}H_{13/2}>$
38	4997.3	$0.92\vert{}^{6}H_{13/2}>$	38	4940.2	$0.98\vert{}^{6}H_{13/2}>$
39	5085.3	$0.93\vert{}^{6}H_{13/2}>$	39	5041.3	$0.97\vert{}^{6}H_{13/2}>$
40	5085.3	$0.93\vert{}^{6}H_{13/2}>$	40	5041.3	$0.97\vert{}^{6}H_{13/2}>$
41	5224.4	$0.93\vert{}^{6}H_{13/2}>$	41	5229.6	$0.94\vert{}^{6}H_{13/2}>$
42	5224.4	$0.93\vert{}^{6}H_{13/2}>$	42	5229.6	$0.94\vert{}^{6}H_{13/2}>$
43	5346.4	$0.92\vert{}^{6}H_{13/2}>$	43	5314.1	$0.95\vert{}^{6}H_{13/2}>$
44	5346.4	$0.92\vert{}^{6}H_{13/2}>$	44	5314.1	$0.95\vert{}^{6}H_{13/2}>$
45	5435.2	$0.93\vert{}^{6}H_{13/2}>$	45	5430.6	$0.93\vert{}^{6}H_{13/2}>$
46	5435.2	$0.93\vert{}^{6}H_{13/2}>$	46	5430.6	$0.93\vert{}^{6}H_{13/2}>$
47	5578.5	$0.94\vert{}^{6}H_{13/2}>$	47	5640.3	$0.96\vert{}^{6}H_{13/2}>$
48	5578.5	$0.94\vert{}^{6}H_{13/2}>$	48	5640.3	$0.96\vert{}^{6}H_{13/2}>$
49	5736.4	$0.92\vert{}^{6}H_{13/2}>$	49	5732.8	$0.96\vert{}^{6}H_{13/2}>$
50	5736.4	$0.92\vert{}^{6}H_{13/2}>$	50	5732.8	$0.96\vert{}^{6}H_{13/2}>$
51	6797.8	$0.91\vert{}^{6}H_{15/2}>$	51	6795.8	$0.94\vert{}^{6}H_{15/2}>$

52	6797.8	0.91|$^6H_{15/2}$>	52	6795.8	0.94|$^6H_{15/2}$>
53	6957.6	0.89|$^6H_{15/2}$>	53	6837.8	0.96|$^6H_{15/2}$>
54	6957.6	0.89|$^6H_{15/2}$>	54	6837.8	0.96|$^6H_{15/2}$>
55	7033.3	0.88|$^6H_{15/2}$>	55	6911.7	0.94|$^6H_{15/2}$>
56	7033.3	0.88|$^6H_{15/2}$>	56	6911.7	0.94|$^6H_{15/2}$>
57	7148.7	0.83|$^6H_{15/2}$>	57	7127.2	0.85|$^6H_{15/2}$>
58	7148.7	0.83|$^6H_{15/2}$>	58	7127.2	0.85|$^6H_{15/2}$>
59	7185.9	0.74|$^6H_{15/2}$> + 0.15|$^6F_{1/2}$>	59	7252.6	0.77|$^6H_{15/2}$> + 0.16|$^6F_{1/2}$>
60	7185.9	0.74|$^6H_{15/2}$> + 0.15|$^6F_{1/2}$>	60	7252.6	0.77|$^6H_{15/2}$> + 0.16|$^6F_{1/2}$>
61	7318.8	0.83|$^6H_{15/2}$>	61	7361.8	0.48|$^6H_{15/2}$> + 0.39|$^6F_{1/2}$>
62	7318.8	0.83|$^6H_{15/2}$>	62	7361.8	0.48|$^6H_{15/2}$> + 0.39|$^6F_{1/2}$>
63	7420.0	0.60|$^6H_{15/2}$> + 0.29|$^6F_{1/2}$>	63	7419.3	0.80|$^6H_{15/2}$>
64	7420.0	0.60|$^6H_{15/2}$> + 0.29|$^6F_{1/2}$>	64	7419.3	0.80|$^6H_{15/2}$>
65	7582.3	0.61|$^6H_{15/2}$> + 0.25|$^6F_{1/2}$>	65	7547.2	0.84|$^6H_{15/2}$>
66	7582.3	0.61|$^6H_{15/2}$> + 0.25|$^6F_{1/2}$>	66	7547.2	0.84|$^6H_{15/2}$>
67	7746.5	0.67|$^6H_{15/2}$> + 0.22|$^6F_{3/2}$>	67	7719.1	0.56|$^6H_{15/2}$> + 0.34|$^6F_{3/2}$>
68	7746.5	0.67|$^6H_{15/2}$> + 0.22|$^6F_{3/2}$>	68	7719.1	0.56|$^6H_{15/2}$> + 0.34|$^6F_{3/2}$>
69	7821.2	0.70|$^6F_{3/2}$> + 0.17|$^6H_{15/2}$>	69	7768.8	0.49|$^6F_{3/2}$> + 0.38|$^6H_{15/2}$>
70	7821.2	0.70|$^6F_{3/2}$> + 0.17|$^6H_{15/2}$>	70	7768.8	0.49|$^6F_{3/2}$> + 0.38|$^6H_{15/2}$>
71	7875.7	0.55|$^6F_{3/2}$> + 0.31|$^6H_{15/2}$>	71	7915.8	0.62|$^6F_{3/2}$> + 0.16|$^6H_{15/2}$>
72	7875.7	0.55|$^6F_{3/2}$> + 0.31|$^6H_{15/2}$>	72	7915.8	0.62|$^6F_{3/2}$> + 0.16|$^6H_{15/2}$>
73	8393.9	0.89|$^6F_{5/2}$>	73	8329.2	0.88|$^6F_{5/2}$>
74	8393.9	0.89|$^6F_{5/2}$>	74	8329.2	0.88|$^6F_{5/2}$>
75	8458.3	0.84|$^6F_{5/2}$> + 0.10|$^6F_{3/2}$>	75	8491.5	0.81|$^6F_{5/2}$>
76	8458.3	0.84|$^6F_{5/2}$> + 0.10|$^6F_{3/2}$>	76	8491.5	0.81|$^6F_{5/2}$>
77	8552.4	0.85|$^6F_{5/2}$>	77	8654.5	0.76|$^6F_{5/2}$> + 0.11|$^6F_{3/2}$>
78	8552.4	0.85|$^6F_{5/2}$>	78	8654.5	0.76|$^6F_{5/2}$> + 0.11|$^6F_{3/2}$>
79	9162.0	0.92|$^6F_{7/2}$>	79	9142.8	0.88|$^6F_{7/2}$>
80	9162.0	0.92|$^6F_{7/2}$>	80	9142.8	0.88|$^6F_{7/2}$>

81	9236.9	0.92|$^6F_{7/2}$>	81	9278.5	0.88|$^6F_{7/2}$>
82	9236.9	0.92|$^6F_{7/2}$>	82	9278.5	0.88|$^6F_{7/2}$>
83	9353.2	0.93|$^6F_{7/2}$>	83	9460.3	0.88|$^6F_{7/2}$>
84	9353.2	0.93|$^6F_{7/2}$>	84	9460.3	0.88|$^6F_{7/2}$>
85	9511.3	0.94|$^6F_{7/2}$>	85	9553.2	0.83|$^6F_{7/2}$>
86	9511.3	0.94|$^6F_{7/2}$>	86	9553.2	0.83|$^6F_{7/2}$>
87	10263.0	0.94|$^6F_{9/2}$>	87	10200.9	0.95|$^6F_{9/2}$>
88	10263.0	0.94|$^6F_{9/2}$>	88	10200.9	0.95|$^6F_{9/2}$>
89	10326.6	0.94|$^6F_{9/2}$>	89	10371.9	0.92|$^6F_{9/2}$>
90	10326.6	0.94|$^6F_{9/2}$>	90	10371.9	0.92|$^6F_{9/2}$>
91	10412.2	0.94|$^6F_{9/2}$>	91	10507.9	0.92|$^6F_{9/2}$>
92	10412.2	0.94|$^6F_{9/2}$>	92	10507.9	0.92|$^6F_{9/2}$>
93	10496.5	0.95|$^6F_{9/2}$>	93	10707.5	0.85|$^6F_{9/2}$>
94	10496.5	0.95|$^6F_{9/2}$>	94	10707.5	0.85|$^6F_{9/2}$>
95	10708.3	0.95|$^6F_{9/2}$>	95	10795.9	0.88|$^6F_{9/2}$>
96	10708.3	0.95|$^6F_{9/2}$>	96	10795.9	0.88|$^6F_{9/2}$>
97	11517.9	0.96|$^6F_{11/2}$>	97	11479.7	0.99|$^6F_{11/2}$>
98	11517.9	0.96|$^6F_{11/2}$>	98	11479.7	0.99|$^6F_{11/2}$>
99	11742.9	0.96|$^6F_{11/2}$>	99	11603.0	0.99|$^6F_{11/2}$>
100	11742.9	0.96|$^6F_{11/2}$>	100	11603.0	0.99|$^6F_{11/2}$>
101	11798.2	0.96|$^6F_{11/2}$>	101	11847.7	0.93|$^6F_{11/2}$>
102	11798.2	0.96|$^6F_{11/2}$>	102	11847.7	0.93|$^6F_{11/2}$>
103	11894.5	0.96|$^6F_{11/2}$>	103	12047.2	0.92|$^6F_{11/2}$>
104	11894.5	0.96|$^6F_{11/2}$>	104	12047.2	0.92|$^6F_{11/2}$>
105	11960.3	0.99|$^6F_{11/2}$>	105	12201.5	0.94|$^6F_{11/2}$>
106	11960.3	0.99|$^6F_{11/2}$>	106	12201.5	0.94|$^6F_{11/2}$>
107	12108.2	0.99|$^6F_{11/2}$>	107	12382.7	0.97|$^6F_{11/2}$>
108	12108.2	0.99|$^6F_{11/2}$>	108	12382.7	0.97|$^6F_{11/2}$>

Table S21. State energies and composition of the Pu complexes computed with CASSCF-SO.

2Pu			3Pu		
**State**	**Energy (cm^{-1})**	**Composition**	**State**	**Energy (cm^{-1})**	**Composition**
1	0	$0.98\|^6H_{5/2}>$	1	0	$0.98\|^6H_{5/2}>$
2	0	$0.98\|^6H_{5/2}>$	2	0	$0.98\|^6H_{5/2}>$
3	379.8	$0.97\|^6H_{5/2}>$	3	426.4	$0.97\|^6H_{5/2}>$
4	379.8	$0.97\|^6H_{5/2}>$	4	426.4	$0.97\|^6H_{5/2}>$
5	763.0	$0.95\|^6H_{5/2}>$	5	968.4	$0.93\|^6H_{5/2}>$
6	763.0	$0.95\|^6H_{5/2}>$	6	968.4	$0.93\|^6H_{5/2}>$
7	1646.4	$0.95\|^6H_{7/2}>$	7	1737.3	$0.95\|^6H_{7/2}>$
8	1646.4	$0.95\|^6H_{7/2}>$	8	1737.3	$0.95\|^6H_{7/2}>$
9	2123.8	$0.95\|^6H_{7/2}>$	9	2160.0	$0.92\|^6H_{7/2}> + 0.10\|^6H_{5/2}>$
10	2123.8	$0.95\|^6H_{7/2}>$	10	2160.0	$0.92\|^6H_{7/2}> + 0.10\|^6H_{5/2}>$
11	2253.8	$0.91\|^6H_{7/2}> + 0.11\|^6H_{5/2}>$	11	2362.8	$0.89\|^6H_{7/2}> + 0.12\|^6H_{5/2}>$
12	2253.8	$0.91\|^6H_{7/2}> + 0.11\|^6H_{5/2}>$	12	2362.8	$0.89\|^6H_{7/2}> + 0.12\|^6H_{5/2}>$
13	2500.7	$0.96\|^6H_{7/2}>$	13	2636.3	$0.95\|^6H_{7/2}>$
14	2500.7	$0.96\|^6H_{7/2}>$	14	2636.3	$0.95\|^6H_{7/2}>$
15	3884.6	$0.94\|^6H_{7/2}>$	15	3959.2	$0.95\|^6H_{7/2}>$
16	3884.6	$0.94\|^6H_{7/2}>$	16	3959.2	$0.95\|^6H_{7/2}>$
17	4089.1	$0.92\|^6H_{7/2}> + 0.10\|^6H_{7/2}>$	17	4174.7	$0.92\|^6H_{7/2}> + 0.10\|^6H_{7/2}>$
18	4089.1	$0.92\|^6H_{7/2}> + 0.10\|^6H_{7/2}>$	18	4174.7	$0.92\|^6H_{7/2}> + 0.10\|^6H_{7/2}>$
19	4345.9	$0.96\|^6H_{7/2}>$	19	4444.8	$0.93\|^6H_{7/2}>$
20	4345.9	$0.96\|^6H_{7/2}>$	20	4444.8	$0.93\|^6H_{7/2}>$
21	4538.3	$0.95\|^6H_{7/2}>$	21	4543.9	$0.93\|^6H_{7/2}>$
22	4538.3	$0.95\|^6H_{7/2}>$	22	4543.9	$0.93\|^6H_{7/2}>$
23	4668.5	$0.96\|^6H_{7/2}>$	23	4873.2	$0.96\|^6H_{7/2}>$
24	4668.5	$0.96\|^6H_{7/2}>$	24	4873.2	$0.96\|^6H_{7/2}>$
25	6381.5	$0.85\|^6H_{11/2}>$	25	6498.8	$0.91\|^6H_{11/2}>$
26	6381.5	$0.85\|^6H_{11/2}>$	26	6498.8	$0.91\|^6H_{11/2}>$

27	6657.5	0.92|$^6H_{11/2}$>	27	6711.4	0.90|$^6H_{11/2}$>
28	6657.5	0.92|$^6H_{11/2}$>	28	6711.4	0.90|$^6H_{11/2}$>
29	6811.5	0.86|$^6H_{11/2}$>	29	6901.7	0.84|$^6H_{11/2}$>
30	6811.5	0.86|$^6H_{11/2}$>	30	6901.7	0.84|$^6H_{11/2}$>
31	7055.9	0.93|$^6H_{11/2}$>	31	7111.3	0.90|$^6H_{11/2}$>
32	7055.9	0.93|$^6H_{11/2}$>	32	7111.3	0.90|$^6H_{11/2}$>
33	7161.3	0.84|$^6H_{11/2}$> + 0.10|$^6F_{1/2}$>	33	7216.3	0.91|$^6H_{11/2}$>
34	7161.3	0.84|$^6H_{11/2}$> + 0.10|$^6F_{1/2}$>	34	7216.3	0.91|$^6H_{11/2}$>
35	7340.7	0.85|$^6H_{11/2}$>	35	7586.7	0.47|$^6H_{11/2}$> + 0.46|$^6F_{1/2}$>
36	7340.7	0.85|$^6H_{11/2}$>	36	7586.7	0.47|$^6H_{11/2}$> + 0.46|$^6F_{1/2}$>
37	7643.6	0.59|$^6F_{1/2}$> + 0.33|$^6H_{11/2}$>	37	7655.7	0.69|$^6H_{11/2}$> + 0.25|$^6F_{1/2}$>
38	7643.6	0.59|$^6F_{1/2}$> + 0.33|$^6H_{11/2}$>	38	7655.7	0.69|$^6H_{11/2}$> + 0.25|$^6F_{1/2}$>
39	8178.7	0.90|$^6F_{3/2}$>	39	8204.8	0.91|$^6F_{3/2}$>
40	8178.7	0.90|$^6F_{3/2}$>	40	8204.8	0.91|$^6F_{3/2}$>
41	8260.2	0.83|$^6F_{3/2}$>	41	8327.4	0.85|$^6F_{3/2}$>
42	8260.2	0.83|$^6F_{3/2}$>	42	8327.4	0.85|$^6F_{3/2}$>
43	9118.4	0.65|$^6F_{5/2}$> + 0.29|$^6H_{13/2}$>	43	9312.1	0.61|$^6F_{5/2}$> + 0.32|$^6H_{13/2}$>
44	9118.4	0.65|$^6F_{5/2}$> + 0.29|$^6H_{13/2}$>	44	9312.1	0.61|$^6F_{5/2}$> + 0.32|$^6H_{13/2}$>
45	9291.7	0.74|$^6F_{5/2}$> + 0.17|$^6H_{13/2}$>	45	9366.7	0.81|$^6F_{5/2}$> + 0.14|$^6H_{13/2}$>
46	9291.7	0.74|$^6F_{5/2}$> + 0.17|$^6H_{13/2}$>	46	9366.7	0.81|$^6F_{5/2}$> + 0.14|$^6H_{13/2}$>
47	9422.8	0.92|$^6F_{5/2}$>	47	9474.6	0.91|$^6F_{5/2}$>
48	9422.8	0.92|$^6F_{5/2}$>	48	9474.6	0.91|$^6F_{5/2}$>
49	9745.5	0.78|$^6H_{13/2}$> + 0.13|$^6F_{5/2}$>	49	9710.9	0.68|$^6H_{13/2}$> + 0.25|$^6F_{5/2}$>
50	9745.5	0.78|$^6H_{13/2}$> + 0.13|$^6F_{5/2}$>	50	9710.9	0.68|$^6H_{13/2}$> + 0.25|$^6F_{5/2}$>
51	9869.9	0.74|$^6H_{13/2}$> + 0.18|$^6F_{5/2}$>	51	9896.1	0.77|$^6H_{13/2}$> + 0.16|$^6F_{5/2}$>
52	9869.9	0.74|$^6H_{13/2}$> + 0.18|$^6F_{5/2}$>	52	9896.1	0.77|$^6H_{13/2}$> + 0.16|$^6F_{5/2}$>
53	10004.3	0.77|$^6H_{13/2}$> + 0.15|$^6F_{5/2}$>	53	10037.6	0.86|$^6H_{13/2}$>
54	10004.3	0.77|$^6H_{13/2}$> + 0.15|$^6F_{5/2}$>	54	10037.6	0.86|$^6H_{13/2}$>
55	10151.6	0.88|$^6H_{13/2}$>	55	10188.7	0.89|$^6H_{13/2}$>

56	10151.6	0.88|$^6H_{13/2}$>	56	10188.7	0.89|$^6H_{13/2}$>
57	10272.6	0.92|$^6H_{13/2}$>	57	10333.1	0.86|$^6H_{13/2}$>
58	10272.6	0.92|$^6H_{13/2}$>	58	10333.1	0.86|$^6H_{13/2}$>
59	10398.9	0.85|$^6H_{13/2}$>	59	10505.7	0.92|$^6H_{13/2}$>
60	10398.9	0.85|$^6H_{13/2}$>	60	10505.7	0.92|$^6H_{13/2}$>
61	10581.8	0.85|$^6H_{13/2}$>	61	10817.4	0.82|$^6H_{13/2}$> + 0.14|$^6F_{7/2}$>
62	10581.8	0.85|$^6H_{13/2}$>	62	10817.4	0.82|$^6H_{13/2}$> + 0.14|$^6F_{7/2}$>
63	11045.8	0.90|$^6F_{7/2}$>	63	11102.9	0.89|$^6F_{7/2}$>
64	11045.8	0.90|$^6F_{7/2}$>	64	11102.9	0.89|$^6F_{7/2}$>
65	11088.7	0.89|$^6F_{7/2}$>	65	11143.7	0.83|$^6F_{7/2}$> + 0.12|$^6H_{13/2}$>
66	11088.7	0.89|$^6F_{7/2}$>	66	11143.7	0.83|$^6F_{7/2}$> + 0.12|$^6H_{13/2}$>
67	11139.4	0.88|$^6F_{7/2}$>	67	11182.5	0.92|$^6F_{7/2}$>
68	11139.4	0.88|$^6F_{7/2}$>	68	11182.5	0.92|$^6F_{7/2}$>
69	11227.5	0.92|$^6F_{7/2}$>	69	11326.5	0.95|$^6F_{7/2}$>
70	11227.5	0.92|$^6F_{7/2}$>	70	11326.5	0.95|$^6F_{7/2}$>
71	12726.9	0.53|$^6F_{9/2}$> + 0.39|$^6H_{15/2}$>	71	12859.6	0.64|$^6H_{15/2}$> + 0.28|$^6F_{9/2}$>
72	12726.9	0.53|$^6F_{9/2}$> + 0.39|$^6H_{15/2}$>	72	12859.6	0.64|$^6H_{15/2}$> + 0.28|$^6F_{9/2}$>
73	12832.8	0.50|$^6F_{9/2}$> + 0.43|$^6H_{15/2}$>	73	12963.8	0.63|$^6F_{9/2}$> + 0.32|$^6H_{15/2}$>
74	12832.8	0.50|$^6F_{9/2}$> + 0.43|$^6H_{15/2}$>	74	12963.8	0.63|$^6F_{9/2}$> + 0.32|$^6H_{15/2}$>
75	13133.5	0.68|$^6F_{9/2}$> + 0.26|$^6H_{15/2}$>	75	13147.8	0.72|$^6F_{9/2}$> + 0.23|$^6H_{15/2}$>
76	13133.5	0.68|$^6F_{9/2}$> + 0.26|$^6H_{15/2}$>	76	13147.8	0.72|$^6F_{9/2}$> + 0.23|$^6H_{15/2}$>
77	13195.2	0.76|$^6F_{9/2}$> + 0.20|$^6H_{15/2}$>	77	13280.9	0.75|$^6F_{9/2}$> + 0.21|$^6H_{15/2}$>
78	13195.2	0.76|$^6F_{9/2}$> + 0.20|$^6H_{15/2}$>	78	13280.9	0.75|$^6F_{9/2}$> + 0.21|$^6H_{15/2}$>
79	13337.1	0.60|$^6F_{9/2}$> + 0.34|$^6H_{15/2}$>	79	13396.3	0.66|$^6F_{9/2}$> + 0.30|$^6H_{15/2}$>
80	13337.1	0.60|$^6F_{9/2}$> + 0.34|$^6H_{15/2}$>	80	13396.3	0.66|$^6F_{9/2}$> + 0.30|$^6H_{15/2}$>
81	13487.1	0.56|$^6H_{15/2}$> + 0.35|$^6F_{9/2}$>	81	13489.2	0.65|$^6H_{15/2}$> + 0.29|$^6F_{9/2}$>
82	13487.1	0.56|$^6H_{15/2}$> + 0.35|$^6F_{9/2}$>	82	13489.2	0.65|$^6H_{15/2}$> + 0.29|$^6F_{9/2}$>
83	13571.1	0.57|$^6F_{9/2}$> + 0.38|$^6H_{15/2}$>	83	13584.7	0.50|$^6F_{9/2}$> + 0.45|$^6H_{15/2}$>
84	13571.1	0.57|$^6F_{9/2}$> + 0.38|$^6H_{15/2}$>	84	13584.7	0.50|$^6F_{9/2}$> + 0.45|$^6H_{15/2}$>

85	13738.0	0.67|$^6H_{15/2}$> + 0.27|$^6F_{9/2}$>	85	13649.2	0.55|$^6H_{15/2}$> + 0.39|$^6F_{9/2}$>
86	13738.0	0.67|$^6H_{15/2}$> + 0.27|$^6F_{9/2}$>	86	13649.2	0.55|$^6H_{15/2}$> + 0.39|$^6F_{9/2}$>
87	13857.3	0.72|$^6H_{15/2}$> + 0.23|$^6F_{9/2}$>	87	13786.7	0.57|$^6H_{15/2}$> + 0.37|$^6F_{9/2}$>
88	13857.3	0.72|$^6H_{15/2}$> + 0.23|$^6F_{9/2}$>	88	13786.7	0.57|$^6H_{15/2}$> + 0.37|$^6F_{9/2}$>
89	13939.0	0.80|$^6H_{15/2}$> + 0.14|$^6F_{9/2}$>	89	13932.1	0.73|$^6H_{15/2}$> + 0.21|$^6F_{9/2}$>
90	13939.0	0.80|$^6H_{15/2}$> + 0.14|$^6F_{9/2}$>	90	13932.1	0.73|$^6H_{15/2}$> + 0.21|$^6F_{9/2}$>
91	14037.2	0.78|$^6H_{15/2}$> + 0.15|$^6F_{9/2}$>	91	14140.3	0.10|$^6H_{13/2}$> + 0.92|$^6H_{15/2}$>
92	14037.2	0.78|$^6H_{15/2}$> + 0.15|$^6F_{9/2}$>	92	14140.3	0.10|$^6H_{13/2}$> + 0.92|$^6H_{15/2}$>
93	14210.2	0.89|$^6H_{15/2}$>	93	14453.0	0.88|$^6H_{15/2}$>
94	14210.2	0.89|$^6H_{15/2}$>	94	14453.0	0.88|$^6H_{15/2}$>
95	14543.5	0.89|$^6H_{15/2}$>	95	14658.4	0.94|$^6H_{15/2}$>
96	14543.5	0.89|$^6H_{15/2}$>	96	14658.4	0.94|$^6H_{15/2}$>
97	15710.6	0.93|$^6F_{11/2}$>	97	15663.2	0.94|$^6F_{11/2}$>
98	15710.6	0.93|$^6F_{11/2}$>	98	15663.2	0.94|$^6F_{11/2}$>
99	15841.8	0.96|$^6F_{11/2}$>	99	15933.2	0.93|$^6F_{11/2}$>
100	15841.8	0.96|$^6F_{11/2}$>	100	15933.2	0.93|$^6F_{11/2}$>
101	15977.7	0.95|$^6F_{11/2}$>	101	16057.5	0.94|$^6F_{11/2}$>
102	15977.7	0.95|$^6F_{11/2}$>	102	16057.5	0.94|$^6F_{11/2}$>
103	16089.9	0.92|$^6F_{11/2}$>	103	16141.7	0.94|$^6F_{11/2}$>
104	16089.9	0.92|$^6F_{11/2}$>	104	16141.7	0.94|$^6F_{11/2}$>
105	16187.7	0.94|$^6F_{11/2}$>	105	16241.3	0.96|$^6F_{11/2}$>
106	16187.7	0.94|$^6F_{11/2}$>	106	16241.3	0.96|$^6F_{11/2}$>
107	16322.1	0.97|$^6F_{11/2}$>	107	16372.7	0.97|$^6F_{11/2}$>
108	16322.1	0.97|$^6F_{11/2}$>	108	16372.7	0.97|$^6F_{11/2}$>

Table S22. State energies and composition of the Pu complexes computed with MS-CASPT2-SO.

2Pu			3Pu		
State	Energy (cm^{-1})	Composition	State	Energy (cm^{-1})	Composition
1	0.0	$0.98\|^6H_{5/2}>$	1	0.0	$0.96\|^6H_{5/2}>$
2	0.0	$0.98\|^6H_{5/2}>$	2	0.0	$0.96\|^6H_{5/2}>$
3	233.3	$0.95\|^6H_{5/2}>$	3	458.6	$0.95\|^6H_{5/2}>$
4	233.3	$0.95\|^6H_{5/2}>$	4	458.6	$0.95\|^6H_{5/2}>$
5	575.4	$0.93\|^6H_{5/2}>$	5	1002.0	$0.93\|^6H_{5/2}>$
6	575.4	$0.93\|^6H_{5/2}>$	6	1002.0	$0.93\|^6H_{5/2}>$
7	1568.3	$0.92\|^6H_{7/2}>$	7	1694.6	$0.94\|^6H_{7/2}>$
8	1568.3	$0.92\|^6H_{7/2}>$	8	1694.6	$0.94\|^6H_{7/2}>$
9	1985.3	$0.95\|^6H_{7/2}>$	9	2171.7	$0.90\|^6H_{7/2}> + 0.11\|^6H_{5/2}>$
10	1985.3	$0.95\|^6H_{7/2}>$	10	2171.7	$0.90\|^6H_{7/2}> + 0.11\|^6H_{5/2}>$
11	2127.2	$0.89\|^6H_{7/2}>$	11	2444.1	$0.87\|^6H_{7/2}> + 0.12\|^6H_{5/2}>$
12	2127.2	$0.89\|^6H_{7/2}>$	12	2444.1	$0.87\|^6H_{7/2}> + 0.12\|^6H_{5/2}>$
13	2407.3	$0.93\|^6H_{7/2}>$	13	2697.5	$0.93\|^6H_{7/2}>$
14	2407.3	$0.93\|^6H_{7/2}>$	14	2697.5	$0.93\|^6H_{7/2}>$
15	3690.4	$0.80\|^6H_{7/2}>$	15	3903.4	$0.91\|^6H_{7/2}>$
16	3690.4	$0.80\|^6H_{7/2}>$	16	3903.4	$0.91\|^6H_{7/2}>$
17	3899.5	$0.90\|^6H_{7/2}>$	17	4166.6	$0.87\|^6H_{7/2}> + 0.11\|^6H_{7/2}>$
18	3899.5	$0.90\|^6H_{7/2}>$	18	4166.6	$0.87\|^6H_{7/2}> + 0.11\|^6H_{7/2}>$
19	4188.8	$0.92\|^6H_{7/2}>$	19	4439.2	$0.89\|^6H_{7/2}>$
20	4188.8	$0.92\|^6H_{7/2}>$	20	4439.2	$0.89\|^6H_{7/2}>$
21	4380.5	$0.91\|^6H_{7/2}>$	21	4606.6	$0.90\|^6H_{7/2}>$
22	4380.5	$0.91\|^6H_{7/2}>$	22	4606.6	$0.90\|^6H_{7/2}>$
23	4547.6	$0.92\|^6H_{7/2}>$	23	4924.3	$0.94\|^6H_{7/2}>$
24	4547.6	$0.92\|^6H_{7/2}>$	24	4924.3	$0.94\|^6H_{7/2}>$
25	5249.7	$0.74\|^6F_{1/2}> + 0.12\|^6H_{11/2}> + 0.10\|^6H_{7/2}>$	25	5610.4	$0.83\|^6F_{1/2}>$
26	5249.7	$0.74\|^6F_{1/2}> + 0.12\|^6H_{11/2}> + 0.10\|^6H_{7/2}>$	26	5610.4	$0.83\|^6F_{1/2}>$

27	5769.3	$0.62|^6F_{3/2}> + 0.25|^6H_{11/2}>$	27	6227.5	$0.49|^6F_{3/2}> + 0.39|^6H_{11/2}>$
28	5769.3	$0.62|^6F_{3/2}> + 0.25|^6H_{11/2}>$	28	6227.5	$0.49|^6F_{3/2}> + 0.39|^6H_{11/2}>$
29	6184.1	$0.65|^6F_{3/2}> + 0.21|^6H_{11/2}>$	29	6389.4	$0.72|^6F_{3/2}> + 0.20|^6H_{11/2}>$
30	6184.1	$0.65|^6F_{3/2}> + 0.21|^6H_{11/2}>$	30	6389.4	$0.72|^6F_{3/2}> + 0.20|^6H_{11/2}>$
31	6451.7	$0.68|^6H_{11/2}> + 0.14|^6F_{3/2}>$	31	6613.0	$0.55|^6H_{11/2}> + 0.31|^6F_{3/2}>$
32	6451.7	$0.68|^6H_{11/2}> + 0.14|^6F_{3/2}>$	32	6613.0	$0.55|^6H_{11/2}> + 0.31|^6F_{3/2}>$
33	6716.7	$0.57|^6H_{11/2}> + 0.17|^6F_{5/2}> + 0.16|^6F_{3/2}>$	33	6879.4	$0.67|^6H_{11/2}> + 0.18|^6F_{3/2}>$
34	6716.7	$0.57|^6H_{11/2}> + 0.17|^6F_{5/2}> + 0.16|^6F_{3/2}>$	34	6879.4	$0.67|^6H_{11/2}> + 0.18|^6F_{3/2}>$
35	6789.5	$0.68|^6H_{11/2}> + 0.13|^6F_{5/2}>$	35	7046.9	$0.75|^6H_{11/2}>$
36	6789.5	$0.68|^6H_{11/2}> + 0.13|^6F_{5/2}>$	36	7046.9	$0.75|^6H_{11/2}>$
37	6905.4	$0.76|^6H_{11/2}> + 0.11|^6F_{5/2}>$	37	7149.5	$0.75|^6H_{11/2}> + 0.11|^6F_{5/2}>$
38	6905.4	$0.76|^6H_{11/2}> + 0.11|^6F_{5/2}>$	38	7149.5	$0.75|^6H_{11/2}> + 0.11|^6F_{5/2}>$
39	7015.9	$0.65|^6H_{11/2}> + 0.16|^6F_{5/2}>$	39	7232.8	$0.79|^6H_{11/2}>$
40	7015.9	$0.65|^6H_{11/2}> + 0.16|^6F_{5/2}>$	40	7232.8	$0.79|^6H_{11/2}>$
41	7185.0	$0.53|^6H_{11/2}> + 0.36|^6F_{5/2}>$	41	7637.8	$0.60|^6H_{11/2}> + 0.33|^6F_{5/2}>$
42	7185.0	$0.53|^6H_{11/2}> + 0.36|^6F_{5/2}>$	42	7637.8	$0.60|^6H_{11/2}> + 0.33|^6F_{5/2}>$
43	7368.2	$0.46|^6F_{5/2}> + 0.45|^6H_{11/2}>$	43	7691.3	$0.76|^6F_{5/2}> + 0.18|^6H_{11/2}>$
44	7368.2	$0.46|^6F_{5/2}> + 0.45|^6H_{11/2}>$	44	7691.3	$0.76|^6F_{5/2}> + 0.18|^6H_{11/2}>$
45	7595.7	$0.65|^6F_{5/2}> + 0.23|^6H_{11/2}>$	45	7743.8	$0.75|^6F_{5/2}> + 0.18|^6H_{11/2}>$
46	7595.7	$0.65|^6F_{5/2}> + 0.23|^6H_{11/2}>$	46	7743.8	$0.75|^6F_{5/2}> + 0.18|^6H_{11/2}>$
47	7734.4	$0.57|^6F_{5/2}> + 0.35|^6H_{11/2}>$	47	7924.0	$0.58|^6F_{5/2}> + 0.36|^6H_{11/2}>$
48	7734.4	$0.57|^6F_{5/2}> + 0.35|^6H_{11/2}>$	48	7924.0	$0.58|^6F_{5/2}> + 0.36|^6H_{11/2}>$
49	8843.9	$0.80|^6F_{7/2}>$	49	9143.5	$0.78|^6F_{7/2}> + 0.14|^6H_{13/2}>$
50	8843.9	$0.80|^6F_{7/2}>$	50	9143.5	$0.78|^6F_{7/2}> + 0.14|^6H_{13/2}>$
51	8926.9	$0.75|^6F_{7/2}> + 0.13|^6H_{13/2}>$	51	9283.0	$0.81|^6F_{7/2}> + 0.12|^6H_{13/2}>$
52	8926.9	$0.75|^6F_{7/2}> + 0.13|^6H_{13/2}>$	52	9283.0	$0.81|^6F_{7/2}> + 0.12|^6H_{13/2}>$
53	9046.0	$0.76|^6F_{7/2}> + 0.10|^6H_{13/2}>$	53	9334.8	$0.83|^6F_{7/2}>$
54	9046.0	$0.76|^6F_{7/2}> + 0.10|^6H_{13/2}>$	54	9334.8	$0.83|^6F_{7/2}>$
55	9179.5	$0.84|^6F_{7/2}>$	55	9495.5	$0.68|^6F_{7/2}> + 0.21|^6H_{13/2}>$

56	9179.5	0.84|$^6F_{7/2}$>	56	9495.5	0.68|$^6F_{7/2}$> + 0.21|$^6H_{13/2}$>
57	9554.0	0.66|$^6H_{13/2}$> + 0.16|$^6F_{7/2}$>	57	9712.1	0.56|$^6H_{13/2}$> + 0.30|$^6F_{7/2}$>
58	9554.0	0.66|$^6H_{13/2}$> + 0.16|$^6F_{7/2}$>	58	9712.1	0.56|$^6H_{13/2}$> + 0.30|$^6F_{7/2}$>
59	9731.4	0.65|$^6H_{13/2}$> + 0.18|$^6F_{9/2}$>	59	9836.3	0.69|$^6H_{13/2}$> + 0.17|$^6F_{7/2}$>
60	9731.4	0.65|$^6H_{13/2}$> + 0.18|$^6F_{9/2}$>	60	9836.3	0.69|$^6H_{13/2}$> + 0.17|$^6F_{7/2}$>
61	9911.0	0.74|$^6H_{13/2}$> + 0.15|$^6F_{9/2}$>	61	10068.8	0.82|$^6H_{13/2}$>
62	9911.0	0.74|$^6H_{13/2}$> + 0.15|$^6F_{9/2}$>	62	10068.8	0.82|$^6H_{13/2}$>
63	10072.5	0.85|$^6H_{13/2}$>	63	10257.1	0.82|$^6H_{13/2}$>
64	10072.5	0.85|$^6H_{13/2}$>	64	10257.1	0.82|$^6H_{13/2}$>
65	10135.5	0.85|$^6H_{13/2}$>	65	10370.1	0.80|$^6H_{13/2}$>
66	10135.5	0.85|$^6H_{13/2}$>	66	10370.1	0.80|$^6H_{13/2}$>
67	10297.8	0.77|$^6H_{13/2}$>	67	10510.0	0.85|$^6H_{13/2}$>
68	10297.8	0.77|$^6H_{13/2}$>	68	10510.0	0.85|$^6H_{13/2}$>
69	10477.5	0.83|$^6H_{13/2}$>	69	10891.2	0.85|$^6H_{13/2}$>
70	10477.5	0.83|$^6H_{13/2}$>	70	10891.2	0.85|$^6H_{13/2}$>
71	11046.2	0.76|$^6F_{9/2}$> + 0.13|$^6H_{13/2}$>	71	11436.8	0.82|$^6F_{9/2}$> + 0.13|$^6H_{13/2}$>
72	11046.2	0.76|$^6F_{9/2}$> + 0.13|$^6H_{13/2}$>	72	11436.8	0.82|$^6F_{9/2}$> + 0.13|$^6H_{13/2}$>
73	11208.4	0.71|$^6F_{9/2}$> + 0.18|$^6H_{13/2}$>	73	11499.2	0.79|$^6F_{9/2}$> + 0.14|$^6H_{13/2}$>
74	11208.4	0.71|$^6F_{9/2}$> + 0.18|$^6H_{13/2}$>	74	11499.2	0.79|$^6F_{9/2}$> + 0.14|$^6H_{13/2}$>
75	11372.0	0.78|$^6F_{9/2}$> + 0.18|$^6H_{13/2}$>	75	11619.6	0.83|$^6F_{9/2}$> + 0.11|$^6H_{13/2}$>
76	11372.0	0.78|$^6F_{9/2}$> + 0.18|$^6H_{13/2}$>	76	11619.6	0.83|$^6F_{9/2}$> + 0.11|$^6H_{13/2}$>
77	11448.5	0.86|$^6F_{9/2}$> + 0.10|$^6H_{13/2}$>	77	11746.0	0.85|$^6F_{9/2}$> + 0.11|$^6H_{13/2}$>
78	11448.5	0.86|$^6F_{9/2}$> + 0.10|$^6H_{13/2}$>	78	11746.0	0.85|$^6F_{9/2}$> + 0.11|$^6H_{13/2}$>
79	11544.5	0.84|$^6F_{9/2}$> + 0.11|$^6H_{13/2}$>	79	11824.5	0.87|$^6F_{9/2}$>
80	11544.5	0.84|$^6F_{9/2}$> + 0.11|$^6H_{13/2}$>	80	11824.5	0.87|$^6F_{9/2}$>
81	12802.6	0.52|$^6H_{15/2}$> + 0.39|$^6F_{11/2}$> +0.11|$^6H_{13/2}$>	81	12981.1	0.69|$^6H_{15/2}$> + 0.18|$^6F_{11/2}$> + 0.10|$^6H_{13/2}$>
82	12802.6	0.52|$^6H_{15/2}$> + 0.39|$^6F_{11/2}$> +0.11|$^6H_{13/2}$>	82	12981.1	0.69|$^6H_{15/2}$> + 0.18|$^6F_{11/2}$> + 0.10|$^6H_{13/2}$>
83	13230.2	0.50|$^6H_{15/2}$> + 0.41|$^6F_{11/2}$>	83	13228.2	0.57|$^6H_{15/2}$> + 0.35|$^6F_{11/2}$>
84	13230.2	0.50|$^6H_{15/2}$> + 0.41|$^6F_{11/2}$>	84	13228.2	0.57|$^6H_{15/2}$> + 0.35|$^6F_{11/2}$>

85	13308.9	0.53|$^6H_{15/2}$> + 0.38|$^6F_{11/2}$>	85	13381.9	0.65|$^6H_{15/2}$> + 0.26|$^6F_{11/2}$>
86	13308.9	0.53|$^6H_{15/2}$> + 0.38|$^6F_{11/2}$>	86	13381.9	0.65|$^6H_{15/2}$> + 0.26|$^6F_{11/2}$>
87	13420.9	0.47|$^6H_{15/2}$> + 0.46|$^6F_{11/2}$>	87	13529.1	0.68|$^6H_{15/2}$> + 0.24|$^6F_{11/2}$>
88	13420.9	0.47|$^6H_{15/2}$> + 0.46|$^6F_{11/2}$>	88	13529.1	0.68|$^6H_{15/2}$> + 0.24|$^6F_{11/2}$>
89	13569.1	0.62|$^6H_{15/2}$> + 0.30|$^6F_{11/2}$>	89	13702.6	0.54|$^6H_{15/2}$> + 0.40|$^6F_{11/2}$>
90	13569.1	0.62|$^6H_{15/2}$> + 0.30|$^6F_{11/2}$>	90	13702.6	0.54|$^6H_{15/2}$> + 0.40|$^6F_{11/2}$>
91	13634.2	0.73|$^6H_{15/2}$> + 0.19|$^6F_{11/2}$>	91	13780.1	0.72|$^6F_{11/2}$> + 0.25|$^6H_{15/2}$>
92	13634.2	0.73|$^6H_{15/2}$> + 0.19|$^6F_{11/2}$>	92	13780.1	0.72|$^6F_{11/2}$> + 0.25|$^6H_{15/2}$>
93	13797.8	0.54|$^6H_{15/2}$> + 0.40|$^6F_{11/2}$>	93	14142.3	0.84|$^6H_{15/2}$> + 0.11|$^6F_{11/2}$> + 0.10|$^6H_{13/2}$>
94	13797.8	0.54|$^6H_{15/2}$> + 0.40|$^6F_{11/2}$>	94	14142.3	0.84|$^6H_{15/2}$> + 0.11|$^6F_{11/2}$> + 0.10|$^6H_{13/2}$>
95	13996.8	0.55|$^6F_{11/2}$> + 0.41|$^6H_{15/2}$>	95	14302.7	0.64|$^6F_{11/2}$> + 0.34|$^6H_{15/2}$>
96	13996.8	0.55|$^6F_{11/2}$> + 0.41|$^6H_{15/2}$>	96	14302.7	0.64|$^6F_{11/2}$> + 0.34|$^6H_{15/2}$>
97	14154.1	0.52|$^6F_{11/2}$> + 0.44|$^6H_{15/2}$>	97	14516.5	0.65|$^6F_{11/2}$> + 0.33|$^6H_{15/2}$>
98	14154.1	0.52|$^6F_{11/2}$> + 0.44|$^6H_{15/2}$>	98	14516.5	0.65|$^6F_{11/2}$> + 0.33|$^6H_{15/2}$>
99	14207.8	0.58|$^6H_{15/2}$> + 0.36|$^6F_{11/2}$>	99	14573.3	0.58|$^6F_{11/2}$> + 0.40|$^6H_{15/2}$>
100	14207.8	0.58|$^6H_{15/2}$> + 0.36|$^6F_{11/2}$>	100	14573.3	0.58|$^6F_{11/2}$> + 0.40|$^6H_{15/2}$>
101	14444.0	0.60|$^6F_{11/2}$> + 0.36|$^6H_{15/2}$>	101	14630.2	0.52|$^6H_{15/2}$> + 0.45|$^6F_{11/2}$>
102	14444.0	0.60|$^6F_{11/2}$> + 0.36|$^6H_{15/2}$>	102	14630.2	0.52|$^6H_{15/2}$> + 0.45|$^6F_{11/2}$>
103	14562.0	0.50|$^6F_{11/2}$> + 0.46|$^6H_{15/2}$>	103	14730.4	0.54|$^6F_{11/2}$> + 0.44|$^6H_{15/2}$>
104	14562.0	0.50|$^6F_{11/2}$> + 0.46|$^6H_{15/2}$>	104	14730.4	0.54|$^6F_{11/2}$> + 0.44|$^6H_{15/2}$>
105	14784.7	0.71|$^6H_{15/2}$> + 0.26|$^6F_{11/2}$>	105	14895.8	0.56|$^6H_{15/2}$> + 0.42|$^6F_{11/2}$>
106	14784.7	0.71|$^6H_{15/2}$> + 0.26|$^6F_{11/2}$>	106	14895.8	0.56|$^6H_{15/2}$> + 0.42|$^6F_{11/2}$>
107	14943.6	0.49|$^6H_{15/2}$> + 0.48|$^6F_{11/2}$>	107	14989.7	0.66|$^6H_{15/2}$> + 0.31|$^6F_{11/2}$>
108	14943.6	0.49|$^6H_{15/2}$> + 0.48|$^6F_{11/2}$>	108	14989.7	0.66|$^6H_{15/2}$> + 0.31|$^6F_{11/2}$>

Table S23. State energies and composition of the Pu complexes computed with XMS-CASPT2-SO.

2Pu			3Pu		
**State**	**Energy (cm^{-1})**	**Composition**	**State**	**Energy (cm^{-1})**	**Composition**
1	0	$0.95\vert^{6}H_{5/2}>$	1	0.0	$0.96\vert^{6}H_{5/2}>$
2	0	$0.95\vert^{6}H_{5/2}>$	2	0.0	$0.96\vert^{6}H_{5/2}>$
3	419.62	$0.93\vert^{6}H_{5/2}>$	3	445.1	$0.91\vert^{6}H_{5/2}>$
4	419.62	$0.93\vert^{6}H_{5/2}>$	4	445.1	$0.91\vert^{6}H_{5/2}>$
5	769.9	$0.84\vert^{6}H_{5/2}>$	5	1166.3	$0.82\vert^{6}H_{5/2}>$
6	769.9	$0.84\vert^{6}H_{5/2}>$	6	1166.3	$0.82\vert^{6}H_{5/2}>$
7	1629.49	$0.88\vert^{6}H_{7/2}> + 0.13\vert^{6}H_{5/2}>$	7	1719.9	$0.89\vert^{6}H_{7/2}> + 0.11\vert^{6}H_{5/2}>$
8	1629.49	$0.88\vert^{6}H_{7/2}> + 0.13\vert^{6}H_{5/2}>$	8	1719.9	$0.89\vert^{6}H_{7/2}> + 0.11\vert^{6}H_{5/2}>$
9	2076.38	$0.90\vert^{6}H_{7/2}>$	9	2268.6	$0.84\vert^{6}H_{7/2}> + 0.15\vert^{6}H_{5/2}>$
10	2076.38	$0.90\vert^{6}H_{7/2}>$	10	2268.6	$0.84\vert^{6}H_{7/2}> + 0.15\vert^{6}H_{5/2}>$
11	2405.7	$0.82\vert^{6}H_{7/2}> + 0.13\vert^{6}H_{5/2}>$	11	2563.1	$0.78\vert^{6}H_{7/2}> + 0.17\vert^{6}H_{5/2}>$
12	2405.7	$0.82\vert^{6}H_{7/2}> + 0.13\vert^{6}H_{5/2}>$	12	2563.1	$0.78\vert^{6}H_{7/2}> + 0.17\vert^{6}H_{5/2}>$
13	2819.13	$0.88\vert^{6}H_{7/2}>$	13	2900.6	$0.88\vert^{6}H_{7/2}>$
14	2819.13	$0.88\vert^{6}H_{7/2}>$	14	2900.6	$0.88\vert^{6}H_{7/2}>$
15	3821.45	$0.84\vert^{6}H_{7/2}> + 0.10\vert^{6}H_{7/2}>$	15	3954.4	$0.87\vert^{6}H_{7/2}> + 0.11\vert^{6}H_{7/2}>$
16	3821.45	$0.84\vert^{6}H_{7/2}> + 0.10\vert^{6}H_{7/2}>$	16	3954.4	$0.87\vert^{6}H_{7/2}> + 0.11\vert^{6}H_{7/2}>$
17	4063.24	$0.84\vert^{6}H_{7/2}> + 0.13\vert^{6}H_{7/2}>$	17	4207.5	$0.86\vert^{6}H_{7/2}> + 0.11\vert^{6}H_{7/2}>$
18	4063.24	$0.84\vert^{6}H_{7/2}> + 0.13\vert^{6}H_{7/2}>$	18	4207.5	$0.86\vert^{6}H_{7/2}> + 0.11\vert^{6}H_{7/2}>$
19	4460.34	$0.89\vert^{6}H_{7/2}>$	19	4584.5	$0.81\vert^{6}H_{7/2}> + 0.14\vert^{6}H_{7/2}>$
20	4460.34	$0.89\vert^{6}H_{7/2}>$	20	4584.5	$0.81\vert^{6}H_{7/2}> + 0.14\vert^{6}H_{7/2}>$
21	4702.46	$0.87\vert^{6}H_{7/2}>$	21	4696.9	$0.84\vert^{6}H_{7/2}> + 0.11\vert^{6}H_{7/2}>$
22	4702.46	$0.87\vert^{6}H_{7/2}>$	22	4696.9	$0.84\vert^{6}H_{7/2}> + 0.11\vert^{6}H_{7/2}>$
23	4948.8	$0.88\vert^{6}H_{7/2}>$	23	5164.2	$0.92\vert^{6}H_{7/2}>$
24	4948.8	$0.88\vert^{6}H_{7/2}>$	24	5164.2	$0.92\vert^{6}H_{7/2}>$
25	5915.85	$0.47\vert^{6}F_{1/2}> + 0.35\vert^{6}H_{11/2}> + 0.12\vert^{6}H_{7/2}>$	25	6176.6	$0.54\vert^{6}F_{1/2}> + 0.27\vert^{6}H_{11/2}>$
26	5915.85	$0.47\vert^{6}F_{1/2}> + 0.35\vert^{6}H_{11/2}> + 0.12\vert^{6}H_{7/2}>$	26	6176.6	$0.54\vert^{6}F_{1/2}> + 0.27\vert^{6}H_{11/2}>$

27	6328.04	$0.49\|^{6}H_{11/2}> + 0.26\|^{6}F_{3/2}> + 0.11\|^{6}F_{1/2}>$	27	6412.5	$0.63\|^{6}H_{11/2}> + 0.11\|^{6}H_{7/2}>$
28	6328.04	$0.49\|^{6}H_{11/2}> + 0.26\|^{6}F_{3/2}> + 0.11\|^{6}F_{1/2}>$	28	6412.5	$0.63\|^{6}H_{11/2}> + 0.11\|^{6}H_{7/2}>$
29	6626.67	$0.69\|^{6}H_{11/2}> + 0.12\|^{6}F_{3/2}> + 0.10\|^{6}H_{7/2}>$	29	6718.0	$0.78\|^{6}H_{11/2}>$
30	6626.67	$0.69\|^{6}H_{11/2}> + 0.12\|^{6}F_{3/2}> + 0.10\|^{6}H_{7/2}>$	30	6718.0	$0.78\|^{6}H_{11/2}>$
31	6899.77	$0.53\|^{6}H_{11/2}> + 0.23\|^{6}F_{3/2}> + 0.14\|^{6}F_{1/2}>$	31	6973.8	$0.51\|^{6}H_{11/2}> + 0.29\|^{6}F_{3/2}>$
32	6899.77	$0.53\|^{6}H_{11/2}> + 0.23\|^{6}F_{3/2}> + 0.14\|^{6}F_{1/2}>$	32	6973.8	$0.51\|^{6}H_{11/2}> + 0.29\|^{6}F_{3/2}>$
33	7066.13	$0.69\|^{6}H_{11/2}> + 0.11\|^{6}F_{3/2}>$	33	7150.3	$0.63\|^{6}H_{11/2}> + 0.24\|^{6}F_{3/2}>$
34	7066.13	$0.69\|^{6}H_{11/2}> + 0.11\|^{6}F_{3/2}>$	34	7150.3	$0.63\|^{6}H_{11/2}> + 0.24\|^{6}F_{3/2}>$
35	7230.68	$0.61\|^{6}H_{11/2}> + 0.29\|^{6}F_{3/2}>$	35	7415.9	$0.53\|^{6}H_{11/2}> + 0.29\|^{6}F_{3/2}>$
36	7230.68	$0.61\|^{6}H_{11/2}> + 0.29\|^{6}F_{3/2}>$	36	7415.9	$0.53\|^{6}H_{11/2}> + 0.29\|^{6}F_{3/2}>$
37	7427.71	$0.49\|^{6}H_{11/2}> + 0.36\|^{6}F_{3/2}>$	37	7559.7	$0.53\|^{6}H_{11/2}> + 0.33\|^{6}F_{3/2}>$
38	7427.71	$0.49\|^{6}H_{11/2}> + 0.36\|^{6}F_{3/2}>$	38	7559.7	$0.53\|^{6}H_{11/2}> + 0.33\|^{6}F_{3/2}>$
39	7598.54	$0.42\|^{6}H_{11/2}> + 0.26\|^{6}F_{3/2}> + 0.14\|^{6}F_{5/2}>$	39	7686.7	$0.43\|^{6}H_{11/2}> + 0.24\|^{6}F_{3/2}> + 0.15\|^{6}F_{1/2}>$
40	7598.54	$0.42\|^{6}H_{11/2}> + 0.26\|^{6}F_{3/2}> + 0.14\|^{6}F_{5/2}>$	40	7686.7	$0.43\|^{6}H_{11/2}> + 0.24\|^{6}F_{3/2}> + 0.15\|^{6}F_{1/2}>$
41	7798.79	$0.71\|^{6}H_{11/2}>$	41	8056.8	$0.85\|^{6}H_{11/2}>$
42	7798.79	$0.71\|^{6}H_{11/2}>$	42	8056.8	$0.85\|^{6}H_{11/2}>$
43	8183.81	$0.66\|^{6}F_{5/2}> + 0.16\|^{6}H_{11/2}>$	43	8400.4	$0.69\|^{6}F_{5/2}>$
44	8183.81	$0.66\|^{6}F_{5/2}> + 0.16\|^{6}H_{11/2}>$	44	8400.4	$0.69\|^{6}F_{5/2}>$
45	8458.01	$0.71\|^{6}F_{5/2}> + 0.12\|^{6}H_{11/2}>$	45	8581.8	$0.71\|^{6}F_{5/2}> + 0.10\|^{6}H_{11/2}>$
46	8458.01	$0.71\|^{6}F_{5/2}> + 0.12\|^{6}H_{11/2}>$	46	8581.8	$0.71\|^{6}F_{5/2}> + 0.10\|^{6}H_{11/2}>$
47	8528.41	$0.78\|^{6}F_{5/2}>$	47	8809.8	$0.79\|^{6}F_{5/2}>$
48	8528.41	$0.78\|^{6}F_{5/2}>$	48	8809.8	$0.79\|^{6}F_{5/2}>$
49	9400.29	$0.57\|^{6}H_{13/2}> + 0.24\|^{6}F_{7/2}>$	49	9464.0	$0.69\|^{6}H_{13/2}> + 0.11\|^{6}F_{7/2}>$
50	9400.29	$0.57\|^{6}H_{13/2}> + 0.24\|^{6}F_{7/2}>$	50	9464.0	$0.69\|^{6}H_{13/2}> + 0.11\|^{6}F_{7/2}>$
51	9612.46	$0.46\|^{6}F_{7/2}> + 0.34\|^{6}H_{13/2}>$	51	9717.6	$0.53\|^{6}F_{7/2}> + 0.27\|^{6}H_{13/2}>$
52	9612.46	$0.46\|^{6}F_{7/2}> + 0.34\|^{6}H_{13/2}>$	52	9717.6	$0.53\|^{6}F_{7/2}> + 0.27\|^{6}H_{13/2}>$
53	9840.88	$0.57\|^{6}H_{13/2}> + 0.25\|^{6}F_{7/2}>$	53	9941.7	$0.60\|^{6}H_{13/2}> + 0.25\|^{6}F_{7/2}>$
54	9840.88	$0.57\|^{6}H_{13/2}> + 0.25\|^{6}F_{7/2}>$	54	9941.7	$0.60\|^{6}H_{13/2}> + 0.25\|^{6}F_{7/2}>$
55	9957.82	$0.63\|^{6}F_{7/2}> + 0.25\|^{6}H_{13/2}>$	55	10104.1	$0.58\|^{6}H_{13/2}> + 0.26\|^{6}F_{7/2}>$

56	9957.82	$0.63\vert{}^{6}F_{7/2}> + 0.25\vert{}^{6}H_{13/2}>$	56	10104.1	$0.58\vert{}^{6}H_{13/2}> + 0.26\vert{}^{6}F_{7/2}>$
57	10073.51	$0.52\vert{}^{6}F_{7/2}> + 0.36\vert{}^{6}H_{13/2}>$	57	10268.5	$0.54\vert{}^{6}F_{7/2}> + 0.35\vert{}^{6}H_{13/2}>$
58	10073.51	$0.52\vert{}^{6}F_{7/2}> + 0.36\vert{}^{6}H_{13/2}>$	58	10268.5	$0.54\vert{}^{6}F_{7/2}> + 0.35\vert{}^{6}H_{13/2}>$
59	10247.83	$0.48\vert{}^{6}H_{13/2}> + 0.39\vert{}^{6}F_{7/2}>$	59	10342.6	$0.70\vert{}^{6}H_{13/2}> + 0.17\vert{}^{6}F_{7/2}>$
60	10247.83	$0.48\vert{}^{6}H_{13/2}> + 0.39\vert{}^{6}F_{7/2}>$	60	10342.6	$0.70\vert{}^{6}H_{13/2}> + 0.17\vert{}^{6}F_{7/2}>$
61	10329.01	$0.50\vert{}^{6}H_{13/2}> + 0.35\vert{}^{6}F_{7/2}>$	61	10550.6	$0.55\vert{}^{6}F_{7/2}> + 0.32\vert{}^{6}H_{13/2}>$
62	10329.01	$0.50\vert{}^{6}H_{13/2}> + 0.35\vert{}^{6}F_{7/2}>$	62	10550.6	$0.55\vert{}^{6}F_{7/2}> + 0.32\vert{}^{6}H_{13/2}>$
63	10425.27	$0.67\vert{}^{6}H_{13/2}> + 0.21\vert{}^{6}F_{7/2}>$	63	10636.0	$0.53\vert{}^{6}F_{7/2}> + 0.36\vert{}^{6}H_{13/2}>$
64	10425.27	$0.67\vert{}^{6}H_{13/2}> + 0.21\vert{}^{6}F_{7/2}>$	64	10636.0	$0.53\vert{}^{6}F_{7/2}> + 0.36\vert{}^{6}H_{13/2}>$
65	10604.95	$0.66\vert{}^{6}H_{13/2}> + 0.22\vert{}^{6}F_{7/2}>$	65	10698.6	$0.46\vert{}^{6}H_{13/2}> + 0.43\vert{}^{6}F_{7/2}>$
66	10604.95	$0.66\vert{}^{6}H_{13/2}> + 0.22\vert{}^{6}F_{7/2}>$	66	10698.6	$0.46\vert{}^{6}H_{13/2}> + 0.43\vert{}^{6}F_{7/2}>$
67	10955.61	$0.67\vert{}^{6}H_{13/2}> + 0.15\vert{}^{6}F_{7/2}>$	67	11065.9	$0.78\vert{}^{6}H_{13/2}> + 0.11\vert{}^{6}F_{7/2}>$
68	10955.61	$0.67\vert{}^{6}H_{13/2}> + 0.15\vert{}^{6}F_{7/2}>$	68	11065.9	$0.78\vert{}^{6}H_{13/2}> + 0.11\vert{}^{6}F_{7/2}>$
69	11063.2	$0.76\vert{}^{6}H_{13/2}>$	69	11287.9	$0.86\vert{}^{6}H_{13/2}>$
70	11063.2	$0.76\vert{}^{6}H_{13/2}>$	70	11287.9	$0.86\vert{}^{6}H_{13/2}>$
71	11835.73	$0.70\vert{}^{6}F_{9/2}> + 0.12\vert{}^{6}H_{13/2}> + 0.12\vert{}^{6}H_{15/2}>$	71	11932.4	$0.79\vert{}^{6}F_{9/2}>$
72	11835.73	$0.70\vert{}^{6}F_{9/2}> + 0.12\vert{}^{6}H_{13/2}> + 0.12\vert{}^{6}H_{15/2}>$	72	11932.4	$0.79\vert{}^{6}F_{9/2}>$
73	12048.37	$0.76\vert{}^{6}F_{9/2}>$	73	12291.1	$0.69\vert{}^{6}F_{9/2}> + 0.11\vert{}^{6}H_{15/2}> + 0.10\vert{}^{6}H_{13/2}>$
74	12048.37	$0.76\vert{}^{6}F_{9/2}>$	74	12291.1	$0.69\vert{}^{6}F_{9/2}> + 0.11\vert{}^{6}H_{15/2}> + 0.10\vert{}^{6}H_{13/2}>$
75	12337.69	$0.83\vert{}^{6}F_{9/2}>$	75	12478.5	$0.75\vert{}^{6}F_{9/2}> + 0.11\vert{}^{6}H_{15/2}>$
76	12337.69	$0.83\vert{}^{6}F_{9/2}>$	76	12478.5	$0.75\vert{}^{6}F_{9/2}> + 0.11\vert{}^{6}H_{15/2}>$
77	12482.2	$0.81\vert{}^{6}F_{9/2}>$	77	12683.4	$0.77\vert{}^{6}F_{9/2}> + 0.14\vert{}^{6}H_{15/2}>$
78	12482.2	$0.81\vert{}^{6}F_{9/2}>$	78	12683.4	$0.77\vert{}^{6}F_{9/2}> + 0.14\vert{}^{6}H_{15/2}>$
79	12558.03	$0.80\vert{}^{6}F_{9/2}>$	79	12869.3	$0.80\vert{}^{6}F_{9/2}>$
80	12558.03	$0.80\vert{}^{6}F_{9/2}>$	80	12869.3	$0.80\vert{}^{6}F_{9/2}>$
81	13278.91	$0.69\vert{}^{6}H_{15/2}> + 0.19\vert{}^{6}F_{11/2}> +0.11\vert{}^{6}H_{13/2}>$	81	13340.0	$0.69\vert{}^{6}H_{15/2}> + 0.17\vert{}^{6}F_{9/2}>$
82	13278.91	$0.69\vert{}^{6}H_{15/2}> + 0.19\vert{}^{6}F_{11/2}> +0.11\vert{}^{6}H_{13/2}>$	82	13340.0	$0.69\vert{}^{6}H_{15/2}> + 0.17\vert{}^{6}F_{9/2}>$
83	13551.95	$0.74\vert{}^{6}H_{15/2}> + 0.13\vert{}^{6}H_{13/2}>$	83	13458.5	$0.63\vert{}^{6}H_{15/2}> + 0.16\vert{}^{6}F_{9/2}> + 0.12\vert{}^{6}F_{11/2}>$
84	13551.95	$0.74\vert{}^{6}H_{15/2}> + 0.13\vert{}^{6}H_{13/2}>$	84	13458.5	$0.63\vert{}^{6}H_{15/2}> + 0.16\vert{}^{6}F_{9/2}> + 0.12\vert{}^{6}F_{11/2}>$

85	13802.54	$0.73\|^6H_{15/2}> + 0.14\|^6F_{9/2}>$	85	13601.7	$0.77\|^6H_{15/2}> + 0.11\|^6H_{13/2}>$
86	13802.54	$0.73\|^6H_{15/2}> + 0.14\|^6F_{9/2}>$	86	13601.7	$0.77\|^6H_{15/2}> + 0.11\|^6H_{13/2}>$
87	13926.45	$0.75\|^6H_{15/2}> + 0.13\|^6F_{11/2}>$	87	13966.6	$0.72\|^6H_{15/2}> + 0.13\|^6F_{11/2}>$
88	13926.45	$0.75\|^6H_{15/2}> + 0.13\|^6F_{11/2}>$	88	13966.6	$0.72\|^6H_{15/2}> + 0.13\|^6F_{11/2}>$
89	14035.64	$0.82\|^6H_{15/2}> + 0.11\|^6H_{13/2}>$	89	14133.1	$0.66\|^6H_{15/2}> + 0.20\|^6F_{11/2}>$
90	14035.64	$0.82\|^6H_{15/2}> + 0.11\|^6H_{13/2}>$	90	14133.1	$0.66\|^6H_{15/2}> + 0.20\|^6F_{11/2}>$
91	14195.95	$0.57\|^6H_{15/2}> + 0.30\|^6F_{11/2}>$	91	14375.7	$0.50\|^6H_{15/2}> + 0.41\|^6F_{11/2}>$
92	14195.95	$0.57\|^6H_{15/2}> + 0.30\|^6F_{11/2}>$	92	14375.7	$0.50\|^6H_{15/2}> + 0.41\|^6F_{11/2}>$
93	14534.17	$0.62\|^6H_{15/2}> + 0.29\|^6F_{11/2}>$	93	14616.1	$0.71\|^6H_{15/2}> + 0.23\|^6F_{11/2}>$
94	14534.17	$0.62\|^6H_{15/2}> + 0.29\|^6F_{11/2}>$	94	14616.1	$0.71\|^6H_{15/2}> + 0.23\|^6F_{11/2}>$
95	14756.31	$0.75\|^6F_{11/2}> + 0.21\|^6H_{15/2}>$	95	14808.3	$0.67\|^6H_{15/2}> + 0.28\|^6F_{11/2}>$
96	14756.31	$0.75\|^6F_{11/2}> + 0.21\|^6H_{15/2}>$	96	14808.3	$0.67\|^6H_{15/2}> + 0.28\|^6F_{11/2}>$
97	14964.3	$0.73\|^6F_{11/2}> + 0.22\|^6H_{15/2}>$	97	15037.3	$0.58\|^6F_{11/2}> + 0.39\|^6H_{15/2}>$
98	14964.3	$0.73\|^6F_{11/2}> + 0.22\|^6H_{15/2}>$	98	15037.3	$0.58\|^6F_{11/2}> + 0.39\|^6H_{15/2}>$
99	15150.27	$0.56\|^6F_{11/2}> + 0.40\|^6H_{15/2}>$	99	15292.3	$0.60\|^6F_{11/2}> + 0.36\|^6H_{15/2}>$
100	15150.27	$0.56\|^6F_{11/2}> + 0.40\|^6H_{15/2}>$	100	15292.3	$0.60\|^6F_{11/2}> + 0.36\|^6H_{15/2}>$
101	15265.12	$0.48\|^6F_{11/2}> + 0.48\|^6H_{15/2}>$	101	15546.2	$0.75\|^6F_{11/2}> + 0.20\|^6H_{15/2}>$
102	15265.12	$0.48\|^6F_{11/2}> + 0.48\|^6H_{15/2}>$	102	15546.2	$0.75\|^6F_{11/2}> + 0.20\|^6H_{15/2}>$
103	15359.31	$0.52\|^6F_{11/2}> + 0.44\|^6H_{15/2}>$	103	15679.0	$0.69\|^6F_{11/2}> + 0.27\|^6H_{15/2}>$
104	15359.31	$0.52\|^6F_{11/2}> + 0.44\|^6H_{15/2}>$	104	15679.0	$0.69\|^6F_{11/2}> + 0.27\|^6H_{15/2}>$
105	15561.4	$0.72\|^6F_{11/2}> + 0.24\|^6H_{15/2}>$	105	15811.1	$0.72\|^6F_{11/2}> + 0.25\|^6H_{15/2}>$
106	15561.4	$0.72\|^6F_{11/2}> + 0.24\|^6H_{15/2}>$	106	15811.1	$0.72\|^6F_{11/2}> + 0.25\|^6H_{15/2}>$
107	15833.64	$0.85\|^6F_{11/2}> + 0.12\|^6H_{15/2}>$	107	16019.2	$0.84\|^6F_{11/2}> + 0.14\|^6H_{15/2}>$
108	15833.64	$0.85\|^6F_{11/2}> + 0.12\|^6H_{15/2}>$	108	16019.2	$0.84\|^6F_{11/2}> + 0.14\|^6H_{15/2}>$

Table S24. State energies and composition of the Pu complexes computed with MS-CASPT2-SO with an additional 16 quartet spin-free states.

2Pu			3Pu		
**State**	**Energy (cm^{-1})**	**Composition**	**State**	**Energy (cm^{-1})**	**Composition**
1	0.0	$0.81|^6H_{5/2}>$	1	0.0	$0.86|^6H_{5/2}>$
2	0.0	$0.81|^6H_{5/2}>$	2	0.0	$0.86|^6H_{5/2}>$
3	798.3	$0.86|^6H_{5/2}>$	3	735.0	$0.88|^6H_{5/2}>$
4	798.3	$0.86|^6H_{5/2}>$	4	735.0	$0.88|^6H_{5/2}>$
5	1624.4	$0.83|^6H_{5/2}>$	5	1574.5	$0.65|^6H_{5/2}> + 0.22|^6H_{7/2}>$
6	1624.4	$0.83|^6H_{5/2}>$	6	1574.5	$0.65|^6H_{5/2}> + 0.22|^6H_{7/2}>$
7	2036.2	$0.75|^6H_{7/2}>$	7	2032.6	$0.22|^6H_{5/2}> + 0.71|^6H_{7/2}>$
8	2036.2	$0.75|^6H_{7/2}>$	8	2032.6	$0.22|^6H_{5/2}> + 0.71|^6H_{7/2}>$
9	2842.0	$0.85|^6H_{7/2}>$	9	2784.7	$0.12|^6H_{5/2}> + 0.82|^6H_{7/2}>$
10	2842.0	$0.85|^6H_{7/2}>$	10	2784.7	$0.12|^6H_{5/2}> + 0.82|^6H_{7/2}>$
11	3188.3	$0.82|^6H_{7/2}>$	11	3171.8	$0.19|^6H_{5/2}> + 0.73|^6H_{7/2}>$
12	3188.3	$0.82|^6H_{7/2}>$	12	3171.8	$0.19|^6H_{5/2}> + 0.73|^6H_{7/2}>$
13	3494.5	$0.89|^6H_{7/2}>$	13	3491.8	$0.85|^6H_{7/2}>$
14	3494.5	$0.89|^6H_{7/2}>$	14	3491.8	$0.85|^6H_{7/2}>$
15	4441.7	$0.72|^6H_{7/2}>$	15	4455.0	$0.80|^6H_{7/2}>$
16	4441.7	$0.72|^6H_{7/2}>$	16	4455.0	$0.80|^6H_{7/2}>$
17	4819.2	$0.83|^6H_{7/2}>$	17	4734.0	$0.12|^6H_{7/2}> + 0.79|^6H_{7/2}>$
18	4819.2	$0.83|^6H_{7/2}>$	18	4734.0	$0.12|^6H_{7/2}> + 0.79|^6H_{7/2}>$
19	5161.6	$0.86|^6H_{7/2}>$	19	5111.1	$0.80|^6H_{7/2}>$
20	5161.6	$0.86|^6H_{7/2}>$	20	5111.1	$0.80|^6H_{7/2}>$
21	5450.1	$0.84|^6H_{7/2}>$	21	5375.6	$0.13|^6H_{7/2}> + 0.81|^6H_{7/2}>$
22	5450.1	$0.84|^6H_{7/2}>$	22	5375.6	$0.13|^6H_{7/2}> + 0.81|^6H_{7/2}>$
23	5658.0	$0.89|^6H_{7/2}>$	23	5854.2	$0.88|^6H_{7/2}>$
24	5658.0	$0.89|^6H_{7/2}>$	24	5854.2	$0.88|^6H_{7/2}>$
25	6335.3	$0.49|^6F_{1/2}> + 0.13|^6H_{7/2}> + 0.26|^6H_{11/2}>$	25	6332.8	$0.51|^6F_{1/2}> + 0.10|^6H_{7/2}> + 0.20|^6H_{11/2}>$

26	6335.3	$0.49\vert{}^{6}F_{1/2}> + 0.13\vert{}^{6}H_{7/2}> + 0.26\vert{}^{6}H_{11/2}>$	26	6332.8	$0.51\vert{}^{6}F_{1/2}> + 0.10\vert{}^{6}H_{7/2}> + 0.20\vert{}^{6}H_{11/2}>$
27	6544.9	$0.42\vert{}^{6}F_{3/2}>$	27	6664.7	$0.22\vert{}^{6}F_{3/2}> + 0.46\vert{}^{6}H_{11/2}>$
28	6544.9	$0.42\vert{}^{6}F_{3/2}>$	28	6664.7	$0.22\vert{}^{6}F_{3/2}> + 0.46\vert{}^{6}H_{11/2}>$
29	7201.0	$0.42\vert{}^{6}H_{11/2}>$	29	6970.3	$0.29\vert{}^{6}F_{3/2}> + 0.47\vert{}^{6}H_{11/2}>$
30	7201.0	$0.42\vert{}^{6}H_{11/2}>$	30	6970.3	$0.29\vert{}^{6}F_{3/2}> + 0.47\vert{}^{6}H_{11/2}>$
31	7402.0	$0.36\vert{}^{6}H_{11/2}>$	31	7189.4	$0.32\vert{}^{6}F_{3/2}> + 0.37\vert{}^{6}H_{11/2}>$
32	7402.0	$0.36\vert{}^{6}H_{11/2}>$	32	7189.4	$0.32\vert{}^{6}F_{3/2}> + 0.37\vert{}^{6}H_{11/2}>$
33	7456.0	$0.50\vert{}^{6}H_{11/2}>$	33	7432.0	$0.15\vert{}^{6}F_{1/2}> + 0.15\vert{}^{6}F_{5/2}> + 0.42\vert{}^{6}H_{11/2}>$
34	7456.0	$0.50\vert{}^{6}H_{11/2}>$	34	7432.0	$0.15\vert{}^{6}F_{1/2}> + 0.15\vert{}^{6}F_{5/2}> + 0.42\vert{}^{6}H_{11/2}>$
35	7693.2	$0.50\vert{}^{6}H_{11/2}>$	35	7620.6	$0.27\vert{}^{6}F_{3/2}> + 0.41\vert{}^{6}H_{11/2}>$
36	7693.2	$0.50\vert{}^{6}H_{11/2}>$	36	7620.6	$0.27\vert{}^{6}F_{3/2}> + 0.41\vert{}^{6}H_{11/2}>$
37	7738.6	$0.47\vert{}^{6}H_{11/2}>$	37	7717.0	$0.13\vert{}^{6}F_{3/2}> + 0.61\vert{}^{6}H_{11/2}>$
38	7738.6	$0.47\vert{}^{6}H_{11/2}>$	38	7717.0	$0.13\vert{}^{6}F_{3/2}> + 0.61\vert{}^{6}H_{11/2}>$
39	7827.8	$0.39\vert{}^{6}H_{11/2}>$	39	7897.4	$0.22\vert{}^{6}F_{5/2}> + 0.51\vert{}^{6}H_{11/2}>$
40	7827.8	$0.39\vert{}^{6}H_{11/2}>$	40	7897.4	$0.22\vert{}^{6}F_{5/2}> + 0.51\vert{}^{6}H_{11/2}>$
41	8024.3	$0.45\vert{}^{6}H_{11/2}>$	41	8168.3	$0.11\vert{}^{6}F_{3/2}> + 0.46\vert{}^{6}F_{5/2}> + 0.16\vert{}^{6}H_{11/2}>$
42	8024.3	$0.45\vert{}^{6}H_{11/2}>$	42	8168.3	$0.11\vert{}^{6}F_{3/2}> + 0.46\vert{}^{6}F_{5/2}> + 0.16\vert{}^{6}H_{11/2}>$
43	8255.3	$0.67\vert{}^{6}H_{11/2}>$	43	8278.6	$0.15\vert{}^{6}F_{3/2}> + 0.36\vert{}^{6}F_{5/2}> + 0.23\vert{}^{6}H_{11/2}>$
44	8255.3	$0.67\vert{}^{6}H_{11/2}>$	44	8278.6	$0.15\vert{}^{6}F_{3/2}> + 0.36\vert{}^{6}F_{5/2}> + 0.23\vert{}^{6}H_{11/2}>$
45	8540.7	$0.41\vert{}^{6}F_{5/2}>$	45	8473.4	$0.46\vert{}^{6}F_{5/2}> + 0.32\vert{}^{6}H_{11/2}>$
46	8540.7	$0.41\vert{}^{6}F_{5/2}>$	46	8473.4	$0.46\vert{}^{6}F_{5/2}> + 0.32\vert{}^{6}H_{11/2}>$
47	8688.1	$0.41\vert{}^{6}F_{5/2}>$	47	8719.1	$0.78\vert{}^{6}H_{11/2}>$
48	8688.1	$0.41\vert{}^{6}F_{5/2}>$	48	8719.1	$0.78\vert{}^{6}H_{11/2}>$
49	9221.6	$0.49\vert{}^{6}F_{7/2}>$	49	9180.4	$0.16\vert{}^{6}F_{5/2}> + 0.20\vert{}^{6}F_{7/2}> + 0.37\vert{}^{6}H_{13/2}>$
50	9221.6	$0.49\vert{}^{6}F_{7/2}>$	50	9180.4	$0.16\vert{}^{6}F_{5/2}> + 0.20\vert{}^{6}F_{7/2}> + 0.37\vert{}^{6}H_{13/2}>$
51	9551.3	$0.31\vert{}^{6}F_{7/2}> + 0.11\vert{}^{6}H_{11/2}> + 0.27\vert{}^{6}H_{13/2}>$	51	9435.0	$0.11\vert{}^{6}F_{5/2}> + 0.43\vert{}^{6}F_{7/2}> + 0.18\vert{}^{6}H_{13/2}>$
52	9551.3	$0.31\vert{}^{6}F_{7/2}> + 0.11\vert{}^{6}H_{11/2}> + 0.27\vert{}^{6}H_{13/2}>$	52	9435.0	$0.11\vert{}^{6}F_{5/2}> + 0.43\vert{}^{6}F_{7/2}> + 0.18\vert{}^{6}H_{13/2}>$
53	9777.9	$0.40\vert{}^{6}F_{7/2}>$	53	9551.3	$0.28\vert{}^{6}F_{7/2}> + 0.38\vert{}^{6}H_{13/2}>$
54	9777.9	$0.40\vert{}^{6}F_{7/2}>$	54	9551.3	$0.28\vert{}^{6}F_{7/2}> + 0.38\vert{}^{6}H_{13/2}>$

55	9859.6	$0.44\|^{6}F_{7/2}> + 0.26\|^{6}H_{13/2}>$	55	9775.4	$0.14\|^{6}F_{5/2}> + 0.31\|^{6}F_{7/2}> + 0.32\|^{6}H_{13/2}>$
56	9859.6	$0.44\|^{6}F_{7/2}> + 0.26\|^{6}H_{13/2}>$	56	9775.4	$0.14\|^{6}F_{5/2}> + 0.31\|^{6}F_{7/2}> + 0.32\|^{6}H_{13/2}>$
57	10027.4	$0.37\|^{6}F_{7/2}> + 0.32\|^{6}H_{13/2}>$	57	9943.6	$0.39\|^{6}F_{7/2}> + 0.34\|^{6}H_{13/2}>$
58	10027.4	$0.37\|^{6}F_{7/2}> + 0.32\|^{6}H_{13/2}>$	58	9943.6	$0.39\|^{6}F_{7/2}> + 0.34\|^{6}H_{13/2}>$
59	10181.6	$0.32\|^{6}F_{7/2}> + 0.40\|^{6}H_{13/2}>$	59	10108.0	$0.29\|^{6}F_{7/2}> + 0.44\|^{6}H_{13/2}>$
60	10181.6	$0.32\|^{6}F_{7/2}> + 0.40\|^{6}H_{13/2}>$	60	10108.0	$0.29\|^{6}F_{7/2}> + 0.44\|^{6}H_{13/2}>$
61	10536.7	$0.18\|^{6}F_{7/2}> + 0.57\|^{6}H_{13/2}>$	61	10255.1	$0.50\|^{6}F_{7/2}> + 0.24\|^{6}H_{13/2}>$
62	10536.7	$0.18\|^{6}F_{7/2}> + 0.57\|^{6}H_{13/2}>$	62	10255.1	$0.50\|^{6}F_{7/2}> + 0.24\|^{6}H_{13/2}>$
63	10674.4	$0.65\|^{6}H_{13/2}>$	63	10503.0	$0.26\|^{6}F_{7/2}> + 0.44\|^{6}H_{13/2}>$
64	10674.4	$0.65\|^{6}H_{13/2}>$	64	10503.0	$0.26\|^{6}F_{7/2}> + 0.44\|^{6}H_{13/2}>$
65	10819.1	$0.73\|^{6}H_{13/2}>$	65	10724.0	$0.16\|^{6}F_{7/2}> + 0.56\|^{6}H_{13/2}>$
66	10819.1	$0.73\|^{6}H_{13/2}>$	66	10724.0	$0.16\|^{6}F_{7/2}> + 0.56\|^{6}H_{13/2}>$
67	11004.3	$0.12\|^{6}F_{7/2}> + 0.11\|^{6}F_{9/2}> + 0.57\|^{6}H_{13/2}>$	67	11077.5	$0.12\|^{6}F_{9/2}> + 0.42\|^{6}H_{13/2}> + 0.22\|^{6}H_{15/2}>$
68	11004.3	$0.12\|^{6}F_{7/2}> + 0.11\|^{6}F_{9/2}> + 0.57\|^{6}H_{13/2}>$	68	11077.5	$0.12\|^{6}F_{9/2}> + 0.42\|^{6}H_{13/2}> + 0.22\|^{6}H_{15/2}>$
69	11257.5	$0.16\|^{6}F_{9/2}> + 0.59\|^{6}H_{13/2}>$	69	11307.3	$0.11\|^{6}F_{9/2}> + 0.43\|^{6}H_{13/2}> + 0.22\|^{6}H_{15/2}>$
70	11257.5	$0.16\|^{6}F_{9/2}> + 0.59\|^{6}H_{13/2}>$	70	11307.3	$0.11\|^{6}F_{9/2}> + 0.43\|^{6}H_{13/2}> + 0.22\|^{6}H_{15/2}>$
71	11468.7	$0.11\|^{6}F_{7/2}> + 0.22\|^{6}F_{9/2}> + 0.35\|^{6}H_{13/2}> + 0.18\|^{6}H_{15/2}>$	71	11599.0	$0.28\|^{6}F_{9/2}> + 0.23\|^{6}H_{13/2}> + 0.26\|^{6}H_{15/2}>$
72	11468.7	$0.11\|^{6}F_{7/2}> + 0.22\|^{6}F_{9/2}> + 0.35\|^{6}H_{13/2}> + 0.18\|^{6}H_{15/2}>$	72	11599.0	$0.28\|^{6}F_{9/2}> + 0.23\|^{6}H_{13/2}> + 0.26\|^{6}H_{15/2}>$
73	11746.4	$0.11\|^{6}F_{7/2}> + 0.43\|^{6}F_{9/2}> + 0.14\|^{6}H_{13/2}> + 0.22\|^{6}H_{15/2}>$	73	11784.3	$0.21\|^{6}F_{9/2}> + 0.55\|^{6}H_{13/2}>$
74	11746.4	$0.11\|^{6}F_{7/2}> + 0.43\|^{6}F_{9/2}> + 0.14\|^{6}H_{13/2}> + 0.22\|^{6}H_{15/2}>$	74	11784.3	$0.21\|^{6}F_{9/2}> + 0.55\|^{6}H_{13/2}>$
75	12148.0	$0.67\|^{6}F_{9/2}> + 0.12\|^{6}H_{13/2}>$	75	12017.3	$0.44\|^{6}F_{9/2}> + 0.26\|^{6}H_{13/2}> + 0.15\|^{6}H_{15/2}>$
76	12148.0	$0.67\|^{6}F_{9/2}> + 0.12\|^{6}H_{13/2}>$	76	12017.3	$0.44\|^{6}F_{9/2}> + 0.26\|^{6}H_{13/2}> + 0.15\|^{6}H_{15/2}>$
77	12267.9	$0.71\|^{6}F_{9/2}> + 0.13\|^{6}H_{13/2}>$	77	12159.5	$0.54\|^{6}F_{9/2}> + 0.21\|^{6}H_{13/2}> + 0.11\|^{6}H_{15/2}>$
78	12267.9	$0.71\|^{6}F_{9/2}> + 0.13\|^{6}H_{13/2}>$	78	12159.5	$0.54\|^{6}F_{9/2}> + 0.21\|^{6}H_{13/2}> + 0.11\|^{6}H_{15/2}>$
79	12364.0	$0.64\|^{6}F_{9/2}> + 0.13\|^{6}H_{13/2}> + 0.12\|^{6}H_{15/2}>$	79	12323.7	$0.59\|^{6}F_{9/2}> + 0.10\|^{6}H_{13/2}> + 0.19\|^{6}H_{15/2}>$
80	12364.0	$0.64\|^{6}F_{9/2}> + 0.13\|^{6}H_{13/2}> + 0.12\|^{6}H_{15/2}>$	80	12323.7	$0.59\|^{6}F_{9/2}> + 0.10\|^{6}H_{13/2}> + 0.19\|^{6}H_{15/2}>$
81	12784.0	$0.14\|^{6}F_{9/2}> + 0.12\|^{6}H_{13/2}> + 0.55\|^{6}H_{15/2}>$	81	12484.5	$0.59\|^{6}F_{9/2}> + 0.11\|^{6}H_{13/2}> + 0.19\|^{6}H_{15/2}>$

82	12784.0	$0.14\|^{6}F_{9/2}> + 0.12\|^{6}H_{13/2}> + 0.55\|^{6}H_{15/2}>$	82	12484.5	$0.59\|^{6}F_{9/2}> + 0.11\|^{6}H_{13/2}> + 0.19\|^{6}H_{15/2}>$
83	13136.1	$0.24\|^{6}F_{9/2}> + 0.11\|^{6}H_{13/2}> + 0.47\|^{6}H_{15/2}>$	83	12675.9	$0.57\|^{6}F_{9/2}> + 0.13\|^{6}H_{13/2}> + 0.19\|^{6}H_{15/2}>$
84	13136.1	$0.24\|^{6}F_{9/2}> + 0.11\|^{6}H_{13/2}> + 0.47\|^{6}H_{15/2}>$	84	12675.9	$0.57\|^{6}F_{9/2}> + 0.13\|^{6}H_{13/2}> + 0.19\|^{6}H_{15/2}>$
85	13276.6	$0.23\|^{6}F_{9/2}> + 0.51\|^{6}H_{15/2}>$	85	13012.6	$0.35\|^{6}F_{9/2}> + 0.14\|^{6}H_{13/2}> + 0.38\|^{6}H_{15/2}>$
86	13276.6	$0.23\|^{6}F_{9/2}> + 0.51\|^{6}H_{15/2}>$	86	13012.6	$0.35\|^{6}F_{9/2}> + 0.14\|^{6}H_{13/2}> + 0.38\|^{6}H_{15/2}>$
87	13598.1	$0.20\|^{6}F_{9/2}> + 0.11\|^{6}H_{13/2}> + 0.57\|^{6}H_{15/2}>$	87	13235.1	$0.34\|^{6}F_{9/2}> + 0.14\|^{6}H_{13/2}> + 0.40\|^{6}H_{15/2}>$
88	13598.1	$0.20\|^{6}F_{9/2}> + 0.11\|^{6}H_{13/2}> + 0.57\|^{6}H_{15/2}>$	88	13235.1	$0.34\|^{6}F_{9/2}> + 0.14\|^{6}H_{13/2}> + 0.40\|^{6}H_{15/2}>$
89	13711.8	$0.21\|^{6}F_{9/2}> + 0.11\|^{6}H_{13/2}> + 0.56\|^{6}H_{15/2}>$	89	13831.7	$0.18\|^{6}H_{13/2}> + 0.59\|^{6}H_{15/2}>$
90	13711.8	$0.21\|^{6}F_{9/2}> + 0.11\|^{6}H_{13/2}> + 0.56\|^{6}H_{15/2}>$	90	13831.7	$0.18\|^{6}H_{13/2}> + 0.59\|^{6}H_{15/2}>$
91	14099.7	$0.12\|^{6}F_{11/2}> + 0.16\|^{6}H_{13/2}> + 0.59\|^{6}H_{15/2}>$	91	14606.1	$0.29\|^{6}F_{11/2}> + 0.59\|^{6}H_{15/2}>$
92	14099.7	$0.12\|^{6}F_{11/2}> + 0.16\|^{6}H_{13/2}> + 0.59\|^{6}H_{15/2}>$	92	14606.1	$0.29\|^{6}F_{11/2}> + 0.59\|^{6}H_{15/2}>$
93	14486.3	$0.16\|^{6}F_{11/2}> + 0.66\|^{6}H_{15/2}>$	93	14841.4	$0.51\|^{6}F_{11/2}> + 0.42\|^{6}H_{15/2}>$
94	14486.3	$0.16\|^{6}F_{11/2}> + 0.66\|^{6}H_{15/2}>$	94	14841.4	$0.51\|^{6}F_{11/2}> + 0.42\|^{6}H_{15/2}>$
95	14881.1	$0.73\|^{6}F_{11/2}> + 0.23\|^{6}H_{15/2}>$	95	15165.9	$0.64\|^{6}F_{11/2}> + 0.32\|^{6}H_{15/2}>$
96	14881.1	$0.73\|^{6}F_{11/2}> + 0.23\|^{6}H_{15/2}>$	96	15165.9	$0.64\|^{6}F_{11/2}> + 0.32\|^{6}H_{15/2}>$
97	15231.4	$0.87\|^{6}F_{11/2}>$	97	15451.4	$0.73\|^{6}F_{11/2}> + 0.24\|^{6}H_{15/2}>$
98	15231.4	$0.87\|^{6}F_{11/2}>$	98	15451.4	$0.73\|^{6}F_{11/2}> + 0.24\|^{6}H_{15/2}>$
99	15339.2	$0.86\|^{6}F_{11/2}>$	99	15466.1	$0.71\|^{6}F_{11/2}> + 0.26\|^{6}H_{15/2}>$
100	15339.2	$0.86\|^{6}F_{11/2}>$	100	15466.1	$0.71\|^{6}F_{11/2}> + 0.26\|^{6}H_{15/2}>$
101	15580.2	$0.84\|^{6}F_{11/2}> + 0.11\|^{6}H_{15/2}>$	101	15554.9	$0.82\|^{6}F_{11/2}> + 0.15\|^{6}H_{15/2}>$
102	15580.2	$0.84\|^{6}F_{11/2}> + 0.11\|^{6}H_{15/2}>$	102	15554.9	$0.82\|^{6}F_{11/2}> + 0.15\|^{6}H_{15/2}>$
103	15687.6	$0.74\|^{6}F_{11/2}> + 0.22\|^{6}H_{15/2}>$	103	15647.5	$0.78\|^{6}F_{11/2}> + 0.19\|^{6}H_{15/2}>$
104	15687.6	$0.74\|^{6}F_{11/2}> + 0.22\|^{6}H_{15/2}>$	104	15647.5	$0.78\|^{6}F_{11/2}> + 0.19\|^{6}H_{15/2}>$
105	15873.6	$0.49\|^{6}F_{11/2}> + 0.44\|^{6}H_{15/2}>$	105	15759.4	$0.73\|^{6}F_{11/2}> + 0.24\|^{6}H_{15/2}>$
106	15873.6	$0.49\|^{6}F_{11/2}> + 0.44\|^{6}H_{15/2}>$	106	15759.4	$0.73\|^{6}F_{11/2}> + 0.24\|^{6}H_{15/2}>$
107	16002.8	$0.66\|^{6}F_{11/2}> + 0.29\|^{6}H_{15/2}>$	107	15844.0	$0.35\|^{6}F_{11/2}> + 0.61\|^{6}H_{15/2}>$
108	16002.8	$0.66\|^{6}F_{11/2}> + 0.29\|^{6}H_{15/2}>$	108	15844.0	$0.35\|^{6}F_{11/2}> + 0.61\|^{6}H_{15/2}>$
109	17280.7	$0.20\|^{4}D_{5/2}> + 0.24\|^{4}F_{5/2}>$	109	17203.3	$0.31\|^{4}D_{3/2}> + 0.23\|^{4}D_{5/2}> + 0.11\|^{4}F_{3/2}> + 0.12\|^{4}F_{5/2}>$

110	17280.7	$0.20\vert^4D_{5/2}> + 0.24\vert^4F_{5/2}>$	110	17203.3	$0.31\vert^4D_{3/2}> + 0.23\vert^4D_{5/2}> + 0.11\vert^4F_{3/2}> + 0.12\vert^4F_{5/2}>$
111	18051.8	$0.15\vert^4D_{5/2}> + 0.12\vert^4K_{11/2}>$	111	18472.7	$0.20\vert^4H_{7/2}> + 0.12\vert^4H_{9/2}>$
112	18051.8	$0.15\vert^4D_{5/2}> + 0.12\vert^4K_{11/2}>$	112	18472.7	$0.20\vert^4H_{7/2}> + 0.12\vert^4H_{9/2}>$
113	18517.7	$0.12\vert^4G_{7/2}> + 0.14\vert^4H_{7/2}> + 0.17\vert^4I_{9/2}>$	113	18742.5	$0.23\vert^4H_{7/2}> + 0.18\vert^4H_{9/2}> + 0.12\vert^4I_{9/2}>$
114	18517.7	$0.12\vert^4G_{7/2}> + 0.14\vert^4H_{7/2}> + 0.17\vert^4I_{9/2}>$	114	18742.5	$0.23\vert^4H_{7/2}> + 0.18\vert^4H_{9/2}> + 0.12\vert^4I_{9/2}>$
115	19427.1	$0.16\vert^4G_{7/2}> + 0.16\vert^4H_{7/2}> + 0.14\vert^4I_{9/2}>$	115	19172.1	$0.10\vert^4G_{7/2}> + 0.24\vert^4H_{7/2}> + 0.11\vert^4H_{9/2}>$
116	19427.1	$0.16\vert^4G_{7/2}> + 0.16\vert^4H_{7/2}> + 0.14\vert^4I_{9/2}>$	116	19172.1	$0.10\vert^4G_{7/2}> + 0.24\vert^4H_{7/2}> + 0.11\vert^4H_{9/2}>$
117	19759.6	$0.12\vert^4G_{7/2}> + 0.14\vert^4H_{7/2}>$	117	19495.1	$0.26\vert^4H_{7/2}> + 0.17\vert^4H_{9/2}>$
118	19759.6	$0.12\vert^4G_{7/2}> + 0.14\vert^4H_{7/2}>$	118	19495.1	$0.26\vert^4H_{7/2}> + 0.17\vert^4H_{9/2}>$
119	20246.9	$0.10\vert^4I_{11/2}>$	119	20087.1	$0.27\vert^4L_{13/2}> + 0.17\vert^4L_{15/2}>$
120	20246.9	$0.10\vert^4I_{11/2}>$	120	20087.1	$0.27\vert^4L_{13/2}> + 0.17\vert^4L_{15/2}>$
121	20547.5	$0.13\vert^4K_{13/2}> + 0.25\vert^4L_{13/2}>$	121	20252.1	$0.17\vert^4L_{13/2}>$
122	20547.5	$0.13\vert^4K_{13/2}> + 0.25\vert^4L_{13/2}>$	122	20252.1	$0.17\vert^4L_{13/2}>$
123	20959.9	$0.14\vert^4I_{11/2}> + 0.12\vert^4K_{11/2}> + 0.12\vert^4K_{13/2}>$	123	20807.7	$0.15\vert^4L_{13/2}> + 0.20\vert^4L_{15/2}>$
124	20959.9	$0.14\vert^4I_{11/2}> + 0.12\vert^4K_{11/2}> + 0.12\vert^4K_{13/2}>$	124	20807.7	$0.15\vert^4L_{13/2}> + 0.20\vert^4L_{15/2}>$
125	21245.8	$0.12\vert^4I_{11/2}> + 0.15\vert^4K_{11/2}> + 0.11\vert^4K_{13/2}>$	125	21181.4	$0.12\vert^4H_{9/2}> + 0.14\vert^4L_{13/2}> + 0.18\vert^4L_{15/2}>$
126	21245.8	$0.12\vert^4I_{11/2}> + 0.15\vert^4K_{11/2}> + 0.11\vert^4K_{13/2}>$	126	21181.4	$0.12\vert^4H_{9/2}> + 0.14\vert^4L_{13/2}> + 0.18\vert^4L_{15/2}>$
127	21733.9	$0.11\vert^4K_{13/2}> + 0.10\vert^4K_{15/2}>$	127	21664.6	$0.21\vert^4H_{9/2}> + 0.17\vert^4L_{15/2}>$
128	21733.9	$0.11\vert^4K_{13/2}> + 0.10\vert^4K_{15/2}>$	128	21664.6	$0.21\vert^4H_{9/2}> + 0.17\vert^4L_{15/2}>$
129	22153.7	$0.13\vert^4G_{7/2}>$	129	21832.4	$0.21\vert^4H_{9/2}> + 0.13\vert^4H_{11/2}>$
130	22153.7	$0.13\vert^4G_{7/2}>$	130	21832.4	$0.21\vert^4H_{9/2}> + 0.13\vert^4H_{11/2}>$
131	22506.3	$0.12\vert^4H_{9/2}> + 0.14\vert^4K_{13/2}> + 0.16\vert^4L_{15/2}>$	131	22416.6	$0.12\vert^4H_{9/2}> + 0.15\vert^4H_{11/2}> + 0.10\vert^4L_{15/2}>$
132	22506.3	$0.12\vert^4H_{9/2}> + 0.14\vert^4K_{13/2}> + 0.16\vert^4L_{15/2}>$	132	22416.6	$0.12\vert^4H_{9/2}> + 0.15\vert^4H_{11/2}> + 0.10\vert^4L_{15/2}>$
133	22841.4	$0.10\vert^4D_{5/2}>$	133	22753.6	$0.17\vert^4L_{15/2}> + 0.10\vert^4L_{17/2}>$
134	22841.4	$0.10\vert^4D_{5/2}>$	134	22753.6	$0.17\vert^4L_{15/2}> + 0.10\vert^4L_{17/2}>$
135	23182.5	$0.15\vert^4G_{9/2}> + 0.11\vert^4K_{15/2}> + 0.15\vert^4L_{15/2}>$	135	23059.6	$0.11\vert^4D_{5/2}>$
136	23182.5	$0.15\vert^4G_{9/2}> + 0.11\vert^4K_{15/2}> + 0.15\vert^4L_{15/2}>$	136	23059.6	$0.11\vert^4D_{5/2}>$
137	23486.4	$0.10\vert^4K_{13/2}>$	137	23353.5	$0.14\vert^4L_{15/2}> + 0.30\vert^4L_{17/2}>$
138	23486.4	$0.10\vert^4K_{13/2}>$	138	23353.5	$0.14\vert^4L_{15/2}> + 0.30\vert^4L_{17/2}>$

139	23915.5	$0.10|^4I_{11/2}> + 0.12|^4I_{13/2}> + 0.10|^4K_{13/2}>$	139	23800.8	$0.10|^4H_{9/2}> + 0.15|^4H_{11/2}> + 0.13|^4L_{17/2}>$
140	23915.5	$0.10|^4I_{11/2}> + 0.12|^4I_{13/2}> + 0.10|^4K_{13/2}>$	140	23800.8	$0.10|^4H_{9/2}> + 0.15|^4H_{11/2}> + 0.13|^4L_{17/2}>$
141	24239.4	$0.15|^4I_{13/2}> + 0.13|^4K_{15/2}>$	141	24115.0	$0.17|^4H_{11/2}> + 0.14|^4L_{17/2}>$
142	24239.4	$0.15|^4I_{13/2}> + 0.13|^4K_{15/2}>$	142	24115.0	$0.17|^4H_{11/2}> + 0.14|^4L_{17/2}>$
143	24492.8	$0.10|^4I_{13/2}> + 0.15|^4K_{15/2}> + 0.12|^4L_{19/2}> + 0.11|^4L_{17/2}>$	143	24430.1	$0.10|^4G_{11/2}> + 0.23|^4H_{11/2}>$
144	24492.8	$0.10|^4I_{13/2}> + 0.15|^4K_{15/2}> + 0.12|^4L_{19/2}> + 0.11|^4L_{17/2}>$	144	24430.1	$0.10|^4G_{11/2}> + 0.23|^4H_{11/2}>$
145	24777.7	$0.11|^4K_{15/2}> + 0.11|^4L_{19/2}> + 0.13|^4L_{17/2}>$	145	24922.0	$0.23|^4H_{11/2}> + 0.14|^4L_{17/2}>$
146	24777.7	$0.11|^4K_{15/2}> + 0.11|^4L_{19/2}> + 0.13|^4L_{17/2}>$	146	24922.0	$0.23|^4H_{11/2}> + 0.14|^4L_{17/2}>$
147	25249.5	$0.10|^4K_{15/2}> + 0.19|^4L_{17/2}>$	147	25186.4	$0.13|^4H_{11/2}> + 0.21|^4L_{17/2}> + 0.14|^4L_{19/2}>$
148	25249.5	$0.10|^4K_{15/2}> + 0.19|^4L_{17/2}>$	148	25186.4	$0.13|^4H_{11/2}> + 0.21|^4L_{17/2}> + 0.14|^4L_{19/2}>$
149	25551.2	$0.12|^4H_{11/2}> + 0.11|^4K_{15/2}> + 0.11|^4L_{19/2}> + 0.17|^4L_{17/2}>$	149	25606.9	$0.15|^4H_{11/2}> + 0.12|^4L_{17/2}>$
150	25551.2	$0.12|^4H_{11/2}> + 0.11|^4K_{15/2}> + 0.11|^4L_{19/2}> + 0.17|^4L_{17/2}>$	150	25606.9	$0.15|^4H_{11/2}> + 0.12|^4L_{17/2}>$
151	25764.7	$0.12|^4G_{11/2}> + 0.12|^4H_{11/2}> + 0.11|^4L_{17/2}>$	151	25838.5	$0.12|^4H_{11/2}> + 0.12|^4L_{17/2}>$
152	25764.7	$0.12|^4G_{11/2}> + 0.12|^4H_{11/2}> + 0.11|^4L_{17/2}>$	152	25838.5	$0.12|^4H_{11/2}> + 0.12|^4L_{17/2}>$
153	26437.2	$0.11|^4F_{7/2}> + 0.12|^4H_{11/2}>$	153	26371.8	$0.14|^4H_{11/2}> + 0.17|^4L_{19/2}>$
154	26437.2	$0.11|^4F_{7/2}> + 0.12|^4H_{11/2}>$	154	26371.8	$0.14|^4H_{11/2}> + 0.17|^4L_{19/2}>$
155	26915.1	$0.14|^4H_{11/2}>$	155	26732.2	$0.18|^4H_{11/2}>$
156	26915.1	$0.14|^4H_{11/2}>$	156	26732.2	$0.18|^4H_{11/2}>$
157	27267.9	$0.13|^4H_{13/2}> + 0.21|^4L_{19/2}>$	157	27466.5	$0.12|^4H_{13/2}> + 0.31|^4L_{19/2}>$
158	27267.9	$0.13|^4H_{13/2}> + 0.21|^4L_{19/2}>$	158	27466.5	$0.12|^4H_{13/2}> + 0.31|^4L_{19/2}>$
159	27614.4	$0.24|^4L_{19/2}> + 0.15|^4L_{17/2}> + 0.13|^4L_{19/2}>$	159	27657.1	$0.43|^4L_{19/2}>$
160	27614.4	$0.24|^4L_{19/2}> + 0.15|^4L_{17/2}> + 0.13|^4L_{19/2}>$	160	27657.1	$0.43|^4L_{19/2}>$
161	27814.8	$0.28|^4L_{19/2}> + 0.13|^4L_{17/2}> + 0.31|^4L_{19/2}>$	161	27895.9	$0.44|^4L_{19/2}>$
162	27814.8	$0.28|^4L_{19/2}> + 0.13|^4L_{17/2}> + 0.31|^4L_{19/2}>$	162	27895.9	$0.44|^4L_{19/2}>$
163	28023.5	$0.13|^4I_{15/2}> + 0.17|^4L_{19/2}> + 0.19|^4L_{19/2}>$	163	28049.6	$0.36|^4L_{19/2}> + 0.10|^4M_{19/2}>$
164	28023.5	$0.13|^4I_{15/2}> + 0.17|^4L_{19/2}> + 0.19|^4L_{19/2}>$	164	28049.6	$0.36|^4L_{19/2}> + 0.10|^4M_{19/2}>$
165	28206.2	$0.11|^4I_{15/2}> + 0.18|^4L_{19/2}> + 0.13|^4L_{17/2}> + 0.14|^4L_{19/2}>$	165	28623.5	$0.37|^4H_{13/2}> + 0.11|^6H_{15/2}>$

166	28206.2	$0.11\|^4\mathrm{I}_{15/2}> + 0.18\|^4\mathrm{L}_{19/2}> + 0.13\|^4\mathrm{L}_{17/2}> + 0.14\|^4\mathrm{L}_{19/2}>$	166	28623.5	$0.37\|^4\mathrm{H}_{13/2}> + 0.11\|^6\mathrm{H}_{15/2}>$
167	28548.5	$0.13\|^4\mathrm{H}_{13/2}> + 0.15\|^4\mathrm{I}_{13/2}> + 0.11\|^4\mathrm{I}_{15/2}>$	167	28928.4	$0.35\|^4\mathrm{H}_{13/2}> + 0.11\|^6\mathrm{H}_{15/2}>$
168	28548.5	$0.13\|^4\mathrm{H}_{13/2}> + 0.15\|^4\mathrm{I}_{13/2}> + 0.11\|^4\mathrm{I}_{15/2}>$	168	28928.4	$0.35\|^4\mathrm{H}_{13/2}> + 0.11\|^6\mathrm{H}_{15/2}>$
169	28944.7	$0.25\|^4\mathrm{I}_{15/2}>$	169	29252.8	$0.17\|^4\mathrm{H}_{13/2}> + 0.16\|^4\mathrm{I}_{13/2}> + 0.22\|^4\mathrm{I}_{15/2}> + 0.11\|^6\mathrm{H}_{15/2}>$
170	28944.7	$0.25\|^4\mathrm{I}_{15/2}>$	170	29252.8	$0.17\|^4\mathrm{H}_{13/2}> + 0.16\|^4\mathrm{I}_{13/2}> + 0.22\|^4\mathrm{I}_{15/2}> + 0.11\|^6\mathrm{H}_{15/2}>$
171	29440.9	$0.12\|^4\mathrm{G}_{11/2}> + 0.12\|^4\mathrm{H}_{11/2}> + 0.13\|^4\mathrm{H}_{13/2}>$	171	29695.3	$0.20\|^4\mathrm{D}_{7/2}> + 0.13\|^4\mathrm{F}_{7/2}> + 0.25\|^4\mathrm{H}_{13/2}>$
172	29440.9	$0.12\|^4\mathrm{G}_{11/2}> + 0.12\|^4\mathrm{H}_{11/2}> + 0.13\|^4\mathrm{H}_{13/2}>$	172	29695.3	$0.20\|^4\mathrm{D}_{7/2}> + 0.13\|^4\mathrm{F}_{7/2}> + 0.25\|^4\mathrm{H}_{13/2}>$

Table S25. State energies and composition of Sm complexes computed with CASSCF-SO. Only m_J contributions ≥ 20% are included.

**2Sm**					
**State**	**Energy (cm^{-1})**	**m_J Composition**	g_1	g_2	g_3
KD1	0	0.78|±5/2>	0.07	0.58	0.90
KD2	200.6	0.50|±3/2>	0.09	0.30	0.77
KD3	406.9	0.71|±1/2>	0.43	0.72	1.29
**3Sm**					
**State**	**Energy (cm^{-1})**	**m_J Composition**	g_1	g_2	g_3
KD1	0	0.43|±1/2> + 0.25|∓3/2>	0.01	0.26	0.71
KD2	282.4	0.63|±5/2> + 0.27|∓3/2>	0.17	0.47	1.16
KD3	537.8	0.32|±1/2> + 0.23|∓3/2>	0.06	0.17	2.42

Table S26. State energies and composition of Sm complexes computed with MS-CASPT2-SO. Only m_J contributions ≥ 20% are included.

**2Sm**					
**State**	**Energy (cm^{-1})**	***m_J* Composition**	g_1	g_2	g_3
KD1	0	0.57|±5/2> + 0.26|±3/2>	0.06	0.73	0.77
KD2	139.9	0.37|±5/2> + 0.29|±3/2>	0.17	0.47	1.04
KD3	305.8	0.59|±1/2>	0.57	0.69	1.38
**3Sm**					
**State**	**Energy (cm^{-1})**	***m_J* Composition**	g_1	g_2	g_3
KD1	0	0.41|∓1/2> + 0.25|±3/2>	0.01	0.27	0.60
KD2	318.5	0.64|∓5/2> + 0.24|±3/2>	0.26	0.47	1.20
KD3	563.3	0.31|∓1/2> + 0.22|±3/2>	0.11	0.21	2.29

Table S27. State energies and composition of Sm complexes computed with XMS-CASPT2-SO. Only m_J contributions ≥ 20% are included.

**2Sm**					
**State**	**Energy (cm^{-1})**	***m_J* Composition**	g_1	g_2	g_3
KD1	0	0.49|∓5/2> + 0.27|∓3/2>	0.20	0.34	0.60
KD2	242.3	0.34|±5/2>	0.20	0.56	1.42
KD3	433.7	0.43|∓1/2> + 0.26|∓3/2>	0.37	0.94	1.71
**3Sm**					
**State**	**Energy (cm^{-1})**	***m_J* Composition**	g_1	g_2	g_3
KD1	0	0.32|∓1/2> + 0.21|∓5/2>	0.04	0.23	0.78
KD2	230.9	0.45|∓5/2> + 0.32|±3/2>	0.15	0.24	0.84
KD3	560.3	0.30|±5/2> + 0.26|±1/2>	0.54	0.76	1.97

Table S28. State energies and composition of Pu complexes computed with CASSCF-SO. Only m_J contributions ≥ 20% are included.

**2Pu**					
**State**	**Energy (cm^{-1})**	***m_J* Composition**	***g_1***	***g_2***	***g_3***
KD1	0	0.87|∓5/2>	0.19	0.47	0.67
KD2	379.8	0.68|∓3/2>	0.11	0.26	0.71
KD3	763.0	0.77|∓1/2>	0.27	0.33	1.55
**3Pu**					
**State**	**Energy (cm^{-1})**	***m_J* Composition**	***g_1***	***g_2***	***g_3***
KD1	0	0.26|±5/2> + 0.26|±1/2> + 0.20|±3/2>	0.09	0.36	0.55
KD2	426.4	0.58|±5/2>	0.26	0.32	1.33
KD3	968.4	0.31|±1/2> + 0.24|±3/2>	0.02	0.09	2.46

Table S29. State energies and composition of Pu complexes computed with MS-CASPT2-SO. Only m_J contributions ≥ 20% are included.

**2Pu**					
**State**	**Energy (cm^{-1})**	***m_J* Composition**	***g_1***	***g_2***	***g_3***
KD1	0	80|∓5/2>	0.01	0.64	0.71
KD2	233.3	0.65|∓3/2>	0.08	0.2	1.03
KD3	575.4	0.62|∓1/2>	0.19	0.52	1.53
**3Pu**					
**State**	**Energy (cm^{-1})**	***m_J* Composition**	***g_1***	***g_2***	***g_3***
KD1	0	0.26|∓1/2> + 0.24|∓3/2> + 0.22|∓5/2>	0.05	0.35	0.46
KD2	458.6	0.62|±5/2>	0.18	0.25	1.27
KD3	1002.0	0.30|∓3/2> + 0.25|∓1/2>	0.02	0.06	2.24

Table S30. State energies and composition of Pu complexes computed with XMS-CASPT2-SO. Only m_J contributions ≥ 20% are included.

2Pu					
**State**	**Energy (cm^{-1})**	**m_J Composition**	g_1	g_2	g_3
KD1	0	71|∓5/2>	0.15	0.36	0.7
KD2	419.6	0.37|∓3/2>	0.2	0.46	1.22
KD3	769.9	0.32|±1/2> + 0.27|∓1/2>	0.24	0.83	1.48
**3Pu**					
**State**	**Energy (cm^{-1})**	**m_J Composition**	g_1	g_2	g_3
KD1	0		0.21	0.33	0.37
KD2	445.1		0.07	0.33	1.58
KD3	1166.3		0.01	0.42	2.79

References

1. Bruker *SMART APEX II.* **2012**. Bruker AXS Inc., Madison, Wisconsin, USA.
2. Bruker *SAINT.* **2012**. Bruker AXS Inc., Madison, Wisconsin, USA.
3. Bruker *SADABS.* **2016**. Bruker AXS Inc., Madison, Wisconsin, USA.
4. Sheldrick, G. M. SHELXTL version 5. **2001**, Bruker AXS Inc, Madison, Wisconsin, USA.
5. Dolomanov, O. V., Bourhis, L. J., Gildea, R. J., Howard, J. A. K., Puschmann, H. OLEX2: a complete structure solution, refinement and analysis program. *J. Appl. Cryst*. **2009**, *42*, 339-341.
6. Farrugia, L. J. WinGX and ORTEP for Windows: an update. *J. Appl. Cryst.* **2012**, *45*, 849-854.
7. Persistence of Vision (TM) Raytracer, Persistence of Vision Pty. Ltd., Williamstown, Victoria, Australia.
8. CrysAlisPRO version 40.69, Oxford Diffraction/Agilent Technologies UK Ltd, Yarnton, England.
9. Hitchcock, P. B.; Lappert, M. F.; Maron, L.; Protchenko, A. V. Lanthanum does form stable molecular compounds in the +2 oxidation state. *Angew. Chem. Int. Ed.* **2008**, *47*, 1488-1491.

10. Sheldrick, G. M. SHELXT – Integrated space-group and crystal-structure determination. *Acta Cryst. Sect. A* **2015**, *71*, 3-8.

11. Sheldrick, G. M. Crystal structure refinement with SHELXL. *Acta Cryst. Sect. C* **2015**, *71*, 3-8.

12. Izod, K.; Liddle, S. T.; Clegg, W. A Convenient Route to Lanthanide Triiodide THF Solvates. Crystal Structures of $LnI_3(THF)_4$ [Ln = Pr] and $LnI_3(THF)_{3.5}$ [Ln = Nd, Gd, Y]. *Inorg. Chem.* **2004**, *43*, 214-218.

13. Goodwin, C. A. P.; Gaunt, A. J.; Janicke, M. T.; Scott, B. L. [$AnI_3(THF)_4$] (An = Np, Pu) preparation bypassing An^0 metal precursors: access to Np^{3+}/Pu^{3+} nonaqueous and organometallic complexes. *J. Am. Chem. Soc.* **2021**, *143*, 20680-20696.

14. Wooles, A. J.; Gregson, M.; Cooper, O. J.; Middleton-Gear, A.; Mills, D. P.; Lewis, W.; Blake, A. J.; Liddle, S. T. Group 1 Bis(iminophosphorano)methanides, Part 1: *N*-Alkyl and Silyl Derivatives of the Sterically Demanding Methanes $H_2C(PPh_2NR)_2$ (R = Adamantyl and Trimethylsilyl). *Organometallics* **2011**, *30*, 5314-5325.

15. Ansell, M. B.; Roberts, D. E.; Cloke, F. G.; Navarro, O.; Spencer, J. Synthesis of an [$(NHC)_2Pd(SiMe_3)_2$] Complex and Catalytic *cis*-Bis(silyl)ations of Alkynes with Unactivated Disilanes. *Angew. Chem. Int. Ed.* **2015**, *54*, 5578-5582.

16. Bailey, P. J.; Coxall, R. A.; Dick, C. M.; Fabre, S.; Henderson, L. C.; Herber, C.; Liddle, S. T.; Loroño-González, D.; Parkin A.; Parsons, S. The first structural characterisation of a group 2 metal alkylperoxide complex: comments on the cleavage of dioxygen by magnesium alkyl complexes. *Chem. Eur. J.* **2003**, *9*, 4820-4828.

17. Fonseca Guerra, C.; Snijders, J. G.; Te Velde, G.; Baerends, E. J. Towards an order-N DFT Method. *Theor. Chem. Acc.* **1998**, *99*, 391-403.

18. Te Velde, G.; Bickelhaupt, F. M.; Baerends, E. J.; Fonseca Guerra, C.; Van Gisbergen, S. J. A.; Snijders, J. G.; Ziegler, T. Chemistry with ADF. *J. Comput. Chem.* **2001**, *22*, 931-967.

19. Van Lenthe, E.; Baerends, E. J.; Snijders, J. G. Relativistic regular two-component Hamiltonians. *J. Chem. Phys.* **1993**, *99*, 4597-4610.

20. Van Lenthe, E.; Baerends, E. J.; Snijders, J. G. Relativistic total energy using regular approximations. *J. Chem. Phys.* **1994**, *101*, 9783-9792.

21. Van Lenthe, E.; Ehlers, A. E.; Baerends, E. J. Geometry optimization in the Zero Order Regular Approximation for relativistic effects. *J. Chem. Phys.* **1999**, *110*, 8943-8953.

22. Vosko, S. H.; Wilk, L.; Nusair, M. Accurate spin-dependent electron liquid correlation energies for local spin density calculations: a critical analysis. *Can. J. Phys.* **1980**, *58*, 1200-1211.

23. Becke, A. D. Density-functional exchange-energy approximation with correct asymptotic behaviour. *Phys. Rev. A.* **1988**, *38*, 3098-3100.

24. Perdew, J. P. Density-functional approximation for the correlation energy of the inhomogeneous electron gas. *Phys. Rev. B.* **1986**, *33*, 8822-8824.

25. Glendening, E. D.; Badenhoop, J. K.; Reed, A. E.; Carpenter, J. E.; Bohmann, J. A.; Morales, C. M.; Landis, C. R.; Weinhold, F. (Theoretical Chemistry Institute, University of Wisconsin, Madison, WI, 2013); http://nbo6.chem.wisc.edu/.

26. Bader, R. F. W. *Atoms in Molecules: A Quantum Theory*, Oxford University Press, New York, 1990.

27. Bader, R. F. W. A bond path: a universal indicator of bonded interactions. *J. Phys. Chem. A* **1998**, *102*, 7314-7323.

28. Roos, B. O. The complete active space SCF method in a fock-matrix-based super-CI formulation. *Int. J. Quantum Chem*. **1980**, *14*, 175-189.

29. Malmqvist, P.-Å.; Roos, B. O. The restricted active space self-consistent-field method, implemented with a split graph unitary group approach. *J Phys. Chem*. **1990**, *94*, 5477-5482.

30. Li Manni, G.; Fdez. Galván, I.; Alavi, A.; Aleotti, F.; Aquilante, F.; Autschbach, J.; Avagliano, D.; Baiardi, A.; Bao, J. J.; Battaglia, S.; Birnoschi, L.; Blanco-González, A.; Bokarev, S. I.; Broer, R.; Cacciari, R.; Calio, P. B.; Carlson, R. K.; Couto, R. C.; Cerdán, L.; Chibotaru, L. F.;

Chilton, N. F.; Church, J. R.; Conti, I.; Coriani, S.; Cuéllar-Zuquin, J.; Daoud, R. E.; Dattani, N.; Decleva, P.; de Graaf, C.; Delcey, M. G.; De Vico, L.; Dobrautz, W.; Dong, S. S.; Feng, R.; Ferré, N.; Filatov, M.; Gagliardi, L.; Garavelli, M.; González, L.; Guan, Y.; Guo, M.; Hennefarth, M. R.; Hermes, M. R.; Hoyer, C. E.; Huix-Rotllant, M.; Jaiswal, V. K.; Kaiser, A.; Kaliakin, D. S.; Khamesian, M.; King, D. S.; Kochetov, V.; Krośnicki, M.; Kumaar, A. A.; Larsson, E. D.; Lehtola, S.; Lepetit, M. -B.; Lischka, H.; López Ríos, P.; Lundberg, M.; Ma, D.; Mai, S.; Marquetand, P.; Merritt, I. C. D.; Montorsi, F.; Mörchen, M.; Nenov, A.; Nguyen, V. H. A.; Nishimoto, Y.; Oakley, M. S.; Olivucci, M.; Oppel, M.; Padula, D.; Pandharkar, R.; Phung, Q. M.; Plasser, F.; Raggi, G.; Rebolini, E.; Reiher, M.; Rivalta, I.; Roca-Sanjuán, D.; Romig, T.; Safari, A. A.; Sánchez-Mansilla, A.; Sand, A. N.; Schapiro, I.; Scott, T. R.; Segarra-Martí, J.; Segatta, F.; Sergentu, D. -C.; Sharma, P.; Shepard, R.; Shu, Y.; Staab, J. K.; Straatsma, T. P.; Sørensen, L. K.; Tenorio, B. N. C.; Truhlar, D. G.; Ungur, L.; Vacher, M.; Veryazov, V.; Voß, T. A.; Weser, O.; Wu, D.; Yang, X.; Yarkony, D.; Zhou, C.; Zobel, J. P.; Lindh, R. The OpenMolcas *Web*: A community-driven approach to advancing computational chemistry. *J. Chem. Theory Comput*. (published online 2023).

31. Douglas, M.; Kroll, N. M. Quantum electrodynamical corrections to the fine structure of helium. *Ann. Phys*. **1974**, *155*, 82-89.
32. Hess, B. A. Relativistic electron-structure calculations employing a two-component no-pair formalism with external-field projection operators. *Phys. Rev. A* **1986**, *33*, 3742-3748.
33. Malmqvist, P. -Å.; Roos, B. O.; Schimmelpfennig, B. The restricted active space (RAS) state interaction approach with spin-orbit coupling. *Chem. Phys. Lett*. **2002**, *357*, 230-240.
34. Roos, B. O.; Malmqvist, P. -Å. Relativistic quantum chemistry: the multiconfigurational approach. *Phys. Chem. Chem. Phys*. **2004**, *6*, 2919-2927.
35. Roos, B. O.; Veryazov, V.; Widmark, P. O. Relativistic atomic natural orbital type basis sets for the alkaline and alkaline-earth atoms applied to the ground-state potential for the corresponding dimers. *Theor. Chem. Acc.* **2004**, *111*, 345-351.

36. Roos, B. O.; Lindh, R.; Malmqvist, P. -Å.; Veryazov, V.; Widmark, P. O. Main group atoms and dimers studied with a new relativistic ANO basis set. *J. Phys. Chem. A* **2004**, *108*, 2851-2858.

37. Roos, B. O.; Lindh, R.; Malmqvist, P. -Å.; Veryazov, V.; Widmark, P. O. New relativistic ANO basis sets for actinide atoms. *Chem. Phys. Lett.* **2005**, *409*, 295-299.

38. Finley, J.; Malmqvist, P. -Å.; Roos, B. O.; Serrano-Andrés, L. The multi-state CASPT2 method. *Chem. Phys. Lett.* **1998**, *228*, 299-306.

39. Shiozaki, T.; Györffy, W.; Celani, P.; Werner, H.-J. Communication: Extended multi-state complete active space second-order perturbation theory: Energy and nuclear gradients. *J. Chem. Phys.* **2011**, *135*, 081106.

40. Granovsky, A. A. Extended multi-configuration quasi-degenerate perturbation theory: The new approach to multi-state multi-reference perturbation theory. *J. Chem. Phys*. **2011**, *134*, 214113.

41. Molcas Suite, available at: https://pypi.org/project/molcas-suite/.

42. Goodwin, C. A. P.; Wooles, A. J.; Murillo, J.; Lu, E.; Boronski, J. T.; Scott, B. L. Gaunt, A. J.; Liddle, S. T. Carbene Complexes of Neptunium. *J. Am. Chem. Soc.* **2022**, *144*, 9764-9774.
